# The RNA-binding protein YTHDF3 affects gastric cancer cell migration and response to paclitaxel by regulating EZRIN

**DOI:** 10.1007/s10120-025-01620-y

**Published:** 2025-05-14

**Authors:** Patrícia Mesquita, Alexandre Coelho, Ana S. Ribeiro, Luís F. C. Póvoas, Catarina de Oliveira, Nelson Leça, Sara Silva, Diana Ferreira, Diana Pádua, Ricardo Coelho, Carmen Jerónimo, Joana Paredes, Carlos Conde, Bruno Pereira, Raquel Almeida

**Affiliations:** 1 i3S - Institute for Research and Innovation in Health, University of Porto, 4200-135 Porto, Portugal; 2 IPATIMUP - Institute of Molecular Pathology and Immunology, University of Porto, 4200-465 Porto, Portugal; 3 ICBAS – School of Medicine and Biomedical Sciences, University of Porto, 4050-313 Porto, Portugal; 4 Biology Department, Faculty of Sciences, University of Porto, 4169-007 Porto, Portugal; 5 IBMC - Institute of Molecular and Cell Biology, University of Porto, 4200-135 Porto, Portugal; 6 Ovarian Cancer Research, Department of Biomedicine, University Hospital Basel and University of Basel, 4031 Basel, Switzerland; 7 Cancer Biology and Epigenetics Group, Research Center of IPO Porto (CI-IPOP)/RISE@CI-IPOP (Health Research Network), Portuguese Oncology Institute of Porto (IPO Porto)/Porto Comprehensive Cancer Center Raquel Seruca (Porto.CCC Raquel Seruca), 4200-072 Porto, Portugal; 8 Pathology Department, Faculty of Medicine, University of Porto, 4200-319 Porto, Portugal

**Keywords:** YTHDF3, EZRIN, N6-methyladenosine, Wound healing, Paclitaxel

## Abstract

**Background:**

Gastric cancer (GC) is the fourth most common cause of cancer-related mortality and the fifth most common cancer worldwide. Despite efforts, the identification of biomarkers and new therapeutic approaches for GC remains elusive. Recent studies have begun to reveal the role of N6-adenosine methylation (m^6^A) in the regulation of gene expression.

**Methods:**

The expression of the reader YT521-B homology domain-containing family 3 (YTHDF3) in GC was assessed in 331 patients using immunohistochemistry. GC cell lines depleted of YTHDF3 using CRISPR-Cas9 were evaluated for migration, metastasis, orientation of the mitotic spindle, and response to paclitaxel. The association between YTHDF3 and *EZRIN* (*EZR*) mRNA was shown using RNA sequencing, immunofluorescence, real-time PCR, and RNA immunoprecipitation. The single-base elongation- and ligation-based qPCR amplification (SELECT) method was used to map m^6^A in the *EZR* transcript.

**Results:**

YTHDF3 was significantly overexpressed in GC, and high levels of YTHDF3 were predictive of the response to chemotherapy. In GC cell lines, YTHDF3 was the most highly expressed reader protein. YTHDF3 depletion impaired cytoskeleton organization, cell migration and metastasis, and orientation of the mitotic spindle, leading to an increased response to paclitaxel. *EZR* was one of the downregulated targets in the *YTHDF3* knockout cell models and was associated with the observed phenotype.

**Conclusion:**

YTHDF3 contributes to cell motility and response to paclitaxel in GC cell lines, at least in part through EZR regulation. The YTHDF3–EZR regulatory axis is a novel molecular player in GC, with clinical relevance and potential therapeutic utility.

**Supplementary Information:**

The online version contains supplementary material available at 10.1007/s10120-025-01620-y.

## Introduction

Gastric cancer (GC) remains one of the most commonly diagnosed cancers and is the fourth leading cause of cancer mortality worldwide, with a 5-year survival rate of less than 35% [1, 2]. Thus, a better understanding of this disease is urgently needed to improve its diagnosis and treatment.

RNA molecules, including mRNAs, can be chemically modified, which contributes to their functional diversity. More than 170 chemical modifications of RNA are currently known and are collectively referred to as the epitranscriptome [3, 4]. The most prevalent chemical modification of mRNA is the methylation of adenosine at the nitrogen-6 position (m^6^A) [5]. This dynamic and reversible modification is catalyzed by a m^6^A methyltransferase protein complex that consists of methyltransferase-like protein 3 (METTL3) and METTL14 along with Wilms tumor 1-associated protein (WTAP) and vir-like m^6^A methyltransferase associated (VIRMA), known as writers [3]. After deposition, m^6^A marks can be removed by RNA demethylases (erasers), which include the enzymes FTO alpha-ketoglutarate-dependent dioxygenase (FTO) and alkB homologue 5 RNA demethylase (ALKBH5). m^6^A marks are recognized by RNA-binding proteins (RBPs), which are known as readers that bind m^6^A-modified mRNAs and, in this way, influence their fate in splicing, nuclear export, localization, stability, and translation [3]. Proteins belonging to the YT521-B homology domain-containing family (YTHDF), comprising the highly homologous YTHDF1, -2, and -3, are the major m^6^A readers identified to date and provide a mechanistic basis for understanding the effects of m^6^A in cells [5, 6].

m^6^A modification and the proteins involved in its deposition are associated with various cancers [7–9]. In GC, m^6^A modifications are associated with a more aggressive phenotype in vitro [9–11], and METTL3 expression in tumors is associated with poor patient prognosis [10, 12]. However, gaps remain in the current knowledge regarding the role of m^6^A in GC, particularly concerning the expression and function of reader proteins.

The goal of this study was to elucidate the role of m^6^A methylation and YTHDF proteins in GC biology. Initial bioinformatic analyses revealed that *YTHDF3* was potentially the most differentially expressed reader in GC compared with normal gastric mucosa. Thus, this study focused on understanding the role of YTHDF3 in GC using cellular models modified for *YTHDF3* expression through a CRISPR-Cas9 approach.

## Materials and methods

### Patient samples

Cohort I was obtained from the GEPIA 2 database (http://gepia2.cancer-pku.cn/#index). Cohort II included surgical specimens from consecutive patients with GC who were surgically treated between January 2008 and December 2014 at Centro Hospitalar São João (CHSJ), Porto, Portugal. Tumor tissue, clinicopathological and treatment data, and follow-up information were available for all patients (*n* = 331). All samples were included in the biobank at CHSJ, and the study was approved by the Ethics Committee of CHSJ (CES 122/15). Relevant clinical information on this series has been previously provided [13, 14].

### Immunohistochemistry

Immunohistochemistry (IHC) against YTHDF3 was performed in the GC samples included in the previously obtained tissue microarrays [13]. The samples were incubated with primary antibody against YTHDF3 (1:100 dilution, ab103328, Abcam, Cambridge, UK) overnight (O/N) at 4 °C and the procedure was as previously described [13, 14]. Samples were considered YTHDF3-Low when they were negative for YTHDF3 or when less than 20% of the malignant cells showed expression. Otherwise, the sample was considered YTHDF3-High. Classification required agreement among three observers.

### Cell line culture

AGS, MKN28, MKN45, and SNU638 cells were cultured in RPMI-1640 + GlutaMAX supplemented with 10% fetal bovine serum (FBS). KATO III cells were cultured in RPMI-1640 + GlutaMAX supplemented with 15% FBS. GP202 cells were cultured in Dulbecco’s modified Eagle’s medium (DMEM) supplemented with 10% FBS. All human cell lines were cultured at 37 °C in a 5% (v/v) CO_2_ humidified atmosphere and were regularly tested for mycoplasma.

### Western blot

The cell pellets were rinsed with phosphate-buffered saline (PBS) at 4 °C and resuspended in RIPA buffer supplemented with a protease inhibitor cocktail containing 1mM PMSF, 1mM Na_3_VO_4_, 20nM NaF (Roche, Basel, Switzerland) and 1x Complete (Roche Applied Science) for 30 min on ice. After centrifugation at 16000×g and 4 °C for 15 min, the supernatants were collected for protein quantification using the bicinchoninic acid (BCA) method. The samples were denatured in Laemmli buffer at 95 °C for 5min, separated by SDS-PAGE, transferred to a nitrocellulose membrane, and probed with appropriate antibodies O/N at 4 °C. The primary antibodies used included anti-YTHDF3 (ab220161, Abcam, Cambridge, UK), diluted 1:1000; anti-METTL3 (ab195352, Abcam, Cambridge, UK), diluted 1:1000; anti-YTHDF1 (ab252346, Abcam, Cambridge, UK), diluted 1:2500; and anti-YTHDF2 (ab220163, Abcam, Cambridge, UK), diluted 1:2500; anti-EZR (3145S, Cell Signaling Technology, Danvers, MA, USA), diluted 1:1000; and anti-β-actin (sc-47778, Santa Cruz Biotechnology, Dallas, TX, USA), diluted 1:900. Afterwards, the membranes were washed with TBS-1% Tween-20 (Sigma‒Aldrich, St. Louis, MO, USA) (3 × 10 min each), incubated with a suitable horseradish peroxidase-conjugated secondary antibody for 1 h at room temperature (RT) and resolved using an enhanced chemiluminescence (ECL) substrate. The bands were detected and quantified via a ChemiDoc imaging system using ImageLab 6.0.1 (Bio-Rad, Hercules, CA, USA).

### RNA extraction and real-time PCR (qRT‒PCR)

Total RNA was extracted using TRI Reagent (Sigma‒Aldrich, St. Louis, MO, USA) according to the manufacturer’s protocol. RNA concentration and quality were assessed using a NanoDrop ND-1000 spectrometer (V3.5.2 Software, Thermo Fisher Scientific, Waltham, MA, USA). Total RNA was reverse transcribed to cDNA in two steps. First, by heating for 10 min at 65 °C, a mixture containing 1 μg of total RNA and 100 ng of random primers was produced in a final reaction volume of 13 μL. Second, 1x SuperScript Buffer, 5mM DTT, 0.5mM dNTPs, 0.6U/µL RNAseOUT RNase Inhibitor, and 5U/µL SuperScript III Reverse Transcriptase in DEPC-treated water were added, resulting in a final reaction volume of 20 μL. This mixture was incubated first at 25 °C for 5min, then at 50 °C for 30–60min, and finally at 70 °C for 15min. For PCR, each reaction was prepared with 4 μL of cDNA diluted 1:20 in DEPC-treated water, 10 μL of Power SYBR Green PCR Master Mix (Thermo Fisher Scientific, Waltham, MA, USA), 0.6 μL of each primer (10μM), and 4.8 μL of DEPC-treated water. The primers used are listed in Supplementary Table 1. The reactions were performed in a 7500 Fast Real-Time PCR System using the software v2.0.6 (Thermo Fisher Scientific, Waltham, MA, USA). Each experiment was performed in triplicate, and three negative controls (without cDNA) were included in each plate. The housekeeping control gene *18S* was used to normalize target gene abundance. The data were analyzed using the comparative 2^-ΔΔCT^ method [15].

### CRISPR-*Cas9-*mediated YTHDF3 knockout cell generation

AGS and SNU638 knockout (KO) cell lines for YTHDF3 were generated using a previously published protocol [16, 17]. Briefly, a pair of single-guide (sg)RNAs (sgRNA1:5’-TGGGTAGCTCCTCGTAACAG-3,’ MIT score 95% and sgRNA2:5’-CTATATTCTTACCCTACGCA-3,’ MIT score 91%) flanking the 601bp genomic region that codifies the functional YTH domain were designed using the UCSC Human Genome Browser software [18]. Guide RNAs were designed to target all potential protein isoforms. The forward and reverse oligos were annealed and cloned into pSpCas9 (BB)-2A-GFP (#PX458, Addgene, Watertown, MA, USA) using the *BsbI* restriction site and T4-DNA ligase, transformed into Stbl3 competent *Escherichia coli* and sequenced for confirmation. AGS and SNU638 cell lines were transiently transfected with plasmids harboring the sgRNAs targeting *YTHDF3* using Lipofectamine (Thermo Fisher Scientific, Waltham, MA, USA) and incubated at 37 °C in a 5% CO_2_ humidified atmosphere for 72 h. Single-cell sorting was performed using BD FACS Aria II Cell Sorter (BD Biosciences, California, CA, USA) to separate the GFP^+^ cells into 96-well flat-bottom plates with RPMI 1640+GlutaMAX supplemented with 10% FBS and 100µg/mL Primocin (InvivoGen, San Diego, CA, USA), which were subsequently incubated for 3 weeks. Clones harboring homozygous *YTHDF3* deletions (designated ∆*YTHDF3*) were identified on the basis of two genotyping PCRs using the primers indicated in Supplementary Table 1. All homozygous KO clones were further validated with DNA sequencing and western blotting.

### Hypoxia assay

Hypoxia was assessed using two methods. A 0.1M cobalt chloride (CoCl_2_) solution was used to induce hypoxia in the AGS and SNU638 cell lines and the corresponding ∆*YTHDF3* clones [19]. The cells were plated in 24-well plates (5 × 10^4^ cells per well) and treated the next day for 24 h or 48 h with different concentrations of CoCl_2_ (AGS: 0, 50, 100, 200, or 300 μM; SNU638: 0, 50, 100, or 200 μM). Hypoxia was also assessed by incubating cells in a hypoxia chamber (1% O_2_) during 72 h. For both, cell viability was assessed using the PrestoBlue Cell Viability Reagent (Thermo Fisher Scientific, Waltham, MA, USA) according to the manufacturer’s instructions.

### Treatment with chemotherapeutic drugs

To evaluate the impact of YTHDF3 on the response to 5-FU, cisplatin and paclitaxel (PTX) 1 × 10^4^ mock and KO cells/well were seeded into 96-well plates in complete media and incubated for 24 h before treatment with DMSO, 4.5 μg/mL 5-FU, 6 μg/mL cisplatin or different concentrations of PTX. Cell viability was measured using a PrestoBlueTM Cell Viability assay (Thermo Fisher Scientific, Waltham, MA, USA) according to the manufacturer’s instructions.

### Modulation of EZRIN expression

NSC668394 (Merck KGaA, Darmstadt, Germany), a known pharmacological inhibitor of PKCι-mediated EZR phosphorylation at Thr567 and EZR-actin binding, was used to inhibit EZR activity. The cells were treated with NSC668394 at 1 or 2 μM, for 24–48 h, depending on the experimental requirements. The control cells were treated with an equivalent volume of DMSO. EZR downregulation and overexpression were performed using esiRNAs (Merck KGaA, Darmstadt, Germany) and pCMV-EZR expression vector (Addgene) transfected with Lipofectamine 2000 (Thermo Fisher Scientific, Waltham, MA, USA). esiRNAs against GFP and empty vector were used as controls, respectively.

### Wound healing assay

5 × 10^4^ AGS mock and AGS Δ*YTHDF3* cells per well and 1 × 10^5^ SNU638 mock and SNU638 Δ*YTHDF3* cells were seeded in an Ibidi Culture-Insert 2 Well system (Ibidi, Gräfelfing, Germany) and maintained for 24 h at 37 °C and 5% CO_2_ to allow cell adhesion. Then the culture inserts were removed, and the cells were rinsed with PBS and incubated with fresh RPMI-1640 + GlutaMAX supplemented with 5% FBS for 24 h. During this period, images of the wounds were captured every 30 min with a 10x objective under phase contrast in a Leica DMI6000 Timelapse microscope (Leica, Wetzlar, Germany) at 37 °C and 5% CO_2_. ImageJ software was used to determine the percentage of the wound area closed, and two-way ANOVA was performed to assess statistical significance. Three wounds were sampled for each condition, and the experiments were performed in triplicate.

### Transwell invasion assay

5 × 10^4^ cells were seeded in the upper compartment of the Transwell Matrigel-coated chambers with 8 µm pore-size membranes (Corning) in 500 µL of RPMI supplemented with 1% FBS. In the lower compartment, 750 µL of RPMI containing 10% FBS was added as a chemoattractant. Following 24 h incubation, non-invasive cells were removed with a cotton swab, whereas invasive cells were fixed in ice-cold methanol for 10 min. The membranes were cut and mounted with Vectashield (Vector) with DAPI. The number of invasive cells was counted after image acquisition using the PhenoImager HT (Akoya Biosciences).

### Lung metastasis assay in mice

SNU638 cells (1 × 10^5^), either mock or ΔYTHDF3, were injected via the tail vein of 6–8-week-old NIH(S)II-nu/nu mice (5 mice per condition). The weight and well-being of the mice were evaluated twice a week for a maximum of 8 weeks after tumor cell inoculation. At this point, the mice were euthanized, and the lungs were harvested and fixed in 10% buffered formaldehyde for up to 48 h for further histological assessment of the metastatic burden. The lungs were serially sectioned at 50-μm intervals for comprehensive analysis, and the presence of metastasis was assessed in H&E-stained slides by two independent observers (RA and ASR). To quantify metastatic foci and metastatic burden, whole-slide images of lung sections were acquired using the PhenoImager HT (Akoya Biosciences) following H&E staining, and image analysis was performed using the QuPath software (version 0.5.1). The total tissue area was delineated using QuPath’s automated tissue detection tool, followed by manual refinement to exclude non-pulmonary parenchymal structures. Metastatic foci were identified manually through annotation. To ensure consistency, one representative slide per mouse was selected at comparable depth across samples. All experiments were conducted with the application of the 3Rs (replacement, reduction, and refinement) (JP_2016_02 Project, animal ethics committee, and animal welfare body of i3S).

### Immunofluorescence

The cells were grown for 48 h on glass coverslips coated with fibronectin. Coverslips were washed with ice-cold PBS, fixed with 4% paraformaldehyde (PFA) in PBS, permeabilized with 0.25% v/v Triton X-100 in PBS, blocked with serum from the same species where the secondary antibody was produced, and diluted 1:5 in PBS + 0.5% Tween-20 and 0.05% BSA for 30 min. Fixed cells were then incubated with the primary antibody anti-EZR (1:200, 3145S, Cell Signaling Technology, Danvers, MA, USA) or anti-(Thr 567) phospho-EZR (1:200, PA5-117400, Thermo Fisher Scientific, Waltham, MA, USA) O/N at 4 °C, followed by probing with an Alexa Fluor-conjugated secondary antibody for 45 min at RT in the dark. In some experiments, after washing with PBS, the cells were incubated with Actin Red 555 Ready Probes reagent (R37112, Thermo Fisher Scientific, Waltham, MA, USA) for 30 min at RT in the dark to stain F-actin. Finally, the coverslips were stained with DAPI for 5 min and mounted on microscope slides using Vectashield Antifade Mounting Medium (Vector Laboratories, Inc., Newark, CA, USA). Images were acquired using a Leica SP5II laser scanning confocal microscope (Leica Microsystems, Wetzlar, Germany).

### RNA sequencing (RNA-seq)

Three paired replicates of mock and Δ*YTHDF3* SNU638 cells were used for RNA extraction and sequencing. Total RNA extraction was performed using the PureLink RNA Mini Kit (Thermo Fisher Scientific, Waltham, MA, USA), according to the manufacturer’s instructions. Quantification and quality control of total RNA were assessed using the NanoDrop ND-1000 and 2100 Bioanalyzer (Agilent Technologies, Santa Clara, CA, USA) systems, and only samples with an RNA integrity number above eight were considered for further study. Preparation of the RNA library, transcriptome sequencing, and bioinformatic analysis were outsourced to Novogene (Cambridge, UK). Genes with adjusted *p* values < 0.05 and log2 (fold-change) > 0.58 were considered differentially expressed. Gene Ontology (GO) and Kyoto Encyclopedia of Genes and Genomes (KEGG) pathway analyses were performed using the EnrichR bioinformatics database [20]. The RNA-seq data were deposited in the Gene Expression Omnibus (GEO) database (https://www.ncbi.nlm.nih.gov/geo/).

### RNA immunoprecipitation (RIP) assay

Cultured cells were washed with PBS and subjected to ultraviolet crosslinking (254nm wavelength) at an energy level of 0.5J/cm^2^ on ice using a UV crosslinker. Cell lysis was performed with a cell scraper in NP-40 lysis buffer (20mM Tris–HCl pH 7.5, 150mM NaCl, 2mM EDTA, and 1% IGEPAL) supplemented with a protease inhibitor cocktail containing 1mM PMSF, 1mM Na_3_VO_4_, 20mM NaF, 1× Complete and 20U/mL RNase OUT ribonuclease inhibitor. After incubation for 30 min on ice, the extracts were centrifuged at 16000×g and 4 °C for 15min, and the supernatants were collected for protein quantification as previously indicated. Five micrograms of anti-YTHDF3 antibody or normal rabbit IgG were incubated with 750 mg of total protein extracts O/N at 4 °C with rotation. Protein‒RNA complexes were recovered on the following day by incubation with 50µL of Dynabeads Protein G for 2 h at 4 °C with rotation. After three washes in lysis buffer containing RNAseOUT, RNA was extracted from the beads with TRI Reagent and used for qRT‒PCR, as previously described. *EZR* mRNA levels were normalized against the housekeeping control *GAPDH* mRNA.

### Single-base elongation- and ligation-based qPCR amplification method (SELECT)

mRNA was purified using the PureLink RNA Mini Kit and DNase treatment. Then 20 ng of mRNA was mixed with 40nM Up and Down primers for each of the predicted m^6^A sites (positions 1903 and 2161 of the *EZR* transcript - NM_003379.5) and, as a control, primers designed for a nearby site known to be unmethylated and located 4 bases upstream of each of these two sites following the protocol described by Ref. [21]. Finally, 5µM dNTPs and 17µL of CutSmart buffer 1x were added and incubated for 6 min, following a gradient of temperatures: 90 °C per 1 min, 80 °C per 1 min, 70 °C per 1 min, 60 °C per 1 min, 50 °C per 1 min and 40 °C for 1 min. Next, 3 µL of a mixture containing 0.01U of Bst DNA polymerase, 0.5U of SplintR ligase, and 10nmol of ATP was added in a total volume of 20 µL. The mixture was incubated at a gradient of temperatures: 40 °C for 20 min and 80 °C for 20 min and finally maintained at 4 °C. qRT‒PCR was conducted using 4 µL of the SELECT reaction mixture obtained for each putative m^6^A site with the one obtained for the control, and we finally compared the respective CTs.

### Characterization of mitotic spindle orientation

The cells were plated on fibronectin-coated coverslips, and immunofluorescence was performed as described above. Spindle microtubules, centrosomes, and F-actin were stained with an anti-β-tubulin antibody (ab6046; Abcam, Cambridge, UK), anti-γ-tubulin antibody GTU-88 (ab11316; Abcam, Cambridge, UK), and Phalloidin CruzFluor™ 647 conjugate (sc-363797; Santa Cruz Biotechnology, Dallas, TX, USA), respectively. Images were acquired using a Leica SP8 laser scanning confocal microscope (Leica Microsystems, Bucks, UK) with an HC PL APO 63×/1.30 glycerol immersion objective and a digital zoom factor of 5. Metaphase fixed cells were imaged in *x*‒*y* optical sections passing through the spindle poles, with a *z* step of 0.3 µm with at least 20 sections, ensuring that the full cell was imaged. Orthogonal projections were created using 3D Project Fiji’s tool. The spindle orientation angles with respect to the fibronectin substrate were determined using Fiji’s angle tool, with the substrate as the reference line (0°). Statistical analysis of data from three independent experiments was performed using Prism with the Mann‒Whitney test. The results are presented in a bar graph showing the median and interquartile range, where 0° represents the substrate axis and 90° represents a perpendicular orientation to the coverslip.

### Chick embryo chorioallantoic membrane (CAM) assay

Eggs were prepared (window opening) at embryonic development day (EDD)3, to allow the growth of the CAM detached from the shell. At EDD9, and under sterile conditions, each CAM was inoculated with 1 × 10^6^ SNU638 mock and 1 × 10^6^ SNU638 ΔYTHDF3 cells in two distinct inoculation sites limited by silicone rings, and resuspended 1:1 in 5 μL of Matrigel and 5 μL of serum-free culture medium. At EDD12, eggs were randomly distributed for topic treatment with PTX (10 μL at 10nM—21 eggs for each cell line) and DMSO (vehicle—16 eggs for each cell line). The experiments ended at EDD14. At the endpoint, CAMs were fixed (10% neutral-buffered formalin), excised from the embryo and photographed *ex ovo*. The pictures were used to determine the tumor size, as described previously [22].

### Statistical analysis

To evaluate the associations between the expression status of YTHDF3 and the clinicopathological features of the tumors, different statistical tests were performed. To determine the associations with patient age, we used Student’s *t* test. For sex and vascular invasion, we applied Fisher’s exact test (two-sided). For the Lauren classification and tumor node metastasis (TNM) staging, we used the Chi-square test (χ^2^). To investigate the association between YTHDF3 expression status and the risk of death, the Kaplan‒Meier method was used to generate plots and survival curves for 5-year overall survival (OS: time from operation to death from any cause), which were compared using the log-rank test. Statistical analysis was performed using IBM SPSS Statistics version 24 (IBM Corporation, Armonk, NY, USA). Differences were considered statistically significant when the *p* value was less than 0.05.

## Results

### YTHDF3 is expressed in GC and predicts therapeutic response

**Fig. 1:**
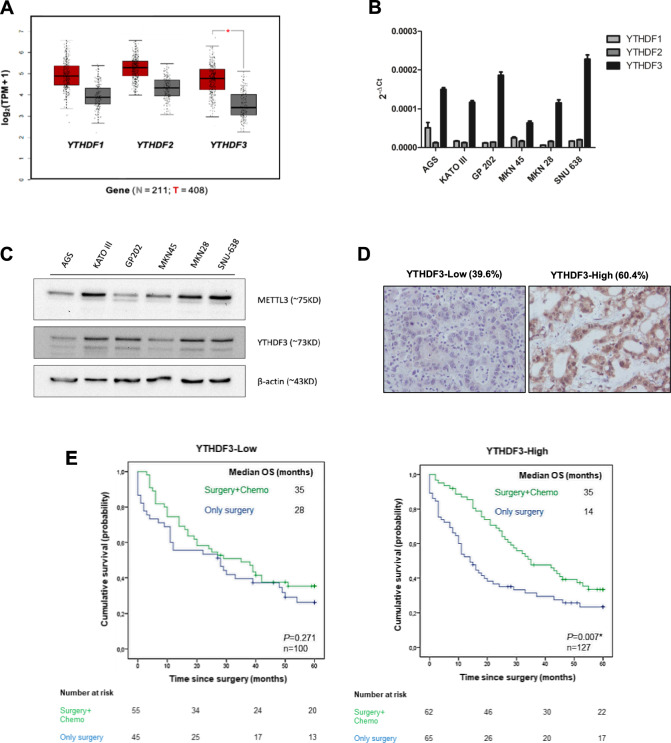
Expression of YTHDF1-3 “reader” proteins in GC. **A** Expression of YTHDF1-3 was compared between normal gastric mucosa (N; *n* = 211, in grey) and GC tissues (T; *n* = 408, in red), using data deposited in the TCGA and GTEx databases (TPM: transcript per kilobase million). Comparison was performed using the free online software GEPIA (http://gepia2.cancer-pku.cn). Results were considered significant when *p < 0.01. **B** qRT-PCR analysis of *YTHDF1-3* mRNA expression in a panel of GC cell lines. **C** Protein expression of METTL3 and YTHDF3 in the same panel. **D** Representative images of low and high YTHDF3 expression in two different GC cases from a 331-patient series (20× objective). **E** Kaplan–Meier curves showing the probability of OS according to treatment options in patients having low and high expression of YTHDF3, respectively. Median OS is shown for each subgroup of patients. The number of patients at risk is specified for 0, 20, 40, and 60 months. *p < 0.05

To assess the relevance of m^6^A reader proteins in GC, we analyzed their expression in comparison with that in normal gastric mucosa through bioinformatics analysis of public databases (TCGA and GTEx). The mRNA expression levels of all three readers analyzed were greater in GC than in normal gastric mucosa; however, *YTHDF3* was the only one whose expression was significantly different (Fig. 1A). Next, we evaluated *YTHDF1-3* expression in a panel of commonly used GC cell lines. *YTHDF3* was the most abundantly expressed reader in all the cell lines tested (Fig. 1B), reinforcing the relevance of this specific RBP in GC. We also analyzed the expression of YTHDF3 and METTL3 using western blotting in the same GC cell line panel and observed that all these proteins were expressed, albeit at different levels, suggesting the presence of an active m^6^A machinery (Fig. 1C). To validate TCGA mRNA data in GC tissues, we evaluated the expression of the YTHDF3 protein in a series of 331 GC patients using immunohistochemistry. YTHDF3 exhibited mainly cytoplasmic localization, which was classified as high in 60.4% of the cases and low in 39.6% of the cases (Fig. 1D; Supplementary Table 2). The clinicopathological features of the 331 GC patients and their associations with YTHDF3 expression are summarized in Supplementary Table 2. High YTHDF3 levels were significantly associated with tumors classified as intestinal (64.1% of the cases) or mixed (65.9% of the cases), according to the Lauren classification (*p *< 0.001), compared with diffuse-type tumors (only 33.3% of the cases). Other clinicopathological parameters, such as age at diagnosis, sex, TNM stage, and vascular invasion, were not significantly associated with YTHDF3 expression. Next, we assessed whether YTHDF3 was a prognostic marker of patient survival in our series of GC patients. Kaplan–Meier analysis revealed that YTHDF3 was not a prognostic indicator in GC, as patients exhibited similar OS rates irrespective of YTHDF3 expression levels (Supplementary Fig. 1A). However, when we stratified patients on the basis of whether they received adjuvant chemotherapy (stages II, III and IV), the results were different (Fig. 1E). Considering patients treated with surgery alone, those with low YTHDF3 expression clearly had a better median OS than those with high YTHDF3 expression (28 vs. 14 months, respectively). When adjuvant chemotherapy was added to the treatment, OS improved to a median of 35 months in both groups. However, the benefit from adjuvant chemotherapy was significant only in the high-expression group, which indicates the clinical relevance of YTHDF3 as a biomarker of therapy response. When further stratifying for stage, YTHDF3 did not appear to be a significant marker of therapy response in patients with stage II tumors, although it exhibited the same tendency (Supplementary Fig. 1B and 1C). However, YTHDF3 was very significant in patients with stage III and stage IV tumors (Supplementary Fig. 1D-G).

### Δ*YTHDF3* clones presented altered cell morphology

**Fig. 2:**
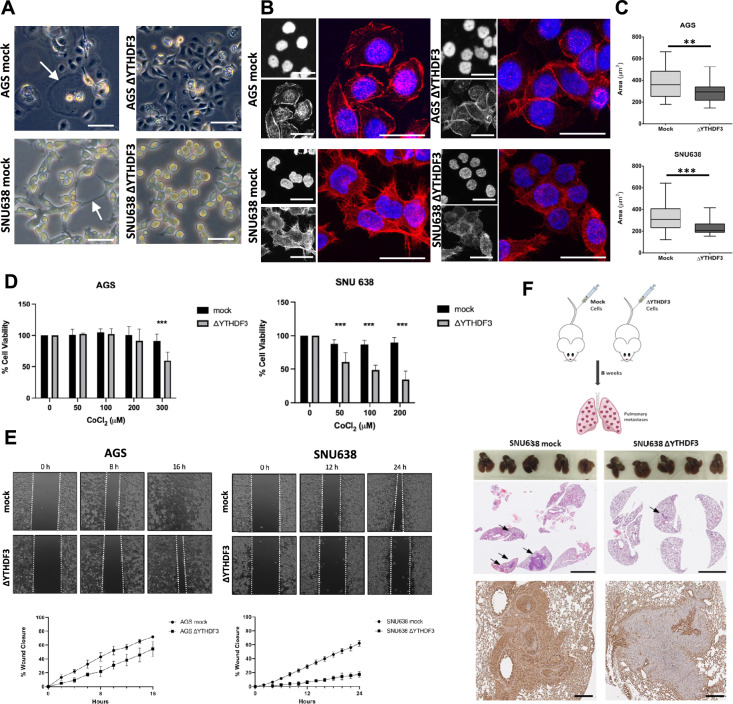
Phenotypic alterations observed after *YTHDF3* deletion in GC cell lines. **A** Morphology of AGS and SNU638—either mock or Δ*YTHDF3* cells. Giant cells in AGS mock and filopodia and lamellipodia protrusions in SNU638 mock cells are indicated with white arrows. Scale bar corresponds to 25 μm and images were obtained with a 40x objective. **B** Immunofluorescence staining actin filaments (F-actin), visualized with confocal microscopy. Nuclei are stained with DAPI. Scale bar corresponds to 25 μm and images were obtained with a 40x objective. **C** AGS and SNU638 Mock and Δ*YTHDF3* cells area was quantified using ImageJ software (*n* = 30, each). Data are represented in a box-and-whisker plot as mean (middle line) with the minimum and maximum distribution values. AGS Δ*YTHDF3* vs Mock, **p < 0.01; SNU638 Δ*YTHDF3* vs Mock, ***p < 0.001; unpaired Student's *t* test. **D** Cell viability in response to hypoxia (mimetized by CoCl_2_) measured in the same cell lines. ***p < 0.001. **E** Wound healing assay. Representative images of the wound closure, at different time points in the upper panels. Graphs indicate average and standard deviation (SD) of % of wound closure every 2 h. ANOVA test was performed and statistical significance was obtained for all timepoints (*p *< 0.001). **F** Representative images of the lungs after cell line injection. Below is a representative H&E from each condition. Scale bar corresponds to 4 mm. The bottom panel shows YTHDF3 expression in each condition. Scale bar corresponds to 400 μm

We used CRISPR-Cas9 to perform loss-of-function experiments of the reader YTHDF3 in two GC cell lines, AGS and SNU638, which presented different basal expression levels (strategy depicted in Supplementary Fig. 2A)*.* Loss of YTHDF3 expression in the KO clones from the designated AGS Δ*YTHDF3* and SNU638 Δ*YTHDF3* strains was verified using western blotting (Supplementary Fig. 2B). Furthermore, we confirmed that both the KO clones and the parental cell lines maintained the same m^6^A levels (Supplementary Fig. 2C). We confirmed that there was no significant alteration in the expression of YTHDF1 or YTHDF2 in the Δ*YTHDF3* clones (Supplementary Fig. 2D and 2E). With respect to the phenotypic characteristics of the mutant cell lines, the Δ*YTHDF3* clones exhibited a visible alteration in morphology. AGS Δ*YTHDF3* exhibited a decrease in giant cells (Fig. 2A), which is characteristic of the parental AGS cell line. SNU638 Δ*YTHDF3* presented a decrease in membrane protrusions and a rounder appearance. F-actin staining revealed a different and less organized actin cytoskeleton, with fewer filopodia and lamellipodia structures in the KO cells (Fig. 2B). There was a clear change in the size of the Δ*YTHDF3* cells in both cell lines (Fig. 2C).

### Δ*YTHDF3* clones presented decreased viability under hypoxia

Next, we assessed whether *YTHDF3* deletion affects cell viability. Under standard cell culture conditions, *YTHDF3* deletion did not significantly affect the viability of either Δ*YTHDF3* clones (Supplementary Fig. 3A and 3B). Given that one of the hallmarks of cancer is resistance to stressful microenvironment conditions, namely, hypoxia, we assessed cellular viability under hypoxic conditions, which was simulated by the addition of CoCl_2_ to the culture medium. Under these conditions, both Δ*YTHDF3* clones exhibited a significant decrease in viability (Fig. 2D). This result was confirmed when the cells were grown in a hypoxia chamber (Supplementary Fig. 3C). We also assessed whether YTHDF3 confers resistance to 5-FU or cisplatin, but no significant differences were observed between the mock and Δ*YTHDF3* clones (Supplementary Fig. 3D and 3E).

### *YTHDF3* depletion impaired cell motility and the formation of lung metastases in vivo

Given that the observed morphological features might affect cellular motility, we performed wound healing assay and Transwell invasion assay to evaluate the ability of the cells to migrate. The results revealed a statistically significant decrease in the ability of the Δ*YTHDF3* cells to repair the wound compared with that of the mock cell lines, which was particularly evident for the SNU638 cells (Fig. 2E). Likewise, in the Transwell assays, we observed a significant decrease in the number of invasive cells for ΔYTHDF3 compared with mock cell lines (Supplementary Fig. 3F). Next, we assessed whether *YTHDF3* depletion had an impact on metastasis in vivo. For this purpose, we injected nude mice with SNU638 mock and Δ*YTHDF3* cells through the tail vein and assessed the capacity of the cells to form lung metastases. SNU638 mock cells formed metastases in 4/5 mice, whereas SNU638 Δ*YTHDF3* cells formed metastases in only 2/5 mice (Fig. F2). Δ*YTHDF3* cells formed less and smaller *foci* than mock cells (Supplementary Table 3). Thus, YTHDF3 has an impact on cell migration in vitro and metastasis formation in vivo.

### *YTHDF3* depletion led to significant alterations in migration-associated genes and signaling pathways

**Fig. 3:**
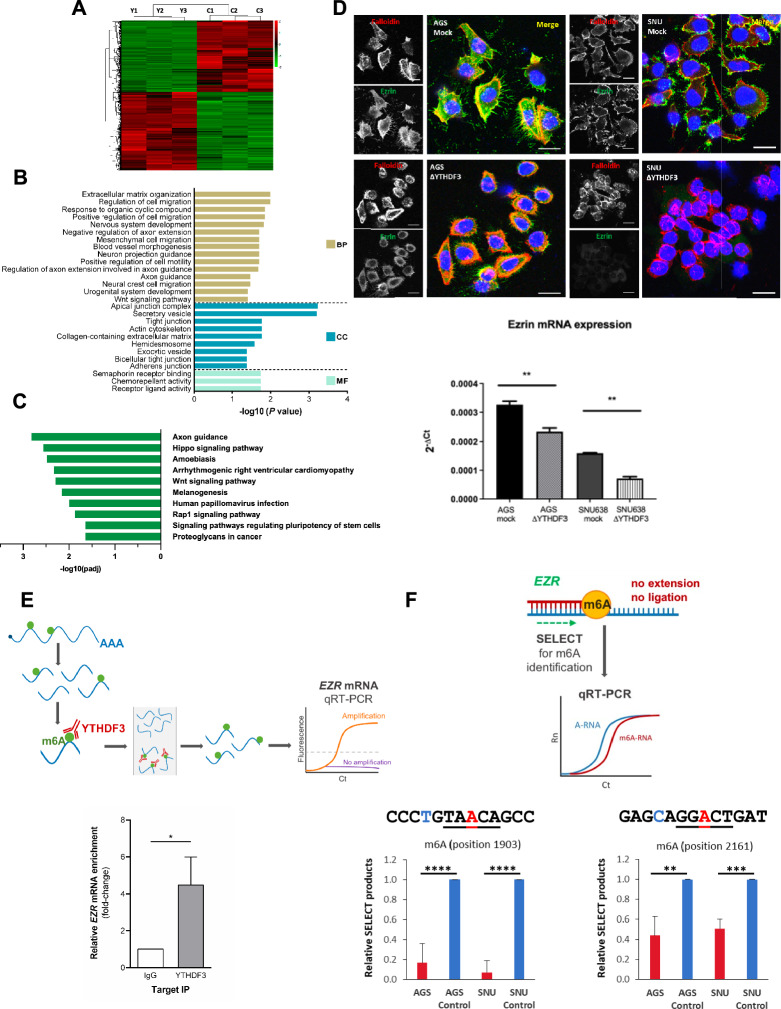
**A** Transcriptomic analysis in SNU638 ΔYTHDF3 (Y1–3) compared with mock SNU638 cell line (C1–3). Hierarchical clustering (*n *= 3 for each group) and heatmap showing DEGs in SNU638 ΔYTHDF3, the downregulated in green and the upregulated in red. **B** GO and **C** KEEG analysis of DEGs. The statistically significant terms in BP (biological processes), MF (molecular function), and CC (cellular component) are depicted according to their *p* value. **D** Immunofluorescence staining and qRT-PCR analysis for *EZR* in AGS and SNU638 mock and KO cell lines. Immunofluorescence was visualized by confocal microscopy. Nuclei are stained with DAPI. The scale bar corresponds to 20 μm and images were acquired with a 40x objective (**p<0.01). **E** Analysis of the physical interaction between the EZR transcript and YTHDF3 protein by RIP assay (*p = 0.042). Normal rabbit IgGs were used as non-specific IgG control. **F** Analysis of methylation in two predicted m^6^A sites in the EZR transcript. Relative SELECT products obtained after performing SELECT reaction with pairs of primers designed for each of the m^6^A sites (A indicated in red) compared with the same reaction using pairs of primers designed for a control non-methylated site (nucleotide indicated in blue). **p < 0.01; ***p < 0.001; ****p < 0.0001)

It is well known that m^6^A is involved in regulating gene expression either by stabilizing, leading to degradation, or increasing translation of its mRNA targets [23, 24]. Therefore, to identify the molecular targets of YTHDF3 that could be the molecular link between *YTHDF3* KO and alterations in the morphology, motility, and metastasis of GC cell lines, we performed RNA-seq in SNU638 Δ*YTHDF3* and its corresponding mock counterpart. A total of 1301 differentially expressed genes (DEGs) were identified in SNU638 Δ*YTHDF3* cells compared with mock control cells (*p* < 0.05, − 1.5 ≥ fold-change ≥ 1.5), 684 of which were downregulated and 617 of which were upregulated (Fig. 3A; Supplementary Table 3). We performed GO (Fig. 3B) and KEGG pathway (Fig. 3C) analyses to cluster DEGs into specific molecular processes and signaling pathways, respectively. Of note, only the downregulated genes grouped in a statistically significant manner. These analyses indicated that a broad spectrum of cellular mechanisms was affected by *YTHDF3* deletion, but notably, multiple entries were related to cell migration, motility, and extracellular matrix organization. Among the specific DEGs, *EZR* was significantly downregulated (approximately twofold) in the Δ*YTHDF3* cells compared with the corresponding mock SNU638 cells (Supplementary Table 4). EZR, a member of the ERM (ezrin–radixin–moesin) family, acts as a linker between the plasma membrane and the actin cytoskeleton, contributing to its reorganization, which is essential for cell motility and invasion and, hence, could be a possible target to explain the phenotype observed in Δ*YTHDF3* cells [25, 26]. Several studies have reported elevated EZR levels in GC tissues [27]. Therefore, to assess the putative relevance of EZR in GC cells, we studied its expression in both mock and *YTHDF3* KO cell lines. As expected from the RNA-seq data, we observed that the EZR transcript and protein levels were significantly decreased in both AGS and SNU638 Δ*YTHDF3* strains (Fig. 3D). Immunocytochemistry analysis using confocal microscopy revealed that the Δ*YTHDF3* cells presented a striking loss of EZR and phospho-EZR staining (Fig. 3D; Supplementary Fig. 4A). EZR expression was also decreased in the mouse metastasis obtained from SNU638 ΔYTHDF3 compared with SNU638 mock (Supplementary Fig. 4B). Moreover, using RNA immunoprecipitation (RIP), we demonstrated that the YTHDF3 protein binds the *EZR* transcript (Fig. 3E). Next, we used the SELECT method to precisely map m^6^A methylation at two predicted target sites in the *EZR* transcript. The sequence-based RNA adenosine methylation site predictor SRAMP, accessible at http://www.cuilab.cn/sramp (Supplementary Fig. 5), predicted three methylation sites with very high confidence, two of which are just 14 nucleotides apart and very close to the stop codon. We chose to validate the site closer to the stop codon and the other site located in the 3’UTR. The negative interference of m^6^A in the enzymatic activity of Bst DNA polymerase and SplintR ligase led to lower amplification at this site than at a non-methylated site just 4 nucleotides apart (Fig. 3F). These results demonstrate that both m^6^A putative sites are highly methylated in AGS and SNU638 cells.

### *YTHDF3* depletion led to mitotic spindle misorientation and increased sensitivity to paclitaxel

**Fig. 4:**
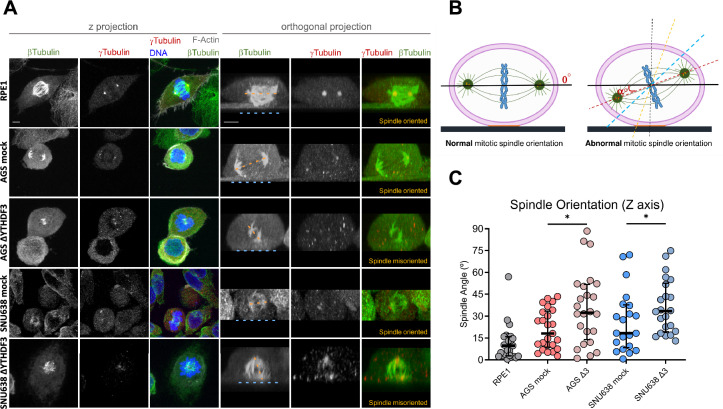
Loss of YTHDF3 leads to misorientation of the mitotic spindle. **A** Representative images of the mitotic spindle in AGS mock, AGS ΔYTHDF3, SNU638 mock and SNU638 ΔYTHDF3 cells; **B** schematic depiction of the mitotic spindle orientation; **C** measurement and quantification of the mitotic spindle angles. RPE1 is the control cell line. Data are presented as median with interquartile range. Symbol colors correspond to measurements from 3 independent experiments. Bigger angles are indicative of aberrant mitotic spindle orientation. Blue and yellow spotted lines represent the coverslip plane and the spindle orientation plane, respectively. *p < 0.05 (Mann–Whitney U test)

EZR is related to mitotic spindle orientation given its role in organizing the cell cortex and maintaining cell polarity [19, 28–30]. Disruptions in EZR function can lead to spindle misorientation, with significant implications for cancer progression and developmental biology [26, 31]. Therefore, to confirm that EZR downregulation contributes to the phenotype observed in *YTHDF3* KO cell models, we assessed whether these cells had misoriented mitotic spindles. In cultured cells with proper spindle orientation, the spindle is usually oriented parallel to the plane of the substrate to which the cells adhere. This results in low orthogonal spindle orientation angles (measured between the spindle and the substrate), similar to those observed for the control epithelial cell line RPE1 as well as the AGS and SNU638 mock cells (median angles of 9.8°, 18.2°, and 18.4°, respectively) (Fig. 4A and 4B). Importantly, the loss of *YTHDF3* led to a significant increase in the spindle angle in both the AGS and SNU63 *ΔYTHDF3* cell lines (median angles of 32.5° and 33.5°, respectively) (Fig. 4C). This result, in combination with the highly heterogeneous distribution of the spindle angles observed in both ΔYTHDF3 cell lines (Fig. 4C), suggests a significant disruption of spindle orientation when YTHDF3 is depleted.

**Fig. 5:**
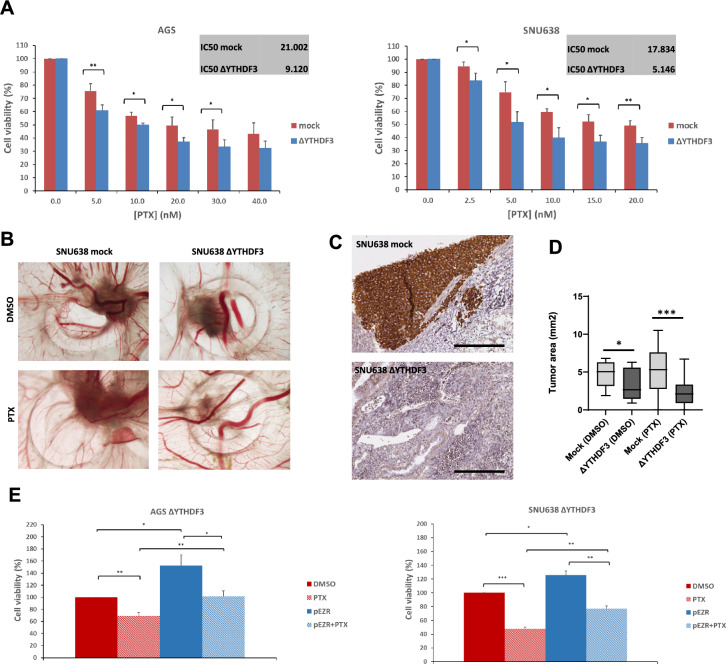
Loss of YTHDF3 leads to increased sensitivity to PTX in vitro and in vivo. **A** Cell viability was assessed in AGS mock, AGS ΔYTHDF3, SNU638 mock, and SNU638 ΔYTHDF3 cells after 48 h treatment with PTX (*p < 0.05; **p < 0.01). IC50 value for PTX was calculated for each condition. **B** Representative images of ex *ovo* SNU638 xenografts, treated with DMSO and PTX. Images with 20x amplification. **C** Immunohistochemistry staining confirming the presence and absence of YTHDF3 in the CAM xenografts formed from SNU638 mock and SNU638 ΔYTHDF3 cells, respectively. The scale bar corresponds to 200 μm. **D** Quantification of the xenograft area in SNU638 mock and SNU638 ΔYTHDF3 cells treated with DMSO and PTX (*p < 0.05; ***p < 0.001). **E** Cell viability was assessed in ΔYTHDF3 cells after EZR overexpression or control vector, and 48 h treatment with DMSO or PTX (*p < 0.05; **p < 0.01; ***p < 0.001)

Taxanes, such as paclitaxel (PTX), exert their anticancer effects through interference with the mitotic spindle, leading to mitotic arrest and subsequent cell death [32, 33]. We, therefore, compared the effect of PTX in *YTHDF3* KO cells (having spindle misorientation) with that in mock-treated cells. We observed significantly increased sensitivity to PTX in both the ΔYTHDF3 cell lines (Fig. 5A). The IC_50_ value for KO cells decreased from 21.0 μM PTX to 9.1 μM (2.3x) in AGS cells and from 17.8 μM to 5.1 μM (3.5x) in SNU638 cells. To assess the response to PTX in vivo, we inoculated SNU638 mock and ΔYTHDF3 cells in the chick chorioallantoic membrane (Fig. 5B and C). The obtained xenografts were smaller with SNU638 ΔYTHDF3 cells which was further amplified by PTX, suggesting increased response (Fig. 5D). Moreover, to study if EZR plays a role in the response to PTX observed in the KO cell lines, we overexpressed it in these cells (Supplementary Fig. 3G). The results show that overexpressing EZR restores increased resistance to PTX in both cell lines (Fig. 5E).

### Pharmacological inhibition of EZR activity mimics the phenotype of *YTHDF3* KO cells

**Fig. 6:**
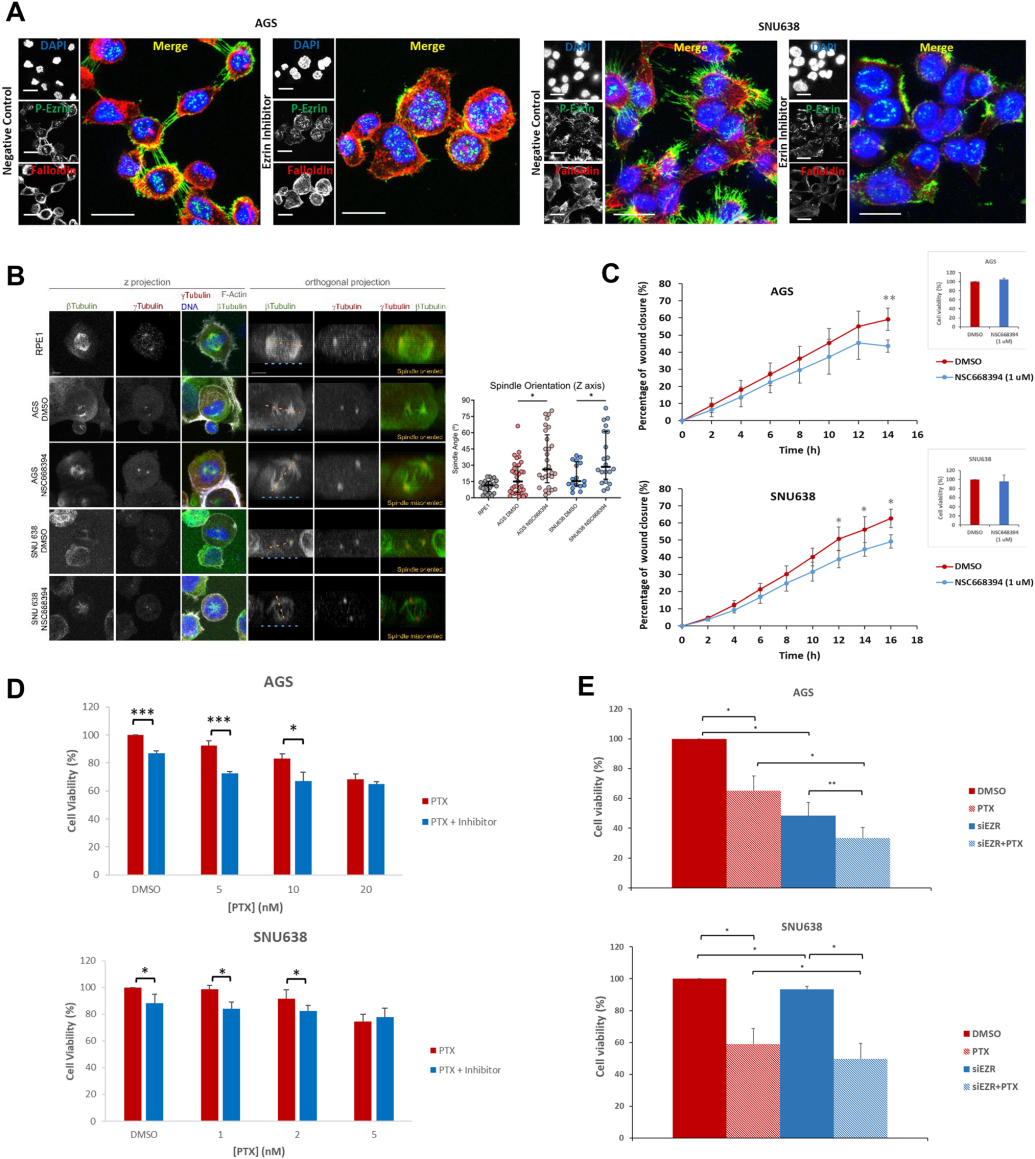
Pharmacological inhibition of EZR activity led to mitotic spindle misorientation and impaired migration. **A** Validation inhibition of EZR phosphorylation with NSC668394 (1 μM) compound, compared with DMSO-treated cells (negative control). Cells were visualized by confocal microscopy. Nuclei are stained with DAPI. The scale bar corresponds to 20 μm and images were acquired with a 40x objective. **B** Representative images of the mitotic spindle in AGS and SNU638 cells treated with NSC668394 (1 μM), compared with DMSO-treated cells, and measurement and quantification of the mitotic spindle angles. RPE1 is the control cell line. Data are presented as median with interquartile range. Bigger angles are indicative of aberrant mitotic spindle orientation. *p < 0.05 (Mann–Whitney U test). **C** Wound healing assay in AGS and SNU638 cells treated with NSC668394 (1 μM), compared with DMSO-treated cells, with left graphs indicating average and standard deviation (SD) of % of wound closure every 2 h (*p < 0.05, **p < 0.01). Cell viability was measured in the same cells to show that NSC668394 did not affect cell viability. **D** Cell viability was assessed in AGS and SNU638 cells treated with NSC668394 (2 μM) for 48 h followed by DMSO or PTX for 24 h (*p < 0.05; ***p < 0.001). **E** Cell viability was assessed in AGS and SNU638 cells with EZR downregulation using siRNAs and treated with DMSO or 20 nM PTX for 48h (*p < 0.05; **p < 0.01)

We next pharmacologically inhibited EZR activity in AGS and SNU638 cells and investigated the migration capacity, mitotic spindle orientation, and response to PTX of these cells. As expected, cells treated with NSC668394, a pharmacological inhibitor of EZR activation, presented a significant reduction in EZR phosphorylation, as determined using immunofluorescence (Fig. 6A). Most strikingly, significant misorientation of the mitotic spindle was noted during cell division (Fig. 6B) and decreased migration (Fig. 6C). EZR inhibition combined with different PTX concentrations led to significantly decreased cell viability in both cell lines (Fig. 6D) which was also observed when inhibiting EZR with siRNAs (Fig. 6E and Supplementary Fig. 3H).

## Discussion

A growing body of evidence has confirmed the impact of m^6^A modifications on the fine-tuning and coordination of gene expression [23, 34]. In this study, we showed that YTHDF3 is required for cell morphology and migration in GC cell lines, controlling the organization of the actin cytoskeleton and the orientation of the mitotic spindle. This impacted the response to paclitaxel which was mediated by the actin-binding protein EZR. These results reveal the role of YTHDF3 in enhancing GC cell aggressiveness, which is further reinforced by the observations in patient clinical samples. In our study, YTHDF3 was a biomarker of response to treatment, which is important in the clinical management of GC patients. GC with high YTHDF3 expression benefit more from treatment with chemotherapy but we could not establish a direct link between the improved patient outcome and the response to each of the drugs tested in vitro. This result, combined with the clear association of YTHDF3 with more aggressive features observed in cell lines, suggests that tumors with high YTHDF3 expression might be more aggressive, thus the impact of chemotherapy in these patients is more noticeable. We observed an association of YTHDF3 with GC of the intestinal type, but to explain these findings, further studies are required to uncover the molecular mechanisms that regulate YTHDF3 expression in GC. Notably, it has been shown, in clinical samples, cell lines, and a mouse model, that *Helicobacter pylori* infection leads to higher m^6^A modification levels [35].

Our results suggest that m^6^A modifications and its molecular machinery improve the ability of cancer cells to migrate by regulating the expression of multiple transcripts that collectively contribute to this phenotype [36–38]. They also suggest that YTHDF3 contributes to the improved capacity of cancer cells to survive, for example, to hypoxia. Thus, it is tempting to speculate that this epitranscriptomic mechanism could lead to a more plastic cellular phenotype, which is increasingly accepted as a cancer cell feature [39]. Consistent with our results, METTL3 expression has also been associated with a more aggressive phenotype [9–11, 36, 40] and YTHDF1 also affects cell migration [41]. In another study in GC, YTHDF3 was found to be associated with poor prognosis and tumor immune infiltration [42]. Importantly, we showed for the first time that YTHDF3 is involved in the response to paclitaxel, used as a third line therapy in GC [43], leading to increased resistance. We identified EZR, a member of the ezrin/radixin/moesin (ERM) protein family, as a potential culprit. EZR is strongly associated with cell migration which is consistent with the phenotype observed [44, 45] and there is one study reporting that EZR expression increases resistance to paclitaxel in breast cancer [46]. We confirmed that YTHDF3 binds to *EZR* mRNA, and we identified the methylated sites within its transcript, using the SELECT approach, which reinforces the link between *EZR* and YTHDF3, but we cannot exclude the role played by other targets. To identify the molecular pathways regulated by YTHDF3, we studied the overall transcriptomic changes induced by the *YTHDF3* KO instead of identifying the direct targets of m^6^A or YTHDF3, which represents a limitation of this study. The advantage of our approach is that it provides a broader picture of the cellular mechanisms affected by YTHDF3. This study focused on YTHDF3 since the analysis of the expression levels of the three paralogues YTHDF1/2/3 in GC and GC cell lines revealed that YTHDF3 was the most abundant reader but other readers might play redundant roles in GC [41, 47–51]. Future studies with concomitant KO of two or more readers may clarify the full spectrum of functions and potential redundancy between these family members in GC.

## Conclusion

Overall, our study revealed a new vulnerability of GC and two potential new therapeutic targets, YTHDF3 and EZR. The study of RNA chemical modifications, as well as the molecular players involved, is a growing field both in basic sciences and biomedicine that might offer novel cancer treatment opportunities. Our observations revealed interesting new molecular complexities in GC, involving epitranscriptomic alterations that provide a novel perspective on cancer development. The finding that m^6^A readers, in this case YTHDF3, are important for aggressive GC behavior supports the targeting of RNA modifications as a new and promising cancer therapeutic avenue.

## Supplementary Information

Below is the link to the electronic supplementary material.

Supplementary file1 (DOCX 8311 kb)

## References

[1] Sung H, Ferlay J, Siegel RL, Laversanne M, Soerjomataram I, Jemal A, et al. Global cancer statistics 2020: GLOBOCAN estimates of incidence and mortality worldwide for 36 cancers in 185 countries. CA Cancer J Clin. 2021;71:209–49. 10.3322/caac.21660. 33538338 10.3322/caac.21660

[2] Sitarz R, Skierucha M, Mielko J, Offerhaus J, Maciejewski R, Polkowski W. Gastric cancer: epidemiology, prevention, classification, and treatment. Cancer Manag Res. 2018;10:239–48. 10.2147/CMAR.S149619. 29445300 10.2147/CMAR.S149619 PMC5808709

[3] Zaccara S, Ries RJ, Jaffrey SR. Reading, writing and erasing mRNA methylation. Nat Rev Mol Cell Biol. 2019;20:608–24. 10.1038/s41580-019-0168-5. 31520073 10.1038/s41580-019-0168-5

[4] Destefanis E, Avşar G, Groza P, Romitelli A, Torrini S, Pir P, et al. A mark of disease: how mRNA modifications shape genetic and acquired pathologies. RNA. 2021;27:367–89. 10.1261/rna.077271.120. 33376192 10.1261/rna.077271.120 PMC7962492

[5] Dominissini D, Moshitch-Moshkovitz S, Schwartz S, Salmon-Divon M, Ungar L, Osenberg S, et al. Topology of the human and mouse m6A RNA methylomes revealed by m6A-seq. Nature. 2012;485:201–6. 10.1038/nature11112. 22575960 10.1038/nature11112

[6] He PC, He C. m^6^A RNA methylation: from mechanisms to therapeutic potential. EMBO J. 2021. 10.15252/embj.2020105977. 33470439 10.15252/embj.2020105977 PMC7849164

[7] Lobo J, Costa AL, Cantante M, Guimarães R, Lopes P, Antunes L, et al. M^6^A RNA modification and its writer/reader VIRMA/YTHDF3 in testicular germ cell tumors: a role in seminoma phenotype maintenance. J Transl Med. 2019;17:79. 10.1186/s12967-019-1837-z. 30866959 10.1186/s12967-019-1837-z PMC6416960

[8] Guimarães-Teixeira C, Lobo J, Miranda-Gonçalves V, Barros-Silva D, Martins-Lima C, Monteiro-Reis S, et al. Downregulation of m^6^A writer complex member METTL14 in bladder urothelial carcinoma suppresses tumor aggressiveness. Mol Oncol. 2022;16:1841–56. 10.1002/1878-0261.13181. 35048498 10.1002/1878-0261.13181 PMC9067151

[9] Yue B, Song C, Yang L, Cui R, Cheng X, Zhang Z, et al. METTL3-mediated N6-methyladenosine modification is critical for epithelial-mesenchymal transition and metastasis of gastric cancer. Mol Cancer. 2019;18:142. 10.1186/s12943-019-1065-4. 31607270 10.1186/s12943-019-1065-4 PMC6790244

[10] Wang Q, Chen C, Ding Q, Zhao Y, Wang Z, Chen J, et al. METTL3-mediated m(6)A modification of HDGF mRNA promotes gastric cancer progression and has prognostic significance. Gut. 2020;69:1193. 10.1136/gutjnl-2019-319639. 31582403 10.1136/gutjnl-2019-319639

[11] Lin S, Liu J, Jiang W, Wang P, Sun C, Wang X, et al. METTL3 promotes the proliferation and mobility of gastric cancer cells. Open Med. 2019;14:25–31. 10.1515/med-2019-0005. 10.1515/med-2019-0005 PMC6419388 30886897

[12] Guan K, Liu X, Li J, Ding Y, Li J, Cui G, et al. Expression status and prognostic value of M6A associated genes in gastric cancer. J Cancer. 2020;11:3027–40. 10.7150/jca.40866. 32226518 10.7150/jca.40866 PMC7086255

[13] Lopes N, Bergsland C, Bruun J, Bjørnslett M, Vieira AF, Mesquita P, et al. A panel of intestinal differentiation markers (CDX2, GPA33, and LI-cadherin) identifies gastric cancer patients with favourable prognosis. Gastric Cancer. 2020;23:811–23. 10.1007/s10120-020-01064-6. 32215766 10.1007/s10120-020-01064-6

[14] Pádua D, Pinto DF, Figueira P, Pereira CF, Almeida R, Mesquita P. HMGA1 has predictive value in response to chemotherapy in gastric cancer. Curr Oncol. 2021;29(1):56–67. 10.3390/curroncol29010005. 35049679 10.3390/curroncol29010005 PMC8774981

[15] Livak KJ, Schmittgen TD. Analysis of relative gene expression data using real-time quantitative PCR and the 2(-Delta Delta C(T)) method. Methods. 2001;25:402–8. 10.1006/meth.2001.1262. 11846609 10.1006/meth.2001.1262

[16] Coelho R, Ricardo S, Amaral AL, Huang Y-L, Nunes M, Neves JP, et al. Regulation of invasion and peritoneal dissemination of ovarian cancer by mesothelin manipulation. Oncogenesis. 2020;9:61. 10.1038/s41389-020-00246-2. 32612258 10.1038/s41389-020-00246-2 PMC7329842

[17] Ran FA, Hsu PD, Wright J, Agarwala V, Scott DA, Zhang F. Genome engineering using the CRISPR-Cas9 system. Nat Protoc. 2013;8:2281–308. 10.1038/nprot.2013.143. 24157548 10.1038/nprot.2013.143 PMC3969860

[18] Kent WJ, Sugnet CW, Furey TS, Roskin KM, Pringle TH, Zahler AM, et al. The human genome browser at UCSC. Genome Res. 2002;12:996–1006. 10.1101/gr.229102. 12045153 10.1101/gr.229102 PMC186604

[19] Muñoz-Sánchez J, Chánez-Cárdenas ME. The use of cobalt chloride as a chemical hypoxia model. J Appl Toxicol. 2019;39(4):556–70. 10.1002/jat.3749. 30484873 10.1002/jat.3749

[20] Kuleshov MV, Jones MR, Rouillard AD, Fernandez NF, Duan Q, Wang Z, et al. Enrichr: a comprehensive gene set enrichment analysis web server 2016 update. Nucleic Acids Res. 2016;44:W90–7. 10.1093/nar/gkw377. 27141961 10.1093/nar/gkw377 PMC4987924

[21] Xiao Y, Wang Y, Tang Q, Wei L, Zhang X, Jia G. An elongation- and ligation-based qPCR amplification method for the radiolabeling-free detection of locus-specific n6-methyladenosine modification. Angewandte Chemie International Edition - Wiley Online Library. 2018;57(49):15995–6000. 10.1002/anie.201807942.20. 10.1002/anie.201807942 30345651

[22] Pinto AT, Pinto ML, Cardoso AP, Monteiro C, Teixeira-Pinto M, Maia AF, Castro P, Figueira R, Monteiro A, Marques M, Mareel M, Dos Santos SG, Seruca R, Barbosa MA, Rocha S, Oliveira MJ. Ionizing radiation modulates human macrophages towards a pro-inflammatory phenotype preserving their pro-invasive and pro-angiogenic capacities. Sci Rep. 2016;6:18765. 10.1038/srep18765. 26735768 10.1038/srep18765 PMC4702523

[23] Murakami S, Jaffrey SR. Hidden codes in mRNA: Control of gene expression by m6A. Mol Cell. 2022;82:2236–51. 10.1016/j.molcel.2022.05.029. 35714585 10.1016/j.molcel.2022.05.029 PMC9216239

[24] Deng X, Qing Y, Horne D, Huang H, Chen J. The roles and implications of RNA m6A modification in cancer. Nat Rev Clin Oncol. 2023;20(8):507–26. 10.1038/s41571-023-00774-x. 37221357 10.1038/s41571-023-00774-x PMC12466201

[25] Ng T, Parsons M, Hughes WE, Monypenny J, Zicha D, Vojnovic B. EZR is a downstream effector of trafficking PKC-integrin complexes involved in the control of cell motility. EMBO J. 2001;20(10):2723–3274. 10.1093/emboj/20.11.2723. 11387207 10.1093/emboj/20.11.2723 PMC125254

[26] Clucas J, Valderrama F. ERM proteins in cancer progression. J Cell Sci. 2014;127(2):267–75. 24421310 10.1242/jcs.133108

[27] Liang F, Wang Y, Shi L, Zhang J. Association of Ezrin expression with the progression and prognosis of gastrointestinal cancer: a meta-analysis. Oncotarget. 2017;8(54):93186–95. 10.18632/oncotarget.21473. 29190988 10.18632/oncotarget.21473 PMC5696254

[28] Mak H, Naba A, Varma S, Schick C, Day A, SenGupta SK, et al. Ezrin phosphorylation on tyrosine 477 regulates invasion and metastasis of breast cancer cells. BMC Cancer. 2012;12:82. 10.1186/1471-2407-12-82. 22397367 10.1186/1471-2407-12-82 PMC3372425

[29] di Pietro F, Echard A, Morin X. Regulation of mitotic spindle orientation: an integrated view. EMBO Rep. 2016;17(8):1106–30. 10.15252/embr.201642292. 27432284 10.15252/embr.201642292 PMC4967962

[30] Machicoane M, de Frutos CA, Fink J, Rocancourt M, Lombardi Y, Garel S, et al. SLK-dependent activation of ERMs controls LGN-NuMA localization and spindle orientation. J Cell Biol. 2014;205(6):791–9. 10.1083/jcb.201401049. 24958772 10.1083/jcb.201401049 PMC4068135

[31] Kschonsak YT, Hoffmann I. Activated ezrin controls MISP levels to ensure correct NuMA polarization and spindle orientation. J Cell Sci. 2018;131:jcs214544. 10.1242/jcs.214544. 29669740 10.1242/jcs.214544

[32] Weaver BA. How Taxol/paclitaxel kills cancer cells. Mol Biol Cell. 2014;25(18):2677–81. 10.1091/mbc.E14-04-0916. 25213191 10.1091/mbc.E14-04-0916 PMC4161504

[33] Škubník J, Pavlíčková V, Ruml T, Rimpelová S. Current perspectives on taxanes: focus on their bioactivity, delivery and combination therapy. Plants (Basel). 2021;10(3):569. 10.3390/plants10030569. 33802861 10.3390/plants10030569 PMC8002726

[34] Boulias K, Greer EL. Biological roles of adenine methylation in RNA. Nat Rev Genet. 2022;24(3):143–60. 10.1038/s41576-022-00534-0. 36261710 10.1038/s41576-022-00534-0 PMC9974562

[35] Li H, Lin J, Cheng S, Chi J, Luo J, Tang Y, Zhao W, Shu Y, Liu X, Xu C. Comprehensive analysis of differences in N6-methyladenosine RNA methylomes in Helicobacter pylori infection. Front Cell Dev Biol. 2023;7(11):1136096. 10.3389/fcell.2023.1136096. 10.3389/fcell.2023.1136096 PMC10289286 37363723

[36] Li G, Fu Q, Liu C, Peng Y, Gong J, Li S, et al. The regulatory role of N6-methyladenosine RNA modification in gastric cancer: molecular mechanisms and potential therapeutic targets. Front Oncol. 2022;12:1074307. 10.3389/fonc.2022.1074307. 36561529 10.3389/fonc.2022.1074307 PMC9763625

[37] Wu W, Zhang F, Zhao J, He P, Li Y. The N6-methyladenosine:mechanisms, diagnostic value, immunotherapy prospects and challenges in gastric cancer. Exp Cell Res. 2022;415: 113115. 10.1016/j.yexcr.2022.113115. 35341774 10.1016/j.yexcr.2022.113115

[38] Xu Y, Huang C. Progress and application of epitranscriptomic m^6^A modification in gastric cancer. RNA Biol. 2022;19:885–96. 10.1080/15476286.2022.2096793. 35796515 10.1080/15476286.2022.2096793 PMC9272831

[39] Hanahan D. Hallmarks of cancer: new dimensions. Cancer Discov. 2022;12:31–46. 10.1158/2159-8290.CD-21-1059. 35022204 10.1158/2159-8290.CD-21-1059

[40] Huo FC, Zhu ZM, Zhu WT, Du QY, Liang J, Mou J. METTL3-mediated m^6^A methylation of SPHK2 promotes gastric cancer progression by targeting KLF2. Oncogene. 2021;40:2968–81. 10.1038/s41388-021-01753-1. 33758320 10.1038/s41388-021-01753-1

[41] Pi J, Wang W, Ji M, Wang X, Wei X, Jin J, et al. YTHDF1 promotes gastric carcinogenesis by controlling translation of FZD7. Cancer Res. 2021;81:2651–65. 10.1158/0008-5472.CAN-20-0066. 32788173 10.1158/0008-5472.CAN-20-0066

[42] Yu Y, Meng LL, Chen XY, Fan HN, Chen M, Zhang J, Zhu JS. m^6^A reader YTHDF3 is associated with clinical prognosis, related RNA signatures and immunosuppression in gastric cancer. Cell Signal. 2023;108: 110699. 10.1016/j.cellsig.2023.110699. 37149073 10.1016/j.cellsig.2023.110699

[43] Lordick F, Carneiro F, Cascinu S, Fleitas T, Haustermans K, Piessen G, Vogel A, Smyth EC, ESMO Guidelines Committee. Gastric cancer: ESMO Clinical Practice Guideline for diagnosis, treatment and follow-up. Ann Oncol. 2022;33(10):1005–20. 10.1016/j.annonc.2022.07.004. 35914639 10.1016/j.annonc.2022.07.004

[44] Neisch AL, Fehon RG. Ezrin, Radixin and Moesin: key regulators of membrane-cortex interactions and signaling. Curr Opin Cell Biol. 2011;23:377–82. 10.1016/j.ceb.2011.04.011. 21592758 10.1016/j.ceb.2011.04.011 PMC3148288

[45] Barik GK, Sahay O, Paul D, Santra MK. Ezrin gone rogue in cancer progression and metastasis: an enticing therapeutic target. Biochim Biophys Acta Rev Cancer. 2022;1877: 188753. 10.1016/j.bbcan.2022.188753. 35752404 10.1016/j.bbcan.2022.188753

[46] Hoskin V, Ghaffari A, Laight BJ, SenGupta S, Madarnas Y, Nicol CJB, Elliott BE, Varma S, Greer PA. Targeting the Ezrin adaptor protein sensitizes metastatic breast cancer cells to chemotherapy and reduces neoadjuvant therapy-induced metastasis. Cancer Res Commun. 2022;2(6):456–70. 10.1158/2767-9764.CRC-21-0117. 36923551 10.1158/2767-9764.CRC-21-0117 PMC10010290

[47] Chen D, Cheung H, Lau HC, Yu J, Wong CC. N6-methyladenosine RNA-binding protein YTHDF1 in gastrointestinal cancers: function, molecular mechanism and clinical implication. Cancers. 2022;14:3489. 10.3390/cancers14143489. 35884552 10.3390/cancers14143489 PMC9320224

[48] Liu T, Yang S, Cheng YP, Kong XL, Du DD, Wang X, et al. The N6-methyladenosine (m^6^A) methylation gene YTHDF1 reveals a potential diagnostic role for gastric cancer. Cancer Manag Res. 2020;12:11953–64. 10.2147/CMAR.S279370. 33244271 10.2147/CMAR.S279370 PMC7685380

[49] Chen XY, Liang R, Yi YC, Fan HN, Chen M, Zhang J, et al. The m^6^A reader YTHDF1 facilitates the tumorigenesis and metastasis of gastric cancer via USP14 translation in an m^6^A-dependent manner. Front Cell Dev Biol. 2021;9: 647702. 10.3389/fcell.2021.647702. 33791305 10.3389/fcell.2021.647702 PMC8006284

[50] Shen X, Zhao K, Xu L, Cheng G, Zhu J, Gan L, et al. YTHDF2 inhibits gastric cancer cell growth by regulating FOXC2 signaling pathway. Front Genet. 2021;11: 592042. 10.3389/fgene.2020.592042. 33505426 10.3389/fgene.2020.592042 PMC7831514

[51] Zhang Y, Zhou X, Cheng X, Hong X, Jiang X, Jing G, et al. PRKAA1, stabilized by FTO in an m^6^A-YTHDF2-dependent manner, promotes cell proliferation and glycolysis of gastric cancer by regulating the redox balance. Neoplasma. 2022;69:1338–48. 10.4149/neo_2022_220714N714. 36305690 10.4149/neo_2022_220714N714

