# Supplementary material for: The RNA-binding protein YTHDF3 affects gastric cancer cell migration and response to paclitaxel by regulating EZRIN
Source: Gastric Cancer. 2025 May 14;28(5):760–75. doi: 10.1007/s10120-025-01620-y (PMC12378723; doi:10.1007/s10120-025-01620-y)
Supplement: Supplementary file 1 — Supplementary file1 (DOCX 8311 kb) [file 10120_2025_1620_MOESM1_ESM.docx]

**SUPPLEMENTARY DATA**

**The RNA-binding protein YTHDF3 affects gastric cancer cell migration and response to Paclitaxel by regulating EZRIN**

Patrícia Mesquita^1,2^, Alexandre Coelho^1,3^, Ana S. Ribeiro^1,2^, Luís F.C. Póvoas^1^, Catarina de Oliveira^1^, Nelson Leça^1,4^, Sara Silva^1^, Diana Ferreira^1^, Diana Pádua^1,3^, Ricardo Coelho^5^, Carmen Jerónimo^3,6^, Joana Paredes^1,2^, Carlos Conde^1,4^, Bruno Pereira^1,2^, Raquel Almeida^1,2,7^

^1^i3S - Institute for Research and Innovation in Health, University of Porto, 4200-135 Porto, Portugal.

^2^IPATIMUP - Institute of Molecular Pathology and Immunology of the University of Porto, 4200-465 Porto, Portugal.

^3^ICBAS – School of Medicine and Biomedical Sciences, University of Porto, 4050-313 Porto, Portugal.

^4^IBMC - Institute of Molecular and Cell Biology, University of Porto, 4200-135 Porto, Portugal.

^5^Ovarian Cancer Research, Department of Biomedicine, University Hospital Basel and University of Basel, 4031 Basel, Switzerland.

^6^Cancer Biology and Epigenetics Group, Research Center of IPO Porto (CI-IPOP)/RISE@CI-IPOP (Health Research Network), Portuguese Oncology Institute of Porto (IPO Porto)/Porto Comprehensive Cancer Center Raquel Seruca (Porto.CCC Raquel Seruca), 4200-072, Porto, Portugal.

^7^Biology Department, Faculty of Sciences of the University of Porto, 4169-007 Porto, Portugal.

Corresponding author: Raquel Almeida

[ralmeida@ipatimup.pt](mailto:ralmeida@ipatimup.pt)

Rua Alfredo Allen, 208

4200-135 Porto

Phone: +351 220 408 800

**Supplementary Table 1.** List of primers used in this study.

| **Real-time PCR** | **Primer Forward** (5’ 🡪 3’) | **Primer Reverse** (5’ 🡪 3’) |
| --- | --- | --- |
| **18S** | CGCCGCTAGAGGTGAAATTC | CATTCTTGGCAAATGCTTTCG |
| **YTHDF1** | CCTACAAGCACACAACCTCCA | GACTCTGCCGTTCCTTGCG |
| **YTHDF2** | CCAGCTACAAGCACACCACT | CCACGACCTTGACGTTCCTTT |
| **YTHDF3** | TGCTACTTTCAAGCATACCACCT | TTTCTCTCCCTACGCATGGC |
| **EZRIN** | aatgccgaaaccaatcaatg | ggaggccgatagtctttacc |
| **qPCR SELECT** | ATGCAGCGACTCAGCCTCTG | TAGCCAGTACCGTAGTGCGTG |
| **CRISPR** | **Primer Forward** (5’ 🡪 3’) | **Primer Reverse** (5’ 🡪 3’) |
| **Pioneer PCR** | CCCTACCCCAGGAGGACAAT | CCCATGTACCCCGTGACATC |
| **WT specific** | CCCTACCCCAGGAGGACAAT | CCCATGTACCCGTGACATC |
| **SELECT** | **Primer Up** (5’ 🡪 3’) | **Primer Down** (5’ 🡪 3’) |
| **EZRIN-1903** | TAGCCAGTACCGTAGTGCGTGCCTGGCCTGGCTG | TACAGGGCCTCGAACTCGTCcagaggctgagtcgctgcat |
| **EZRIN-1903-4** | TAGCCAGTACCGTAGTGCGTGCCTGGCCTGGCTGTTAC | GGGCCTCGAACTCGTCcagaggctgagtcgctgcat |
| **EZRIN-2161** | TAGCCAGTACCGTAGTGCGTGGTAATTCAATCAG | CCTGCTCCCAGCACAACACACAGAGGCTGAGTCGCTGCAT |
| **EZRIN-2161-4** | TAGCCAGTACCGTAGTGCGTGGTAATTCAATCAGTCCT | CTCCCAGCACAACACAcagaggctgagtcgctgcat |

**Supplementary Table 2.** Clinico-pathological data and association with YTHDF3 in all patients included in the study.

|  | **All cases** | | **YTHDF3 Low** | | **YTHDF3 High** | | ***p*** |
| --- | --- | --- | --- | --- | --- | --- | --- |
|  | **n** | **%** | **n** | **%** | **n** | **%** |  |
| **Patients** | **331** |  | **131** | 39.6 | **200** | 60.4 |  |
| **Age** |  |  |  |  |  |  |  |
| **Mean±SD** | **67.5+11.9** |  | **66.8+12.8** |  | **68.0+11.3** |  | 0.384 |
|  | **32-95** |  | **32-92** |  | **32-95** |  |  |
| **Sex** |  |  |  |  |  |  |  |
| Female | **142** | 42.9 | **56** | 39.4 | **86** | 60.6 | 1.000 |
| Male | **189** | 57.1 | **75** | 39.7 | **114** | 60.3 |  |
| **Laurén Classification** |  |  |  |  |  |  |  |
| Intestinal | **153** | 46.2 | **55** | 35.9 | **98** | 64.1 | 0.001* |
| Diffuse | **42** | 12.7 | **28** | 66.7 | **14** | 33.3 |  |
| Mixed | **85** | 25.7 | **29** | 34.1 | **56** | 65.9 |  |
| Unclassified | **51** | 15.4 |  |  |  |  |  |
| **TNM** |  |  |  |  |  |  |  |
| I | **101** | 30.5 | **30** | 29.7 | **71** | 70.3 | 0.086 |
| II | **72** | 21.8 | **33** | 45.8 | **39** | 54.2 |  |
| III | **91** | 27.5 | **37** | 40.7 | **54** | 59.3 |  |
| IV | **67** | 20.2 | **31** | 46.3 | **36** | 53.7 |  |
| **Vascular Invasion** |  |  |  |  |  |  |  |
| No | **136** | 41.1 | **48** | 35.3 | **88** | 64.7 | 0.300 |
| Yes | **187** | 56.5 | **77** | 41.2 | **110** | 58.8 |  |
| ND | **8** | 2.4 |  |  |  |  |  |

Notes: *p* values (statistical significance threshold < 0.05) were obtained using Student’s t test for the continuous variable, Fisher’s exact test (2-sided) and Chi-square (χ^2^) test for categorical variables; * comparisons with *p*<0.05. ND = not determined; SD = standard deviation.

**Supplementary Table 3.** Quantification of the number of tumor foci and area occupied by the tumor

in mice with lung metastasis.

| **Mouse** | **Lung area (mm^2^)** | **nº of tumor foci** | **Tumor area (mm^2^)** | **Metastatic burden**  **(% of area with tumor)** |
| --- | --- | --- | --- | --- |
| Mock-1 | 63.17 | 42 | 3.02 | 4.78 |
| Mock-2 | 55.20 | 16 | 5.08 | 9.19 |
| Mock-3 | 54.52 | 3 | 0.71 | 1.31 |
| Mock-4 | 46.41 | 2 | 0.30 | 0.65 |
| ΔYTHDF3-1 | 44.67 | 2 | 0.02 | 0.05 |
| ΔYTHDF3-2 | 50.06 | 5 | 0.30 | 0.61 |

**Supplementary Table 4.** Gene expression signature of YTHDF3 KO SNU638 cells.

| **Gene ID** | **Gene Symbol** | **SNU638_Y3_1** | **SNU638_Y3_2** | **SNU638_Y3_3** | **SNU638_C_1** | **SNU638_C_2** | **SNU638_C_3** | **YTHDF3 KO** | **Mock** | **log2 (Fold-Change)** | **Adjusted *P* value** |
| --- | --- | --- | --- | --- | --- | --- | --- | --- | --- | --- | --- |
| ENSG00000100453 | **GZMB** | 14137.3300570701 | 12107.1197151687 | 13530.9589805202 | 785.204542274874 | 666.070168367031 | 685.2809824393 | 13258.469584253 | 712.185231027069 | 4.218 | 0.00000E+00 |
| ENSG00000123094 | **RASSF8** | 8352.76153935944 | 8473.40220431303 | 7971.88035794372 | 675.575745799742 | 630.651370639981 | 631.553331449237 | 8266.01470053873 | 645.926815962987 | 3.677 | 0.00000E+00 |
| ENSG00000111252 | **SH2B3** | 11993.4205163067 | 12362.1521193582 | 11206.2157026481 | 1844.01257660734 | 1668.61891514104 | 1770.98502414417 | 11853.9294461043 | 1761.20550529752 | 2.751 | 0.00000E+00 |
| ENSG00000145536 | **ADAMTS16** | 5156.83343996683 | 5089.77460919363 | 5866.76523288308 | 838.613443121733 | 790.035960411708 | 859.642415841016 | 5371.12442734785 | 829.430606458152 | 2.695 | 4.11902E-295 |
| ENSG00000161681 | **SHANK1** | 2928.06720302637 | 2679.81723937151 | 2410.72977166147 | 290.469460746075 | 243.996162119681 | 224.034167335925 | 2672.87140468645 | 252.83326340056 | 3.400 | 3.08040E-243 |
| ENSG00000197467 | **COL13A1** | 1349.52818016579 | 1478.79254522309 | 1319.84088057444 | 151.793718196336 | 137.739768938529 | 178.416350457569 | 1382.7205353211 | 155.983279197478 | 3.150 | 2.47554E-170 |
| ENSG00000133657 | **ATP13A3** | 12314.4446440128 | 12018.1549230095 | 12664.878151509 | 4531.32358763877 | 4687.08756587967 | 4344.8436253474 | 12332.4925728438 | 4521.08492628861 | 1.448 | 3.39159E-167 |
| ENSG00000131459 | **GFPT2** | 14203.7840962449 | 14645.5817847762 | 13538.2108534904 | 4947.35081528799 | 5289.20712723953 | 5103.11311479207 | 14129.1922448372 | 5113.22368577319 | 1.466 | 3.47316E-166 |
| ENSG00000134531 | **EMP1** | 12803.1374244062 | 10892.2560533511 | 12437.9981257274 | 3216.71502819766 | 2661.32910699087 | 2933.73248991026 | 12044.4638678282 | 2937.25887503293 | 2.036 | 6.69628E-163 |
| ENSG00000156299 | **TIAM1** | 4139.5754556752 | 4833.75370731343 | 3954.34273245889 | 893.896340489534 | 920.888740903311 | 995.48213721212 | 4309.22396514917 | 936.755739534988 | 2.202 | 1.17669E-159 |
| ENSG00000069020 | **MAST4** | 2009.97909258025 | 1984.90336283954 | 2011.87675830107 | 432.893196337699 | 369.929665149193 | 338.585574163796 | 2002.25307124029 | 380.469478550229 | 2.394 | 3.53294E-152 |
| ENSG00000118503 | **TNFAIP3** | 2453.68760030143 | 2659.05878786771 | 2568.19901329987 | 642.78080668325 | 643.441492041416 | 690.349628759118 | 2560.315133823 | 658.857309161261 | 1.959 | 2.37953E-151 |
| ENSG00000106366 | **SERPINE1** | 6116.83871358477 | 4824.85722809752 | 7526.40816120353 | 132.116754726441 | 125.933503029513 | 142.935826218848 | 6156.03470096194 | 133.6620279916 | 5.526 | 2.90384E-148 |
| ENSG00000112541 | **PDE10A** | 8719.7923095712 | 9469.80787649543 | 9206.77072658166 | 3232.64399862567 | 3473.00988823578 | 3369.63607341455 | 9132.12363754943 | 3358.42998675867 | 1.443 | 3.49744E-144 |
| ENSG00000104419 | **NDRG1** | 25264.803324437 | 21154.8390777535 | 19827.6566823502 | 1111.27993691885 | 825.454758138758 | 846.463935409491 | 22082.4330281803 | 927.732876822368 | 4.573 | 1.59513E-143 |
| novel.1 | **-** | 2237.96756544159 | 2609.63390333486 | 1968.36552047993 | 5.62198956282726 | 4.91927746209034 | 10.1372926396346 | 2271.98899641879 | 6.8928532215174 | 8.375 | 3.05958E-139 |
| novel.522 | **-** | 1077.57780446571 | 1069.55450129103 | 1115.75245555626 | 15.9289704280106 | 6.88698844692647 | 12.1647511675615 | 1087.628253771 | 11.6602366808329 | 6.536 | 6.62655E-138 |
| ENSG00000136830 | **FAM129B** | 12194.8273734981 | 11521.9290822997 | 11611.2846071258 | 4595.97646761128 | 4067.25860565629 | 4474.60097113472 | 11776.0136876412 | 4379.27868146743 | 1.427 | 1.92803E-131 |
| novel.106 | **-** | 1077.57780446571 | 1126.88736734914 | 1063.95336291205 | 15.9289704280106 | 4.91927746209034 | 8.10983411170769 | 1089.47284490897 | 9.65269400060287 | 6.802 | 7.62516E-127 |
| ENSG00000108854 | **SMURF2** | 7992.8873579819 | 6805.80660017443 | 9043.08559382596 | 2095.12811041363 | 1992.30737214659 | 2058.88413510979 | 7947.25985066076 | 2048.77320589 | 1.956 | 1.20000E-122 |
| ENSG00000124942 | **AHNAK** | 97592.3571924889 | 108851.388699783 | 106335.249343737 | 44035.170249105 | 45866.3592010379 | 44369.9161544168 | 104259.66507867 | 44757.1485348532 | 1.220 | 8.37108E-121 |
| ENSG00000232044 | **LINC01105** | 2585.57330881763 | 3313.44425908274 | 2723.59629123249 | 67.4638747539271 | 93.4662717797164 | 108.46903124409 | 2874.20461971096 | 89.7997259259113 | 5.003 | 5.64373E-119 |
| ENSG00000230439 | **AL512488.1** | 723.837842088921 | 607.926079754143 | 640.23678508242 | 17.802966948953 | 20.6609653407794 | 14.1922096954885 | 657.333568975161 | 17.552047328407 | 5.224 | 2.28185E-110 |
| ENSG00000151322 | **NPAS3** | 1128.69629613866 | 1478.79254522309 | 1362.31613654269 | 221.131589471206 | 192.835676513941 | 218.965521016108 | 1323.26832596814 | 210.977595667085 | 2.649 | 8.70715E-110 |
| ENSG00000228536 | **AL513283.1** | 1007.03428595704 | 951.923276102829 | 1089.85290923415 | 189.273648615184 | 192.835676513941 | 194.636018680985 | 1016.27015709801 | 192.248447936703 | 2.402 | 2.50363E-103 |
| ENSG00000011422 | **PLAUR** | 4335.87046369931 | 3676.22291155392 | 3837.27678308298 | 1316.48255596205 | 1312.4632268857 | 1356.36975518311 | 3949.79005277874 | 1328.43851267695 | 1.572 | 8.78934E-103 |
| ENSG00000115825 | **PRKD3** | 7319.14563773247 | 7543.22587740466 | 7669.37365690154 | 3540.9164263207 | 3503.50940850074 | 3357.47132224699 | 7510.58172401289 | 3467.29905235614 | 1.115 | 2.24020E-96 |
| ENSG00000137936 | **BCAR3** | 3593.62996460813 | 3659.41845081274 | 3346.22138481588 | 1197.48377688221 | 928.759584842655 | 978.248739724741 | 3533.08993341225 | 1034.8307004832 | 1.771 | 9.69471E-94 |
| ENSG00000163565 | **IFI16** | 5337.79290048906 | 5606.75890140732 | 4544.85238860287 | 1677.22688624347 | 1442.33215188489 | 1346.23246254348 | 5163.13473016642 | 1488.59716689061 | 1.794 | 3.42283E-92 |
| ENSG00000230090 | **AC108025.1** | 605.242941407686 | 651.419978143057 | 455.832015269037 | 30.9209425955499 | 21.6448208331975 | 23.3157730711596 | 570.831644939926 | 25.293845499969 | 4.492 | 9.27377E-90 |
| ENSG00000085063 | **CD59** | 15106.5366591891 | 14785.9484568495 | 15166.7743262243 | 7689.94472368722 | 7529.44608347547 | 7086.98128436856 | 15019.753147421 | 7435.45736384375 | 1.014 | 4.04269E-88 |
| ENSG00000156273 | **BACH1** | 2469.02314780331 | 2537.47357191689 | 2905.92909734011 | 802.070510963356 | 790.035960411708 | 910.328879039189 | 2637.47527235344 | 834.145116804751 | 1.661 | 1.19166E-84 |
| ENSG00000136158 | **SPRY2** | 4442.19692637904 | 3630.75201778369 | 4447.47009443176 | 1499.19721675394 | 1515.13745832382 | 1496.26439361007 | 4173.47301286483 | 1503.53302289594 | 1.473 | 1.31909E-76 |
| ENSG00000175137 | **SH3BP5L** | 4020.98055499396 | 3219.53697847032 | 3666.33977735709 | 1274.31763424085 | 1322.30178180988 | 1310.75193830476 | 3635.61910360712 | 1302.45711811849 | 1.481 | 7.04084E-76 |
| ENSG00000148516 | **ZEB1** | 1187.99374647927 | 1210.909671055 | 1174.80342117065 | 383.232288532725 | 349.268699808414 | 371.024910610627 | 1191.23561290164 | 367.841966317255 | 1.695 | 1.36721E-74 |
| ENSG00000260032 | **NORAD** | 18080.6105047211 | 19058.2354758697 | 19560.3733643061 | 10105.526239182 | 10413.1265317528 | 10077.4826130608 | 18899.7397816323 | 10198.7117946652 | 0.890 | 6.29794E-70 |
| ENSG00000171161 | **ZNF672** | 3331.90328724265 | 3097.95176251949 | 3184.60821576595 | 1480.45725154451 | 1375.42997840046 | 1355.35602591915 | 3204.82108850936 | 1403.74775195471 | 1.191 | 7.17464E-70 |
| ENSG00000133816 | **MICAL2** | 8649.24879106253 | 8088.8766026474 | 9513.42135503537 | 3812.64592185735 | 3652.07158785587 | 4060.99943143763 | 8750.5155829151 | 3841.90564705028 | 1.188 | 1.11588E-68 |
| ENSG00000159167 | **STC1** | 55142.5393374408 | 46751.9867773202 | 43894.5511067025 | 8401.12640338487 | 6826.97326188897 | 6492.93593568597 | 48596.3590738211 | 7240.34520031994 | 2.747 | 1.22446E-68 |
| ENSG00000115738 | **ID2** | 2811.51704201205 | 2663.01277863034 | 3032.31888339198 | 1137.51588821205 | 1197.35213427279 | 1200.25544853274 | 2835.61623467812 | 1178.37449033919 | 1.267 | 3.84552E-68 |
| ENSG00000133083 | **DCLK1** | 3677.46429095176 | 3741.46375913729 | 3977.13433322235 | 1772.80070881153 | 1625.32927347465 | 1492.20947655422 | 3798.6874611038 | 1630.1131529468 | 1.220 | 3.88169E-63 |
| ENSG00000175166 | **PSMD2** | 17508.0833979841 | 15832.7675112555 | 16801.5536900755 | 9151.66201002231 | 8981.61679028454 | 9150.93406579817 | 16714.1348664384 | 9094.737622035 | 0.878 | 2.36849E-62 |
| novel.213 | **-** | 893.551234443103 | 898.544400807343 | 902.340193862116 | 282.036476401834 | 271.544115907387 | 263.5696086305 | 898.145276370854 | 272.38340031324 | 1.721 | 3.43858E-62 |
| ENSG00000108055 | **SMC3** | 11221.5312920452 | 10757.8203674217 | 11363.6849442865 | 6350.03721121339 | 6239.61153291538 | 6241.53107822303 | 11114.3455345845 | 6277.05994078393 | 0.824 | 1.31607E-61 |
| novel.199 | **-** | 1883.20523323135 | 1825.75523464374 | 1613.02374494066 | 649.339794506548 | 506.685578595305 | 548.427531804233 | 1773.99473760525 | 568.150968302029 | 1.642 | 2.66048E-61 |
| ENSG00000138639 | **ARHGAP24** | 7206.68495605198 | 7525.43291897283 | 7276.73653465844 | 4108.73737216625 | 3801.61762270341 | 3888.66545656384 | 7336.28480322775 | 3933.0068171445 | 0.899 | 1.33892E-59 |
| ENSG00000243742 | **RPLP0P2** | 402.813714382818 | 319.284754082257 | 426.82452338828 | 39.3539269397908 | 23.6125318180336 | 26.35696086305 | 382.974330617785 | 29.7744732069581 | 3.679 | 2.21184E-59 |
| ENSG00000164684 | **ZNF704** | 301.599100870384 | 325.2157402262 | 303.542682895063 | 20.6139617303666 | 19.6771098483613 | 19.2608560153058 | 310.119174663882 | 19.8506425313446 | 3.965 | 2.70045E-58 |
| ENSG00000163814 | **CDCP1** | 12251.0577143383 | 11561.4689899259 | 11349.1811983461 | 6450.29602508381 | 5804.7474052666 | 5730.61152918545 | 11720.5693008701 | 5995.21831984528 | 0.967 | 7.14784E-58 |
| ENSG00000119953 | **SMNDC1** | 4520.91940355538 | 4174.42574764512 | 4390.49109252313 | 2291.89774511258 | 2202.85244752405 | 2166.33943708992 | 4361.94541457454 | 2220.36320990885 | 0.974 | 2.96014E-57 |
| ENSG00000237499 | **AL357060.1** | 397.701865215523 | 382.548606284314 | 373.989448891187 | 58.093892149215 | 56.0797630678298 | 54.7413802540269 | 384.746640130341 | 56.3050118236906 | 2.772 | 3.08581E-55 |
| novel.42 | **-** | 449.842726721928 | 515.007296832372 | 395.745067801755 | 70.2748695353407 | 69.8537399616828 | 75.0159655332962 | 453.531697118685 | 71.7148583434399 | 2.662 | 3.35356E-54 |
| ENSG00000160691 | **SHC1** | 9811.68329170533 | 9299.7862737024 | 10261.4002528177 | 5348.38607076966 | 5528.28401189712 | 5373.77882827031 | 9790.95660607516 | 5416.8163036457 | 0.854 | 4.17242E-54 |
| ENSG00000196954 | **CASP4** | 2219.56490843933 | 2084.74162959591 | 2007.73283088953 | 958.549220462048 | 858.905844880973 | 929.589735054495 | 2104.01312297493 | 915.681600132505 | 1.200 | 1.37059E-53 |
| ENSG00000118276 | **B4GALT6** | 5216.13089030745 | 5087.79761381232 | 4592.50755383554 | 2463.36842677881 | 2228.43269032692 | 2073.07634480528 | 4965.4786859851 | 2254.95915397034 | 1.139 | 8.45185E-53 |
| ENSG00000135052 | **GOLM1** | 5113.89390696156 | 5286.4856496344 | 5138.4699903055 | 2908.44260050263 | 2699.69947119518 | 2861.75771216885 | 5179.61651563382 | 2823.29992795555 | 0.875 | 2.95755E-52 |
| ENSG00000110841 | **PPFIBP1** | 3245.00185139864 | 3225.46796461426 | 3181.5002702073 | 1695.02985319242 | 1714.86012328469 | 1638.18649056495 | 3217.3233620734 | 1682.69215568069 | 0.935 | 3.15647E-52 |
| ENSG00000119681 | **LTBP2** | 684.987788417482 | 635.604015092543 | 693.071859579513 | 194.895638178012 | 156.433023294473 | 177.402621193606 | 671.221221029846 | 176.243760888697 | 1.928 | 4.82126E-52 |
| ENSG00000164543 | **STK17A** | 6558.50248163903 | 6278.93733105417 | 7084.04391002198 | 3584.95534456285 | 3608.78194618947 | 3538.92886049645 | 6640.49457423839 | 3577.55538374959 | 0.892 | 6.23571E-50 |
| novel.144 | **-** | 362.94129087792 | 338.066210204743 | 246.563680986433 | 27.1729495536651 | 13.7739768938529 | 21.2883145432327 | 315.857060689699 | 20.7450803302502 | 3.924 | 1.26900E-49 |
| ENSG00000246695 | **RASSF8-AS1** | 547.990230733986 | 492.271849947257 | 462.047906386342 | 118.061780819372 | 94.4501272721344 | 92.249363020675 | 500.769995689195 | 101.587090370727 | 2.299 | 1.92574E-49 |
| novel.208 | **-** | 560.258668735493 | 701.833360366572 | 578.077873909369 | 118.061780819372 | 149.546034847546 | 143.949555482812 | 613.389967670478 | 137.185790383243 | 2.163 | 2.34637E-49 |
| ENSG00000102804 | **TSC22D1** | 5823.41857138206 | 6049.60586682172 | 6114.3648957224 | 3510.93248198562 | 3346.09252971385 | 3316.92215168845 | 5995.79644464206 | 3391.31572112931 | 0.822 | 2.80470E-49 |
| ENSG00000196660 | **SLC30A10** | 371.120249545591 | 346.962689420657 | 394.70908594887 | 37.4799304188484 | 52.1443410981576 | 63.8649436296981 | 370.930674971706 | 51.163071715568 | 2.863 | 1.52129E-48 |
| ENSG00000258657 | **AL136018.1** | 251.502979030896 | 256.0209018802 | 248.635644692202 | 16.8659686884818 | 22.6286763256155 | 15.2059389594519 | 252.053175201099 | 18.2335279911831 | 3.789 | 1.42011E-46 |
| ENSG00000122133 | **PAEP** | 4557.7247175599 | 5140.18799141714 | 4249.59756053088 | 533.152010208118 | 402.396896398989 | 309.187425508856 | 4649.17008983598 | 414.912110705321 | 3.486 | 2.54388E-46 |
| novel.520 | **-** | 800.515579598341 | 733.4652864676 | 591.545637996864 | 180.840664270943 | 190.867965529105 | 163.210411498117 | 708.508834687602 | 178.306347099389 | 1.990 | 4.58926E-46 |
| ENSG00000147394 | **ZNF185** | 6845.78840484098 | 5790.61947186954 | 6785.68113639134 | 1193.73578384032 | 901.21163105495 | 805.914764850952 | 6474.02967103396 | 966.954059915408 | 2.743 | 1.61960E-45 |
| ENSG00000171617 | **ENC1** | 409.97030321703 | 451.743444630314 | 361.557666656577 | 70.2748695353407 | 80.6761503782815 | 78.0571533251866 | 407.757138167974 | 76.3360577462696 | 2.419 | 3.49804E-45 |
| ENSG00000133030 | **MPRIP** | 12125.3062248229 | 12269.2333364365 | 12299.1765574409 | 7592.49690459821 | 7771.47453461031 | 7819.90754221414 | 12231.2387062334 | 7727.95966047422 | 0.662 | 1.76785E-44 |
| ENSG00000123095 | **BHLHE41** | 292.397772369253 | 257.997897261514 | 330.478211070052 | 31.8579408560211 | 39.3542196967227 | 29.3981486549404 | 293.624626900273 | 33.5367697358947 | 3.129 | 2.91353E-44 |
| ENSG00000169908 | **TM4SF1** | 3455.61003709118 | 2462.34774742694 | 3650.80004956383 | 439.452184160997 | 439.783405110876 | 517.001924621366 | 3189.58594469398 | 465.41250463108 | 2.777 | 4.90227E-44 |
| ENSG00000196611 | **MMP1** | 4606.79846956593 | 2841.93086063929 | 4645.34262833264 | 288.595464225133 | 209.561219885048 | 181.45753824946 | 4031.35731951262 | 226.53807411988 | 4.153 | 1.19354E-43 |
| ENSG00000070087 | **PFN2** | 11892.2059027943 | 11601.0088975522 | 12069.1885861006 | 7368.55432034559 | 7395.64173650661 | 7573.57133107102 | 11854.134462149 | 7445.92246264108 | 0.671 | 1.99346E-43 |
| ENSG00000261253 | **AC137932.2** | 742.240499091182 | 734.453784158257 | 654.740531022798 | 226.753579034033 | 243.012306627263 | 213.89687469629 | 710.478271424079 | 227.887586785862 | 1.640 | 4.84022E-43 |
| ENSG00000113810 | **SMC4** | 18625.5336259548 | 20518.2465649703 | 20250.337278327 | 11661.8803498247 | 12057.1490595834 | 12017.7604242868 | 19798.0391564173 | 11912.2632778983 | 0.733 | 5.16433E-43 |
| ENSG00000106211 | **HSPB1** | 10550.8566812961 | 10324.8583789139 | 9924.70615063039 | 5889.97106532202 | 5924.7777753416 | 5150.75839019835 | 10266.8070702801 | 5655.16907695399 | 0.860 | 2.02646E-42 |
| ENSG00000129521 | **EGLN3** | 219.80951419367 | 229.331464232457 | 239.311808016244 | 5.62198956282726 | 11.8062659090168 | 5.06864631981731 | 229.484262147457 | 7.49896726388712 | 4.937 | 5.18819E-42 |
| ENSG00000187678 | **SPRY4** | 5765.1434908749 | 5679.90773051594 | 6079.14151272434 | 3316.03684380761 | 3504.49326399315 | 3427.41864146046 | 5841.39757803839 | 3415.98291642041 | 0.774 | 5.90145E-42 |
| ENSG00000107554 | **DNMBP** | 10002.8664505622 | 11843.1908317632 | 9785.88458234391 | 4964.21678397647 | 5610.92787326024 | 5052.42665159389 | 10543.9806215564 | 5209.19043627687 | 1.017 | 1.01142E-41 |
| ENSG00000119231 | **SENP5** | 3823.66317713639 | 3804.72761133934 | 3901.5076579618 | 2267.53579034033 | 2228.43269032692 | 2223.10827587187 | 3843.29948214585 | 2239.69225217971 | 0.779 | 1.97130E-41 |
| ENSG00000125148 | **MT2A** | 36741.927074847 | 35113.414967523 | 31536.3235836472 | 18457.9287330224 | 19212.73005594 | 16241.9702672226 | 34463.8885420057 | 17970.8763520617 | 0.939 | 7.08702E-41 |
| ENSG00000163960 | **UBXN7** | 5133.31893379728 | 5719.44763814223 | 5395.39348982078 | 3054.61432913614 | 3167.03083009376 | 3058.42118937776 | 5416.0533539201 | 3093.35544953589 | 0.808 | 1.49557E-40 |
| ENSG00000114850 | **SSR3** | 17998.8209180444 | 15633.0909777427 | 16873.0364379245 | 9565.81524115058 | 9941.85975088457 | 9890.9564284915 | 16834.9827779039 | 9799.54380684222 | 0.781 | 2.89678E-40 |
| ENSG00000229124 | **VIM-AS1** | 425.305850718914 | 528.846264501572 | 373.989448891187 | 84.3298434424089 | 95.4339827645525 | 93.2630922846385 | 442.713854703891 | 91.0089728305333 | 2.284 | 2.84434E-39 |
| ENSG00000163577 | **EIF5A2** | 2042.69492725094 | 1827.73223002506 | 2275.01614893365 | 949.179237857335 | 931.71115131991 | 907.287691247298 | 2048.48110206988 | 929.392693474848 | 1.140 | 1.22886E-38 |
| ENSG00000258077 | **AC078923.1** | 272.972745533534 | 264.917381096114 | 207.196370576835 | 24.3619547722515 | 31.4833757573781 | 17.2333974873789 | 248.362165735494 | 24.3595760056695 | 3.348 | 3.19213E-38 |
| ENSG00000185551 | **NR2F2** | 16378.364732012 | 16366.5562642103 | 15987.2719537086 | 9880.64665666891 | 10791.9108963338 | 10357.2718899147 | 16244.0643166436 | 10343.2764809725 | 0.651 | 1.38509E-36 |
| ENSG00000180884 | **ZNF792** | 857.768290272041 | 853.073507037114 | 800.813972279467 | 340.130368551049 | 350.252555300832 | 320.338447412454 | 837.218589862874 | 336.907123754778 | 1.313 | 1.59428E-36 |
| ENSG00000107249 | **GLIS3** | 2134.70821226224 | 2148.99397948863 | 1928.99821007033 | 1097.22496301179 | 1060.59622082668 | 1041.09995409048 | 2070.9001339404 | 1066.30704597631 | 0.958 | 1.91533E-36 |
| ENSG00000008952 | **SEC62** | 8187.1376263391 | 8570.27497799743 | 8301.32258716089 | 5413.97594900265 | 5345.28689030736 | 5353.50424299104 | 8352.91173049914 | 5370.92236076702 | 0.637 | 3.10990E-36 |
| ENSG00000240891 | **PLCXD2** | 864.924879106253 | 806.614115576229 | 970.714996152471 | 345.752358113876 | 339.430144884233 | 338.585574163796 | 880.751330278318 | 341.256025720635 | 1.367 | 3.43818E-36 |
| ENSG00000118855 | **MFSD1** | 2765.5103995064 | 2563.17451187397 | 2517.43590250854 | 1448.59931068849 | 1419.70347555927 | 1453.6877645236 | 2615.37360462964 | 1440.66351692379 | 0.860 | 6.26934E-36 |
| ENSG00000281106 | **TMEM272** | 254.570088531273 | 356.847666327229 | 209.268334282603 | 27.1729495536651 | 32.4672312497962 | 27.3706901270135 | 273.562029713702 | 29.0036236434916 | 3.239 | 1.07708E-35 |
| ENSG00000041353 | **RAB27B** | 1895.47367123285 | 2063.98317809211 | 1426.54701142151 | 672.764751018329 | 658.199324427687 | 527.139217261 | 1795.33462024883 | 619.367764235672 | 1.535 | 2.17411E-35 |
| ENSG00000127528 | **KLF2** | 775.978703595327 | 536.754246026829 | 728.295242577575 | 194.895638178012 | 196.771098483613 | 193.622289417021 | 680.342730733243 | 195.096342026215 | 1.802 | 7.12079E-35 |
| ENSG00000127603 | **MACF1** | 17126.739450104 | 20102.0890372037 | 16830.5611819563 | 10208.5960478338 | 10386.5624334575 | 9908.18982597888 | 18019.7965564213 | 10167.7827690901 | 0.826 | 7.12079E-35 |
| ENSG00000163655 | **GMPS** | 7319.14563773247 | 7164.63126188297 | 7654.86991096116 | 4685.92830061652 | 4740.21576247025 | 4685.45665803912 | 7379.54893685887 | 4703.86690704196 | 0.650 | 2.91564E-34 |
| ENSG00000126010 | **GRPR** | 329.203086373775 | 319.284754082257 | 472.407724915184 | 44.0389182421469 | 76.7407284086092 | 58.7962973098808 | 373.631855123739 | 59.858647986879 | 2.644 | 5.29135E-34 |
| ENSG00000161217 | **PCYT1A** | 4141.62019534212 | 4087.43795086729 | 4191.58257676937 | 2556.13125456546 | 2609.18476589271 | 2502.89755272579 | 4140.21357432626 | 2556.07119106132 | 0.696 | 6.79917E-34 |
| ENSG00000258088 | **AC078820.1** | 200.38448735795 | 229.331464232457 | 209.268334282603 | 29.0469460746075 | 21.6448208331975 | 26.35696086305 | 212.99476195767 | 25.6829092569517 | 3.050 | 1.03661E-33 |
| ENSG00000163975 | **MELTF** | 1794.25905772042 | 1746.67541939117 | 1824.36404292903 | 989.470163057597 | 882.518376699006 | 931.617193582421 | 1788.43284001354 | 934.535244446342 | 0.936 | 1.24613E-33 |
| ENSG00000198814 | **GK** | 1107.22652963602 | 1051.7615428592 | 1110.57254629183 | 521.908031082464 | 445.686538065384 | 410.560351905202 | 1089.85353959568 | 459.38497368435 | 1.245 | 1.79484E-33 |
| ENSG00000186642 | **PDE2A** | 153.355475018839 | 200.6650312034 | 190.620660930688 | 9.3699826047121 | 14.757832386271 | 9.12356337567116 | 181.547055717642 | 11.0837927888848 | 4.035 | 3.61351E-33 |
| ENSG00000257732 | **AC089983.1** | 280.129334367746 | 242.181934211 | 360.521684803693 | 20.6139617303666 | 39.3542196967227 | 48.6590046702462 | 294.27765112748 | 36.2090620324452 | 3.030 | 5.75894E-33 |
| ENSG00000186432 | **KPNA4** | 9353.66160631573 | 8272.73717310963 | 9462.65824424405 | 5529.22673504061 | 5343.31917932252 | 5272.40590187397 | 9029.68567455647 | 5381.65060541237 | 0.746 | 7.15085E-33 |
| ENSG00000189223 | **PAX8-AS1** | 5969.61745756668 | 7220.97563025043 | 6194.13549839448 | 3489.38152199479 | 3656.99086531796 | 3529.80529712077 | 6461.57619540386 | 3558.72589481117 | 0.861 | 7.91780E-33 |
| ENSG00000172216 | **CEBPB** | 2798.22623417709 | 2722.32264006977 | 2416.94566277878 | 1273.38063598037 | 1472.83167214985 | 1304.66956272098 | 2645.83151234188 | 1350.2939569504 | 0.971 | 1.29157E-32 |
| ENSG00000100557 | **CCDC198** | 570.482367070082 | 459.651426155572 | 428.896487094048 | 145.234730373038 | 94.4501272721344 | 111.510219035981 | 486.343426773234 | 117.065025560384 | 2.052 | 2.50750E-32 |
| ENSG00000135842 | **FAM129A** | 442.686137887716 | 415.169030076 | 514.882980883435 | 136.801746028797 | 116.094948105332 | 139.894638426958 | 457.57938294905 | 130.930444187029 | 1.805 | 8.51462E-32 |
| ENSG00000104783 | **KCNN4** | 272.972745533534 | 255.032404189543 | 162.649150902815 | 2.81099478141363 | 6.88698844692647 | 0 | 230.218100208631 | 3.23266107611337 | 6.145 | 2.43152E-31 |
| ENSG00000184432 | **COPB2** | 9716.60289719365 | 9822.70155206003 | 9795.20841901987 | 6353.78520425527 | 6721.70072420023 | 6434.13963837609 | 9778.17095609118 | 6503.2085222772 | 0.588 | 2.46109E-31 |
| ENSG00000183778 | **B3GALT5** | 2591.70752781838 | 3147.37664705234 | 2549.55133994795 | 1385.82042723692 | 1427.57431949862 | 1353.32856739122 | 2762.87850493956 | 1388.90777137559 | 0.992 | 2.46505E-31 |
| ENSG00000253276 | **CCDC71L** | 925.24469928033 | 769.051203331257 | 844.325210100602 | 362.618326802358 | 280.398815339149 | 274.720630534098 | 846.20703757073 | 305.912590891868 | 1.466 | 2.86182E-31 |
| ENSG00000172716 | **SLFN11** | 3849.22242297286 | 4300.95345204923 | 4006.1418251031 | 2199.13491732593 | 2477.34812990869 | 2269.73982201419 | 4052.10590004173 | 2315.40762308294 | 0.808 | 3.36217E-31 |
| ENSG00000152784 | **PRDM8** | 4090.50170366917 | 4223.85063217797 | 3737.8225252061 | 2400.58954332724 | 2251.06136665254 | 2091.32347155662 | 4017.39162035108 | 2247.6581271788 | 0.838 | 3.49807E-31 |
| novel.524 | **-** | 8473.4011797076 | 7358.37680925177 | 6297.7336836829 | 2023.91624261781 | 1580.07192082342 | 1570.2666298794 | 7376.50389088075 | 1724.75159777354 | 2.096 | 3.56988E-31 |
| ENSG00000033170 | **FUT8** | 5110.82679746118 | 5831.14787718649 | 5198.55693777279 | 3281.36790817018 | 3039.12961607941 | 3110.1213818399 | 5380.17720414015 | 3143.53963536316 | 0.775 | 4.88191E-31 |
| ENSG00000167034 | **NKX3-1** | 1191.06085597965 | 968.727736844 | 1121.96834667356 | 488.1760937055 | 482.089191284853 | 481.521400382644 | 1093.9189798324 | 483.928895124333 | 1.176 | 9.29134E-31 |
| ENSG00000114503 | **NCBP2** | 4465.7114325486 | 4300.95345204923 | 4538.63649748557 | 2761.33387360866 | 2649.52284108185 | 2847.56550247336 | 4435.10046069447 | 2752.80740572129 | 0.688 | 9.44590E-31 |
| ENSG00000169047 | **IRS1** | 5838.75411888394 | 6119.78920285837 | 6406.51177823573 | 3845.44086097384 | 3872.45521815751 | 3943.40683681787 | 6121.68503332602 | 3887.10097198307 | 0.655 | 3.94134E-30 |
| ENSG00000244405 | **ETV5** | 4298.04277986133 | 4575.75581005192 | 4009.24977066175 | 2610.47715367279 | 2530.47632649927 | 2553.58401592396 | 4294.349453525 | 2564.84583203201 | 0.744 | 5.56081E-30 |
| ENSG00000026025 | **VIM** | 434.507179220045 | 486.340863803314 | 364.665612215229 | 124.620768642671 | 122.981936552258 | 113.537677563908 | 428.504551746196 | 120.380127586279 | 1.832 | 7.09256E-30 |
| ENSG00000173559 | **NABP1** | 5094.46888012584 | 5333.93353878594 | 5764.20302944755 | 3294.48588381677 | 3372.65662800913 | 3217.57668382003 | 5397.53514945311 | 3294.90639854865 | 0.712 | 1.06014E-29 |
| ENSG00000162909 | **CAPN2** | 23514.5061695553 | 22789.8142581004 | 21457.256136937 | 15229.969725699 | 14826.7022707403 | 14080.6994764525 | 22587.1921881976 | 14712.4571576306 | 0.618 | 1.81170E-29 |
| ENSG00000205542 | **TMSB4X** | 1731.89449787942 | 1710.10100483686 | 1617.1676723522 | 959.486218722519 | 826.438613631176 | 822.134433074368 | 1686.38772502283 | 869.353088476021 | 0.955 | 4.22130E-29 |
| ENSG00000176438 | **SYNE3** | 206.518706358703 | 210.550008109971 | 179.224860548962 | 29.0469460746075 | 7.87084393934454 | 20.2745852792692 | 198.764525005879 | 19.0641250977404 | 3.375 | 5.30964E-29 |
| ENSG00000224128 | **LINC01248** | 150.288365518462 | 190.780054296829 | 131.56969531629 | 8.43298434424089 | 6.88698844692647 | 5.06864631981731 | 157.546038377194 | 6.79620637032822 | 4.528 | 7.21175E-29 |
| novel.65 | **-** | 276.039855033911 | 263.928883405457 | 237.239844310476 | 60.9048869306286 | 50.1766301133214 | 47.6452754062827 | 259.069527583281 | 52.9089308167443 | 2.289 | 8.17307E-29 |
| ENSG00000101680 | **LAMA1** | 703.390445419743 | 572.340162890486 | 490.019416414214 | 200.517627740839 | 141.675190908202 | 131.78480431525 | 588.583341574814 | 157.992540988097 | 1.895 | 1.70321E-28 |
| ENSG00000124762 | **CDKN1A** | 2275.79524927957 | 1827.73223002506 | 2136.19458064717 | 1065.36702215577 | 820.535480676668 | 784.626450307719 | 2079.90735331727 | 890.176317713384 | 1.223 | 2.02777E-28 |
| ENSG00000114120 | **SLC25A36** | 3572.1601981055 | 3915.43935269294 | 3590.71310209655 | 2169.15097299085 | 2324.85052858389 | 2193.71012721693 | 3692.77088429833 | 2229.23720959723 | 0.728 | 2.09032E-28 |
| ENSG00000215817 | **ZC3H11B** | 595.019243073096 | 693.925378841314 | 601.905456525705 | 233.312566857331 | 239.07688465759 | 259.514691574646 | 630.283359480039 | 243.968047696523 | 1.370 | 2.11964E-28 |
| ENSG00000134686 | **PHC2** | 12299.1090965109 | 10994.0713154887 | 10822.902417081 | 7280.4764838613 | 6761.05494389696 | 7216.73863015589 | 11372.0276096935 | 7086.09001930471 | 0.682 | 8.77374E-28 |
| ENSG00000031081 | **ARHGAP31** | 1326.01367399623 | 1099.20943201074 | 1315.6969531629 | 605.300876264401 | 450.605815527475 | 453.136980991667 | 1246.97335305662 | 503.014557594515 | 1.308 | 9.36625E-28 |
| ENSG00000224715 | **Z82186.1** | 149.265995685004 | 194.734045059457 | 122.245858640333 | 10.3069808651833 | 6.88698844692647 | 6.08237558378077 | 155.415299794931 | 7.75878163196352 | 4.317 | 1.15463E-27 |
| ENSG00000163918 | **RFC4** | 3510.81800809796 | 3531.90224871797 | 3336.89754813993 | 2113.86807562305 | 2155.62738388799 | 2231.21810998358 | 3459.87260165195 | 2166.90452316487 | 0.675 | 1.75539E-27 |
| ENSG00000172380 | **GNG12** | 12396.2342306895 | 12422.4504784883 | 11485.9308029268 | 8195.92378434167 | 7945.61695676831 | 7508.69265817736 | 12101.5385040349 | 7883.41113309578 | 0.618 | 2.66177E-27 |
| ENSG00000174013 | **FBXO45** | 2017.13568141447 | 2073.86815499869 | 2068.8557602097 | 1207.79075774739 | 1246.54490889369 | 1207.35155338048 | 2053.28653220762 | 1220.56240667385 | 0.750 | 3.12379E-27 |
| novel.176 | **-** | 2758.35381067219 | 3042.59589184269 | 2428.3414631605 | 1486.07924110734 | 1460.04155074841 | 1475.9898083308 | 2743.09705522513 | 1474.03686672885 | 0.896 | 3.12568E-27 |
| ENSG00000188596 | **CFAP54** | 121.662010181612 | 167.056109721057 | 154.361296079742 | 5.62198956282726 | 10.8224104165987 | 10.1372926396346 | 147.693138660804 | 8.86056420635354 | 4.067 | 4.50043E-27 |
| ENSG00000121879 | **PIK3CA** | 2838.09865768198 | 2820.18391144483 | 2688.37290823443 | 1700.65184275525 | 1704.03771286809 | 1752.73789739283 | 2782.21849245375 | 1719.14248433872 | 0.695 | 4.87459E-27 |
| ENSG00000121864 | **ZNF639** | 2775.73409784099 | 2621.49587562274 | 2799.22296649304 | 1700.65184275525 | 1648.94180529268 | 1683.80430744331 | 2732.15097998559 | 1677.79931849708 | 0.703 | 7.58694E-27 |
| ENSG00000058056 | **USP13** | 3267.49398773473 | 3209.65200156374 | 3085.15395788907 | 1999.55428784556 | 2045.43556873716 | 1934.19543564229 | 3187.43331572918 | 1993.061764075 | 0.677 | 8.26801E-27 |
| ENSG00000073792 | **IGF2BP2** | 3583.40626627354 | 3786.93465290752 | 4294.1447802049 | 2167.27697646991 | 2287.46401987201 | 2279.87711465383 | 3888.16189979532 | 2244.87270366525 | 0.792 | 1.61541E-26 |
| ENSG00000134853 | **PDGFRA** | 135.975187850037 | 143.332165145286 | 151.253350521089 | 6.55898782329847 | 6.88698844692647 | 15.2059389594519 | 143.520234505471 | 9.55063840989229 | 3.921 | 1.97475E-26 |
| ENSG00000185650 | **ZFP36L1** | 16838.4311570685 | 17240.3882227512 | 15486.8927187655 | 10343.5237973417 | 11039.8424804231 | 9595.96121267813 | 16521.9040328618 | 10326.4424968143 | 0.678 | 2.44492E-26 |
| ENSG00000018408 | **WWTR1** | 14336.6921745946 | 13108.4678758044 | 14868.4115525937 | 9174.14996827361 | 8782.87798081609 | 9370.91331607824 | 14104.5238676642 | 9109.31375505598 | 0.631 | 2.51576E-26 |
| ENSG00000161202 | **DVL3** | 4025.0700343278 | 4125.00086311226 | 3981.27826063388 | 2524.27331370944 | 2656.40982952878 | 2673.20406907165 | 4043.78305269131 | 2617.96240410329 | 0.627 | 3.11117E-26 |
| ENSG00000180530 | **NRIP1** | 654.316693413714 | 682.063406553429 | 689.96391402086 | 288.595464225133 | 311.882191096527 | 293.981486549404 | 675.448004662668 | 298.153047290355 | 1.180 | 3.36711E-26 |
| ENSG00000066697 | **MSANTD3** | 3738.8064809593 | 3641.62549238092 | 3447.74760639853 | 2342.49565117802 | 2220.56184638758 | 2134.91382990705 | 3609.39319324625 | 2232.65710915755 | 0.693 | 4.78399E-26 |
| ENSG00000152492 | **CCDC50** | 3416.75998341974 | 3487.4198526384 | 3090.33386715349 | 2085.75812780891 | 1976.5656842679 | 1928.1130600585 | 3331.50456773721 | 1996.81229071177 | 0.738 | 5.48833E-26 |
| ENSG00000080823 | **MOK** | 863.902509272794 | 702.821858057229 | 947.92339538902 | 325.13839638351 | 329.591589960052 | 358.860159443066 | 838.215920906348 | 337.863381928876 | 1.311 | 5.95516E-26 |
| novel.207 | **-** | 352.71759254333 | 391.445085500229 | 430.968450799817 | 116.18778429843 | 88.546994317626 | 130.771075051287 | 391.710376281125 | 111.835284555781 | 1.809 | 6.09874E-26 |
| ENSG00000141753 | **IGFBP4** | 3733.69463179201 | 2949.67710892092 | 3102.7656493881 | 1814.02863227226 | 1559.41095548264 | 1702.05143419465 | 3262.04579670034 | 1691.83034064985 | 0.947 | 6.84271E-26 |
| ENSG00000164949 | **GEM** | 2916.82113485832 | 3184.93955929732 | 2660.40139820656 | 839.550441382204 | 727.069208896952 | 546.400073276306 | 2920.72069745407 | 704.33990785182 | 2.052 | 1.15662E-25 |
| ENSG00000143322 | **ABL2** | 3539.44436343481 | 3556.6146909844 | 3236.40730841016 | 2184.14294515839 | 2153.65967290315 | 2096.39211787644 | 3444.15545427646 | 2144.73157864599 | 0.683 | 1.28845E-25 |
| ENSG00000140564 | **FURIN** | 1879.11575389751 | 1806.97377852126 | 1763.24111360886 | 1090.66597518849 | 977.952359463559 | 1046.16860041029 | 1816.44354867588 | 1038.26231168745 | 0.807 | 1.67139E-25 |
| ENSG00000151474 | **FRMD4A** | 136.997557683496 | 145.3091605266 | 120.173894934564 | 6.55898782329847 | 10.8224104165987 | 6.08237558378077 | 134.160204381554 | 7.82125794122599 | 4.101 | 1.69421E-25 |
| ENSG00000169715 | **MT1E** | 13728.3821236865 | 13759.8878539474 | 10757.6355603493 | 6932.85012922648 | 7321.85257457526 | 6220.2427636798 | 12748.6351793277 | 6824.98182249385 | 0.901 | 1.95620E-25 |
| ENSG00000227744 | **LINC01940** | 1673.61941737227 | 1996.76533512743 | 1758.06120434444 | 996.966149141367 | 978.936214955977 | 889.040564495956 | 1809.48198561471 | 954.980976197767 | 0.922 | 1.98663E-25 |
| ENSG00000114331 | **ACAP2** | 3391.20073758326 | 3732.56727992137 | 3550.30980983407 | 2269.40978686127 | 2262.86763256155 | 2227.16319292773 | 3558.02594244623 | 2253.14687078352 | 0.659 | 2.45434E-25 |
| ENSG00000136521 | **NDUFB5** | 3001.67783103541 | 3236.34143921149 | 3021.95906486314 | 1938.64940091493 | 1915.56664373798 | 1984.88189884046 | 3086.65944503668 | 1946.36598116446 | 0.665 | 7.96995E-25 |
| ENSG00000213186 | **TRIM59** | 2366.78616445742 | 2432.69281670723 | 2161.05814511639 | 1335.22252117147 | 1355.7528685521 | 1418.20724028488 | 2320.17904209368 | 1369.72754333615 | 0.761 | 8.84517E-25 |
| ENSG00000115648 | **MLPH** | 2170.4911564333 | 2450.48577513906 | 2366.18255198745 | 1225.59372469634 | 1348.86588010517 | 1406.04248911732 | 2329.05316118661 | 1326.83403130628 | 0.812 | 1.15143E-24 |
| ENSG00000125845 | **BMP2** | 404.858454049736 | 377.606117831029 | 439.25630562289 | 148.982723414922 | 132.820491476439 | 139.894638426958 | 407.240292501218 | 140.565951106106 | 1.534 | 1.31444E-24 |
| ENSG00000163904 | **SENP2** | 2765.5103995064 | 2629.403857148 | 2595.13454147486 | 1704.39983579713 | 1661.73192669412 | 1589.52748589471 | 2663.34959937642 | 1651.88641612865 | 0.689 | 1.54753E-24 |
| ENSG00000069849 | **ATP1B3** | 4920.66600843782 | 4450.21660333846 | 5322.87476011889 | 2901.88361267934 | 3049.95202649601 | 2960.08945077331 | 4897.91912396506 | 2970.64169664955 | 0.721 | 2.80850E-24 |
| ENSG00000139508 | **SLC46A3** | 515.2743960633 | 679.097913481457 | 561.502164263223 | 189.273648615184 | 222.351341286483 | 232.144001447633 | 585.291491269327 | 214.5896637831 | 1.449 | 3.35344E-24 |
| ENSG00000110880 | **CORO1C** | 13520.8410474943 | 11429.9987970685 | 13808.6021170932 | 8176.24682087178 | 7858.0538179431 | 7748.9464937367 | 12919.8139872187 | 7927.74904418386 | 0.705 | 6.05343E-24 |
| ENSG00000119138 | **KLF9** | 670.674610749057 | 694.913876531972 | 701.359714402586 | 288.595464225133 | 310.898335604109 | 338.585574163796 | 688.982733894538 | 312.693124664346 | 1.141 | 6.48296E-24 |
| ENSG00000142227 | **EMP3** | 2105.05948709193 | 2022.46627508451 | 1608.87981752912 | 1011.02112304844 | 869.728255297571 | 778.544074723939 | 1912.13519323519 | 886.431151023315 | 1.109 | 1.40033E-23 |
| ENSG00000155903 | **RASA2** | 1267.73859348907 | 1273.1850255664 | 1306.37311648694 | 750.535606637439 | 709.359810033426 | 691.363358023081 | 1282.43224518081 | 717.086258231315 | 0.838 | 1.58212E-23 |
| ENSG00000173905 | **GOLIM4** | 2966.91725669781 | 3164.18110779351 | 3048.89459303813 | 1855.256555733 | 2017.88761494946 | 1943.31899901796 | 3059.99765250982 | 1938.8210565668 | 0.659 | 1.77958E-23 |
| ENSG00000087074 | **PPP1R15A** | 6453.19838879276 | 5484.18518776583 | 6264.5822643906 | 3516.55447154845 | 3722.90918330997 | 3888.66545656384 | 6067.32194698306 | 3709.37637047409 | 0.710 | 1.98715E-23 |
| ENSG00000162407 | **PLPP3** | 229.0108426948 | 242.181934211 | 215.484225399908 | 56.2198956282726 | 58.047474052666 | 52.7139217261 | 228.892334101903 | 55.6604304690129 | 2.040 | 5.50405E-23 |
| ENSG00000133135 | **RNF128** | 1082.68965363301 | 908.429377713915 | 1293.94133425233 | 496.609078049741 | 481.105335792435 | 453.136980991667 | 1095.02012186642 | 476.950464944614 | 1.198 | 6.30565E-23 |
| ENSG00000145390 | **USP53** | 1805.50512588847 | 1782.26133625483 | 1884.45099039631 | 988.533164797126 | 1117.65983938692 | 1096.85506360847 | 1824.07248417987 | 1067.68268926417 | 0.773 | 7.94858E-23 |
| ENSG00000255282 | **WTAPP1** | 165.623913020346 | 103.792257519 | 154.361296079742 | 10.3069808651833 | 12.7901214014349 | 15.2059389594519 | 141.259155539696 | 12.76768040869 | 3.472 | 8.00362E-23 |
| ENSG00000143772 | **ITPKB** | 3353.37305374528 | 3485.44285725709 | 2678.01308970559 | 1650.99093495027 | 1785.69771873879 | 1709.1475390424 | 3172.27633356932 | 1715.27873091049 | 0.887 | 1.10546E-22 |
| ENSG00000178695 | **KCTD12** | 956.938164117557 | 904.475386951286 | 937.563576860178 | 478.806111100788 | 492.911601701452 | 500.78225639795 | 932.99237597634 | 490.83332306673 | 0.927 | 1.20818E-22 |
| ENSG00000130449 | **ZSWIM6** | 990.676368621701 | 945.992289958886 | 1093.99683664569 | 500.357071091626 | 536.201243367847 | 520.043112413256 | 1010.22183174209 | 518.867142290909 | 0.961 | 1.25172E-22 |
| ENSG00000043093 | **DCUN1D1** | 3303.2769319058 | 3440.96046117752 | 3297.53023773033 | 2146.66301473954 | 2275.65775396299 | 2151.13349813047 | 3347.25587693788 | 2191.15142227767 | 0.611 | 1.46823E-22 |
| ENSG00000118515 | **SGK1** | 391.56764621477 | 271.836864930714 | 261.067426926812 | 85.2668417028801 | 72.805306438937 | 68.9335899495154 | 308.157312690765 | 75.6685793637775 | 2.024 | 2.25925E-22 |
| ENSG00000231023 | **LINC00326** | 248.43586953052 | 233.285454995086 | 212.376279841256 | 58.093892149215 | 60.0151850375021 | 55.7551095179904 | 231.365868122287 | 57.9547289015692 | 1.997 | 2.71682E-22 |
| ENSG00000173889 | **PHC3** | 2578.41671998342 | 2831.05738604206 | 2691.48085379308 | 1677.22688624347 | 1757.16590945867 | 1574.32154693526 | 2700.31831993952 | 1669.5714475458 | 0.694 | 4.69973E-22 |
| ENSG00000162981 | **FAM84A** | 286.2635533685 | 274.802358002686 | 307.6866103066 | 93.699826047121 | 84.6115723479538 | 89.2081752287846 | 289.584173892595 | 89.1731912079531 | 1.699 | 6.77303E-22 |
| ENSG00000138678 | **GPAT3** | 2179.69248493444 | 1965.1334090264 | 2852.05804099013 | 1084.10698736519 | 1033.04826703897 | 1185.04950957329 | 2332.29464498366 | 1100.73492132582 | 1.083 | 8.04022E-22 |
| ENSG00000181788 | **SIAH2** | 1060.19751729691 | 1111.07140429863 | 1034.94587103129 | 605.300876264401 | 526.362688443666 | 579.8531389871 | 1068.73826420894 | 570.505567898389 | 0.905 | 1.02203E-21 |
| ENSG00000285108 | **AC103718.1** | 234.122691862095 | 208.573012728657 | 213.41226169414 | 57.1568938887438 | 54.1120520829937 | 51.7001924621366 | 218.702655428297 | 54.3230461446247 | 2.008 | 1.60553E-21 |
| ENSG00000230882 | **AC005077.4** | 229.0108426948 | 271.836864930714 | 135.713622727827 | 38.4169286793196 | 25.5802428028697 | 16.2196682234154 | 212.18711011778 | 26.7389465685349 | 2.982 | 1.74859E-21 |
| ENSG00000198843 | **SELENOT** | 5215.10852047399 | 4394.86073266166 | 5213.06068371317 | 3149.25115344374 | 2951.5664772542 | 2995.56997501203 | 4941.00997894961 | 3032.12920190332 | 0.704 | 1.76414E-21 |
| ENSG00000120742 | **SERP1** | 7241.44553038959 | 6708.93382649003 | 7170.03040381137 | 4444.18274941495 | 4979.29264712784 | 4622.60544367339 | 7040.136586897 | 4682.02694673872 | 0.588 | 3.14542E-21 |
| ENSG00000064393 | **HIPK2** | 3489.34824159532 | 3871.94545430403 | 4045.5091355127 | 2411.83352245289 | 2467.50957498451 | 2389.35987516188 | 3802.26761047068 | 2422.90099086643 | 0.650 | 6.04318E-21 |
| ENSG00000176771 | **NCKAP5** | 938.535507115296 | 1074.49698974431 | 1181.01931228796 | 515.349043259165 | 555.878353216208 | 542.345156220452 | 1064.68393638252 | 537.857517565275 | 0.985 | 6.75671E-21 |
| ENSG00000059804 | **SLC2A3** | 251.502979030896 | 296.549307197143 | 248.635644692202 | 81.5188486609952 | 77.7245839010273 | 74.0022362693327 | 265.56264364008 | 77.7485562771184 | 1.772 | 9.63736E-21 |
| ENSG00000125618 | **PAX8** | 747.352348258477 | 776.959184856515 | 706.539623667007 | 400.098257221207 | 320.73689052829 | 336.558115635869 | 743.617052260666 | 352.464421128455 | 1.076 | 2.71916E-20 |
| ENSG00000061938 | **TNK2** | 1727.80501854559 | 1906.81204527763 | 1702.1181842887 | 1088.79197866755 | 1064.53164279635 | 1096.85506360847 | 1778.91174937064 | 1083.39289502412 | 0.716 | 7.71005E-20 |
| ENSG00000133131 | **MORC4** | 3286.91901457045 | 3392.52407433532 | 3847.63660161182 | 2095.12811041363 | 2157.59509487282 | 2310.28899257273 | 3509.02656350586 | 2187.67073261973 | 0.682 | 1.73955E-19 |
| ENSG00000258245 | **RPL10P13** | 112.460681680482 | 101.815262137686 | 93.2383667595757 | 8.43298434424089 | 7.87084393934454 | 4.05491705585385 | 102.504770192581 | 6.78624844647976 | 3.908 | 2.00435E-19 |
| novel.206 | **-** | 310.800429371514 | 286.664330290572 | 270.39126360277 | 106.817801693718 | 85.5954278403718 | 91.2356337567116 | 289.285341088285 | 94.5496210969338 | 1.612 | 2.90076E-19 |
| ENSG00000175110 | **MRPS22** | 3015.99100870384 | 2603.70291719091 | 2961.87211739585 | 1815.9026287932 | 1803.40711760232 | 1819.64402881441 | 2860.5220144302 | 1812.98459173665 | 0.658 | 3.67490E-19 |
| ENSG00000114744 | **COMMD2** | 1845.37754939337 | 1853.43316998214 | 1814.00422440019 | 1202.16876818456 | 1151.11092612914 | 1182.0083217814 | 1837.60498125857 | 1178.42933869837 | 0.641 | 4.92053E-19 |
| ENSG00000101457 | **DNTTIP1** | 2614.19966415448 | 2021.47777739386 | 1850.26358925114 | 1198.42077514268 | 1008.45187972852 | 955.946695917545 | 2161.98034359982 | 1054.27311692958 | 1.036 | 7.69007E-19 |
| ENSG00000180573 | **HIST1H2AC** | 681.920678917105 | 763.120217187314 | 692.035877726628 | 340.130368551049 | 384.687497535464 | 345.68167901154 | 712.358924610349 | 356.833181699351 | 0.998 | 8.56626E-19 |
| ENSG00000073803 | **MAP3K13** | 1383.26638466993 | 1358.19582696291 | 1317.76891686867 | 789.88953357723 | 767.407284086092 | 862.683603632906 | 1353.07704283384 | 806.660140432076 | 0.747 | 1.34593E-18 |
| ENSG00000145107 | **TM4SF19** | 5296.89810715071 | 4998.83282165317 | 3667.37575920998 | 1755.93474012305 | 1636.15168389125 | 1529.71745932086 | 4654.36889600462 | 1640.60129444505 | 1.504 | 3.66003E-18 |
| ENSG00000136167 | **LCP1** | 29001.5650657294 | 26906.9071396874 | 23689.7970299024 | 18233.0491505093 | 16396.9356366395 | 15244.4606714825 | 26532.7564117731 | 16624.8151528771 | 0.674 | 5.06452E-18 |
| ENSG00000175054 | **ATR** | 2437.32968296608 | 2508.80713888783 | 2806.47483946323 | 1663.1719123364 | 1663.69963767895 | 1654.40615878837 | 2584.20388710571 | 1660.42590293457 | 0.638 | 6.12293E-18 |
| ENSG00000177311 | **ZBTB38** | 1159.36739114242 | 1341.39136622174 | 980.038832828429 | 624.040841473826 | 573.587752079733 | 500.78225639795 | 1160.26586339753 | 566.136949983836 | 1.035 | 1.23196E-17 |
| ENSG00000181744 | **C3orf58** | 1639.88121286812 | 1643.87165956283 | 1703.15416614158 | 1015.70611435079 | 1087.16031912196 | 1076.5804783292 | 1662.30234619084 | 1059.81563726732 | 0.650 | 1.53064E-17 |
| ENSG00000114126 | **TFDP2** | 2405.63621812886 | 2503.86465043454 | 2242.90071149424 | 1564.78709498692 | 1588.92662025518 | 1548.97831533617 | 2384.13386001921 | 1567.56401019276 | 0.605 | 2.16560E-17 |
| ENSG00000156463 | **SH3RF2** | 1451.76516351168 | 1367.09230617883 | 1643.0672186743 | 844.23543268456 | 918.921029918475 | 836.326642769856 | 1487.30822945494 | 866.49436845763 | 0.779 | 3.08783E-17 |
| ENSG00000163754 | **GYG1** | 1441.54146517709 | 1336.44887776846 | 1479.3820859186 | 829.243460517021 | 857.921989388554 | 908.301420511262 | 1419.12414295472 | 865.155623472279 | 0.714 | 3.66454E-17 |
| ENSG00000223784 | **LINP1** | 216.742404693293 | 246.135924973629 | 299.398755483526 | 55.2828973678014 | 71.8214509465189 | 87.1807167008577 | 254.092361716816 | 71.4283550050593 | 1.834 | 3.66857E-17 |
| ENSG00000170291 | **ELP5** | 1804.48275605501 | 1746.67541939117 | 1789.14065993097 | 1190.92478905891 | 1172.75574696234 | 1105.97862698414 | 1780.09961179238 | 1156.55305433513 | 0.622 | 7.22524E-17 |
| ENSG00000173546 | **CSPG4** | 1240.13460798568 | 1149.62281423426 | 891.980375333274 | 590.308904096862 | 533.249676890592 | 494.699880814169 | 1093.9125991844 | 539.419487267208 | 1.020 | 1.22960E-16 |
| ENSG00000115756 | **HPCAL1** | 1968.06192940844 | 1752.60640553511 | 2000.48095791934 | 1151.57086211912 | 1121.5952613566 | 1271.21649701018 | 1907.04976428763 | 1181.4608734953 | 0.691 | 2.36344E-16 |
| ENSG00000110852 | **CLEC2B** | 99.169873845516 | 140.366672073314 | 77.6986389663131 | 0 | 0.983855492418067 | 5.06864631981731 | 105.745061628381 | 2.01750060407846 | 5.753 | 2.47928E-16 |
| ENSG00000197903 | **HIST1H2BK** | 1543.77844852298 | 1189.16272186054 | 1423.43906586286 | 828.306462256549 | 711.327521018262 | 755.228301652779 | 1385.46007874879 | 764.954094975864 | 0.856 | 7.83909E-16 |
| ENSG00000168209 | **DDIT4** | 647.160104579502 | 776.959184856515 | 588.437692438211 | 321.390403341625 | 343.365566853905 | 315.269801092637 | 670.852327291409 | 326.675257096056 | 1.039 | 8.13787E-16 |
| ENSG00000114125 | **RNF7** | 2758.35381067219 | 2548.34704651411 | 2396.2260257211 | 1710.95882362043 | 1738.47265510272 | 1599.66477853434 | 2567.64229430247 | 1683.0320857525 | 0.609 | 1.06028E-15 |
| ENSG00000104998 | **IL27RA** | 2264.54918111153 | 2058.05219194817 | 2469.78073727587 | 1481.39424980498 | 1451.18685131665 | 1352.31483812726 | 2264.12737011186 | 1428.2986464163 | 0.664 | 1.31357E-15 |
| ENSG00000196428 | **TSC22D2** | 2007.93435291334 | 1963.15641364509 | 2108.22307061929 | 1291.18360292933 | 1385.26853332464 | 1370.5619648786 | 2026.43794572591 | 1349.00470037752 | 0.587 | 1.32040E-15 |
| ENSG00000082996 | **RNF13** | 1542.75607868952 | 1509.43597363346 | 1475.23815850706 | 961.360215243461 | 957.291394122779 | 1015.75672249139 | 1509.14340361001 | 978.13611061921 | 0.626 | 1.57041E-15 |
| ENSG00000185201 | **IFITM2** | 2505.82846180783 | 2085.73012728657 | 2359.96666087015 | 1555.41711238221 | 1409.86492063509 | 1336.09516990384 | 2317.17508332152 | 1433.79240097371 | 0.692 | 2.66866E-15 |
| ENSG00000087086 | **FTL** | 51063.2837019396 | 44401.3392689376 | 58490.4994319876 | 31590.8963517868 | 32239.9606310476 | 34902.698558262 | 51318.3741342883 | 32911.1851803655 | 0.641 | 3.61937E-15 |
| ENSG00000165376 | **CLDN2** | 109.393572180105 | 101.815262137686 | 162.649150902815 | 14.9919721675394 | 20.6609653407794 | 25.3432315990865 | 124.619328406869 | 20.3320563691351 | 2.620 | 7.09112E-15 |
| ENSG00000114127 | **XRN1** | 2009.97909258025 | 2497.9336642906 | 2106.15110691353 | 1236.837703822 | 1406.91335415784 | 1385.76790383805 | 2204.68795459479 | 1343.17298727263 | 0.715 | 1.14799E-14 |
| ENSG00000105810 | **CDK6** | 1482.43625851545 | 1526.24043437463 | 1679.32658352525 | 975.415189150529 | 1043.87067745557 | 972.16636414096 | 1562.66775880511 | 997.150743582353 | 0.648 | 1.30703E-14 |
| ENSG00000235897 | **TM4SF19-AS1** | 330.225456207234 | 327.192735607514 | 242.419753574897 | 93.699826047121 | 126.917358521931 | 94.276821548602 | 299.945981796548 | 104.964668705885 | 1.516 | 1.36582E-14 |
| ENSG00000229563 | **LINC01204** | 517.319135730218 | 627.696033567286 | 586.365728732443 | 274.540490318064 | 306.962913634437 | 212.883145432327 | 577.126966009982 | 264.795516461609 | 1.123 | 1.37507E-14 |
| ENSG00000085274 | **MYNN** | 1872.98153489676 | 1637.94067341889 | 1945.57391971648 | 1182.49180471467 | 1147.17550415947 | 1156.66509018231 | 1818.83204267737 | 1162.11079968548 | 0.646 | 1.91241E-14 |
| ENSG00000124145 | **SDC4** | 2260.45970177769 | 1793.13481085206 | 2255.33249372885 | 1347.4034985576 | 1254.41575283304 | 1237.76343129939 | 2102.9756687862 | 1279.86089423001 | 0.716 | 4.45501E-14 |
| ENSG00000138623 | **SEMA7A** | 894.573604276562 | 773.005194093886 | 984.182760239966 | 518.160038040579 | 441.751116095712 | 486.590046702462 | 883.920519536805 | 482.167066946251 | 0.874 | 6.14003E-14 |
| ENSG00000238266 | **LINC00707** | 127.796229182366 | 122.573713641486 | 190.620660930688 | 34.6689356374348 | 31.4833757573781 | 32.4393364468308 | 146.99686791818 | 32.8638826138812 | 2.160 | 6.34202E-14 |
| ENSG00000150593 | **PDCD4** | 2247.16889394272 | 2496.94516659994 | 2326.81524157786 | 1566.66109150786 | 1679.44132555764 | 1444.56420114793 | 2356.97643404017 | 1563.55553940448 | 0.592 | 9.37804E-14 |
| ENSG00000146592 | **CREB5** | 895.595974110021 | 1003.325156017 | 1114.71647370337 | 612.796862348171 | 575.555463064569 | 541.331426956489 | 1004.54586794346 | 576.561250789743 | 0.800 | 9.50593E-14 |
| ENSG00000163961 | **RNF168** | 2129.59636309495 | 2243.88975779172 | 1930.03419192322 | 1386.75742549739 | 1441.34829639247 | 1269.18903848225 | 2101.17343760329 | 1365.76492012404 | 0.621 | 9.92429E-14 |
| ENSG00000179981 | **TSHZ1** | 933.423657948002 | 1133.80685118374 | 961.391159476514 | 506.916058914924 | 620.8128157158 | 573.770763403319 | 1009.54055620275 | 567.166546011348 | 0.833 | 1.13840E-13 |
| ENSG00000185798 | **WDR53** | 758.598416426525 | 738.407774920886 | 793.562099309278 | 411.342236346861 | 458.476659466819 | 459.219356575448 | 763.522763552229 | 443.012750796376 | 0.786 | 1.24898E-13 |
| ENSG00000229771 | **AL035665.1** | 67.4764090082893 | 112.688736734914 | 58.0149837615138 | 0 | 2.9515664772542 | 4.05491705585385 | 79.3933765015724 | 2.33549451103602 | 5.117 | 1.34103E-13 |
| novel.165 | **-** | 466.200644057271 | 439.881472342429 | 420.608632270975 | 226.753579034033 | 227.270618748573 | 183.484996777387 | 442.230249556892 | 212.503064853331 | 1.056 | 1.36271E-13 |
| ENSG00000130513 | **GDF15** | 747.352348258477 | 659.327959668314 | 615.3732206132 | 374.799304188484 | 239.07688465759 | 323.379635204344 | 674.017842846663 | 312.418608016806 | 1.108 | 1.41349E-13 |
| ENSG00000167723 | **TRPV3** | 279.106964534287 | 225.377473469829 | 206.160388723951 | 95.5738225680634 | 57.0636185602479 | 46.6315461423192 | 236.881608909356 | 66.4229957568768 | 1.830 | 1.87998E-13 |
| ENSG00000115616 | **SLC9A2** | 129.840968849284 | 136.412681310686 | 78.7346208191972 | 20.6139617303666 | 22.6286763256155 | 14.1922096954885 | 114.996090326389 | 19.1449492504902 | 2.584 | 2.09333E-13 |
| ENSG00000186001 | **LRCH3** | 2784.93542634212 | 3062.36584565583 | 2541.26348512488 | 1765.30472272776 | 1953.93700794228 | 1839.91861409368 | 2796.18825237428 | 1853.05344825457 | 0.594 | 3.08718E-13 |
| ENSG00000175182 | **FAM131A** | 848.56696177091 | 836.269046295943 | 723.115333313154 | 455.381154589008 | 481.105335792435 | 457.191898047521 | 802.650447126669 | 464.559462809655 | 0.789 | 3.16985E-13 |
| novel.86 | **-** | 97.1251341785982 | 87.9762944684857 | 160.577187197047 | 0 | 2.9515664772542 | 0 | 115.226205281377 | 0.983855492418067 | 6.880 | 3.49425E-13 |
| novel.525 | **-** | 185.048939856066 | 87.9762944684857 | 145.037459403784 | 28.1099478141363 | 22.6286763256155 | 28.3844193909769 | 139.354231242779 | 26.3743478435763 | 2.401 | 3.60787E-13 |
| ENSG00000139354 | **GAS2L3** | 2483.33632547174 | 2715.40315623517 | 2177.63385476253 | 1618.19599583378 | 1631.23240642916 | 1543.90966901635 | 2458.79111215648 | 1597.7793570931 | 0.622 | 3.82059E-13 |
| ENSG00000214160 | **ALG3** | 2090.74630942351 | 1920.65101294683 | 2339.24702381247 | 1350.21449333901 | 1301.6408164691 | 1446.59165967586 | 2116.8814487276 | 1366.14898982799 | 0.632 | 4.44751E-13 |
| ENSG00000135318 | **NT5E** | 843.455112603616 | 769.051203331257 | 797.706026720814 | 506.916058914924 | 355.171832762922 | 342.64049121965 | 803.404114218562 | 401.576127632499 | 0.999 | 5.52523E-13 |
| ENSG00000154310 | **TNIK** | 1313.74523599472 | 1281.09300709166 | 1453.4825395965 | 872.345380498696 | 883.502232191424 | 874.848354800468 | 1349.44026089429 | 876.898655830196 | 0.622 | 6.20911E-13 |
| ENSG00000113838 | **TBCCD1** | 1055.08566812961 | 1150.61131192491 | 1070.16925402935 | 692.441714488224 | 692.634266662319 | 710.624214038387 | 1091.95541136129 | 698.566731729643 | 0.645 | 7.10913E-13 |
| ENSG00000155974 | **GRIP1** | 97.1251341785982 | 99.8382667563715 | 96.3463123182282 | 13.1179756465969 | 18.6932543559433 | 21.2883145432327 | 97.7699044177326 | 17.6998481819243 | 2.471 | 7.97940E-13 |
| ENSG00000171658 | **NMRAL2P** | 1031.57116196006 | 919.302852311143 | 1858.55144407421 | 105.880803433247 | 132.820491476439 | 194.636018680985 | 1269.80848611514 | 144.44577119689 | 3.137 | 8.23549E-13 |
| ENSG00000233058 | **LINC00884** | 184.026570022607 | 240.204938829686 | 221.700116517213 | 75.896859098168 | 82.6438613631176 | 68.9335899495154 | 215.310541789835 | 75.8247701369337 | 1.505 | 8.89683E-13 |
| ENSG00000120217 | **CD274** | 1723.71553921175 | 1336.44887776846 | 1849.22760739825 | 990.407161318069 | 957.291394122779 | 882.958188912175 | 1636.46400812615 | 943.552248117674 | 0.794 | 8.89683E-13 |
| ENSG00000145012 | **LPP** | 1661.35097937076 | 1881.11110532054 | 1980.79730271454 | 1248.08168294765 | 1210.14225567422 | 1133.34931711115 | 1841.08646246861 | 1197.19108524434 | 0.621 | 9.19946E-13 |
| ENSG00000155893 | **PXYLP1** | 1028.50405245968 | 1200.03619645777 | 1170.65949375912 | 731.795641428015 | 648.360769503506 | 714.679131094241 | 1133.06658089219 | 698.278514008587 | 0.698 | 1.35610E-12 |
| ENSG00000075420 | **FNDC3B** | 1185.94900681236 | 1331.50638931517 | 1336.41659022058 | 847.983425726445 | 838.244879540193 | 815.038328226623 | 1284.62399544937 | 833.755544497754 | 0.624 | 1.59640E-12 |
| ENSG00000176928 | **GCNT4** | 118.594900681236 | 99.8382667563715 | 73.5547115547764 | 14.9919721675394 | 11.8062659090168 | 20.2745852792692 | 97.3292929974612 | 15.6909411186085 | 2.636 | 1.87125E-12 |
| ENSG00000270170 | **NCBP2-AS2** | 1827.99726222456 | 1583.57330043274 | 1643.0672186743 | 1095.35096649084 | 1154.06249260639 | 1116.11591962377 | 1684.87926044387 | 1121.84312624034 | 0.587 | 2.01416E-12 |
| ENSG00000116106 | **EPHA4** | 1617.38907653202 | 1750.6294101538 | 1577.8003619426 | 910.762309178016 | 1072.40248673569 | 1140.44542195889 | 1648.60628287614 | 1041.20340595753 | 0.664 | 2.50032E-12 |
| ENSG00000163359 | **COL6A3** | 1010.10139545742 | 1216.84065719894 | 1179.98333043507 | 738.354629251313 | 688.698844692647 | 657.910292312287 | 1135.64179436381 | 694.987922085416 | 0.708 | 2.74016E-12 |
| ENSG00000172667 | **ZMAT3** | 1012.14613512434 | 950.934778412172 | 1069.13327217647 | 652.150789287962 | 611.958116284038 | 648.786728936616 | 1010.73806190433 | 637.631878169538 | 0.664 | 3.06224E-12 |
| ENSG00000078070 | **MCCC1** | 1407.80326067294 | 1439.2526375968 | 1267.00580607735 | 922.00628830367 | 906.13090851704 | 876.875813328395 | 1371.35390144903 | 901.671003383035 | 0.605 | 3.23668E-12 |
| novel.107 | **-** | 180.95946052223 | 113.677234425571 | 279.715100278727 | 22.487958251309 | 41.3219306815588 | 50.6864631981731 | 191.450598408843 | 38.165450710347 | 2.330 | 3.86830E-12 |
| ENSG00000156510 | **HKDC1** | 71.565888342125 | 86.9877967778286 | 109.814076405722 | 16.8659686884818 | 14.757832386271 | 9.12356337567116 | 89.4559205085587 | 13.582454816808 | 2.713 | 4.24678E-12 |
| ENSG00000140379 | **BCL2A1** | 162.55680351997 | 107.746248281629 | 168.86504202012 | 1.87399652094242 | 0 | 0 | 146.38936460724 | 0.624665506980806 | 7.813 | 4.37142E-12 |
| ENSG00000205413 | **SAMD9** | 357.829441710625 | 426.042504673229 | 238.27582616336 | 150.856719935865 | 88.546994317626 | 88.1944459648212 | 340.715924182405 | 109.199386739437 | 1.639 | 7.02946E-12 |
| ENSG00000260422 | **Z97205.2** | 618.533749242652 | 665.258945812257 | 640.23678508242 | 400.098257221207 | 362.058821209849 | 316.2835303566 | 641.343160045776 | 359.480202929218 | 0.834 | 7.33376E-12 |
| ENSG00000152133 | **GPATCH11** | 1654.19439053655 | 1517.34395515871 | 1454.51852144938 | 996.029150880896 | 979.920070448395 | 1088.74522949676 | 1542.01895571488 | 1021.56481694202 | 0.594 | 7.46153E-12 |
| ENSG00000178031 | **ADAMTSL1** | 3601.8089232758 | 3077.19331101569 | 4251.66952423665 | 2257.22880947514 | 2280.57703142508 | 2378.20885325828 | 3643.55725284271 | 2305.33823138617 | 0.660 | 7.86116E-12 |
| ENSG00000272622 | **AC010735.2** | 300.576731036925 | 264.917381096114 | 381.241321861376 | 138.675742549739 | 133.804346968857 | 124.688699467506 | 315.578477998138 | 132.389596328701 | 1.252 | 8.20588E-12 |
| ENSG00000254303 | **AC037486.1** | 90.9909151778446 | 92.9187829217715 | 72.5187297018922 | 8.43298434424089 | 16.7255433711071 | 15.2059389594519 | 85.4761426005028 | 13.4548222249333 | 2.674 | 8.71007E-12 |
| ENSG00000124813 | **RUNX2** | 1695.0891838749 | 1885.06509608317 | 1470.05824924264 | 1143.13787777488 | 984.839347910485 | 977.235010460777 | 1683.40417640024 | 1035.07074538205 | 0.701 | 8.89461E-12 |
| ENSG00000145014 | **TMEM44** | 1263.64911415524 | 1337.43737545911 | 1064.98934476493 | 758.96859098168 | 785.116682949618 | 708.59675551046 | 1222.02527812643 | 750.894009813919 | 0.703 | 9.30578E-12 |
| ENSG00000251493 | **FOXD1** | 5159.90054946721 | 3497.30482954497 | 4431.9303666385 | 1967.69634698954 | 1783.73000775396 | 1648.32378320459 | 4363.04524855023 | 1799.91671264936 | 1.277 | 9.90195E-12 |
| ENSG00000166401 | **SERPINB8** | 1372.02031650188 | 1167.41577266609 | 1108.50058258607 | 809.566497047125 | 617.861249238546 | 686.294711703264 | 1215.97889058468 | 704.574152662978 | 0.787 | 1.08134E-11 |
| ENSG00000154102 | **C16orf74** | 522.430984897512 | 423.077011601257 | 441.328269328658 | 247.367540764399 | 234.1576071955 | 236.198918503487 | 462.278755275809 | 239.241355487795 | 0.950 | 1.21673E-11 |
| ENSG00000198682 | **PAPSS2** | 259.681937698568 | 251.078413426914 | 229.987971340287 | 112.439791256545 | 102.320971211479 | 75.0159655332962 | 246.91610748859 | 96.5922426671068 | 1.352 | 1.22279E-11 |
| ENSG00000117152 | **RGS4** | 65.4316693413714 | 46.4593914608857 | 106.70613084707 | 0.93699826047121 | 2.9515664772542 | 0 | 72.8657305497757 | 1.29618824590847 | 5.805 | 3.62066E-11 |
| ENSG00000260823 | **AC026461.3** | 65.4316693413714 | 64.2523498927143 | 70.4467659961239 | 7.49598608376968 | 10.8224104165987 | 5.06864631981731 | 66.7102617434032 | 7.79568094006191 | 3.094 | 3.73916E-11 |
| ENSG00000249992 | **TMEM158** | 286.2635533685 | 205.607519656686 | 183.368787960499 | 89.9518330052361 | 78.7084393934454 | 57.7825680459173 | 225.079953661895 | 75.4809468148663 | 1.574 | 4.55635E-11 |
| ENSG00000106665 | **CLIP2** | 1595.91931002939 | 1760.51438706037 | 1460.73441256669 | 1125.33491082592 | 1024.19356760721 | 1018.79791028328 | 1605.72270321881 | 1056.1087962388 | 0.604 | 4.56031E-11 |
| ENSG00000214135 | **AC132008.2** | 1556.04688652449 | 1919.66251525617 | 1848.19162554537 | 1077.54799954189 | 1219.9808105984 | 1159.7062779742 | 1774.63367577534 | 1152.41169603817 | 0.623 | 4.77129E-11 |
| ENSG00000182197 | **EXT1** | 1221.73195098342 | 1240.56460177471 | 1435.87084809747 | 837.676444861262 | 874.647532759662 | 843.4227476176 | 1299.38913361853 | 851.915575079508 | 0.609 | 5.97347E-11 |
| ENSG00000101307 | **SIRPB1** | 62.3645598409946 | 73.1488291086286 | 78.7346208191972 | 0 | 0 | 0 | 71.4160032562735 | 0 | 8.633 | 6.07184E-11 |
| ENSG00000120279 | **MYCT1** | 106.326462679729 | 83.0338060152 | 96.3463123182282 | 25.2989530327227 | 10.8224104165987 | 18.2471267513423 | 95.2355270043856 | 18.1228300668879 | 2.389 | 6.24531E-11 |
| novel.884 | **-** | 55.2079710067821 | 50.4133822235143 | 74.5906934076606 | 1.87399652094242 | 1.96771098483613 | 1.01372926396346 | 60.0706822126523 | 1.61847892324734 | 5.205 | 6.67621E-11 |
| ENSG00000128340 | **RAC2** | 85.87906601055 | 94.8957783030857 | 82.8785482307339 | 16.8659686884818 | 18.6932543559433 | 18.2471267513423 | 87.8844641814566 | 17.9354499319225 | 2.294 | 7.45424E-11 |
| novel.66 | **-** | 104.281723012811 | 64.2523498927143 | 109.814076405722 | 21.5509599908378 | 13.7739768938529 | 14.1922096954885 | 92.7827164370825 | 16.5057155267264 | 2.485 | 9.97222E-11 |
| ENSG00000068885 | **IFT80** | 1012.14613512434 | 1163.46178190346 | 1166.51556634758 | 704.62269187435 | 750.681740714985 | 722.788965205948 | 1114.04116112513 | 726.031132598428 | 0.618 | 1.17948E-10 |
| ENSG00000113594 | **LIFR** | 344.538633875659 | 476.455886896743 | 406.104886330596 | 206.139617303666 | 188.900254544269 | 210.8556869044 | 409.033135700999 | 201.965186250778 | 1.019 | 1.65356E-10 |
| ENSG00000173334 | **TRIB1** | 2460.84418913564 | 2096.6036018838 | 2871.74169619493 | 1430.79634373954 | 1629.26469544432 | 1637.17276130099 | 2476.39649573812 | 1565.74460016162 | 0.661 | 2.29989E-10 |
| ENSG00000255794 | **RMST** | 127.796229182366 | 132.458690548057 | 110.850058258607 | 24.3619547722515 | 31.4833757573781 | 44.6040876143923 | 123.701659329677 | 33.4831393813406 | 1.891 | 2.39317E-10 |
| ENSG00000164086 | **DUSP7** | 1510.04024401884 | 1266.2655417318 | 1336.41659022058 | 929.50227438744 | 937.614284274418 | 838.354101297783 | 1370.90745865707 | 901.82355331988 | 0.604 | 2.71815E-10 |
| ENSG00000248323 | **LUCAT1** | 749.397087925394 | 689.971388078686 | 1101.24870961588 | 456.318152849479 | 443.718827080548 | 387.244578834042 | 846.872395206653 | 429.093852921357 | 0.980 | 3.18870E-10 |
| ENSG00000222041 | **CYTOR** | 1258.53726498794 | 1190.1512195512 | 1269.07776978311 | 829.243460517021 | 711.327521018262 | 881.944459648212 | 1239.25541810742 | 807.505147061165 | 0.618 | 3.21645E-10 |
| ENSG00000205464 | **ATP6AP1L** | 423.261111051996 | 481.398375350029 | 404.032922624828 | 258.611519890054 | 223.335196778901 | 196.663477208912 | 436.230803008951 | 226.203397959289 | 0.946 | 3.61302E-10 |
| novel.600 | **-** | 254.570088531273 | 204.619021966029 | 197.872533900877 | 59.9678886701574 | 106.256393181151 | 54.7413802540269 | 219.020548132726 | 73.6552207017785 | 1.572 | 3.96480E-10 |
| ENSG00000184809 | **B3GALT5-AS1** | 392.590016048228 | 438.892974651772 | 411.284795595017 | 236.123561638745 | 227.270618748573 | 225.047896599889 | 414.255928765006 | 229.480692329069 | 0.852 | 7.47267E-10 |
| ENSG00000134954 | **ETS1** | 3814.46184863526 | 2621.49587562274 | 3860.06838384643 | 1599.45603062435 | 1337.05961419615 | 1340.1500869597 | 3432.00870270148 | 1425.55524392673 | 1.267 | 9.88095E-10 |
| ENSG00000168743 | **NPNT** | 417.126892051243 | 464.593914608857 | 380.205340008492 | 230.501572075918 | 220.383630301647 | 236.198918503487 | 420.642048889531 | 229.028040293684 | 0.878 | 1.02375E-09 |
| ENSG00000028277 | **POU2F2** | 208.563446025621 | 204.619021966029 | 194.764588342225 | 90.8888312657073 | 91.4985607948802 | 70.9610484774423 | 202.649018777958 | 84.4494801793433 | 1.261 | 1.24203E-09 |
| novel.53 | **-** | 79.7448470097964 | 64.2523498927143 | 49.7271289384404 | 11.2439791256545 | 8.8546994317626 | 8.10983411170769 | 64.574775280317 | 9.40283755637494 | 2.776 | 2.05143E-09 |
| ENSG00000247809 | **NR2F2-AS1** | 200.38448735795 | 212.527003491286 | 186.476733519151 | 95.5738225680634 | 69.8537399616828 | 82.1120703810404 | 199.796074789462 | 82.5132109702622 | 1.275 | 2.15869E-09 |
| ENSG00000251893 | **RF00156** | 61.3421900075357 | 48.4363868422 | 53.8710563499771 | 2.81099478141363 | 4.91927746209034 | 9.12356337567116 | 54.5498777332376 | 5.61794520639171 | 3.293 | 2.16795E-09 |
| ENSG00000197632 | **SERPINB2** | 72.5882581755839 | 31.6319261010286 | 114.993985670143 | 4.68499130235605 | 7.87084393934454 | 10.1372926396346 | 73.0713899822519 | 7.56437596044507 | 3.278 | 2.85973E-09 |
| ENSG00000230795 | **HLA-K** | 444.730877554634 | 529.834762192229 | 459.975942680573 | 292.343457267017 | 241.044595642426 | 273.706901270135 | 478.180527475812 | 269.031651393193 | 0.830 | 3.65813E-09 |
| ENSG00000198795 | **ZNF521** | 83.8343263436321 | 127.516202094771 | 85.9864937893865 | 30.9209425955499 | 19.6771098483613 | 19.2608560153058 | 99.1123407425967 | 23.286302819739 | 2.086 | 3.70330E-09 |
| ENSG00000276116 | **FUT8-AS1** | 291.375402535795 | 316.319261010286 | 308.722592159484 | 178.02966948953 | 137.739768938529 | 142.935826218848 | 305.472418568521 | 152.901754882302 | 0.997 | 4.97293E-09 |
| ENSG00000132535 | **DLG4** | 313.867538871891 | 311.376772557 | 256.923499515275 | 162.100699061519 | 135.772057953693 | 135.839721371104 | 294.055936981389 | 144.570826128772 | 1.023 | 6.54365E-09 |
| ENSG00000101096 | **NFATC2** | 61.3421900075357 | 62.2753545114 | 41.439274115367 | 0 | 2.9515664772542 | 0 | 55.0189395447676 | 0.983855492418067 | 5.815 | 7.42989E-09 |
| ENSG00000273669 | **AC015819.1** | 97.1251341785982 | 80.0683129432286 | 76.6626571134289 | 18.7399652094242 | 23.6125318180336 | 19.2608560153058 | 84.6187014117519 | 20.5377843475879 | 2.043 | 8.91778E-09 |
| ENSG00000057019 | **DCBLD2** | 490.737520060286 | 397.376071644172 | 417.500686712322 | 261.422514671468 | 239.07688465759 | 233.157730711596 | 435.20475947226 | 244.552376680218 | 0.831 | 1.12263E-08 |
| ENSG00000077092 | **RARB** | 88.9461755109268 | 107.746248281629 | 87.0224756422706 | 24.3619547722515 | 28.5318092801239 | 25.3432315990865 | 94.571633144942 | 26.0789985504873 | 1.860 | 1.22884E-08 |
| ENSG00000187193 | **MT1X** | 1247.29119681989 | 1766.44537320431 | 904.412157567884 | 407.594243304976 | 380.752075565792 | 399.409330001604 | 1306.04957586403 | 395.918549624124 | 1.722 | 1.29266E-08 |
| ENSG00000283646 | **LINC02009** | 39.8724235048982 | 61.2868568207429 | 35.2233829980619 | 0.93699826047121 | 0.983855492418067 | 3.04118779189039 | 45.4608877745677 | 1.65401384825989 | 4.800 | 1.36072E-08 |
| ENSG00000227038 | **GTF2IP7** | 72.5882581755839 | 77.1028198712572 | 37.2953467038303 | 7.49598608376968 | 6.88698844692647 | 12.1647511675615 | 62.3288082502238 | 8.84924189941923 | 2.822 | 2.14450E-08 |
| ENSG00000166311 | **SMPD1** | 665.562761581762 | 595.0756097756 | 590.509656143979 | 425.397210253929 | 362.058821209849 | 349.736596067394 | 617.049342500447 | 379.064209177057 | 0.702 | 2.49031E-08 |
| ENSG00000204103 | **MAFB** | 59.2974503406178 | 65.2408475833714 | 41.439274115367 | 11.2439791256545 | 6.88698844692647 | 4.05491705585385 | 55.3258573464521 | 7.39529487614494 | 2.893 | 3.45662E-08 |
| ENSG00000240024 | **LINC00888** | 389.522906547852 | 443.835463105057 | 361.557666656577 | 221.131589471206 | 226.286763256155 | 232.144001447633 | 398.305345436495 | 226.520784724998 | 0.815 | 3.53583E-08 |
| ENSG00000257219 | **LINC02407** | 37.8276838379803 | 31.6319261010286 | 38.3313285567145 | 0 | 0 | 0 | 35.9303128319078 | 0 | 7.642 | 4.29611E-08 |
| ENSG00000123104 | **ITPR2** | 295.46488186963 | 355.859168636571 | 295.25482807199 | 152.730716456807 | 190.867965529105 | 141.922096954885 | 315.526292859397 | 161.840259646932 | 0.964 | 6.07376E-08 |
| ENSG00000135617 | **PRADC1** | 574.571846403918 | 594.087112084943 | 556.322254998802 | 373.862305928013 | 347.300988823578 | 383.189661778189 | 574.993737829221 | 368.117652176593 | 0.644 | 6.32912E-08 |
| ENSG00000114529 | **C3orf52** | 519.363875397136 | 409.238043932057 | 541.818509058423 | 318.579408560211 | 251.867006059025 | 229.102813655742 | 490.140142795872 | 266.516409424993 | 0.877 | 6.95214E-08 |
| ENSG00000258791 | **LINC00520** | 37.8276838379803 | 48.4363868422 | 34.1874011451778 | 2.81099478141363 | 0 | 3.04118779189039 | 40.1504906084527 | 1.95072752443467 | 4.362 | 7.09912E-08 |
| ENSG00000166670 | **MMP10** | 122.684380015071 | 62.2753545114 | 116.029967523028 | 30.9209425955499 | 21.6448208331975 | 24.3295023351231 | 100.329900683166 | 25.6317552546235 | 1.965 | 7.98578E-08 |
| ENSG00000092969 | **TGFB2** | 361.918921044461 | 408.2495462414 | 465.155851944994 | 246.430542503928 | 236.125318180336 | 228.089084391779 | 411.774773076952 | 236.881648358681 | 0.797 | 1.16091E-07 |
| ENSG00000073150 | **PANX2** | 907.864412111528 | 635.604015092543 | 916.843939802494 | 469.436128496076 | 421.090150754933 | 541.331426956489 | 820.104122335522 | 477.285902069166 | 0.781 | 1.52253E-07 |
| ENSG00000185112 | **FAM43A** | 529.587573731725 | 410.226541622714 | 350.161866274851 | 203.328622522253 | 232.189896210664 | 250.391128198975 | 429.99199387643 | 228.636548977297 | 0.912 | 1.53252E-07 |
| ENSG00000259039 | **AL161804.1** | 89.9685453443857 | 73.1488291086286 | 89.094439348039 | 30.9209425955499 | 13.7739768938529 | 19.2608560153058 | 84.0706046003511 | 21.3185918349029 | 1.974 | 1.73980E-07 |
| ENSG00000222032 | **AC112721.2** | 37.8276838379803 | 31.6319261010286 | 44.5472196740195 | 1.87399652094242 | 3.93542196967227 | 2.02745852792692 | 38.0022765376761 | 2.61229233951387 | 3.865 | 1.89150E-07 |
| ENSG00000142694 | **EVA1B** | 647.160104579502 | 596.064107466257 | 527.314763118045 | 364.492323323301 | 384.687497535464 | 375.079827666481 | 590.179658387935 | 374.753216175082 | 0.656 | 1.89150E-07 |
| ENSG00000058866 | **DGKG** | 516.296765896759 | 456.6859330836 | 572.897964644948 | 335.445377248693 | 313.849902081363 | 298.036403605258 | 515.293554541769 | 315.777227645105 | 0.705 | 2.61579E-07 |
| novel.171 | **-** | 354.762332210248 | 398.364569334829 | 331.514192922936 | 209.887610345551 | 221.367485794065 | 191.594830889094 | 361.547031489337 | 207.616642342903 | 0.800 | 2.79100E-07 |
| ENSG00000238178 | **AC078993.1** | 83.8343263436321 | 75.1258244899429 | 104.634167141302 | 19.6769634698954 | 33.4510867422143 | 19.2608560153058 | 87.8647726582922 | 24.1296354091385 | 1.864 | 2.83638E-07 |
| novel.802 | **-** | 461.088794889977 | 347.951187111314 | 432.004432652701 | 252.052532066755 | 209.561219885048 | 243.295023351231 | 413.681471551331 | 234.969591767678 | 0.815 | 3.19371E-07 |
| ENSG00000255150 | **EID3** | 615.466639742275 | 394.4105785722 | 712.755514784312 | 245.493544243457 | 281.382670831567 | 342.64049121965 | 574.210911032929 | 289.838902098225 | 0.987 | 3.95357E-07 |
| ENSG00000151812 | **SLC35F4** | 82.8119565101732 | 143.332165145286 | 65.266856731703 | 30.9209425955499 | 20.6609653407794 | 23.3157730711596 | 97.1369927957206 | 24.965893669163 | 1.959 | 4.14706E-07 |
| ENSG00000167103 | **PIP5KL1** | 439.619028387339 | 432.961988507829 | 369.84552147965 | 267.044504234295 | 248.915439581771 | 223.020438071962 | 414.142179458273 | 246.326793962676 | 0.749 | 5.20549E-07 |
| ENSG00000276733 | **MIR7158** | 33.7382045041446 | 69.194838346 | 37.2953467038303 | 0 | 1.96771098483613 | 0 | 46.7427965179916 | 0.655903661612045 | 6.165 | 7.20087E-07 |
| ENSG00000143702 | **CEP170** | 1087.8015028003 | 701.833360366572 | 944.815449830367 | 596.867891920161 | 561.781486170716 | 463.274273631302 | 911.483437665746 | 540.641217240726 | 0.753 | 7.32341E-07 |
| ENSG00000261379 | **AC010735.1** | 119.617270514695 | 133.447188238714 | 156.43325978551 | 51.5349043259165 | 40.3380751891408 | 66.9061314215885 | 136.499239512973 | 52.9263703122153 | 1.368 | 8.31493E-07 |
| ENSG00000100417 | **PMM1** | 322.046497539562 | 309.399777175686 | 252.779572103739 | 182.714660791886 | 141.675190908202 | 148.004472538665 | 294.741948939662 | 157.464774746251 | 0.903 | 9.30971E-07 |
| ENSG00000130270 | **ATP8B3** | 366.008400378296 | 406.272550860086 | 339.802047746009 | 215.509599908378 | 239.07688465759 | 202.745852792692 | 370.694332994797 | 219.110779119554 | 0.759 | 1.01427E-06 |
| ENSG00000280106 | **AC008555.8** | 116.550161014318 | 138.389676692 | 139.857550139364 | 51.5349043259165 | 62.9667515147563 | 46.6315461423192 | 131.599129281894 | 53.711067327664 | 1.293 | 1.23181E-06 |
| ENSG00000141574 | **SECTM1** | 251.502979030896 | 282.710339527943 | 140.893531992248 | 104.006806912304 | 91.4985607948802 | 87.1807167008577 | 225.035616850362 | 94.2286948026807 | 1.256 | 1.23876E-06 |
| ENSG00000185760 | **KCNQ5** | 55.2079710067821 | 67.2178429646857 | 70.4467659961239 | 16.8659686884818 | 10.8224104165987 | 19.2608560153058 | 64.2908599891972 | 15.6497450401288 | 2.039 | 1.33894E-06 |
| ENSG00000166002 | **SMCO4** | 606.265311241144 | 535.765748336172 | 490.019416414214 | 337.319373769635 | 375.832798103702 | 323.379635204344 | 544.01682533051 | 345.510602359227 | 0.655 | 1.38017E-06 |
| ENSG00000108175 | **ZMIZ1** | 515.2743960633 | 603.972088991514 | 452.724069710384 | 295.154452048431 | 342.381711361487 | 331.489469316052 | 523.990184921733 | 323.00854424199 | 0.699 | 1.58203E-06 |
| ENSG00000166888 | **STAT6** | 1485.50336801582 | 1419.48268378366 | 1343.66846319077 | 1151.57086211912 | 869.728255297571 | 803.887306323025 | 1416.21817166342 | 941.728807913238 | 0.588 | 1.59094E-06 |
| ENSG00000172061 | **LRRC15** | 38.8500536714393 | 27.6779353384 | 36.2593648509461 | 0 | 0.983855492418067 | 2.02745852792692 | 34.2624512869285 | 1.003771340115 | 5.126 | 1.82482E-06 |
| novel.526 | **-** | 24.5368760030143 | 49.4248845328572 | 32.1154374394094 | 0 | 2.9515664772542 | 0 | 35.3590659917603 | 0.983855492418067 | 5.177 | 2.12175E-06 |
| ENSG00000158373 | **HIST1H2BD** | 341.471524375282 | 350.916680183286 | 454.796033416153 | 222.068587731677 | 216.448208331975 | 231.130272183669 | 382.394745991573 | 223.215689415774 | 0.776 | 2.12952E-06 |
| ENSG00000106123 | **EPHB6** | 250.480609197437 | 257.997897261514 | 367.773557773882 | 151.793718196336 | 133.804346968857 | 167.265328553971 | 292.084021410945 | 150.954464573055 | 0.952 | 2.35250E-06 |
| ENSG00000185022 | **MAFF** | 1336.23737233082 | 949.946280721515 | 1395.46755583498 | 743.976618814141 | 802.826081813143 | 840.38155982571 | 1227.21706962911 | 795.728086817664 | 0.625 | 2.57002E-06 |
| ENSG00000184678 | **HIST2H2BE** | 239.234541029389 | 289.629823362543 | 274.535191014306 | 116.18778429843 | 162.336156248981 | 151.045660330556 | 267.799851802079 | 143.189866959322 | 0.905 | 2.65180E-06 |
| ENSG00000169860 | **P2RY1** | 260.704307532027 | 370.686633996429 | 277.643136572959 | 148.045725154451 | 172.174711173162 | 171.320245609825 | 303.011359367138 | 163.846893979146 | 0.889 | 2.92064E-06 |
| ENSG00000229320 | **KRT8P12** | 484.603301059532 | 460.639923846229 | 522.134853853624 | 306.398431174086 | 339.430144884233 | 310.201154772819 | 489.126026253128 | 318.676576943713 | 0.618 | 3.12404E-06 |
| ENSG00000039600 | **SOX30** | 200.38448735795 | 199.676533512743 | 149.181386815321 | 102.132810391362 | 69.8537399616828 | 80.0846118531135 | 183.080802562005 | 84.023720735386 | 1.122 | 3.18697E-06 |
| ENSG00000133401 | **PDZD2** | 53.1632313398643 | 61.2868568207429 | 35.2233829980619 | 12.1809773861257 | 6.88698844692647 | 9.12356337567116 | 49.8911570528897 | 9.39717640290779 | 2.406 | 3.64754E-06 |
| ENSG00000181826 | **RELL1** | 515.2743960633 | 496.225840709886 | 420.608632270975 | 323.264399862567 | 286.301948293658 | 303.105049925075 | 477.36962301472 | 304.223799360433 | 0.650 | 4.11178E-06 |
| ENSG00000113916 | **BCL6** | 375.209728879427 | 358.824661708543 | 294.218846219106 | 188.336650354713 | 184.964832574597 | 225.047896599889 | 342.751078935692 | 199.449793176399 | 0.782 | 4.32795E-06 |
| ENSG00000149968 | **MMP3** | 166.646282853805 | 55.3558706768 | 66.3028385845872 | 4.68499130235605 | 4.91927746209034 | 0 | 96.1016640383975 | 3.20142292148213 | 4.898 | 4.63749E-06 |
| ENSG00000153071 | **DAB2** | 125.751489515448 | 149.263151289229 | 113.958003817259 | 53.408900846859 | 60.0151850375021 | 54.7413802540269 | 129.657548207312 | 56.0551553794627 | 1.211 | 5.51455E-06 |
| ENSG00000140678 | **ITGAX** | 50.0961218394875 | 38.5514099356286 | 25.8995463221044 | 8.43298434424089 | 2.9515664772542 | 1.01372926396346 | 38.1823593657401 | 4.13276002848618 | 3.187 | 6.26184E-06 |
| ENSG00000114771 | **AADAC** | 841.410372936698 | 732.476788776943 | 1181.01931228796 | 593.119898878276 | 529.31425492092 | 579.8531389871 | 918.302158000533 | 567.429097595432 | 0.694 | 7.01766E-06 |
| novel.88 | **-** | 27.6039855033911 | 21.7469491944571 | 48.6911470855562 | 0.93699826047121 | 0 | 0 | 32.6806939278015 | 0.312332753490403 | 6.543 | 7.60682E-06 |
| ENSG00000222022 | **AC112721.1** | 27.6039855033911 | 32.6204237916857 | 24.8635644692202 | 0.93699826047121 | 0.983855492418067 | 2.02745852792692 | 28.3626579214323 | 1.3161040936054 | 4.442 | 8.40822E-06 |
| ENSG00000112837 | **TBX18** | 27.6039855033911 | 33.6089214823429 | 23.827582616336 | 0.93699826047121 | 1.96771098483613 | 2.02745852792692 | 28.3468298673566 | 1.64405592441142 | 4.119 | 8.77452E-06 |
| ENSG00000256235 | **SMIM3** | 980.452670287112 | 1112.05990198929 | 708.611587372775 | 615.607857129585 | 621.796671208218 | 524.09802946911 | 933.708053216391 | 587.167519268971 | 0.669 | 8.86427E-06 |
| ENSG00000165617 | **DACT1** | 26.5816156699321 | 24.7124422664286 | 47.655165232672 | 5.62198956282726 | 0.983855492418067 | 2.02745852792692 | 32.9830743896776 | 2.87776786105742 | 3.500 | 9.42126E-06 |
| ENSG00000174007 | **CEP19** | 384.411057380557 | 387.4910947376 | 322.190356246978 | 229.564573815446 | 226.286763256155 | 226.061625863852 | 364.697502788378 | 227.304320978485 | 0.682 | 1.06694E-05 |
| ENSG00000122136 | **OBP2A** | 23.5145061695554 | 16.8044607411714 | 16.5757096461468 | 0 | 0 | 0 | 18.9648921856245 | 0 | 6.720 | 1.10540E-05 |
| ENSG00000185222 | **TCEAL9** | 1083.71202346646 | 1267.25403942246 | 860.900919746749 | 758.96859098168 | 716.246798480353 | 659.937750840214 | 1070.62232754522 | 711.717713434082 | 0.589 | 1.11186E-05 |
| ENSG00000144655 | **CSRNP1** | 357.829441710625 | 314.342265628972 | 332.55017477582 | 214.572601647907 | 197.754953976031 | 211.869416168364 | 334.907294038472 | 208.065657264101 | 0.686 | 1.27305E-05 |
| ENSG00000119227 | **PIGZ** | 253.547718697814 | 329.169730988829 | 288.0029551018 | 169.596685145289 | 168.239289203489 | 172.333974873789 | 290.240134929481 | 170.056649740856 | 0.772 | 1.28866E-05 |
| ENSG00000146090 | **RASGEF1C** | 344.538633875659 | 324.227242535543 | 324.262319952747 | 218.320594689792 | 205.625797915376 | 196.663477208912 | 331.009398787983 | 206.869956604693 | 0.677 | 1.46283E-05 |
| ENSG00000204335 | **SP5** | 41.9171631718161 | 50.4133822235143 | 23.827582616336 | 4.68499130235605 | 5.9031329545084 | 7.09610484774423 | 38.7193760038888 | 5.89474303486956 | 2.721 | 1.49694E-05 |
| ENSG00000100450 | **GZMH** | 39.8724235048982 | 22.7354468851143 | 22.7916007634518 | 0.93699826047121 | 1.96771098483613 | 3.04118779189039 | 28.4664903844881 | 1.98196567906591 | 3.859 | 1.53716E-05 |
| ENSG00000143369 | **ECM1** | 132.908078349661 | 115.654229806886 | 90.1304212009232 | 53.408900846859 | 43.289641666395 | 41.5628998225019 | 112.89757645249 | 46.087147445252 | 1.291 | 1.75501E-05 |
| ENSG00000137198 | **GMPR** | 534.69942289902 | 450.754946939657 | 416.464704859438 | 324.201398123039 | 284.334237308821 | 292.96775728544 | 467.306358232705 | 300.501130905767 | 0.636 | 1.81835E-05 |
| ENSG00000236098 | **AC097059.2** | 161.534433686511 | 133.447188238714 | 158.505223491279 | 89.9518330052361 | 58.047474052666 | 57.7825680459173 | 151.162281805501 | 68.5939583679398 | 1.136 | 1.82407E-05 |
| ENSG00000248719 | **AC021127.1** | 165.623913020346 | 237.239445757714 | 188.54869722492 | 102.132810391362 | 104.288682196315 | 99.3454678684193 | 197.137352000993 | 101.922320152032 | 0.952 | 1.90996E-05 |
| ENSG00000258976 | **AC013451.2** | 53.1632313398643 | 17.7929584318286 | 48.6911470855562 | 8.43298434424089 | 3.93542196967227 | 3.04118779189039 | 39.882445619083 | 5.13653136860118 | 2.944 | 1.96411E-05 |
| ENSG00000072422 | **RHOBTB1** | 290.353032702336 | 275.790855693343 | 250.70760839797 | 168.659686884818 | 154.465312309637 | 164.224140762081 | 272.28383226455 | 162.449713318845 | 0.745 | 2.06274E-05 |
| ENSG00000121797 | **CCRL2** | 27.6039855033911 | 26.6894376477429 | 25.8995463221044 | 1.87399652094242 | 0.983855492418067 | 0 | 26.7309898244128 | 0.952617337786829 | 4.779 | 2.20134E-05 |
| ENSG00000185338 | **SOCS1** | 158.467324186134 | 106.757750590971 | 175.080933137425 | 69.3378712748695 | 71.8214509465189 | 51.7001924621366 | 146.768669304844 | 64.2865048945083 | 1.188 | 2.47641E-05 |
| ENSG00000170439 | **METTL7B** | 84.856696177091 | 100.826764447029 | 93.2383667595757 | 38.4169286793196 | 40.3380751891408 | 31.4256071828673 | 92.9739424612318 | 36.7268703504426 | 1.339 | 2.60641E-05 |
| ENSG00000102265 | **TIMP1** | 5018.81351244988 | 3336.17970596786 | 5419.22107243712 | 2399.65254506677 | 2301.23799676586 | 2338.67341196371 | 4591.40476361828 | 2346.52131793211 | 0.968 | 2.61504E-05 |
| ENSG00000179630 | **LACC1** | 321.024127706103 | 339.0547078954 | 342.909993304662 | 224.87958251309 | 218.415919316811 | 178.416350457569 | 334.329609635388 | 207.237284095823 | 0.689 | 2.61504E-05 |
| ENSG00000283154 | **IQCJ-SCHIP1** | 348.628113209495 | 255.032404189543 | 387.457212978681 | 211.761606866493 | 164.303867233817 | 183.484996777387 | 330.372576792573 | 186.516823625899 | 0.823 | 2.61992E-05 |
| ENSG00000225760 | **LINC00431** | 280.129334367746 | 390.456587809572 | 371.917485185419 | 228.627575554975 | 187.916399051851 | 200.718394264765 | 347.501135787579 | 205.754122957197 | 0.756 | 2.67669E-05 |
| ENSG00000109220 | **CHIC2** | 389.522906547852 | 292.595316434514 | 410.248813742133 | 236.123561638745 | 215.464352839557 | 203.759582056656 | 364.122345574833 | 218.449165511652 | 0.736 | 2.67669E-05 |
| novel.205 | **-** | 281.151704201205 | 223.400478088514 | 227.916007634518 | 150.856719935865 | 125.933503029513 | 137.867179899031 | 244.156063308079 | 138.219134288136 | 0.820 | 2.82424E-05 |
| ENSG00000124635 | **HIST1H2BJ** | 168.691022520723 | 180.895077390257 | 180.260842401846 | 104.006806912304 | 83.6277168555357 | 92.249363020675 | 176.615647437609 | 93.294628929505 | 0.920 | 2.98868E-05 |
| ENSG00000076641 | **PAG1** | 39.8724235048982 | 44.4823960795714 | 44.5472196740195 | 7.49598608376968 | 6.88698844692647 | 13.178480431525 | 42.9673464194964 | 9.18715165407372 | 2.231 | 3.38260E-05 |
| ENSG00000160712 | **IL6R** | 141.087037017332 | 145.3091605266 | 167.829060167236 | 83.3928451819377 | 79.6922948858634 | 52.7139217261 | 151.408419237056 | 71.933020597967 | 1.071 | 3.86172E-05 |
| ENSG00000196639 | **HRH1** | 531.632313398643 | 494.248845328572 | 463.083888239226 | 378.547297230369 | 305.979058142019 | 300.063862133185 | 496.321682322147 | 328.196739168524 | 0.596 | 3.99815E-05 |
| ENSG00000213123 | **TCTEX1D2** | 141.087037017332 | 109.723243662943 | 91.1664030538073 | 47.7869112840317 | 50.1766301133214 | 48.6590046702462 | 113.992227911361 | 48.8741820225331 | 1.222 | 4.31820E-05 |
| ENSG00000105711 | **SCN1B** | 47.0290123391107 | 62.2753545114 | 41.439274115367 | 6.55898782329847 | 18.6932543559433 | 9.12356337567116 | 50.2478803219592 | 11.4586018516376 | 2.137 | 4.39517E-05 |
| ENSG00000129675 | **ARHGEF6** | 84.856696177091 | 126.527704404114 | 97.3822941711124 | 44.0389182421469 | 47.2250636360672 | 33.4530657107942 | 102.922231584106 | 41.5723491963361 | 1.307 | 4.59614E-05 |
| ENSG00000232104 | **RFX3-AS1** | 306.710950037678 | 367.721140924457 | 286.966973248916 | 218.320594689792 | 192.835676513941 | 165.237870026044 | 320.466354737017 | 192.131380409926 | 0.737 | 4.78506E-05 |
| ENSG00000250271 | **AC068647.2** | 292.397772369253 | 258.986394952172 | 424.752559682512 | 176.155672968587 | 164.303867233817 | 201.732123528729 | 325.378909001312 | 180.730554577045 | 0.848 | 4.79150E-05 |
| ENSG00000278607 | **AC015819.2** | 30.6710950037678 | 26.6894376477429 | 25.8995463221044 | 2.81099478141363 | 3.93542196967227 | 3.04118779189039 | 27.7533596578717 | 3.26253484765876 | 3.090 | 5.37931E-05 |
| ENSG00000153132 | **CLGN** | 299.554361203466 | 255.032404189543 | 286.966973248916 | 189.273648615184 | 152.4976013248 | 166.251599290008 | 280.517912880642 | 169.340949743331 | 0.727 | 5.41984E-05 |
| ENSG00000224273 | **AC005077.2** | 34.7605743376036 | 42.5054006982572 | 24.8635644692202 | 5.62198956282726 | 6.88698844692647 | 4.05491705585385 | 34.043179835027 | 5.52129835520252 | 2.623 | 5.73730E-05 |
| ENSG00000184524 | **CEND1** | 252.525348864355 | 229.331464232457 | 242.419753574897 | 148.045725154451 | 134.788202461275 | 150.031931066592 | 241.425522223903 | 144.288619560773 | 0.742 | 5.97238E-05 |
| ENSG00000166025 | **AMOTL1** | 54.1856011733232 | 69.194838346 | 74.5906934076606 | 7.49598608376968 | 22.6286763256155 | 26.35696086305 | 65.9903776423279 | 18.8272077574784 | 1.817 | 7.95587E-05 |
| ENSG00000189410 | **SH2D5** | 277.06222486737 | 267.882874168086 | 269.355281749885 | 186.462653833771 | 158.400734279309 | 157.128035914337 | 271.43346026178 | 167.330474675805 | 0.697 | 8.29210E-05 |
| ENSG00000049247 | **UTS2** | 5394.02324132931 | 6299.69578255797 | 4299.32468946932 | 3292.61188729583 | 3264.43252384315 | 2907.37552904721 | 5331.01457111887 | 3154.80664672873 | 0.757 | 8.50280E-05 |
| ENSG00000159899 | **NPR2** | 391.56764621477 | 398.364569334829 | 496.235307531519 | 237.060559899216 | 296.140503217838 | 293.981486549404 | 428.722507693706 | 275.727516555486 | 0.637 | 9.27756E-05 |
| ENSG00000151388 | **ADAMTS12** | 20.4473966691786 | 13.8389676692 | 8.2878548230734 | 0 | 0 | 0 | 14.1914063871507 | 0 | 6.303 | 1.19648E-04 |
| ENSG00000273143 | **AL355512.1** | 27.6039855033911 | 15.8159630505143 | 20.7196370576835 | 0 | 0 | 1.01372926396346 | 21.3798618705296 | 0.337909754654487 | 5.931 | 1.19902E-04 |
| ENSG00000123570 | **RAB9B** | 75.6553676759607 | 93.9072806124286 | 54.9070382028612 | 28.1099478141363 | 24.5963873104517 | 28.3844193909769 | 74.8232288304168 | 27.0302515051883 | 1.470 | 1.30895E-04 |
| ENSG00000260328 | **AC104024.2** | 98.1475040120571 | 76.1143221806 | 108.778094552838 | 42.1649217212044 | 37.3865087118865 | 40.5491705585385 | 94.3466402484985 | 40.0335336638765 | 1.235 | 1.34370E-04 |
| ENSG00000226650 | **KIF4B** | 73.6106280090428 | 64.2523498927143 | 62.1589111730505 | 23.4249565117802 | 26.5640982952878 | 22.3020438071962 | 66.6739630249359 | 24.0970328714214 | 1.468 | 1.37005E-04 |
| ENSG00000141505 | **ASGR1** | 169.713392354182 | 157.171132814486 | 134.677640874943 | 74.0228625772256 | 86.5792833327899 | 83.1257996450039 | 153.85405534787 | 81.2426485183398 | 0.922 | 1.43076E-04 |
| ENSG00000251141 | **MRPS30-DT** | 253.547718697814 | 321.261749463572 | 258.995463221044 | 186.462653833771 | 174.142422157998 | 142.935826218848 | 277.934977127476 | 167.846967403539 | 0.727 | 1.67055E-04 |
| ENSG00000276953 | **TRBV12-4** | 11.2460681680482 | 12.8504699785429 | 14.5037459403784 | 0 | 0 | 0 | 12.8667613623232 | 0 | 6.161 | 1.92448E-04 |
| ENSG00000244752 | **CRYBB2** | 58.2750805071589 | 80.0683129432286 | 65.266856731703 | 28.1099478141363 | 20.6609653407794 | 25.3432315990865 | 67.8700833940302 | 24.7047149180008 | 1.457 | 1.95724E-04 |
| ENSG00000187837 | **HIST1H1C** | 389.522906547852 | 400.341564716143 | 470.335761209415 | 312.020420736913 | 228.254474240992 | 281.816735381842 | 420.066744157803 | 274.030543453249 | 0.615 | 2.01569E-04 |
| ENSG00000165030 | **NFIL3** | 262.749047198945 | 312.365270247657 | 274.535191014306 | 181.777662531415 | 191.851821021523 | 167.265328553971 | 283.216502820303 | 180.298270702303 | 0.652 | 2.45724E-04 |
| ENSG00000125735 | **TNFSF14** | 19.4250268357196 | 11.8619722878857 | 7.25187297018922 | 0 | 0 | 0 | 12.8462906979315 | 0 | 6.159 | 2.47852E-04 |
| ENSG00000284753 | **EEF1AKMT4** | 258.659567865109 | 246.135924973629 | 268.319299897001 | 170.53368340576 | 166.271578218653 | 152.059389594519 | 257.704930911913 | 162.954883739644 | 0.660 | 2.49952E-04 |
| ENSG00000246528 | **AC079089.1** | 30.6710950037678 | 22.7354468851143 | 21.7556189105677 | 5.62198956282726 | 1.96771098483613 | 1.01372926396346 | 25.0540535998166 | 2.86780993720895 | 3.107 | 2.51616E-04 |
| ENSG00000108244 | **KRT23** | 18.4026570022607 | 23.7239445757714 | 35.2233829980619 | 1.87399652094242 | 0.983855492418067 | 6.08237558378077 | 25.7833281920314 | 2.98007586571375 | 3.127 | 2.62867E-04 |
| ENSG00000151967 | **SCHIP1** | 90.9909151778446 | 86.9877967778286 | 103.598185288417 | 44.0389182421469 | 45.2573526512311 | 37.5079827666481 | 93.8589657480302 | 42.268084553342 | 1.149 | 2.63249E-04 |
| ENSG00000108448 | **TRIM16L** | 511.184916729464 | 456.6859330836 | 787.346208191973 | 328.886389425395 | 369.929665149193 | 387.244578834042 | 585.072352668346 | 362.02021113621 | 0.692 | 2.68756E-04 |
| ENSG00000168685 | **IL7R** | 24.5368760030143 | 22.7354468851143 | 29.0074918807569 | 4.68499130235605 | 2.9515664772542 | 3.04118779189039 | 25.4266049229618 | 3.55924852383355 | 2.830 | 2.83849E-04 |
| ENSG00000225339 | **AL354740.1** | 311.822799204973 | 312.365270247657 | 247.599662839318 | 194.895638178012 | 193.819532006359 | 149.018201802629 | 290.595910763983 | 179.244457329 | 0.696 | 2.94208E-04 |
| ENSG00000270607 | **AC009549.1** | 301.599100870384 | 255.032404189543 | 233.095916898939 | 188.336650354713 | 143.642901893038 | 144.963284746775 | 263.242473986289 | 158.980945664842 | 0.726 | 3.15190E-04 |
| ENSG00000183760 | **ACP7** | 22.4921363360964 | 35.5859168636572 | 12.4317822346101 | 0 | 1.96771098483613 | 4.05491705585385 | 23.5032784781212 | 2.00754268022999 | 3.577 | 3.31317E-04 |
| ENSG00000185634 | **SHC4** | 35.7829441710625 | 35.5859168636572 | 42.4752559682511 | 7.49598608376968 | 8.8546994317626 | 12.1647511675615 | 37.9480390009903 | 9.50514556103128 | 2.001 | 3.68787E-04 |
| ENSG00000232533 | **AC093673.1** | 309.778059538055 | 245.147427282971 | 349.125884421967 | 202.391624261781 | 175.126277650416 | 188.553643097204 | 301.350457080998 | 188.690515003134 | 0.674 | 4.10257E-04 |
| ENSG00000234956 | **LINC02539** | 37.8276838379803 | 30.6434284103714 | 22.7916007634518 | 2.81099478141363 | 5.9031329545084 | 8.10983411170769 | 30.4209043372679 | 5.60798728254324 | 2.450 | 4.18517E-04 |
| ENSG00000172183 | **ISG20** | 377.254468546345 | 338.066210204743 | 336.694102187357 | 282.973474662305 | 169.223144695908 | 187.53991383324 | 350.671593646148 | 213.245511063818 | 0.716 | 4.33193E-04 |
| ENSG00000180611 | **MB21D2** | 210.608185692539 | 259.974892642829 | 249.671626545086 | 145.234730373038 | 145.610612877874 | 158.1417651783 | 240.084901626818 | 149.662369476404 | 0.683 | 4.38177E-04 |
| ENSG00000247993 | **FOXD1-AS1** | 65.4316693413714 | 84.0223037058572 | 70.4467659961239 | 32.7949391164923 | 23.6125318180336 | 32.4393364468308 | 73.3002463477841 | 29.6156024604522 | 1.308 | 4.51256E-04 |
| ENSG00000198959 | **TGM2** | 132.908078349661 | 74.1373267992857 | 108.778094552838 | 50.5979060654453 | 38.3703642043046 | 50.6864631981731 | 105.274499900595 | 46.551577822641 | 1.176 | 4.52258E-04 |
| ENSG00000188277 | **C15orf62** | 196.295008024114 | 319.284754082257 | 190.620660930688 | 114.313787777488 | 147.57832386271 | 125.702428731469 | 235.400141012353 | 129.198180123889 | 0.867 | 4.68514E-04 |
| ENSG00000069667 | **RORA** | 50.0961218394875 | 46.4593914608857 | 43.5112378211353 | 14.9919721675394 | 13.7739768938529 | 15.2059389594519 | 46.6889170405028 | 14.6572960069481 | 1.672 | 4.69281E-04 |
| novel.682 | **-** | 135.975187850037 | 153.217142051857 | 7.25187297018922 | 3.74799304188484 | 1.96771098483613 | 4.05491705585385 | 98.8147342906946 | 3.25687369419161 | 4.923 | 5.44740E-04 |
| ENSG00000229656 | **ITGB1-DT** | 82.8119565101732 | 86.9877967778286 | 112.922021964375 | 50.5979060654453 | 34.4349422346324 | 42.5766290864654 | 94.2405917507923 | 42.536492462181 | 1.145 | 5.69964E-04 |
| ENSG00000120051 | **CFAP58** | 54.1856011733232 | 42.5054006982572 | 66.3028385845872 | 11.2439791256545 | 22.6286763256155 | 19.2608560153058 | 54.3312801520558 | 17.7111704888586 | 1.620 | 5.95505E-04 |
| ENSG00000132970 | **WASF3** | 11.2460681680482 | 8.89647921591429 | 12.4317822346101 | 0 | 0 | 0 | 10.8581098728575 | 0 | 5.915 | 6.19154E-04 |
| ENSG00000237914 | **SIRPG-AS1** | 9.20132850113035 | 10.8734745972286 | 12.4317822346101 | 0 | 0 | 0 | 10.835528444323 | 0 | 5.913 | 6.23217E-04 |
| ENSG00000136999 | **NOV** | 323.068867373021 | 302.480293341086 | 158.505223491279 | 158.352706019634 | 146.594468370292 | 105.4278434522 | 261.351461401795 | 136.791672614042 | 0.933 | 6.41973E-04 |
| ENSG00000114796 | **KLHL24** | 1237.0674984853 | 1813.89326235586 | 1231.78242307928 | 725.236653604716 | 877.599099236916 | 769.420511348268 | 1427.58106130681 | 790.7520880633 | 0.852 | 6.82753E-04 |
| ENSG00000180596 | **HIST1H2BC** | 63.3869296744535 | 66.2293452740286 | 72.5187297018922 | 28.1099478141363 | 29.515664772542 | 25.3432315990865 | 67.3783348834581 | 27.656281395255 | 1.284 | 7.25098E-04 |
| ENSG00000196542 | **SPTSSB** | 119.617270514695 | 168.044607411714 | 125.353804198985 | 74.9598608376968 | 68.8698844692647 | 76.0296947972596 | 137.671894041798 | 73.2864800347404 | 0.911 | 7.26212E-04 |
| ENSG00000180447 | **GAS1** | 30.6710950037678 | 29.6549307197143 | 15.5397277932626 | 6.55898782329847 | 2.9515664772542 | 1.01372926396346 | 25.2885845055816 | 3.50809452150538 | 2.833 | 7.49120E-04 |
| ENSG00000170786 | **SDR16C5** | 110.415942013564 | 96.8727736844 | 59.0509656143979 | 34.6689356374348 | 39.3542196967227 | 40.5491705585385 | 88.7798937707874 | 38.1907752975653 | 1.219 | 8.32736E-04 |
| ENSG00000123096 | **SSPN** | 22.4921363360964 | 26.6894376477429 | 24.8635644692202 | 2.81099478141363 | 6.88698844692647 | 2.02745852792692 | 24.6817128176865 | 3.90848058542234 | 2.659 | 8.41864E-04 |
| ENSG00000179862 | **CITED4** | 927.289438947248 | 575.305655962457 | 661.992403992987 | 555.639968459427 | 437.81569412604 | 441.985959088069 | 721.529166300898 | 478.480540557846 | 0.592 | 8.60388E-04 |
| ENSG00000172020 | **GAP43** | 24.5368760030143 | 18.7814561224857 | 19.6836552047993 | 2.81099478141363 | 3.93542196967227 | 1.01372926396346 | 21.0006624434331 | 2.58671533834979 | 3.014 | 9.40189E-04 |
| ENSG00000183935 | **HTR7P1** | 177.892351021854 | 195.722542750114 | 165.757096461468 | 83.3928451819377 | 123.965792044676 | 105.4278434522 | 179.790663411145 | 104.262160226271 | 0.788 | 9.60202E-04 |
| ENSG00000138411 | **HECW2** | 58.2750805071589 | 57.3328660581143 | 40.4032922624828 | 17.802966948953 | 22.6286763256155 | 12.1647511675615 | 52.0037462759187 | 17.53213148071 | 1.567 | 9.62270E-04 |
| ENSG00000223669 | **AL357033.2** | 84.856696177091 | 84.0223037058572 | 77.6986389663131 | 44.9759165026181 | 34.4349422346324 | 33.4530657107942 | 82.1925462830871 | 37.6213081493482 | 1.125 | 1.06199E-03 |
| ENSG00000102796 | **DHRS12** | 88.9461755109268 | 111.700239044257 | 78.7346208191972 | 41.2279234607332 | 46.2412081436492 | 45.6178168783558 | 93.1270117914604 | 44.3623161609127 | 1.072 | 1.06299E-03 |
| ENSG00000262583 | **AC009163.5** | 35.7829441710625 | 27.6779353384 | 39.3673104095986 | 10.3069808651833 | 6.88698844692647 | 9.12356337567116 | 34.2760633063537 | 8.77251089592698 | 1.963 | 1.07632E-03 |
| ENSG00000231683 | **AL033397.1** | 16.3579173353429 | 7.90798152525714 | 75.6266752605447 | 3.74799304188484 | 3.93542196967227 | 5.06864631981731 | 33.2975247070482 | 4.25068711045814 | 2.970 | 1.08786E-03 |
| novel.214 | **-** | 180.95946052223 | 179.9065796996 | 160.577187197047 | 93.699826047121 | 105.272537688733 | 113.537677563908 | 173.814409139626 | 104.170013766587 | 0.740 | 1.12762E-03 |
| ENSG00000136842 | **TMOD1** | 173.802871688018 | 171.998598174343 | 142.965495698016 | 90.8888312657073 | 106.256393181151 | 88.1944459648212 | 162.922321853459 | 95.1132234705599 | 0.777 | 1.18752E-03 |
| ENSG00000173727 | **AP000769.1** | 309.778059538055 | 333.123721751457 | 227.916007634518 | 194.895638178012 | 190.867965529105 | 170.306516345862 | 290.27259630801 | 185.356706684326 | 0.647 | 1.25187E-03 |
| novel.450 | **-** | 325.113607039939 | 284.687334909257 | 352.233829980619 | 235.186563378274 | 171.190855680744 | 225.047896599889 | 320.678257309939 | 210.475105219635 | 0.607 | 1.29400E-03 |
| ENSG00000259985 | **AC017100.1** | 168.691022520723 | 137.401179001343 | 148.145404962437 | 97.4478190890058 | 77.7245839010273 | 85.1532581729308 | 151.412535494834 | 86.7752203876546 | 0.802 | 1.32527E-03 |
| ENSG00000112139 | **MDGA1** | 219.80951419367 | 258.986394952172 | 237.239844310476 | 138.675742549739 | 173.15856666558 | 148.004472538665 | 238.678584485439 | 153.279593917995 | 0.640 | 1.34065E-03 |
| ENSG00000128683 | **GAD1** | 77.7001073428786 | 70.1833360366572 | 67.3388204374713 | 31.8579408560211 | 25.5802428028697 | 36.4942535026846 | 71.740754605669 | 31.3108123871918 | 1.197 | 1.34771E-03 |
| ENSG00000275322 | **AC103746.1** | 183.004200189148 | 136.412681310686 | 133.641659022058 | 70.2748695353407 | 96.4178382569706 | 84.1395289089673 | 151.019513507297 | 83.6107455670929 | 0.854 | 1.36607E-03 |
| ENSG00000088882 | **CPXM1** | 22.4921363360964 | 14.8274653598571 | 13.4677640874943 | 0.93699826047121 | 1.96771098483613 | 1.01372926396346 | 16.9291219278159 | 1.30614616975694 | 3.699 | 1.53603E-03 |
| ENSG00000131737 | **KRT34** | 24.5368760030143 | 9.88497690657143 | 17.611691499031 | 0.93699826047121 | 0.983855492418067 | 2.02745852792692 | 17.3445148028722 | 1.3161040936054 | 3.730 | 1.53648E-03 |
| ENSG00000231770 | **TMEM44-AS1** | 167.668652687264 | 160.136625886457 | 187.512715372036 | 109.628796475132 | 99.3694047342248 | 104.414114188237 | 171.772664648586 | 104.470771799198 | 0.717 | 1.66287E-03 |
| ENSG00000235495 | **AC007422.2** | 22.4921363360964 | 44.4823960795714 | 19.6836552047993 | 8.43298434424089 | 4.91927746209034 | 4.05491705585385 | 28.8860625401557 | 5.80239295406169 | 2.310 | 1.70648E-03 |
| ENSG00000256943 | **AC148477.3** | 11.2460681680482 | 34.597419173 | 21.7556189105677 | 2.81099478141363 | 3.93542196967227 | 3.04118779189039 | 22.5330354172053 | 3.26253484765876 | 2.791 | 1.73713E-03 |
| ENSG00000166780 | **C16orf45** | 24.5368760030143 | 50.4133822235143 | 17.611691499031 | 4.68499130235605 | 9.83855492418067 | 4.05491705585385 | 30.8539832418532 | 6.19282109413019 | 2.319 | 1.77995E-03 |
| ENSG00000148677 | **ANKRD1** | 53.1632313398643 | 20.7584515038 | 70.4467659961239 | 19.6769634698954 | 10.8224104165987 | 8.10983411170769 | 48.1228162799294 | 12.8697359994006 | 1.895 | 1.81209E-03 |
| ENSG00000173040 | **EVC2** | 42.939533005275 | 52.3903776048286 | 43.5112378211353 | 18.7399652094242 | 15.7416878786891 | 14.1922096954885 | 46.280382810413 | 16.2246209278672 | 1.510 | 1.82475E-03 |
| ENSG00000255916 | **AC148477.1** | 18.4026570022607 | 9.88497690657143 | 20.7196370576835 | 1.87399652094242 | 0 | 1.01372926396346 | 16.3357569888385 | 0.962575261635294 | 4.067 | 1.87640E-03 |
| ENSG00000226835 | **AC097059.1** | 68.4987788417482 | 61.2868568207429 | 64.2308748788188 | 23.4249565117802 | 29.515664772542 | 30.4118779189039 | 64.6721701804366 | 27.7841664010754 | 1.221 | 1.88679E-03 |
| ENSG00000228705 | **LINC00659** | 280.129334367746 | 256.0209018802 | 308.722592159484 | 195.832636438483 | 157.416878786891 | 205.787040584583 | 281.62427613581 | 186.345518603319 | 0.595 | 1.89300E-03 |
| ENSG00000141837 | **CACNA1A** | 17.3802871688018 | 24.7124422664286 | 11.3958003817259 | 1.87399652094242 | 0 | 3.04118779189039 | 17.8295099389854 | 1.63839477094427 | 3.451 | 1.98590E-03 |
| ENSG00000049249 | **TNFRSF9** | 64.4092995079125 | 42.5054006982572 | 58.0149837615138 | 14.0549739070681 | 17.7093988635252 | 28.3844193909769 | 54.9765613225611 | 20.0495973871901 | 1.460 | 1.98590E-03 |
| ENSG00000091986 | **CCDC80** | 18.4026570022607 | 28.6664330290571 | 16.5757096461468 | 4.68499130235605 | 1.96771098483613 | 3.04118779189039 | 21.2149332258216 | 3.23129669302752 | 2.709 | 2.10327E-03 |
| ENSG00000165621 | **OXGR1** | 27.6039855033911 | 1.97699538131429 | 16.5757096461468 | 0 | 0 | 1.01372926396346 | 15.385563510284 | 0.337909754654487 | 5.455 | 2.10552E-03 |
| ENSG00000232233 | **LINC02043** | 80.7672168432553 | 54.3673729861429 | 61.1229293201663 | 30.9209425955499 | 31.4833757573781 | 13.178480431525 | 65.4191730498548 | 25.1942662614844 | 1.372 | 2.17733E-03 |
| ENSG00000224093 | **AL109613.1** | 60.3198201740768 | 37.5629122449714 | 32.1154374394094 | 16.8659686884818 | 13.7739768938529 | 10.1372926396346 | 43.3327232861525 | 13.5924127406564 | 1.669 | 2.24428E-03 |
| ENSG00000099937 | **SERPIND1** | 21.4697665026375 | 13.8389676692 | 14.5037459403784 | 2.81099478141363 | 0.983855492418067 | 1.01372926396346 | 16.6041600374053 | 1.60285984593172 | 3.357 | 2.65441E-03 |
| ENSG00000005243 | **COPZ2** | 96.1027643451392 | 111.700239044257 | 88.0584574951548 | 59.9678886701574 | 43.289641666395 | 48.6590046702462 | 98.6204869615171 | 50.6388450022662 | 0.961 | 2.68251E-03 |
| ENSG00000237013 | **LINC01812** | 36.8053140045214 | 14.8274653598571 | 42.4752559682511 | 5.62198956282726 | 3.93542196967227 | 11.1510219035981 | 31.3693451108766 | 6.90281114536587 | 2.188 | 2.75140E-03 |
| ENSG00000240602 | **AADACP1** | 1996.68828474529 | 1489.66601982031 | 2551.62330365372 | 1148.7598673377 | 1121.5952613566 | 1186.06323883725 | 2012.65920273977 | 1152.13945584385 | 0.805 | 2.89482E-03 |
| ENSG00000057704 | **TMCC3** | 21.4697665026375 | 10.8734745972286 | 15.5397277932626 | 0 | 1.96771098483613 | 2.02745852792692 | 15.9609896310429 | 1.33172317092102 | 3.605 | 2.97249E-03 |
| ENSG00000115758 | **ODC1** | 27066.2189709917 | 18691.5028326359 | 27495.9943573989 | 16345.9346539203 | 14260.9853625999 | 15951.0299684651 | 24417.9053870088 | 15519.3166616617 | 0.654 | 3.01352E-03 |
| novel.827 | **-** | 35.7829441710625 | 41.5169030076 | 6.21589111730505 | 2.81099478141363 | 6.88698844692647 | 4.05491705585385 | 27.8385794319892 | 4.58430009473132 | 2.607 | 3.12628E-03 |
| ENSG00000255857 | **PXN-AS1** | 175.847611354936 | 181.883575080914 | 164.721114608584 | 108.69179821466 | 97.4016937493886 | 120.633782411652 | 174.150767014811 | 108.909091458567 | 0.678 | 3.17246E-03 |
| ENSG00000172137 | **CALB2** | 19.4250268357196 | 3.95399076262857 | 4.1439274115367 | 0 | 0 | 0 | 9.17431500329497 | 0 | 5.672 | 3.19421E-03 |
| ENSG00000138193 | **PLCE1** | 77.7001073428786 | 100.826764447029 | 95.310330465344 | 41.2279234607332 | 39.3542196967227 | 56.7688387819539 | 91.2790674184171 | 45.7836606464699 | 0.998 | 3.26020E-03 |
| ENSG00000235385 | **LINC02154** | 13.2908078349661 | 19.7699538131429 | 11.3958003817259 | 1.87399652094242 | 0.983855492418067 | 0 | 14.8188540099449 | 0.952617337786829 | 3.933 | 3.37217E-03 |
| ENSG00000103145 | **HCFC1R1** | 321.024127706103 | 338.066210204743 | 328.406247364283 | 247.367540764399 | 160.368445264145 | 250.391128198975 | 329.165528425043 | 219.375704742506 | 0.585 | 3.63686E-03 |
| ENSG00000198431 | **TXNRD1** | 59744.2259578394 | 46495.96587544 | 73152.7505958573 | 31244.2069954125 | 34149.6241418311 | 40058.5255947802 | 59797.6474763789 | 35150.7855773413 | 0.767 | 3.70239E-03 |
| ENSG00000258623 | **AL121820.1** | 46.0066425056518 | 42.5054006982572 | 42.4752559682511 | 14.0549739070681 | 14.757832386271 | 19.2608560153058 | 43.6624330573867 | 16.0245541028816 | 1.449 | 3.74527E-03 |
| ENSG00000246228 | **CASC8** | 173.802871688018 | 123.562211332143 | 170.937005725889 | 104.006806912304 | 92.4824162872983 | 77.0434240612231 | 156.100696248683 | 91.1775490869419 | 0.773 | 3.77036E-03 |
| ENSG00000279673 | **AC092919.2** | 92.0132850113035 | 83.0338060152 | 73.5547115547764 | 52.4719025863877 | 38.3703642043046 | 20.2745852792692 | 82.8672675270933 | 37.0389506899872 | 1.157 | 3.85269E-03 |
| ENSG00000237596 | **AL138828.1** | 93.0356548447625 | 88.9647921591429 | 49.7271289384404 | 34.6689356374348 | 40.3380751891408 | 28.3844193909769 | 77.2425253141152 | 34.4638100725175 | 1.164 | 3.87322E-03 |
| novel.210 | **-** | 9.20132850113035 | 3.95399076262857 | 11.3958003817259 | 0 | 0 | 0 | 8.18370654849495 | 0 | 5.506 | 4.08513E-03 |
| ENSG00000159840 | **ZYX** | 355.784702043707 | 207.584515038 | 385.385249272913 | 217.383596429321 | 186.932543559433 | 185.512455305314 | 316.251488784873 | 196.609531764689 | 0.684 | 4.12456E-03 |
| ENSG00000151773 | **CCDC122** | 229.0108426948 | 214.5039988726 | 274.535191014306 | 177.092671229059 | 161.352300756563 | 130.771075051287 | 239.350010860569 | 156.405349012303 | 0.612 | 4.48782E-03 |
| ENSG00000236530 | **KPNA2P1** | 82.8119565101732 | 95.8842759937429 | 103.598185288417 | 57.1568938887438 | 53.1281965905756 | 25.3432315990865 | 94.0981392641112 | 45.209440692802 | 1.054 | 4.60381E-03 |
| ENSG00000241399 | **CD302** | 177.892351021854 | 203.630524275371 | 207.196370576835 | 128.368761684556 | 132.820491476439 | 125.702428731469 | 196.239748624687 | 128.963893964155 | 0.606 | 4.94615E-03 |
| ENSG00000188958 | **UTS2B** | 52.1408615064053 | 45.4708937702286 | 47.655165232672 | 24.3619547722515 | 17.7093988635252 | 15.2059389594519 | 48.4223068364353 | 19.0924308650762 | 1.339 | 5.03576E-03 |
| ENSG00000150630 | **VEGFC** | 11.2460681680482 | 13.8389676692 | 8.2878548230734 | 0 | 0 | 1.01372926396346 | 11.1242968867739 | 0.337909754654487 | 4.990 | 5.09729E-03 |
| novel.836 | **-** | 70.5435185086661 | 41.5169030076 | 43.5112378211353 | 17.802966948953 | 6.88698844692647 | 28.3844193909769 | 51.8572197791338 | 17.6914582622855 | 1.554 | 5.26919E-03 |
| ENSG00000240429 | **LRRFIP1P1** | 51.1184916729464 | 67.2178429646857 | 47.655165232672 | 17.802966948953 | 28.5318092801239 | 23.3157730711596 | 55.3304999567681 | 23.2168497667455 | 1.256 | 5.52967E-03 |
| ENSG00000141337 | **ARSG** | 42.939533005275 | 48.4363868422 | 40.4032922624828 | 11.2439791256545 | 21.6448208331975 | 16.2196682234154 | 43.9264040366526 | 16.3694893940891 | 1.428 | 5.57877E-03 |
| ENSG00000105865 | **DUS4L** | 123.70674984853 | 94.8957783030857 | 32.1154374394094 | 40.290925200262 | 30.4995202649601 | 26.35696086305 | 83.5726551970085 | 32.3824687760907 | 1.366 | 5.57877E-03 |
| ENSG00000270959 | **LPP-AS2** | 117.572530847777 | 125.539206713457 | 123.281840493217 | 66.5268764934559 | 62.9667515147563 | 84.1395289089673 | 122.131192684817 | 71.2110523057265 | 0.780 | 5.66613E-03 |
| ENSG00000132669 | **RIN2** | 69.5211486752071 | 56.3443683674572 | 59.0509656143979 | 36.5429321583772 | 23.6125318180336 | 21.2883145432327 | 61.6388275523541 | 27.1479261732145 | 1.178 | 5.88304E-03 |
| ENSG00000283959 | **AP002851.1** | 31.6934648372268 | 50.4133822235143 | 44.5472196740195 | 16.8659686884818 | 14.757832386271 | 15.2059389594519 | 42.2180222449202 | 15.6099133447349 | 1.435 | 6.19466E-03 |
| ENSG00000012124 | **CD22** | 10.2236983345893 | 17.7929584318286 | 12.4317822346101 | 0 | 1.96771098483613 | 1.01372926396346 | 13.4828130003427 | 0.993813416266532 | 3.781 | 6.25465E-03 |
| ENSG00000236908 | **AC005865.1** | 128.818599015825 | 173.975593555657 | 158.505223491279 | 103.069808651833 | 82.6438613631176 | 96.3042800765289 | 153.76647202092 | 94.0059833638265 | 0.710 | 6.29055E-03 |
| ENSG00000129946 | **SHC2** | 65.4316693413714 | 94.8957783030857 | 45.5832015269037 | 27.1729495536651 | 29.515664772542 | 34.4667949747577 | 68.6368830571203 | 30.3851364336549 | 1.179 | 6.46412E-03 |
| ENSG00000183323 | **CCDC125** | 169.713392354182 | 137.401179001343 | 58.0149837615138 | 59.9678886701574 | 60.0151850375021 | 54.7413802540269 | 121.70985170568 | 58.2414846538955 | 1.064 | 6.59802E-03 |
| ENSG00000158406 | **HIST1H4H** | 134.952818016579 | 129.493197476086 | 154.361296079742 | 71.2118677958119 | 99.3694047342248 | 81.098341117077 | 139.602437190802 | 83.8932045490379 | 0.735 | 6.63704E-03 |
| ENSG00000114698 | **PLSCR4** | 79.7448470097964 | 90.9417875404572 | 72.5187297018922 | 34.6689356374348 | 51.1604856057395 | 37.5079827666481 | 81.0684547507153 | 41.1124680032741 | 0.981 | 6.67715E-03 |
| ENSG00000135625 | **EGR4** | 129.840968849284 | 106.757750590971 | 147.109423109553 | 69.3378712748695 | 79.6922948858634 | 77.0434240612231 | 127.902714183269 | 75.3578634073187 | 0.763 | 6.79541E-03 |
| ENSG00000064042 | **LIMCH1** | 179.937090688771 | 250.089915736257 | 193.728606489341 | 134.927749507854 | 142.65904640062 | 129.757345787323 | 207.918537638123 | 135.781380565266 | 0.615 | 6.90077E-03 |
| ENSG00000065413 | **ANKRD44** | 120.639640348154 | 163.102118958429 | 103.598185288417 | 68.4008730143983 | 82.6438613631176 | 70.9610484774423 | 129.113314865 | 74.0019276183194 | 0.804 | 6.92072E-03 |
| ENSG00000272468 | **AL021807.1** | 51.1184916729464 | 54.3673729861429 | 60.0869474672821 | 30.9209425955499 | 24.5963873104517 | 10.1372926396346 | 55.1909373754571 | 21.8848741818787 | 1.328 | 7.00401E-03 |
| ENSG00000196787 | **HIST1H2AG** | 215.720034859834 | 361.790154780514 | 309.758574012368 | 195.832636438483 | 212.512786362303 | 173.347704137752 | 295.756254550905 | 193.897708979512 | 0.609 | 7.04308E-03 |
| ENSG00000248476 | **BACH1-IT1** | 50.0961218394875 | 38.5514099356286 | 47.655165232672 | 14.0549739070681 | 17.7093988635252 | 22.3020438071962 | 45.4342323359294 | 18.0221388592632 | 1.337 | 7.46777E-03 |
| ENSG00000277449 | **CEBPB-AS1** | 141.087037017332 | 172.987095865 | 99.4542578768807 | 90.8888312657073 | 60.9990405299202 | 81.098341117077 | 137.842796919738 | 77.6620709709015 | 0.828 | 7.97825E-03 |
| ENSG00000275896 | **PRSS2** | 26.5816156699321 | 41.5169030076 | 30.0434737336411 | 13.1179756465969 | 2.9515664772542 | 12.1647511675615 | 32.7139974703911 | 9.41143109713756 | 1.796 | 8.03323E-03 |
| ENSG00000069702 | **TGFBR3** | 153.355475018839 | 204.619021966029 | 140.893531992248 | 78.7078538795816 | 101.337115719061 | 121.647511675615 | 166.289342992372 | 100.564160424753 | 0.729 | 8.59492E-03 |
| ENSG00000259023 | **LINC00524** | 54.1856011733232 | 63.2638522020572 | 68.3748022903555 | 25.2989530327227 | 26.5640982952878 | 35.4805242387212 | 61.9414185552453 | 29.1145251889105 | 1.092 | 8.63026E-03 |
| ENSG00000139364 | **TMEM132B** | 18.4026570022607 | 26.6894376477429 | 22.7916007634518 | 5.62198956282726 | 5.9031329545084 | 5.06864631981731 | 22.6278984711518 | 5.53125627905099 | 2.032 | 8.79216E-03 |
| ENSG00000182742 | **HOXB4** | 164.601543186887 | 167.056109721057 | 166.793078314352 | 103.069808651833 | 106.256393181151 | 116.578865355798 | 166.150243740766 | 108.635022396261 | 0.614 | 8.81950E-03 |
| ENSG00000136155 | **SCEL** | 22.4921363360964 | 9.88497690657143 | 12.4317822346101 | 3.74799304188484 | 0 | 1.01372926396346 | 14.936298492426 | 1.5872407686161 | 3.210 | 9.09771E-03 |
| ENSG00000109339 | **MAPK10** | 110.415942013564 | 88.9647921591429 | 77.6986389663131 | 52.4719025863877 | 50.1766301133214 | 48.6590046702462 | 92.3597910463401 | 50.4358457899851 | 0.872 | 9.27151E-03 |
| novel.71 | **-** | 118.594900681236 | 147.286155907914 | 151.253350521089 | 101.195812130891 | 76.7407284086092 | 74.0022362693327 | 139.04480237008 | 83.9795922696109 | 0.725 | 9.31857E-03 |
| ENSG00000185339 | **TCN2** | 208.563446025621 | 211.538505800629 | 141.929513845132 | 142.423735591624 | 112.15952613566 | 86.1669874368943 | 187.343821890461 | 113.583416388059 | 0.720 | 9.40604E-03 |
| ENSG00000198892 | **SHISA4** | 144.154146517709 | 132.458690548057 | 137.785586433595 | 89.0148347447649 | 79.6922948858634 | 91.2356337567116 | 138.13280783312 | 86.64758779578 | 0.673 | 9.63794E-03 |
| ENSG00000285444 | **AL162377.3** | 31.6934648372268 | 32.6204237916857 | 15.5397277932626 | 5.62198956282726 | 6.88698844692647 | 9.12356337567116 | 26.617872140725 | 7.21084712847496 | 1.890 | 9.99497E-03 |
| ENSG00000276180 | **HIST1H4I** | 95.0803945116803 | 103.792257519 | 84.9505119365023 | 58.093892149215 | 55.0959075754118 | 45.6178168783558 | 94.6077213223942 | 52.9358722009942 | 0.837 | 1.03038E-02 |
| novel.632 | **-** | 132.908078349661 | 100.826764447029 | 131.56969531629 | 76.8338573586392 | 79.6922948858634 | 57.7825680459173 | 121.768179370993 | 71.4362400968067 | 0.767 | 1.04486E-02 |
| ENSG00000124116 | **WFDC3** | 17.3802871688018 | 20.7584515038 | 18.6476733519151 | 4.68499130235605 | 4.91927746209034 | 2.02745852792692 | 18.9288040081723 | 3.8772424307911 | 2.281 | 1.05363E-02 |
| ENSG00000235448 | **LURAP1L-AS1** | 47.0290123391107 | 25.7009399570857 | 34.1874011451778 | 14.9919721675394 | 10.8224104165987 | 11.1510219035981 | 35.6391178137914 | 12.3218014959121 | 1.529 | 1.06113E-02 |
| ENSG00000223750 | **SIRPB3P** | 4.08947933383571 | 9.88497690657143 | 6.21589111730505 | 0 | 0 | 0 | 6.73011578590406 | 0 | 5.227 | 1.06474E-02 |
| ENSG00000236056 | **GAPDHP14** | 4.08947933383571 | 4.94248845328572 | 11.3958003817259 | 0 | 0 | 0 | 6.80925605628245 | 0 | 5.241 | 1.07219E-02 |
| ENSG00000150556 | **LYPD6B** | 17.3802871688018 | 23.7239445757714 | 30.0434737336411 | 5.62198956282726 | 6.88698844692647 | 6.08237558378077 | 23.7159018260714 | 6.1971178645115 | 1.937 | 1.07832E-02 |
| ENSG00000253111 | **AC091114.1** | 16.3579173353429 | 4.94248845328572 | 22.7916007634518 | 2.81099478141363 | 0.983855492418067 | 1.01372926396346 | 14.6973355173601 | 1.60285984593172 | 3.180 | 1.09882E-02 |
| ENSG00000147573 | **TRIM55** | 44.9842726721928 | 52.3903776048286 | 47.655165232672 | 18.7399652094242 | 21.6448208331975 | 23.3157730711596 | 48.3432718365645 | 21.2335197045938 | 1.189 | 1.15041E-02 |
| ENSG00000245105 | **A2M-AS1** | 57.2527106737 | 46.4593914608857 | 41.439274115367 | 31.8579408560211 | 9.83855492418067 | 12.1647511675615 | 48.3837920833176 | 17.9537489825878 | 1.422 | 1.17367E-02 |
| ENSG00000280206 | **AC026401.3** | 94.0580246782214 | 94.8957783030857 | 82.8785482307339 | 44.9759165026181 | 55.0959075754118 | 52.7139217261 | 90.610783737347 | 50.9285819347099 | 0.833 | 1.17663E-02 |
| ENSG00000119699 | **TGFB3** | 208.563446025621 | 182.872072771571 | 151.253350521089 | 118.998779079844 | 124.949647537095 | 112.523948299944 | 180.896289772761 | 118.824124972294 | 0.606 | 1.20945E-02 |
| ENSG00000273983 | **HIST1H3G** | 108.371202346646 | 138.389676692 | 101.526221582649 | 32.7949391164923 | 89.5308498100441 | 57.7825680459173 | 116.095700207099 | 60.0361189908179 | 0.954 | 1.22162E-02 |
| ENSG00000123685 | **BATF3** | 78.7224771763375 | 69.194838346 | 65.266856731703 | 40.290925200262 | 38.3703642043046 | 31.4256071828673 | 71.0613907513468 | 36.6956321958113 | 0.952 | 1.25504E-02 |
| ENSG00000058335 | **RASGRF1** | 4.08947933383571 | 14.8274653598571 | 2.07196370576835 | 0 | 0 | 0 | 6.9963027998204 | 0 | 5.285 | 1.25790E-02 |
| ENSG00000182798 | **MAGEB17** | 23.5145061695554 | 15.8159630505143 | 11.3958003817259 | 0.93699826047121 | 3.93542196967227 | 4.05491705585385 | 16.9087565339319 | 2.97577909533244 | 2.519 | 1.34126E-02 |
| ENSG00000138166 | **DUSP5** | 20.4473966691786 | 8.89647921591429 | 6.21589111730505 | 0 | 1.96771098483613 | 0 | 11.853255667466 | 0.655903661612045 | 4.183 | 1.35813E-02 |
| ENSG00000139174 | **PRICKLE1** | 120.639640348154 | 92.9187829217715 | 141.929513845132 | 74.9598608376968 | 56.0797630678298 | 76.0296947972596 | 118.495979038352 | 69.0231062342621 | 0.779 | 1.35906E-02 |
| ENSG00000256915 | **AC090023.2** | 120.639640348154 | 100.826764447029 | 164.721114608584 | 53.408900846859 | 66.9021734844286 | 97.3180093404923 | 128.729173134589 | 72.5430278905933 | 0.829 | 1.42632E-02 |
| ENSG00000163395 | **IGFN1** | 27.6039855033911 | 9.88497690657143 | 10.3598185288417 | 4.68499130235605 | 0.983855492418067 | 1.01372926396346 | 15.9495936462681 | 2.22752535291253 | 2.820 | 1.43863E-02 |
| ENSG00000168916 | **ZNF608** | 49.0737520060286 | 68.2063406553429 | 53.8710563499771 | 28.1099478141363 | 19.6771098483613 | 32.4393364468308 | 57.0503830037828 | 26.7421313697761 | 1.095 | 1.54300E-02 |
| ENSG00000139209 | **SLC38A4** | 13.2908078349661 | 20.7584515038 | 10.3598185288417 | 0.93699826047121 | 4.91927746209034 | 0 | 14.8030259558693 | 1.95209190752052 | 2.923 | 1.57521E-02 |
| ENSG00000105856 | **HBP1** | 31.6934648372268 | 148.274653598571 | 41.439274115367 | 25.2989530327227 | 34.4349422346324 | 21.2883145432327 | 73.8024641837217 | 27.0074032701959 | 1.452 | 1.58022E-02 |
| ENSG00000162415 | **ZSWIM5** | 192.205528690278 | 210.550008109971 | 195.800570195109 | 119.935777340315 | 170.207000188326 | 98.3317386044558 | 199.518702331786 | 129.491505377699 | 0.623 | 1.58271E-02 |
| ENSG00000197385 | **ZNF860** | 139.042297350414 | 175.952588936971 | 133.641659022058 | 113.376789517016 | 91.4985607948802 | 75.0159655332962 | 149.545515103148 | 93.2971052817309 | 0.679 | 1.63464E-02 |
| ENSG00000154133 | **ROBO4** | 8.17895866767143 | 3.95399076262857 | 6.21589111730505 | 0 | 0 | 0 | 6.11628018253501 | 0 | 5.087 | 1.64421E-02 |
| ENSG00000254680 | **AC079329.1** | 49.0737520060286 | 58.3213637487714 | 79.7706026720814 | 29.0469460746075 | 31.4833757573781 | 31.4256071828673 | 62.3885728089605 | 30.6519763382843 | 1.025 | 1.70084E-02 |
| novel.149 | **-** | 136.997557683496 | 74.1373267992857 | 83.9145300836181 | 41.2279234607332 | 6.88698844692647 | 16.2196682234154 | 98.3498048554667 | 21.4448600436917 | 2.194 | 1.86215E-02 |
| ENSG00000187688 | **TRPV2** | 47.0290123391107 | 45.4708937702286 | 26.9355281749885 | 18.7399652094242 | 15.7416878786891 | 13.178480431525 | 39.8118114281093 | 15.8867111732128 | 1.324 | 1.89277E-02 |
| novel.147 | **-** | 16.3579173353429 | 11.8619722878857 | 4.1439274115367 | 0.93699826047121 | 0 | 1.01372926396346 | 10.7879390115884 | 0.65024250814489 | 4.051 | 1.89816E-02 |
| ENSG00000212722 | **KRTAP4-9** | 13.2908078349661 | 5.93098614394286 | 6.21589111730505 | 0.93699826047121 | 0 | 0 | 8.47922836540466 | 0.312332753490403 | 4.597 | 1.93719E-02 |
| novel.651 | **-** | 143.13177668425 | 153.217142051857 | 119.13791308168 | 75.896859098168 | 101.337115719061 | 87.1807167008577 | 138.495610605929 | 88.1382305060289 | 0.654 | 1.97229E-02 |
| ENSG00000240871 | **KRTAP4-7** | 8.17895866767143 | 7.90798152525714 | 2.07196370576835 | 0 | 0 | 0 | 6.05296796623231 | 0 | 5.074 | 1.97442E-02 |
| ENSG00000182931 | **WFDC10B** | 10.2236983345893 | 5.93098614394286 | 2.07196370576835 | 0 | 0 | 0 | 6.07554939476683 | 0 | 5.079 | 2.00498E-02 |
| ENSG00000066056 | **TIE1** | 15.3355475018839 | 3.95399076262857 | 12.4317822346101 | 0.93699826047121 | 0 | 1.01372926396346 | 10.5737734997075 | 0.65024250814489 | 4.020 | 2.08820E-02 |
| ENSG00000240207 | **AC080013.1** | 127.796229182366 | 120.596718260171 | 134.677640874943 | 90.8888312657073 | 82.6438613631176 | 69.9473192134789 | 127.690196105827 | 81.1600039474346 | 0.652 | 2.09914E-02 |
| ENSG00000120885 | **CLU** | 1778.92351021854 | 1684.40006487977 | 2438.70128168935 | 1185.30279949608 | 1218.99695510599 | 1477.00353759476 | 1967.34161892922 | 1293.76776406561 | 0.605 | 2.12496E-02 |
| ENSG00000177409 | **SAMD9L** | 7.1565888342125 | 9.88497690657143 | 7.25187297018922 | 0.93699826047121 | 0 | 0 | 8.09781290365772 | 0.312332753490403 | 4.532 | 2.15274E-02 |
| ENSG00000099822 | **HCN2** | 74.6329978425018 | 74.1373267992857 | 64.2308748788188 | 33.7319373769636 | 43.289641666395 | 38.5217120306116 | 71.0003998402021 | 38.51443035799 | 0.884 | 2.15972E-02 |
| ENSG00000170500 | **LONRF2** | 15.3355475018839 | 12.8504699785429 | 16.5757096461468 | 2.81099478141363 | 2.9515664772542 | 3.04118779189039 | 14.9205757088579 | 2.93458301685274 | 2.346 | 2.32548E-02 |
| ENSG00000253471 | **AC012574.1** | 14.313177668425 | 9.88497690657143 | 14.5037459403784 | 1.87399652094242 | 0.983855492418067 | 3.04118779189039 | 12.900633505125 | 1.96634660175029 | 2.718 | 2.33020E-02 |
| ENSG00000172238 | **ATOH1** | 50.0961218394875 | 54.3673729861429 | 39.3673104095986 | 21.5509599908378 | 21.6448208331975 | 24.3295023351231 | 47.9436017450763 | 22.5084277197195 | 1.093 | 2.43760E-02 |
| ENSG00000267034 | **AC010980.2** | 20.4473966691786 | 14.8274653598571 | 12.4317822346101 | 0 | 4.91927746209034 | 4.05491705585385 | 15.9022147545486 | 2.99139817264806 | 2.426 | 2.60925E-02 |
| ENSG00000153814 | **JAZF1** | 146.198886184627 | 130.481695166743 | 133.641659022058 | 82.4558469214665 | 90.5147053024622 | 98.3317386044558 | 136.774080124476 | 90.4340969427948 | 0.598 | 2.77151E-02 |
| ENSG00000257803 | **AC010200.1** | 32.7158346706857 | 39.5399076262857 | 29.0074918807569 | 20.6139617303666 | 8.8546994317626 | 8.10983411170769 | 33.7544113925761 | 12.526165091279 | 1.423 | 2.85293E-02 |
| novel.480 | **-** | 29.6487251703089 | 35.5859168636572 | 93.2383667595757 | 10.3069808651833 | 24.5963873104517 | 26.35696086305 | 52.8243362645139 | 20.4201096795617 | 1.374 | 2.87830E-02 |
| ENSG00000254639 | **AC116021.1** | 42.939533005275 | 48.4363868422 | 49.7271289384404 | 24.3619547722515 | 18.6932543559433 | 24.3295023351231 | 47.0343495953051 | 22.4615704877726 | 1.066 | 2.91990E-02 |
| ENSG00000182326 | **C1S** | 41.9171631718161 | 42.5054006982572 | 62.1589111730505 | 20.6139617303666 | 16.7255433711071 | 30.4118779189039 | 48.8604916810412 | 22.5837943401259 | 1.115 | 2.98156E-02 |
| ENSG00000262089 | **AC040977.1** | 30.6710950037678 | 35.5859168636572 | 30.0434737336411 | 14.9919721675394 | 9.83855492418067 | 13.178480431525 | 32.100161867022 | 12.669669174415 | 1.340 | 2.99553E-02 |
| ENSG00000134668 | **SPOCD1** | 654.316693413714 | 435.9274815798 | 763.518625575637 | 405.720246784034 | 286.301948293658 | 372.038639874591 | 617.92093352305 | 354.686944984094 | 0.801 | 3.00357E-02 |
| ENSG00000112514 | **CUTA** | 425.305850718914 | 311.376772557 | 1405.82737436382 | 187.399652094242 | 179.061699620088 | 205.787040584583 | 714.169999213246 | 190.749464099638 | 1.905 | 3.09081E-02 |
| ENSG00000140961 | **OSGIN1** | 591.95213357272 | 428.019500054543 | 750.050861488142 | 275.477488578536 | 331.559300944889 | 412.587810433129 | 590.007498371802 | 339.874866652184 | 0.796 | 3.33691E-02 |
| ENSG00000087076 | **HSD17B14** | 75.6553676759607 | 91.9302852311143 | 52.8350744970929 | 48.7239095445029 | 40.3380751891408 | 27.3706901270135 | 73.4735758013893 | 38.810891620219 | 0.919 | 3.34629E-02 |
| ENSG00000256894 | **AC022509.3** | 18.4026570022607 | 13.8389676692 | 19.6836552047993 | 1.87399652094242 | 4.91927746209034 | 6.08237558378077 | 17.30842662542 | 4.29188318893784 | 2.021 | 3.38968E-02 |
| ENSG00000250510 | **GPR162** | 33.7382045041446 | 30.6434284103714 | 14.5037459403784 | 5.62198956282726 | 12.7901214014349 | 7.09610484774423 | 26.2951262849648 | 8.50273860400212 | 1.632 | 3.40248E-02 |
| novel.634 | **-** | 30.6710950037678 | 41.5169030076 | 49.7271289384404 | 19.6769634698954 | 16.7255433711071 | 18.2471267513423 | 40.6383756499361 | 18.2165445307816 | 1.157 | 3.50819E-02 |
| ENSG00000239467 | **AC007405.3** | 63.3869296744535 | 73.1488291086286 | 67.3388204374713 | 36.5429321583772 | 36.4026532194685 | 41.5628998225019 | 67.9581930735178 | 38.1694950667825 | 0.833 | 3.51957E-02 |
| ENSG00000132801 | **ZSWIM3** | 127.796229182366 | 148.274653598571 | 113.958003817259 | 83.3928451819377 | 89.5308498100441 | 85.1532581729308 | 130.009628866066 | 86.0256510549709 | 0.597 | 3.74530E-02 |
| ENSG00000232284 | **GNG12-AS1** | 29.6487251703089 | 53.3788752954857 | 29.0074918807569 | 16.8659686884818 | 15.7416878786891 | 15.2059389594519 | 37.3450307821838 | 15.9378651755409 | 1.229 | 4.06910E-02 |
| ENSG00000265982 | **AC103810.3** | 107.348832513187 | 70.1833360366572 | 100.490239729765 | 59.0308904096862 | 47.2250636360672 | 59.8100265738442 | 92.6741360932032 | 55.3553268731992 | 0.742 | 4.08625E-02 |
| ENSG00000213139 | **CRYGS** | 95.0803945116803 | 157.171132814486 | 129.497731610522 | 83.3928451819377 | 82.6438613631176 | 78.0571533251866 | 127.249752978896 | 81.3646199567473 | 0.646 | 4.08769E-02 |
| novel.521 | **-** | 23.5145061695554 | 11.8619722878857 | 16.5757096461468 | 6.55898782329847 | 2.9515664772542 | 4.05491705585385 | 17.3173960345293 | 4.52182378546884 | 1.930 | 4.47600E-02 |
| ENSG00000285257 | **AL157893.1** | 62.3645598409946 | 92.9187829217715 | 56.9790019086296 | 40.290925200262 | 24.5963873104517 | 48.6590046702462 | 70.7541148904652 | 37.8487723936533 | 0.905 | 4.57206E-02 |
| ENSG00000167772 | **ANGPTL4** | 37.8276838379803 | 29.6549307197143 | 37.2953467038303 | 11.2439791256545 | 20.6609653407794 | 13.178480431525 | 34.925987087175 | 15.0278082993196 | 1.218 | 4.57997E-02 |
| ENSG00000122035 | **RASL11A** | 131.885708516202 | 114.665732116229 | 163.6851327557 | 103.069808651833 | 75.7568729161912 | 91.2356337567116 | 136.74552446271 | 90.0207717749119 | 0.601 | 4.67929E-02 |
| ENSG00000267147 | **LINC01842** | 17.3802871688018 | 34.597419173 | 18.6476733519151 | 8.43298434424089 | 9.83855492418067 | 5.06864631981731 | 23.541793231239 | 7.78006186274629 | 1.596 | 4.68057E-02 |
| novel.803 | **-** | 72.5882581755839 | 26.6894376477429 | 40.4032922624828 | 11.2439791256545 | 29.515664772542 | 18.2471267513423 | 46.5603293619365 | 19.6689235498463 | 1.245 | 4.72181E-02 |
| ENSG00000088756 | **ARHGAP28** | 36.8053140045214 | 28.6664330290571 | 22.7916007634518 | 11.2439791256545 | 9.83855492418067 | 14.1922096954885 | 29.4211159323435 | 11.7582479151079 | 1.325 | 4.83000E-02 |
| ENSG00000273017 | **AP000240.1** | 34.7605743376036 | 24.7124422664286 | 32.1154374394094 | 16.8659686884818 | 8.8546994317626 | 11.1510219035981 | 30.5294846811472 | 12.2905633412808 | 1.308 | 4.87679E-02 |
| ENSG00000215068 | **AC025171.2** | 81.7895866767143 | 117.6312251882 | 87.0224756422706 | 47.7869112840317 | 64.9344624995924 | 62.8512143657346 | 95.4810958357283 | 58.5241960497863 | 0.709 | 4.91692E-02 |
| ENSG00000116132 | **PRRX1** | 11.2460681680482 | 6.9194838346 | 7.25187297018922 | 0 | 1.96771098483613 | 0 | 8.47247499094581 | 0.655903661612045 | 3.698 | 4.98592E-02 |
| ENSG00000163637 | **PRICKLE2** | 28.62635533685 | 15.8159630505143 | 34.1874011451778 | 3703.95412364269 | 4019.0496865278 | 3808.58084471073 | 26.2099065108473 | 3843.86155162707 | -7.204 | 0.00000E+00 |
| ENSG00000099812 | **MISP** | 46.0066425056518 | 37.5629122449714 | 67.3388204374713 | 2380.91257985734 | 2430.12306627263 | 2727.94544932568 | 50.3027917293648 | 2512.99369848522 | -5.648 | 0.00000E+00 |
| ENSG00000163636 | **PSMD6** | 3156.05567588771 | 2853.79283292717 | 3173.21241538423 | 31979.7506298824 | 31744.0974628689 | 31331.3303613187 | 3061.02030806637 | 31685.05948469 | -3.372 | 0.00000E+00 |
| ENSG00000117115 | **PADI2** | 305.68858020422 | 418.134523147972 | 300.434737336411 | 3612.12829411651 | 3773.08581342329 | 3539.94258976041 | 341.419280229534 | 3641.71889910007 | -3.413 | 2.93745E-276 |
| ENSG00000181885 | **CLDN7** | 452.909836222305 | 410.226541622714 | 401.96095891906 | 2667.63404756153 | 2598.36235547612 | 2492.76026008615 | 421.699112254693 | 2586.25222104127 | -2.617 | 9.75004E-232 |
| ENSG00000163735 | **CXCL5** | 26.5816156699321 | 12.8504699785429 | 22.7916007634518 | 1707.21083057854 | 1477.75094961194 | 1894.65999434771 | 20.7412288039756 | 1693.2072581794 | -6.357 | 4.42035E-197 |
| ENSG00000175874 | **CREG2** | 61.3421900075357 | 85.0108013965143 | 79.7706026720814 | 1169.37382906807 | 1336.07575870374 | 1220.53003381201 | 75.3745313587105 | 1241.9932071946 | -4.041 | 5.23777E-191 |
| ENSG00000129354 | **AP1M2** | 35.7829441710625 | 37.5629122449714 | 53.8710563499771 | 885.463356145293 | 917.937174426057 | 997.509595740047 | 42.4056375886703 | 933.636708770465 | -4.463 | 1.67912E-155 |
| novel.117 | **-** | 129.840968849284 | 121.585215950829 | 101.526221582649 | 1304.30157857592 | 1471.84781665743 | 1145.51406827871 | 117.650802127587 | 1307.22115450402 | -3.473 | 8.82667E-155 |
| ENSG00000204869 | **IGFL4** | 18.4026570022607 | 31.6319261010286 | 25.8995463221044 | 865.786392675398 | 786.100538442036 | 782.598991779793 | 25.3113764751312 | 811.495307632409 | -5.000 | 7.18340E-136 |
| ENSG00000167693 | **NXN** | 884.349905941973 | 894.590410044715 | 1021.4781069438 | 3227.02200906285 | 3326.41541986549 | 3764.9904863603 | 933.472807643494 | 3439.47597176288 | -1.882 | 1.06382E-118 |
| ENSG00000170961 | **HAS2** | 9.20132850113035 | 19.7699538131429 | 13.4677640874943 | 698.063704051051 | 823.487047153922 | 728.871340789729 | 14.1463488005892 | 750.140697331567 | -5.723 | 1.76544E-111 |
| ENSG00000062038 | **CDH3** | 220.831884027128 | 261.951888024143 | 183.368787960499 | 1410.18238200917 | 1263.2704522648 | 1297.57345787323 | 222.050853337257 | 1323.67543071573 | -2.574 | 1.07505E-110 |
| ENSG00000021355 | **SERPINB1** | 133.93044818312 | 103.792257519 | 137.785586433595 | 986.659168276184 | 868.744399805153 | 1024.88028586706 | 125.169430711905 | 960.094617982799 | -2.941 | 1.22264E-110 |
| ENSG00000159267 | **HLCS** | 1623.52329553278 | 1581.59630505143 | 1626.49150902815 | 4032.84051306809 | 4011.17884258846 | 4227.25103072764 | 1610.53703653745 | 4090.42346212806 | -1.345 | 7.92100E-110 |
| ENSG00000183421 | **RIPK4** | 391.56764621477 | 381.560108593657 | 376.061412596955 | 1338.97051421336 | 1402.97793218816 | 1421.24842807677 | 383.063055801794 | 1387.73229149277 | -1.857 | 2.02936E-96 |
| ENSG00000039068 | **CDH1** | 36.8053140045214 | 37.5629122449714 | 17.611691499031 | 520.034034561521 | 559.81377518588 | 595.059077946552 | 30.6599725828413 | 558.302295897985 | -4.182 | 1.50766E-92 |
| ENSG00000104327 | **CALB1** | 2867.74738285229 | 2836.988372186 | 2880.029551018 | 6597.40475197779 | 6118.59730734796 | 6729.13485418946 | 2861.5884353521 | 6481.71230450507 | -1.180 | 5.00116E-92 |
| ENSG00000183018 | **SPNS2** | 62.3645598409946 | 73.1488291086286 | 40.4032922624828 | 603.426879743459 | 613.925827268874 | 603.16891205826 | 58.6388937373687 | 606.840539690198 | -3.368 | 5.93910E-88 |
| ENSG00000223756 | **TSSC2** | 4.08947933383571 | 1.97699538131429 | 8.2878548230734 | 1204.97976296598 | 1162.91719203816 | 1189.10442662914 | 4.78477651274113 | 1185.66712721109 | -7.967 | 2.85456E-87 |
| ENSG00000165025 | **SYK** | 2.04473966691786 | 5.93098614394286 | 3.10794555865252 | 1659.42391929451 | 1519.0728802935 | 1583.44511031093 | 3.69455712317108 | 1587.31396996631 | -8.736 | 6.93397E-84 |
| ENSG00000124225 | **PMEPA1** | 22.4921363360964 | 11.8619722878857 | 20.7196370576835 | 664.331766674088 | 473.23449185309 | 471.38410774301 | 18.3579152272219 | 536.316788756729 | -4.874 | 4.32507E-81 |
| ENSG00000143867 | **OSR1** | 72.5882581755839 | 65.2408475833714 | 52.8350744970929 | 536.900003250003 | 547.023653784445 | 542.345156220452 | 63.5547267520161 | 542.0896044183 | -3.091 | 1.13155E-80 |
| ENSG00000124766 | **SOX4** | 2015.09094174755 | 1968.09890209837 | 1925.89026451168 | 4198.68920517149 | 4255.17500470814 | 4164.3998163619 | 1969.69336945253 | 4206.08800874718 | -1.094 | 2.00450E-79 |
| ENSG00000158710 | **TAGLN2** | 888.439385275809 | 866.912474706314 | 1070.16925402935 | 3087.40926825264 | 2702.65103767243 | 3040.17406262642 | 941.840371337158 | 2943.41145618383 | -1.644 | 3.04062E-79 |
| ENSG00000163162 | **RNF149** | 2984.29754386661 | 3131.56068400183 | 2812.69073058053 | 6691.10457802491 | 6928.31037760803 | 6317.56077302029 | 2976.18298614966 | 6645.65857621774 | -1.159 | 4.43712E-78 |
| ENSG00000134369 | **NAV1** | 57.2527106737 | 76.1143221806 | 40.4032922624828 | 529.404017166234 | 571.620041094897 | 584.921785306918 | 57.9234417055943 | 561.981947856016 | -3.274 | 1.88415E-76 |
| ENSG00000164292 | **RHOBTB3** | 2681.67607316277 | 2974.38955118734 | 2608.60230556235 | 6201.99148605894 | 6079.24308765124 | 6032.70284984656 | 2754.88930997082 | 6104.64580785225 | -1.148 | 2.64836E-76 |
| ENSG00000184363 | **PKP3** | 2042.69492725094 | 1731.84795403131 | 1914.49446412995 | 5088.83755261914 | 4489.33261190364 | 4681.40174098327 | 1896.34578180407 | 4753.19063516868 | -1.326 | 9.05379E-73 |
| ENSG00000142408 | **CACNG8** | 998.855327289373 | 876.797451612886 | 1153.04780226009 | 2873.7736648652 | 2921.06695698924 | 2753.28868092476 | 1009.56686038745 | 2849.37643425973 | -1.498 | 4.75165E-66 |
| ENSG00000137449 | **CPEB2** | 113.483051513941 | 142.343667454629 | 117.065949375912 | 639.969811901836 | 606.054983329529 | 745.091009013144 | 124.297556114827 | 663.705268081503 | -2.415 | 2.25464E-63 |
| ENSG00000149418 | **ST14** | 2066.20943342049 | 1878.14561224857 | 1991.15712124338 | 3974.74662091887 | 3897.05160546796 | 3922.11852227463 | 1978.50405563748 | 3931.30558288716 | -0.991 | 7.43932E-58 |
| ENSG00000119888 | **EPCAM** | 5547.37871634814 | 5366.55396257763 | 5898.88067032249 | 10027.7553835629 | 10450.5130404647 | 10640.1023545605 | 5604.27111641609 | 10372.7902595294 | -0.888 | 2.29056E-57 |
| ENSG00000182107 | **TMEM30B** | 187.093679522984 | 171.010100483686 | 192.692624636456 | 837.676444861262 | 725.101497912116 | 687.308440967227 | 183.598801547709 | 750.028794580201 | -2.032 | 2.71818E-57 |
| ENSG00000136205 | **TNS3** | 141.087037017332 | 193.7455473688 | 250.70760839797 | 2679.81502494766 | 2929.921656421 | 3233.79635204344 | 195.180064261367 | 2947.8443444707 | -3.917 | 4.42493E-56 |
| ENSG00000104413 | **ESRP1** | 812.784017599848 | 930.176326908372 | 836.037355277529 | 2007.05027392933 | 1999.19436059351 | 1962.57985503326 | 859.665899928583 | 1989.60816318537 | -1.210 | 1.01416E-54 |
| ENSG00000183742 | **MACC1** | 253.547718697814 | 267.882874168086 | 196.836552047993 | 927.628277866498 | 837.261024047775 | 862.683603632906 | 239.422381637964 | 875.857635182393 | -1.870 | 3.76484E-53 |
| ENSG00000168615 | **ADAM9** | 6069.80970124566 | 6014.01994995806 | 6653.07545922217 | 11069.6974492069 | 11302.5318968988 | 11619.3648235492 | 6245.63503680863 | 11330.5313898849 | -0.859 | 1.25487E-52 |
| ENSG00000166689 | **PLEKHA7** | 17.3802871688018 | 12.8504699785429 | 8.2878548230734 | 280.162479880892 | 328.607734467634 | 268.638254950317 | 12.8395373234727 | 292.469489766281 | -4.508 | 5.31867E-52 |
| ENSG00000115339 | **GALNT3** | 77.7001073428786 | 120.596718260171 | 98.4182760239966 | 521.908031082464 | 528.330399428502 | 502.809714925877 | 98.9050338756822 | 517.682715145614 | -2.386 | 1.88324E-51 |
| ENSG00000070404 | **FSTL3** | 205.496336525245 | 211.538505800629 | 200.98047945953 | 698.063704051051 | 665.086312874613 | 730.898799317656 | 206.005107261801 | 698.016272081107 | -1.760 | 3.65832E-50 |
| ENSG00000159166 | **LAD1** | 4.08947933383571 | 4.94248845328572 | 4.1439274115367 | 409.468239825919 | 347.300988823578 | 372.038639874591 | 4.39196506621938 | 376.269289508029 | -6.419 | 1.43610E-48 |
| ENSG00000196155 | **PLEKHG4** | 120.639640348154 | 140.366672073314 | 124.317822346101 | 583.749916273564 | 498.81473465596 | 554.509907388014 | 128.441378255856 | 545.691519439179 | -2.086 | 1.77758E-48 |
| ENSG00000131149 | **GSE1** | 581.72843523813 | 518.961287595 | 573.933946497833 | 1534.80315065184 | 1343.94660264308 | 1360.42467223897 | 558.207889776988 | 1413.05814184463 | -1.341 | 1.49134E-47 |
| ENSG00000188191 | **PRKAR1B** | 1054.06329829615 | 913.3718661672 | 963.463123182282 | 2154.15900082331 | 2047.403279722 | 2168.36689561784 | 976.966095881879 | 2123.30972538772 | -1.120 | 2.25078E-47 |
| ENSG00000164251 | **F2RL1** | 4361.42970953579 | 4247.57457675374 | 4686.781902448 | 8186.55380173696 | 7888.55333820806 | 7677.98544525926 | 4431.92872957918 | 7917.69752840143 | -0.837 | 3.18901E-47 |
| ENSG00000130545 | **CRB3** | 183.004200189148 | 154.205639742514 | 114.993985670143 | 666.20576319503 | 670.005590336704 | 617.361121753748 | 150.734608533935 | 651.190825095161 | -2.110 | 4.93275E-47 |
| ENSG00000092820 | **EZR** | 11035.4599823557 | 10053.0215139831 | 11498.3625851615 | 19862.4891254687 | 19095.6512523423 | 21131.1865073184 | 10862.2813605001 | 20029.7756283764 | -0.883 | 9.41360E-47 |
| ENSG00000119411 | **BSPRY** | 304.666210370761 | 276.779353384 | 252.779572103739 | 817.999481391366 | 820.535480676668 | 836.326642769856 | 278.075045286166 | 824.953868279297 | -1.569 | 4.51701E-46 |
| ENSG00000132688 | **NES** | 104.281723012811 | 130.481695166743 | 111.886040111491 | 568.757944106024 | 522.427266473994 | 461.246815103375 | 115.549819430348 | 517.477341894464 | -2.163 | 1.47184E-45 |
| ENSG00000137501 | **SYTL2** | 6510.45109946646 | 7793.31579314092 | 8259.88331304552 | 15737.8227828744 | 15130.7136178975 | 15270.8176323456 | 7521.21673521763 | 15379.7846777058 | -1.032 | 6.22255E-45 |
| ENSG00000188064 | **WNT7B** | 233.100322028636 | 231.308459613771 | 330.478211070052 | 908.888312657073 | 920.888740903311 | 912.356337567116 | 264.962330904153 | 914.044463709167 | -1.788 | 6.56620E-45 |
| ENSG00000078018 | **MAP2** | 108.371202346646 | 165.079114339743 | 170.937005725889 | 674.638747539271 | 618.845104730964 | 622.429768073566 | 148.129107470759 | 638.637873447934 | -2.108 | 6.61827E-45 |
| ENSG00000122641 | **INHBA** | 62.3645598409946 | 80.0683129432286 | 88.0584574951548 | 584.686914534035 | 460.444370451655 | 389.272037361969 | 76.8304434264593 | 478.134440782553 | -2.639 | 1.98353E-44 |
| ENSG00000185101 | **ANO9** | 469.267753557648 | 532.8002552642 | 483.803525296909 | 1157.19285168194 | 1189.48129033344 | 1193.15934368499 | 495.290511372919 | 1179.94449523346 | -1.252 | 1.07709E-42 |
| novel.26 | **-** | 69.5211486752071 | 56.3443683674572 | 98.4182760239966 | 439.452184160997 | 388.622919505137 | 465.301732159229 | 74.7612643555536 | 431.125611941788 | -2.530 | 2.01948E-42 |
| ENSG00000188042 | **ARL4C** | 1254.44778565411 | 1421.45967916497 | 1232.81840493217 | 2655.45307017541 | 2599.34621096853 | 2554.59774518792 | 1302.90862325041 | 2603.13234211062 | -0.998 | 1.05137E-41 |
| ENSG00000101596 | **SMCHD1** | 4097.65829250338 | 4211.00016219943 | 4805.91981552968 | 7753.66060539926 | 8361.78783006115 | 8316.63488155624 | 4371.5260900775 | 8144.02777233888 | -0.898 | 1.29791E-40 |
| ENSG00000164674 | **SYTL3** | 12.2684380015071 | 16.8044607411714 | 18.6476733519151 | 208.95061208508 | 221.367485794065 | 243.295023351231 | 15.9068573648646 | 224.537707076792 | -3.819 | 2.09288E-40 |
| ENSG00000049323 | **LTBP1** | 145.176516351168 | 178.918082008943 | 140.893531992248 | 564.072952803668 | 537.185098860265 | 542.345156220452 | 154.996043450786 | 547.867735961462 | -1.820 | 7.78761E-40 |
| ENSG00000075213 | **SEMA3A** | 3522.06407626601 | 3808.68160210197 | 3745.07439817629 | 6021.15082178799 | 6128.43586227214 | 6346.95892167523 | 3691.94002551476 | 6165.51520191179 | -0.740 | 3.22043E-38 |
| ENSG00000180801 | **ARSJ** | 1133.80814530595 | 1084.38196665089 | 1270.113751636 | 2225.37086861912 | 2362.23703729578 | 2502.89755272579 | 1162.76795453094 | 2363.5018195469 | -1.023 | 6.56821E-37 |
| ENSG00000235750 | **KIAA0040** | 244.346390196684 | 261.951888024143 | 217.556189105677 | 755.220597939795 | 715.262942987935 | 646.759270408689 | 241.284822442168 | 705.747603778806 | -1.548 | 1.49575E-36 |
| ENSG00000085552 | **IGSF9** | 36.8053140045214 | 47.4478891515429 | 38.3313285567145 | 259.548518150525 | 279.414959846731 | 258.500962310683 | 40.8615105709262 | 265.821480102646 | -2.700 | 1.93397E-36 |
| ENSG00000145349 | **CAMK2D** | 1913.87632823511 | 2057.06369425751 | 1990.1211393905 | 3377.87872899871 | 3370.6889170243 | 3405.11659765327 | 1987.02038729438 | 3384.56141455876 | -0.768 | 1.05106E-35 |
| ENSG00000166949 | **SMAD3** | 3637.59186744687 | 4143.78231923474 | 4804.8838336768 | 8169.68783304848 | 8573.31676093104 | 9369.89958681428 | 4195.41934011947 | 8704.30139359793 | -1.053 | 2.33656E-35 |
| ENSG00000132698 | **RAB25** | 493.804629560662 | 497.214338400543 | 469.299779356531 | 1168.4368308076 | 1021.24200112995 | 1115.10219035981 | 486.772915772579 | 1101.59367409912 | -1.178 | 7.12079E-35 |
| ENSG00000124839 | **RAB17** | 390.545276381311 | 382.548606284314 | 302.506701042179 | 922.00628830367 | 942.533561736508 | 904.246503455408 | 358.533527902601 | 922.928784498529 | -1.364 | 1.66883E-34 |
| ENSG00000173801 | **JUP** | 2116.30555525998 | 2143.06299334469 | 1726.98174875792 | 4179.0122417016 | 3881.30991758927 | 3825.81424219811 | 1995.45009912086 | 3962.04546716299 | -0.989 | 4.87588E-34 |
| ENSG00000083307 | **GRHL2** | 6.13421900075357 | 1.97699538131429 | 2.07196370576835 | 228.627575554975 | 206.609653407794 | 260.52842083861 | 3.3943926959454 | 231.921883267126 | -6.098 | 8.97973E-33 |
| ENSG00000110090 | **CPT1A** | 3348.26120457799 | 2993.17100730983 | 3375.22887669664 | 5336.20509338354 | 5437.76930659466 | 5596.79926634227 | 3238.88702952815 | 5456.92455544016 | -0.753 | 1.07479E-32 |
| ENSG00000073350 | **LLGL2** | 2369.8532739578 | 2463.3362451176 | 2276.05213078653 | 3822.95290272254 | 4168.59572137535 | 4241.44324042312 | 2369.74721662064 | 4077.66395484034 | -0.783 | 1.12357E-32 |
| ENSG00000260220 | **CCDC187** | 38.8500536714393 | 59.3098614394286 | 76.6626571134289 | 314.831415518326 | 302.043636172347 | 363.928805762883 | 58.2741907414323 | 326.934619151185 | -2.489 | 4.15390E-32 |
| ENSG00000176871 | **WSB2** | 1658.28386987038 | 1559.84935585697 | 1498.02975927052 | 2873.7736648652 | 2746.92453483124 | 2681.31390318336 | 1572.05432833262 | 2767.3373676266 | -0.816 | 4.42524E-32 |
| ENSG00000130433 | **CACNG6** | 328.180716540316 | 343.997196348686 | 410.248813742133 | 845.172430945031 | 909.082474994294 | 1009.67434690761 | 360.808908877045 | 921.309750948978 | -1.352 | 1.26340E-31 |
| ENSG00000111885 | **MAN1A1** | 729.972061089675 | 923.256843073772 | 776.986389663131 | 1653.80192973169 | 1730.60181116338 | 1710.16126830636 | 810.071764608859 | 1698.18833640048 | -1.067 | 2.27702E-31 |
| ENSG00000139793 | **MBNL2** | 549.012600567444 | 669.212936574886 | 595.6895654084 | 1236.837703822 | 1481.68637158161 | 1358.39721371104 | 604.63836751691 | 1358.97376303821 | -1.168 | 4.85920E-31 |
| ENSG00000176658 | **MYO1D** | 2174.58063576714 | 2253.77473469829 | 2006.69684903665 | 3703.01712538222 | 3667.81327573455 | 3529.80529712077 | 2145.01740650069 | 3633.54523274585 | -0.760 | 5.62340E-31 |
| ENSG00000115112 | **TFCP2L1** | 265.816156699321 | 265.905878786771 | 252.779572103739 | 646.528799725135 | 663.118601889777 | 645.745541144725 | 261.500535863277 | 651.797647586546 | -1.317 | 6.39348E-31 |
| novel.63 | **-** | 1817.77356388997 | 1806.97377852126 | 2102.00717950199 | 3480.01153939007 | 3420.86554713762 | 3271.30433481009 | 1908.91817397107 | 3390.72714044593 | -0.829 | 7.61861E-30 |
| ENSG00000102271 | **KLHL4** | 9.20132850113035 | 17.7929584318286 | 16.5757096461468 | 167.722688624347 | 172.174711173162 | 166.251599290008 | 14.5233321930352 | 168.716333029172 | -3.536 | 1.88964E-29 |
| ENSG00000049130 | **KITLG** | 9615.38828368122 | 9495.50881645252 | 10457.2008230129 | 15189.6788004988 | 15462.2729188423 | 14717.3214542215 | 9856.03264104887 | 15123.0910578542 | -0.618 | 3.03259E-29 |
| ENSG00000156802 | **ATAD2** | 2902.5079571899 | 3270.93885838449 | 3198.07597985345 | 4941.72882572516 | 5126.87097099055 | 5077.76988319298 | 3123.84093180928 | 5048.7898933029 | -0.693 | 2.36230E-28 |
| ENSG00000135525 | **MAP7** | 790.291881263752 | 948.957783030857 | 787.346208191973 | 1617.25899757331 | 1698.13457991358 | 1663.52972216404 | 842.198624162194 | 1659.64109988364 | -0.978 | 2.91985E-28 |
| ENSG00000135480 | **KRT7** | 3.06710950037678 | 2.96549307197143 | 3.10794555865252 | 175.218674708116 | 169.223144695908 | 280.803006117879 | 3.04684937700025 | 208.414941840634 | -6.096 | 3.89445E-28 |
| ENSG00000140832 | **MARVELD3** | 537.766532399396 | 630.661526639257 | 579.113855762253 | 1184.36580123561 | 1156.03020359123 | 1176.93967546158 | 582.513971600302 | 1172.44522676281 | -1.009 | 4.52438E-28 |
| ENSG00000128567 | **PODXL** | 684.987788417482 | 798.706134050972 | 708.611587372775 | 1489.82723414922 | 1436.42901893038 | 1369.54823561464 | 730.76850328041 | 1431.93482956475 | -0.970 | 5.50772E-28 |
| ENSG00000081923 | **ATP8B1** | 16.3579173353429 | 6.9194838346 | 4.1439274115367 | 143.360733852095 | 167.255433711071 | 164.224140762081 | 9.14044286049318 | 158.280102775082 | -4.114 | 7.71026E-28 |
| ENSG00000100867 | **DHRS2** | 615.466639742275 | 643.5119966178 | 687.891950315092 | 1251.82967598954 | 1215.06153313631 | 1245.87326541109 | 648.956862225056 | 1237.58815817898 | -0.932 | 7.15568E-27 |
| ENSG00000162496 | **DHRS3** | 3.06710950037678 | 3.95399076262857 | 8.2878548230734 | 123.6837703822 | 149.546034847546 | 178.416350457569 | 5.10298502869292 | 150.548718562438 | -4.888 | 2.61303E-26 |
| ENSG00000079308 | **TNS1** | 7.1565888342125 | 13.8389676692 | 10.3598185288417 | 160.226702540577 | 145.610612877874 | 131.78480431525 | 10.4517916774181 | 145.874039911234 | -3.800 | 2.61303E-26 |
| ENSG00000165272 | **AQP3** | 23.5145061695554 | 13.8389676692 | 29.0074918807569 | 149.919721675394 | 206.609653407794 | 262.555879366537 | 22.1203219065041 | 206.361751483241 | -3.225 | 2.74590E-26 |
| ENSG00000183087 | **GAS6** | 33.7382045041446 | 18.7814561224857 | 36.2593648509461 | 201.45462600131 | 180.045555112506 | 229.102813655742 | 29.5930084925255 | 203.534331589853 | -2.786 | 4.89755E-26 |
| ENSG00000132470 | **ITGB4** | 1974.19614840919 | 2153.93646794191 | 1821.25609737038 | 3458.46057939924 | 3238.85228104028 | 3482.16002171449 | 1983.12957124049 | 3393.15762738467 | -0.775 | 5.60051E-26 |
| ENSG00000167355 | **OR51B5** | 55.2079710067821 | 58.3213637487714 | 63.1948930259346 | 290.469460746075 | 225.302907763737 | 256.473503782756 | 58.9080759271627 | 257.415290764189 | -2.128 | 4.96384E-25 |
| ENSG00000156711 | **MAPK13** | 1059.17514746345 | 949.946280721515 | 969.679014299587 | 1712.83282014137 | 1752.24663199658 | 1721.31229020996 | 992.933480828184 | 1728.7972474493 | -0.800 | 5.58338E-25 |
| ENSG00000175707 | **KDF1** | 126.773859348907 | 117.6312251882 | 137.785586433595 | 363.555325062829 | 372.881231626447 | 398.395600737641 | 127.396890323567 | 378.277385808972 | -1.571 | 2.83718E-24 |
| ENSG00000187017 | **ESPN** | 51.1184916729464 | 54.3673729861429 | 60.0869474672821 | 264.233509452881 | 210.545075377466 | 245.322481879158 | 55.1909373754571 | 240.033688903168 | -2.122 | 1.03658E-23 |
| ENSG00000082781 | **ITGB5** | 1960.90534057422 | 1726.90546557803 | 1962.14962936263 | 3179.23509777881 | 3089.30624619273 | 2987.46014090032 | 1883.32014517163 | 3085.33382829062 | -0.713 | 1.63813E-23 |
| ENSG00000149485 | **FADS1** | 81.7895866767143 | 101.815262137686 | 66.3028385845872 | 289.532462485604 | 330.575445452471 | 305.132508453002 | 83.3025624663291 | 308.413472130359 | -1.886 | 1.84455E-23 |
| ENSG00000189159 | **JPT1** | 8189.18236600602 | 7188.35520645874 | 7933.54902938701 | 11491.3466664189 | 11670.4938510631 | 11918.4149564184 | 7770.36220061726 | 11693.4184913001 | -0.590 | 2.56640E-23 |
| ENSG00000108984 | **MAP2K6** | 30.6710950037678 | 36.5744145543143 | 26.9355281749885 | 190.210646875656 | 194.803387498777 | 162.196682234154 | 31.3936792443569 | 182.403572202862 | -2.537 | 3.11999E-23 |
| ENSG00000137460 | **FHDC1** | 114.5054213474 | 153.217142051857 | 101.526221582649 | 435.704191119113 | 367.961954164357 | 391.299495889896 | 123.082928327302 | 398.321880391122 | -1.693 | 5.44567E-23 |
| ENSG00000256747 | **AC009511.2** | 170.735762187641 | 163.102118958429 | 116.029967523028 | 513.475046738223 | 424.041717232187 | 433.876124976362 | 149.955949556366 | 457.130962982257 | -1.608 | 7.27910E-23 |
| ENSG00000214049 | **UCA1** | 0 | 0 | 3.10794555865252 | 475.995116319375 | 369.929665149193 | 396.368142209714 | 1.03598185288417 | 414.097641226094 | -8.672 | 9.57647E-23 |
| ENSG00000035115 | **SH3YL1** | 730.994430923134 | 764.108714877972 | 884.728502363085 | 1411.11938026964 | 1461.02540624083 | 1516.53897888934 | 793.27721605473 | 1462.8945884666 | -0.883 | 2.29881E-22 |
| ENSG00000088992 | **TESC** | 16.3579173353429 | 21.7469491944571 | 17.611691499031 | 143.360733852095 | 150.529890339964 | 130.771075051287 | 18.5721860096103 | 141.553899747782 | -2.928 | 8.84465E-22 |
| ENSG00000143850 | **PLEKHA6** | 52.1408615064053 | 54.3673729861429 | 46.6191833797878 | 193.95863991754 | 243.996162119681 | 216.938062488181 | 51.042472624112 | 218.297621508467 | -2.095 | 1.82268E-21 |
| ENSG00000183696 | **UPP1** | 960.005273617934 | 699.856364985257 | 1030.80194361975 | 2160.71798864661 | 1918.51821021523 | 1773.01248267209 | 896.887860740982 | 1950.74956051131 | -1.122 | 2.35671E-21 |
| ENSG00000052344 | **PRSS8** | 30.6710950037678 | 20.7584515038 | 11.3958003817259 | 186.462653833771 | 170.207000188326 | 133.812262843177 | 20.9417822964313 | 163.493972288424 | -2.964 | 3.25158E-21 |
| ENSG00000242265 | **PEG10** | 458.0216853896 | 657.350964287 | 408.176850036365 | 2192.57592950263 | 2270.7384765009 | 2179.51791752144 | 507.849833237655 | 2214.27744117499 | -2.124 | 3.54923E-21 |
| ENSG00000189052 | **CGB5** | 8.17895866767143 | 2.96549307197143 | 14.5037459403784 | 187.399652094242 | 99.3694047342248 | 126.716157995433 | 8.54939922667377 | 137.8284049413 | -4.022 | 4.51758E-21 |
| ENSG00000162069 | **BICDL2** | 6.13421900075357 | 3.95399076262857 | 3.10794555865252 | 120.872775600786 | 101.337115719061 | 115.565136091835 | 4.39871844067822 | 112.591675803894 | -4.678 | 5.67951E-21 |
| ENSG00000104332 | **SFRP1** | 5.11184916729464 | 11.8619722878857 | 4.1439274115367 | 132.116754726441 | 140.691335415784 | 91.2356337567116 | 7.03924962223902 | 121.347907966312 | -4.098 | 6.21074E-21 |
| ENSG00000187800 | **PEAR1** | 42.939533005275 | 41.5169030076 | 30.0434737336411 | 176.155672968587 | 205.625797915376 | 180.443808985496 | 38.166636582172 | 187.408426623153 | -2.294 | 7.13144E-21 |
| ENSG00000163734 | **CXCL3** | 76.6777375094196 | 54.3673729861429 | 104.634167141302 | 277.351485099478 | 303.027491664765 | 334.530657107942 | 78.559759212288 | 304.969877957395 | -1.959 | 1.26545E-20 |
| ENSG00000255190 | **TRIM51DP** | 102.236983345893 | 124.5507090228 | 105.670148994186 | 314.831415518326 | 335.494722914561 | 324.393364468308 | 110.819280454293 | 324.906500967065 | -1.551 | 1.29392E-20 |
| ENSG00000150687 | **PRSS23** | 106.326462679729 | 114.665732116229 | 179.224860548962 | 377.610298969898 | 454.541237497147 | 530.180405052891 | 133.405685114973 | 454.110647173312 | -1.768 | 2.41906E-20 |
| ENSG00000126583 | **PRKCG** | 244.346390196684 | 272.825362621371 | 220.664134664329 | 555.639968459427 | 575.555463064569 | 537.276509900635 | 245.945295827461 | 556.15731380821 | -1.176 | 3.18846E-20 |
| ENSG00000275234 | **AC010503.4** | 62.3645598409946 | 72.1603314179714 | 47.655165232672 | 225.816580773562 | 225.302907763737 | 242.281294087267 | 60.7266854972127 | 231.133594208189 | -1.926 | 1.05293E-19 |
| ENSG00000168528 | **SERINC2** | 530.609943565184 | 463.6054169182 | 467.227815650763 | 913.573303959429 | 886.453798668678 | 946.823132541873 | 487.147725378049 | 915.61674505666 | -0.910 | 1.28527E-19 |
| ENSG00000092871 | **RFFL** | 1390.42297350414 | 1280.104509401 | 1430.69093883304 | 2082.01013476703 | 2247.12594468287 | 2347.79697533938 | 1367.07280724606 | 2225.64435159642 | -0.703 | 1.75084E-19 |
| ENSG00000121281 | **ADCY7** | 887.41701544235 | 965.762243772029 | 905.448139420768 | 1452.34730373038 | 1588.92662025518 | 1562.15679576769 | 919.542466211716 | 1534.47690658442 | -0.738 | 2.02636E-19 |
| ENSG00000112297 | **CRYBG1** | 163.579173353429 | 147.286155907914 | 164.721114608584 | 427.271206774872 | 392.558341474809 | 376.093556930444 | 158.528814623309 | 398.641035060042 | -1.331 | 2.19326E-19 |
| ENSG00000159176 | **CSRP1** | 750.419457758853 | 701.833360366572 | 543.890472764191 | 1524.49616978666 | 1344.9304581355 | 1235.73597277146 | 665.381096963205 | 1368.38753356454 | -1.040 | 5.68746E-19 |
| ENSG00000170485 | **NPAS2** | 7.1565888342125 | 18.7814561224857 | 6.21589111730505 | 121.809773861257 | 125.933503029513 | 105.4278434522 | 10.7179786913344 | 117.72370678099 | -3.448 | 7.44856E-19 |
| ENSG00000151364 | **KCTD14** | 32.7158346706857 | 22.7354468851143 | 25.8995463221044 | 147.10872689398 | 138.723624430947 | 166.251599290008 | 27.1169426259681 | 150.694650204978 | -2.475 | 7.69007E-19 |
| ENSG00000132849 | **PATJ** | 968.184232285605 | 1020.12961675817 | 1078.45710885243 | 1620.06999235472 | 1657.79650472444 | 1673.66701480368 | 1022.2569859654 | 1650.51117062761 | -0.691 | 9.47648E-19 |
| ENSG00000156928 | **MALSU1** | 1421.09406850791 | 1324.58690548057 | 1361.28015468981 | 2049.21519565054 | 2125.12786362302 | 2204.86114912053 | 1368.98704289276 | 2126.40140279803 | -0.635 | 1.24502E-18 |
| ENSG00000054793 | **ATP9A** | 465.178274223812 | 528.846264501572 | 492.091380119983 | 951.990232638749 | 853.986567418882 | 984.331115308521 | 495.371972948456 | 930.102638455384 | -0.909 | 1.57293E-18 |
| ENSG00000048740 | **CELF2** | 2.04473966691786 | 1.97699538131429 | 1.03598185288417 | 139.61274081021 | 120.030370075004 | 151.045660330556 | 1.68590563370544 | 136.896257071923 | -6.338 | 2.02784E-18 |
| ENSG00000231298 | **LINC00704** | 4.08947933383571 | 5.93098614394286 | 5.17990926442087 | 110.565794735603 | 97.4016937493886 | 83.1257996450039 | 5.06679158073315 | 97.0310960433318 | -4.258 | 2.81299E-18 |
| ENSG00000164855 | **TMEM184A** | 637.958776078371 | 617.811056660714 | 607.085365790126 | 1188.11379427749 | 1019.27429014512 | 1096.85506360847 | 620.951732843071 | 1101.41438267703 | -0.827 | 3.06982E-18 |
| ENSG00000132170 | **PPARG** | 1660.3286095373 | 1732.83645172197 | 1598.51999900028 | 2435.25847896467 | 2562.94355774906 | 2528.24078432487 | 1663.89502008652 | 2508.81427367954 | -0.592 | 5.18091E-18 |
| ENSG00000149573 | **MPZL2** | 4.08947933383571 | 0 | 4.1439274115367 | 115.250786037959 | 100.353260226643 | 95.2905508125654 | 2.74446891512414 | 103.631532359056 | -5.257 | 6.83010E-18 |
| ENSG00000119408 | **NEK6** | 287.285923201959 | 247.124422664286 | 219.628152811445 | 567.820945845553 | 522.427266473994 | 558.564824443867 | 251.346166225897 | 549.604345587805 | -1.129 | 1.89335E-17 |
| ENSG00000126822 | **PLEKHG3** | 941.602616615673 | 852.085009346457 | 1042.19774400148 | 1541.36213847514 | 1584.00734279309 | 1709.1475390424 | 945.295123321203 | 1611.50567343687 | -0.770 | 2.54005E-17 |
| ENSG00000183251 | **OR51B4** | 14.313177668425 | 9.88497690657143 | 24.8635644692202 | 123.6837703822 | 122.981936552258 | 117.592594619762 | 16.3539063480722 | 121.419433851407 | -2.898 | 3.21341E-17 |
| ENSG00000111859 | **NEDD9** | 2.04473966691786 | 4.94248845328572 | 1.03598185288417 | 98.384817349477 | 84.6115723479538 | 114.551406827871 | 2.67440332436258 | 99.1825988417673 | -5.197 | 3.33741E-17 |
| ENSG00000135678 | **CPM** | 17.3802871688018 | 17.7929584318286 | 18.6476733519151 | 120.872775600786 | 104.288682196315 | 130.771075051287 | 17.9403063175152 | 118.644177616129 | -2.725 | 4.93472E-17 |
| ENSG00000025708 | **TYMP** | 98.1475040120571 | 96.8727736844 | 76.6626571134289 | 304.524434653143 | 289.253514770912 | 235.185189239523 | 90.560978269962 | 276.321046221193 | -1.609 | 9.51568E-17 |
| ENSG00000106688 | **SLC1A1** | 751.441827592312 | 922.268345383114 | 675.460168080482 | 1479.52025328404 | 1552.52396703571 | 1357.38348444708 | 783.056780351969 | 1463.14256825561 | -0.901 | 1.02992E-16 |
| ENSG00000152056 | **AP1S3** | 353.739962376789 | 368.709638615114 | 422.680595976743 | 682.134733623041 | 773.310417040601 | 767.393052820341 | 381.710065656216 | 740.946067827994 | -0.957 | 1.07817E-16 |
| ENSG00000188158 | **NHS** | 406.903193716653 | 513.030301451057 | 584.293765026674 | 962.297213503932 | 1051.74152139491 | 982.303656780595 | 501.409086731462 | 998.78079722648 | -0.994 | 1.35563E-16 |
| ENSG00000148737 | **TCF7L2** | 929.334178614166 | 950.934778412172 | 971.750978005355 | 1438.29232982331 | 1508.2504698769 | 1598.65104927038 | 950.673311677231 | 1515.06461632353 | -0.672 | 1.35841E-16 |
| ENSG00000126217 | **MCF2L** | 324.09123720648 | 328.181233298171 | 291.110900660453 | 663.394768413616 | 606.054983329529 | 591.004160890698 | 314.461123721702 | 620.151304211281 | -0.980 | 1.80661E-16 |
| ENSG00000171219 | **CDC42BPG** | 865.947248939712 | 879.762944684857 | 698.251768843934 | 1437.35533156284 | 1357.72057953693 | 1465.85251569117 | 814.653987489501 | 1420.30947559698 | -0.801 | 2.77667E-16 |
| ENSG00000122786 | **CALD1** | 3.06710950037678 | 3.95399076262857 | 0 | 108.69179821466 | 95.4339827645525 | 78.0571533251866 | 2.34036675433512 | 94.0609781014665 | -5.314 | 5.52857E-16 |
| ENSG00000148356 | **LRSAM1** | 408.947933383571 | 457.674430774257 | 385.385249272913 | 749.598608376968 | 796.922948858634 | 754.214572388816 | 417.33587114358 | 766.912043208139 | -0.877 | 8.65560E-16 |
| ENSG00000165757 | **JCAD** | 243.324020363225 | 187.814561224857 | 282.82304583738 | 667.142761455501 | 528.330399428502 | 521.056841677219 | 237.987209141821 | 572.176667520408 | -1.267 | 1.06028E-15 |
| ENSG00000249853 | **HS3ST5** | 12.2684380015071 | 7.90798152525714 | 21.7556189105677 | 144.297732112566 | 105.272537688733 | 94.276821548602 | 13.9773461457773 | 114.615697116634 | -3.043 | 1.21852E-15 |
| ENSG00000144485 | **HES6** | 1127.6739263052 | 1080.42797588826 | 907.520103126537 | 2006.11327566886 | 1803.40711760232 | 1628.04919792532 | 1038.54066844 | 1812.5231970655 | -0.804 | 1.28573E-15 |
| ENSG00000267795 | **SMIM22** | 1.02236983345893 | 0 | 2.07196370576835 | 193.021641657069 | 142.65904640062 | 150.031931066592 | 1.03144451307576 | 161.90420637476 | -7.317 | 1.29247E-15 |
| ENSG00000271843 | **AC012557.1** | 7.1565888342125 | 4.94248845328572 | 6.21589111730505 | 82.4558469214665 | 82.6438613631176 | 83.1257996450039 | 6.10498946826775 | 82.7418359765293 | -3.763 | 1.40790E-15 |
| ENSG00000175274 | **TP53I11** | 598.086352573473 | 620.776549732686 | 558.39421870457 | 1079.42199606283 | 1106.83742897033 | 932.630922846385 | 592.41904033691 | 1039.63011595985 | -0.811 | 1.52488E-15 |
| ENSG00000125798 | **FOXA2** | 5.11184916729464 | 10.8734745972286 | 4.1439274115367 | 84.3298434424089 | 82.6438613631176 | 90.2219044927481 | 6.70975039201997 | 85.7318697660915 | -3.666 | 2.02684E-15 |
| ENSG00000003436 | **TFPI** | 74.6329978425018 | 53.3788752954857 | 45.5832015269037 | 216.446598168849 | 192.835676513941 | 201.732123528729 | 57.8650248882971 | 203.671466070507 | -1.816 | 2.56888E-15 |
| ENSG00000173210 | **ABLIM3** | 7.1565888342125 | 18.7814561224857 | 7.25187297018922 | 137.738744289268 | 91.4985607948802 | 90.2219044927481 | 11.0633059756291 | 106.486403192299 | -3.260 | 2.70053E-15 |
| ENSG00000138134 | **STAMBPL1** | 1367.93083716805 | 1186.19722878857 | 1345.74042689654 | 1902.10646875656 | 2135.95027403962 | 2224.12200513584 | 1299.95616428439 | 2087.39291597734 | -0.683 | 2.85120E-15 |
| ENSG00000140284 | **SLC27A2** | 468.245383724189 | 464.593914608857 | 413.356759300786 | 775.834559670162 | 782.165116472363 | 885.999376704066 | 448.732019211277 | 814.666350948864 | -0.860 | 3.11401E-15 |
| ENSG00000105855 | **ITGB8** | 47.0290123391107 | 61.2868568207429 | 82.8785482307339 | 195.832636438483 | 254.818572536279 | 219.979250280071 | 63.7314724635292 | 223.543486418278 | -1.811 | 5.82174E-15 |
| ENSG00000165474 | **GJB2** | 299.554361203466 | 247.124422664286 | 320.11839254121 | 595.930893659689 | 570.636185602479 | 567.688387819539 | 288.932392136321 | 578.085155693902 | -1.002 | 6.72443E-15 |
| ENSG00000130762 | **ARHGEF16** | 1016.23561445817 | 946.980787649543 | 975.894905416892 | 1621.00699061519 | 1551.54011154329 | 1432.39944998037 | 979.70376917487 | 1534.98218404629 | -0.648 | 9.01684E-15 |
| ENSG00000255874 | **LINC00346** | 125.751489515448 | 168.044607411714 | 123.281840493217 | 322.327401602096 | 356.15568825534 | 356.832700915139 | 139.025979140126 | 345.105263590858 | -1.310 | 1.01748E-14 |
| ENSG00000167165 | **UGT1A6** | 0 | 0 | 0 | 136.801746028797 | 168.239289203489 | 204.773311320619 | 0 | 169.938115517635 | -9.828 | 1.30383E-14 |
| ENSG00000174307 | **PHLDA3** | 77.7001073428786 | 50.4133822235143 | 73.5547115547764 | 276.414486839007 | 202.674231438122 | 205.787040584583 | 67.2227337070564 | 228.29191962057 | -1.767 | 1.41160E-14 |
| ENSG00000184254 | **ALDH1A3** | 2206.27410060437 | 2366.4634714332 | 2554.73124921237 | 3381.6267220406 | 3707.16749543128 | 4266.78647202221 | 2375.82294041665 | 3785.19356316469 | -0.672 | 1.51216E-14 |
| ENSG00000164078 | **MST1R** | 1219.6872113165 | 1338.42587314977 | 1397.53951954075 | 1924.59442700786 | 2009.03291551769 | 2056.85667658186 | 1318.55086800234 | 1996.82800636914 | -0.599 | 2.12657E-14 |
| ENSG00000172818 | **OVOL1** | 27.6039855033911 | 26.6894376477429 | 37.2953467038303 | 137.738744289268 | 144.626757385456 | 120.633782411652 | 30.5295899516547 | 134.333094695459 | -2.140 | 4.77260E-14 |
| ENSG00000164548 | **TRA2A** | 3586.47337577392 | 4150.70180306934 | 3923.26327687237 | 5312.78013687176 | 6544.60673556498 | 5933.35738197814 | 3886.81281857188 | 5930.24808480496 | -0.609 | 4.77260E-14 |
| ENSG00000188120 | **DAZ1** | 270.928005866616 | 338.066210204743 | 282.82304583738 | 569.694942366496 | 552.926786738954 | 643.718082616798 | 297.272420636246 | 588.779937240749 | -0.985 | 5.30453E-14 |
| ENSG00000135338 | **LCA5** | 155.400214685757 | 201.653528894057 | 209.268334282603 | 433.83019459817 | 386.6552085203 | 442.999688352033 | 188.774025954139 | 421.161697156834 | -1.158 | 5.50760E-14 |
| ENSG00000071242 | **RPS6KA2** | 27.6039855033911 | 44.4823960795714 | 53.8710563499771 | 180.840664270943 | 151.513745832382 | 167.265328553971 | 41.9858126443132 | 166.539912885766 | -1.989 | 6.49730E-14 |
| ENSG00000180875 | **GREM2** | 3.06710950037678 | 3.95399076262857 | 0 | 79.6448521400528 | 77.7245839010273 | 72.9885070053693 | 2.34036675433512 | 76.7859810154831 | -5.021 | 6.72337E-14 |
| ENSG00000104881 | **PPP1R13L** | 736.106280090428 | 777.947682547172 | 606.049383937242 | 1223.7197281754 | 1202.27141173488 | 1168.82984134987 | 706.701115524947 | 1198.27366042005 | -0.761 | 7.20256E-14 |
| ENSG00000109756 | **RAPGEF2** | 679.875939250187 | 787.832659453743 | 792.526117456393 | 1173.12182210995 | 1256.38346381787 | 1226.61240939579 | 753.411572053441 | 1218.7058984412 | -0.694 | 8.36767E-14 |
| ENSG00000164379 | **FOXQ1** | 4.08947933383571 | 0.988497690657143 | 8.2878548230734 | 63.7158817120423 | 74.7730174237731 | 86.1669874368943 | 4.45527728252208 | 74.8852955242365 | -4.084 | 1.39954E-13 |
| ENSG00000129514 | **FOXA1** | 2880.0158208538 | 2971.42405811537 | 3591.74908394943 | 4584.73248848563 | 4860.24613254525 | 5538.00296903239 | 3147.7296543062 | 4994.32719668776 | -0.666 | 1.47666E-13 |
| ENSG00000261150 | **EPPK1** | 387.478166880934 | 388.479592428257 | 379.169358155608 | 829.243460517021 | 687.714989200229 | 643.718082616798 | 385.042372488266 | 720.225510778016 | -0.904 | 1.56641E-13 |
| ENSG00000132031 | **MATN3** | 67.4764090082893 | 50.4133822235143 | 46.6191833797878 | 202.391624261781 | 171.190855680744 | 169.292787081898 | 54.8363248705305 | 180.958422341474 | -1.723 | 4.16171E-13 |
| ENSG00000170955 | **CAVIN3** | 178.914720855312 | 192.757049678143 | 108.778094552838 | 537.837001510474 | 397.477618936899 | 376.093556930444 | 160.149955028765 | 437.136059125939 | -1.448 | 4.60841E-13 |
| ENSG00000148180 | **GSN** | 1003.96717645667 | 999.371165254372 | 929.275722037104 | 1561.97610020551 | 1420.68733105169 | 1821.67148734234 | 977.538021249381 | 1601.44497286651 | -0.712 | 4.60841E-13 |
| ENSG00000206077 | **ZDHHC11B** | 23.5145061695554 | 29.6549307197143 | 26.9355281749885 | 99.3218156099482 | 137.739768938529 | 130.771075051287 | 26.7016550214194 | 122.610886533255 | -2.197 | 5.61693E-13 |
| ENSG00000106789 | **CORO2A** | 158.467324186134 | 144.320662835943 | 113.958003817259 | 386.98028157461 | 307.946769126855 | 312.228613300746 | 138.915330279779 | 335.718554667404 | -1.273 | 9.58359E-13 |
| ENSG00000106868 | **SUSD1** | 271.950375700075 | 257.997897261514 | 290.074918807569 | 507.853057175396 | 487.008468746943 | 538.290239164598 | 273.341063923053 | 511.050588362312 | -0.903 | 1.21233E-12 |
| ENSG00000284837 | **AC007846.1** | 0 | 0 | 0 | 144.297732112566 | 84.6115723479538 | 103.400384924273 | 0 | 110.769896461598 | -9.213 | 1.29227E-12 |
| ENSG00000136603 | **SKIL** | 475.401972558402 | 594.087112084943 | 487.947452708446 | 986.659168276184 | 898.260064577695 | 847.477664673454 | 519.145512450597 | 910.798965842444 | -0.811 | 1.29713E-12 |
| ENSG00000009950 | **MLXIPL** | 82.8119565101732 | 79.0798152525714 | 46.6191833797878 | 223.005585992148 | 210.545075377466 | 206.800769848546 | 69.5036517141775 | 213.45047707272 | -1.617 | 1.47358E-12 |
| ENSG00000008300 | **CELSR3** | 526.520464231348 | 625.719038185972 | 577.041892056485 | 957.612222201576 | 909.082474994294 | 963.042800765289 | 576.427131491268 | 943.24583265372 | -0.710 | 1.94774E-12 |
| ENSG00000113319 | **RASGRF2** | 1066.33173629766 | 901.509893879315 | 1073.277199588 | 1631.31397148038 | 1520.05673578591 | 1553.03323239202 | 1013.70627658833 | 1568.13464655277 | -0.630 | 2.12170E-12 |
| ENSG00000102996 | **MMP15** | 547.990230733986 | 617.811056660714 | 488.98343456133 | 940.746253513095 | 915.96946344122 | 918.438713150896 | 551.594907318677 | 925.051476701737 | -0.745 | 2.50032E-12 |
| ENSG00000233893 | **EZR-AS1** | 215.720034859834 | 191.768551987486 | 223.772080222982 | 401.972253742149 | 428.960994694277 | 416.642727488983 | 210.420222356767 | 415.858658641803 | -0.983 | 2.58533E-12 |
| ENSG00000139211 | **AMIGO2** | 3.06710950037678 | 6.9194838346 | 12.4317822346101 | 69.3378712748695 | 68.8698844692647 | 88.1944459648212 | 7.47279185652896 | 75.4674005696518 | -3.339 | 2.69620E-12 |
| ENSG00000187957 | **DNER** | 0 | 0 | 0 | 94.6368243075922 | 103.304826703897 | 95.2905508125654 | 0 | 97.7440672746849 | -9.031 | 3.40994E-12 |
| ENSG00000185483 | **ROR1** | 0 | 4.94248845328572 | 3.10794555865252 | 55.2828973678014 | 59.031329545084 | 90.2219044927481 | 2.68347800397941 | 68.1787104685445 | -4.656 | 3.41315E-12 |
| ENSG00000105339 | **DENND3** | 272.972745533534 | 255.032404189543 | 263.13939063258 | 449.759165026181 | 517.507989011903 | 521.056841677219 | 263.714846785219 | 496.107998571768 | -0.911 | 3.92769E-12 |
| ENSG00000163485 | **ADORA1** | 72.5882581755839 | 70.1833360366572 | 38.3313285567145 | 237.060559899216 | 198.73880946845 | 173.347704137752 | 60.3676409229852 | 203.049024501806 | -1.749 | 4.02175E-12 |
| novel.475 | **-** | 103.259353179352 | 91.9302852311143 | 76.6626571134289 | 278.288483359949 | 233.173751703082 | 210.8556869044 | 90.6174318412983 | 240.77264065581 | -1.410 | 6.17772E-12 |
| ENSG00000170542 | **SERPINB9** | 165.623913020346 | 144.320662835943 | 185.440751666267 | 329.823387685866 | 356.15568825534 | 368.9974520827 | 165.128442507519 | 351.658842674635 | -1.091 | 7.02223E-12 |
| ENSG00000144535 | **DIS3L2** | 506.073067562169 | 532.8002552642 | 583.25778317379 | 848.920423986916 | 875.63138825208 | 901.205315663518 | 540.71036866672 | 875.252375967504 | -0.695 | 7.05346E-12 |
| ENSG00000135423 | **GLS2** | 658.40617274755 | 646.477489689772 | 598.797510967053 | 1059.74503259294 | 994.677902834666 | 950.878049597727 | 634.560391134791 | 1001.76699500844 | -0.659 | 8.30975E-12 |
| ENSG00000251138 | **AC090502.1** | 12.2684380015071 | 25.7009399570857 | 17.611691499031 | 89.9518330052361 | 103.304826703897 | 101.372926396346 | 18.5270231525413 | 98.2098620351598 | -2.402 | 1.16292E-11 |
| ENSG00000242193 | **CRYZL2P** | 9.20132850113035 | 16.8044607411714 | 11.3958003817259 | 69.3378712748695 | 88.546994317626 | 91.2356337567116 | 12.4671965413426 | 83.0401664497357 | -2.731 | 1.17576E-11 |
| ENSG00000120278 | **PLEKHG1** | 79.7448470097964 | 119.608220569514 | 102.562203435533 | 240.808552941101 | 256.786283521116 | 258.500962310683 | 100.638423671615 | 252.0319329243 | -1.323 | 1.39954E-11 |
| ENSG00000120708 | **TGFBI** | 2.04473966691786 | 7.90798152525714 | 3.10794555865252 | 71.2118677958119 | 56.0797630678298 | 61.8374851017712 | 4.35355558360917 | 63.0430386551376 | -3.845 | 1.50558E-11 |
| ENSG00000152766 | **ANKRD22** | 2.04473966691786 | 1.97699538131429 | 2.07196370576835 | 57.1568938887438 | 61.9828960223382 | 66.9061314215885 | 2.03123291800016 | 62.0153071108902 | -4.932 | 1.54501E-11 |
| ENSG00000019582 | **CD74** | 15.3355475018839 | 13.8389676692 | 13.4677640874943 | 101.195812130891 | 75.7568729161912 | 78.0571533251866 | 14.2140930861927 | 85.0032794574228 | -2.581 | 1.64703E-11 |
| ENSG00000074410 | **CA12** | 80.7672168432553 | 74.1373267992857 | 84.9505119365023 | 193.021641657069 | 198.73880946845 | 242.281294087267 | 79.9516851930145 | 211.347248404262 | -1.402 | 1.99829E-11 |
| ENSG00000008735 | **MAPK8IP2** | 788.247141596834 | 742.361765683514 | 698.251768843934 | 1325.85253856676 | 1114.70827290967 | 1120.17083667963 | 742.953558708094 | 1186.91054938535 | -0.676 | 2.11521E-11 |
| ENSG00000228437 | **LINC02474** | 0 | 0 | 0 | 71.2118677958119 | 86.5792833327899 | 82.1120703810404 | 0 | 79.9677405032141 | -8.741 | 2.63227E-11 |
| ENSG00000170231 | **FABP6** | 48.0513821725696 | 47.4478891515429 | 22.7916007634518 | 158.352706019634 | 140.691335415784 | 142.935826218848 | 39.4302906958548 | 147.326622551422 | -1.899 | 3.04249E-11 |
| ENSG00000117480 | **FAAH** | 329.203086373775 | 367.721140924457 | 335.658120334472 | 644.654803204192 | 604.087272344693 | 555.523636651977 | 344.194115877568 | 601.421904066954 | -0.805 | 3.21043E-11 |
| ENSG00000167771 | **RCOR2** | 39.8724235048982 | 39.5399076262857 | 33.1514192922936 | 121.809773861257 | 136.755913446111 | 130.771075051287 | 37.5212501411592 | 129.778920786218 | -1.789 | 4.30269E-11 |
| ENSG00000105514 | **RAB3D** | 179.937090688771 | 147.286155907914 | 137.785586433595 | 362.618326802358 | 319.753035035872 | 315.269801092637 | 155.002944343427 | 332.547054310289 | -1.102 | 4.45254E-11 |
| ENSG00000162105 | **SHANK2** | 15.3355475018839 | 13.8389676692 | 19.6836552047993 | 91.8258295261786 | 85.5954278403718 | 81.098341117077 | 16.2860567919611 | 86.1731994945424 | -2.406 | 5.28013E-11 |
| ENSG00000105289 | **TJP3** | 34.7605743376036 | 44.4823960795714 | 25.8995463221044 | 141.486737331153 | 156.433023294473 | 106.441572716163 | 35.0475055797598 | 134.78711111393 | -1.941 | 6.42103E-11 |
| ENSG00000184292 | **TACSTD2** | 640.003515745289 | 489.306356875286 | 680.640077344903 | 1067.24101867671 | 950.404405675853 | 1189.10442662914 | 603.316649988492 | 1068.9166169939 | -0.826 | 7.41554E-11 |
| ENSG00000140022 | **STON2** | 126.773859348907 | 131.4701928574 | 135.713622727827 | 281.099478141363 | 278.431104354313 | 275.734359798062 | 131.319224978045 | 278.421647431246 | -1.084 | 1.11833E-10 |
| ENSG00000068615 | **REEP1** | 8.17895866767143 | 1.97699538131429 | 1.03598185288417 | 59.9678886701574 | 57.0636185602479 | 57.7825680459173 | 3.7306453006233 | 58.2713584254409 | -3.968 | 1.19808E-10 |
| ENSG00000118898 | **PPL** | 323.068867373021 | 528.846264501572 | 386.421231125797 | 779.582552712047 | 751.665596207403 | 905.260232719371 | 412.778787666797 | 812.169460546274 | -0.975 | 1.63819E-10 |
| ENSG00000120833 | **SOCS2** | 340.449154541823 | 352.8936755646 | 386.421231125797 | 613.733860608642 | 576.539318556987 | 626.484685129419 | 359.921353744073 | 605.585954765016 | -0.751 | 1.89589E-10 |
| ENSG00000169067 | **ACTBL2** | 12.2684380015071 | 15.8159630505143 | 20.7196370576835 | 94.6368243075922 | 80.6761503782815 | 79.07088258915 | 16.268012703235 | 84.7946190916746 | -2.384 | 1.96001E-10 |
| ENSG00000181449 | **SOX2** | 302.621470703843 | 371.675131687086 | 354.305793686388 | 556.576966719899 | 635.570648102071 | 604.182641322223 | 342.867465359105 | 598.776752048064 | -0.804 | 2.01734E-10 |
| ENSG00000167315 | **ACAA2** | 154.377844852298 | 219.446487325886 | 127.425767904753 | 390.728274616494 | 387.639064012718 | 353.791513123248 | 167.083366694312 | 377.386283917487 | -1.174 | 2.26497E-10 |
| ENSG00000082482 | **KCNK2** | 0 | 0 | 0 | 65.5898782329847 | 66.9021734844286 | 56.7688387819539 | 0 | 63.086963499789 | -8.400 | 2.70869E-10 |
| ENSG00000233532 | **LINC00460** | 197.317377857573 | 217.469491944571 | 187.512715372036 | 395.413265918851 | 371.897376134029 | 371.024910610627 | 200.766528391393 | 379.445184221169 | -0.918 | 3.51453E-10 |
| ENSG00000135111 | **TBX3** | 516.296765896759 | 518.961287595 | 520.062890147856 | 754.283599679324 | 838.244879540193 | 843.4227476176 | 518.440314546538 | 811.983742279039 | -0.647 | 3.81260E-10 |
| ENSG00000075223 | **SEMA3C** | 150.288365518462 | 107.746248281629 | 226.880025781634 | 661.520771892674 | 634.586792609653 | 744.077279749181 | 161.638213193908 | 680.061614750503 | -2.074 | 4.00067E-10 |
| novel.174 | **-** | 475.401972558402 | 444.823960795714 | 535.602617941118 | 838.613443121733 | 748.714029730149 | 774.489157668085 | 485.276183765078 | 787.272210173322 | -0.699 | 4.45918E-10 |
| ENSG00000004139 | **SARM1** | 212.652925359457 | 186.8260635342 | 149.181386815321 | 371.051311146599 | 381.73593105821 | 353.791513123248 | 182.886791902993 | 368.859585109352 | -1.012 | 4.70292E-10 |
| ENSG00000108797 | **CNTNAP1** | 230.033212528259 | 235.2624503764 | 209.268334282603 | 425.397210253929 | 414.203162308006 | 393.326954417823 | 224.854665729087 | 410.97577565992 | -0.870 | 4.87861E-10 |
| ENSG00000188112 | **C6orf132** | 316.934648372268 | 201.653528894057 | 289.038936954685 | 537.837001510474 | 494.879312686288 | 561.606012235758 | 269.20903807367 | 531.440775477507 | -0.982 | 5.13589E-10 |
| ENSG00000126016 | **AMOT** | 107.348832513187 | 141.355169763971 | 99.4542578768807 | 268.918500755237 | 265.640982952878 | 252.418586726902 | 116.05275338468 | 262.326023478339 | -1.175 | 5.82056E-10 |
| ENSG00000189120 | **SP6** | 140.064667183873 | 95.8842759937429 | 99.4542578768807 | 252.989530327227 | 276.463393369477 | 249.377398935012 | 111.801067018166 | 259.610107543905 | -1.216 | 5.83280E-10 |
| novel.473 | **-** | 67.4764090082893 | 72.1603314179714 | 74.5906934076606 | 184.588657312828 | 171.190855680744 | 186.526184569277 | 71.4091446113071 | 180.768565854283 | -1.340 | 5.91191E-10 |
| ENSG00000174705 | **SH3PXD2B** | 1210.48588281537 | 1336.44887776846 | 1056.70148994186 | 1748.43875403928 | 1941.14688654085 | 1739.5594169613 | 1201.21208350856 | 1809.71501918047 | -0.591 | 6.19400E-10 |
| ENSG00000143502 | **SUSD4** | 30.6710950037678 | 25.7009399570857 | 32.1154374394094 | 123.6837703822 | 98.3855492418067 | 104.414114188237 | 29.495824133421 | 108.827811270748 | -1.886 | 7.49619E-10 |
| ENSG00000102890 | **ELMO3** | 366.008400378296 | 413.192034694686 | 336.694102187357 | 709.307683176706 | 627.699804162727 | 585.935514570881 | 371.964845753446 | 640.981000636771 | -0.785 | 7.86055E-10 |
| ENSG00000206075 | **SERPINB5** | 260.704307532027 | 217.469491944571 | 268.319299897001 | 467.562131975134 | 430.928705679113 | 452.123251727704 | 248.831033124533 | 450.20469646065 | -0.856 | 8.98927E-10 |
| ENSG00000151150 | **ANK3** | 99.169873845516 | 181.883575080914 | 140.893531992248 | 312.020420736913 | 314.833757573781 | 357.846430179102 | 140.648993639559 | 328.233536163265 | -1.221 | 9.95449E-10 |
| ENSG00000115525 | **ST3GAL5** | 129.840968849284 | 109.723243662943 | 103.598185288417 | 243.619547722515 | 272.527971399805 | 239.240106295377 | 114.387465933548 | 251.795875139232 | -1.138 | 1.02689E-09 |
| ENSG00000169129 | **AFAP1L2** | 0 | 0 | 0 | 49.6609078049741 | 44.273497158813 | 72.9885070053693 | 0 | 55.6409706563855 | -8.218 | 1.12142E-09 |
| ENSG00000197321 | **SVIL** | 837.320893602862 | 805.625617885572 | 1020.44212509091 | 1311.79756465969 | 1345.91431362792 | 1420.23469881281 | 887.796212193115 | 1359.31552570014 | -0.615 | 1.32039E-09 |
| ENSG00000138735 | **PDE5A** | 114.5054213474 | 159.1481281958 | 112.922021964375 | 274.540490318064 | 324.672312497962 | 263.5696086305 | 128.858523835858 | 287.594137148842 | -1.157 | 1.62692E-09 |
| ENSG00000227036 | **LINC00511** | 20.4473966691786 | 22.7354468851143 | 24.8635644692202 | 96.5108208285346 | 89.5308498100441 | 89.2081752287846 | 22.6821360078377 | 91.7499486224545 | -2.017 | 1.64257E-09 |
| ENSG00000163082 | **SGPP2** | 479.491451892237 | 479.421379968714 | 543.890472764191 | 796.448521400528 | 770.358850563347 | 766.379323556377 | 500.934434875048 | 777.728898506751 | -0.635 | 2.24483E-09 |
| ENSG00000010030 | **ETV7** | 49.0737520060286 | 43.4938983889143 | 51.7990926442087 | 142.423735591624 | 128.885069506767 | 143.949555482812 | 48.1222476797172 | 138.419453527067 | -1.525 | 2.37880E-09 |
| ENSG00000115598 | **IL1RL2** | 20.4473966691786 | 12.8504699785429 | 14.5037459403784 | 74.0228625772256 | 80.6761503782815 | 78.0571533251866 | 15.9338708627 | 77.5853887602312 | -2.285 | 2.85997E-09 |
| ENSG00000136231 | **IGF2BP3** | 3664.1734831168 | 4165.5292684292 | 2467.7087735701 | 8763.74473018723 | 9651.62238062124 | 8778.89542592358 | 3432.47050837203 | 9064.75417891068 | -1.401 | 2.91197E-09 |
| ENSG00000185215 | **TNFAIP2** | 110.415942013564 | 73.1488291086286 | 72.5187297018922 | 210.824608606022 | 200.706520453286 | 218.965521016108 | 85.3611669413617 | 210.165550025139 | -1.300 | 3.56390E-09 |
| ENSG00000253368 | **TRNP1** | 133.93044818312 | 127.516202094771 | 106.70613084707 | 276.414486839007 | 248.915439581771 | 250.391128198975 | 122.71759370832 | 258.573684873251 | -1.075 | 3.97110E-09 |
| ENSG00000169116 | **PARM1** | 49.0737520060286 | 61.2868568207429 | 46.6191833797878 | 185.5256555733 | 126.917358521931 | 145.977014010739 | 52.3265974021864 | 152.806676035323 | -1.546 | 4.51194E-09 |
| ENSG00000111700 | **SLCO1B3** | 377.254468546345 | 458.662928464914 | 391.601140390218 | 608.111871045815 | 756.584873669494 | 701.500650662716 | 409.172845800492 | 688.732465126008 | -0.750 | 4.51194E-09 |
| ENSG00000253313 | **C1orf210** | 7.1565888342125 | 10.8734745972286 | 2.07196370576835 | 59.9678886701574 | 58.047474052666 | 54.7413802540269 | 6.70067571240314 | 57.5855809922834 | -3.094 | 4.65957E-09 |
| ENSG00000198910 | **L1CAM** | 477.44671222532 | 406.272550860086 | 413.356759300786 | 770.212570107334 | 655.247757950433 | 684.267253175337 | 432.35867412873 | 703.242527077701 | -0.702 | 4.78221E-09 |
| ENSG00000138119 | **MYOF** | 8444.77482437075 | 8451.65525511857 | 11261.122740851 | 17891.9817836977 | 20829.2046299829 | 22943.734431285 | 9385.85094011343 | 20554.9736149886 | -1.131 | 4.79228E-09 |
| ENSG00000110002 | **VWA5A** | 101.214613512434 | 97.8612713750572 | 91.1664030538073 | 223.005585992148 | 223.335196778901 | 196.663477208912 | 96.7474293137661 | 214.334753326654 | -1.148 | 6.27812E-09 |
| ENSG00000124788 | **ATXN1** | 325.113607039939 | 388.479592428257 | 355.341775539272 | 610.922865827229 | 564.733052647971 | 576.81195119521 | 356.311658335823 | 584.155956556803 | -0.713 | 6.37358E-09 |
| ENSG00000153933 | **DGKE** | 428.372960219291 | 523.903776048286 | 440.292287475774 | 724.299655344245 | 753.633307192239 | 732.926257845583 | 464.189674581117 | 736.953073460689 | -0.666 | 6.54310E-09 |
| ENSG00000064270 | **ATP2C2** | 5.11184916729464 | 1.97699538131429 | 4.1439274115367 | 39.3539269397908 | 59.031329545084 | 46.6315461423192 | 3.74425732004854 | 48.3389342090647 | -3.696 | 6.91971E-09 |
| ENSG00000142910 | **TINAGL1** | 83.8343263436321 | 27.6779353384 | 70.4467659961239 | 191.147645136127 | 219.399774809229 | 182.471267513423 | 60.653009226052 | 197.672895819593 | -1.707 | 7.40036E-09 |
| ENSG00000241111 | **PRICKLE2-AS1** | 0 | 0 | 1.03598185288417 | 77.7708556191104 | 82.6438613631176 | 67.9198606855519 | 0.345327284294725 | 76.11152588926 | -7.709 | 7.78353E-09 |
| ENSG00000135638 | **EMX1** | 114.5054213474 | 121.585215950829 | 107.742112699954 | 246.430542503928 | 260.721705490788 | 218.965521016108 | 114.610916666061 | 242.039256336941 | -1.078 | 8.44181E-09 |
| ENSG00000267308 | **LINC01764** | 0 | 0 | 0 | 47.7869112840317 | 41.3219306815588 | 42.5766290864654 | 0 | 43.895157017352 | -7.877 | 8.55527E-09 |
| ENSG00000253377 | **AC068672.2** | 0 | 0 | 0 | 46.8499130235605 | 38.3703642043046 | 46.6315461423192 | 0 | 43.9506077900614 | -7.879 | 8.62707E-09 |
| ENSG00000127324 | **TSPAN8** | 0 | 4.94248845328572 | 8.2878548230734 | 39.3539269397908 | 55.0959075754118 | 64.8786728936616 | 4.41011442545304 | 53.1095024696214 | -3.591 | 1.01354E-08 |
| ENSG00000091592 | **NLRP1** | 57.2527106737 | 79.0798152525714 | 92.2023849066915 | 198.643631219896 | 198.73880946845 | 172.333974873789 | 76.1783036109877 | 189.905471854045 | -1.319 | 1.12988E-08 |
| ENSG00000148444 | **COMMD3** | 300.576731036925 | 281.721841837286 | 304.578664747947 | 491.924086747385 | 476.186058330345 | 495.713610078133 | 295.625745874053 | 487.941251718621 | -0.723 | 1.14220E-08 |
| ENSG00000130508 | **PXDN** | 0 | 0 | 0 | 23.4249565117802 | 51.1604856057395 | 59.8100265738442 | 0 | 44.798489563788 | -7.904 | 1.30062E-08 |
| ENSG00000166016 | **ABTB2** | 131.885708516202 | 87.9762944684857 | 102.562203435533 | 229.564573815446 | 225.302907763737 | 268.638254950317 | 107.474735473407 | 241.168578843167 | -1.166 | 1.35276E-08 |
| ENSG00000196092 | **PAX5** | 97.1251341785982 | 94.8957783030857 | 81.8425663778498 | 205.202619043195 | 197.754953976031 | 202.745852792692 | 91.2878262865112 | 201.901141937306 | -1.145 | 1.67984E-08 |
| ENSG00000105219 | **CNTD2** | 12.2684380015071 | 3.95399076262857 | 6.21589111730505 | 48.7239095445029 | 68.8698844692647 | 53.7276509900635 | 7.47943996048025 | 57.1071483346104 | -2.936 | 1.81771E-08 |
| ENSG00000133216 | **EPHB2** | 23.5145061695554 | 16.8044607411714 | 18.6476733519151 | 98.384817349477 | 65.9183179920105 | 86.1669874368943 | 19.655546754214 | 83.4900409261273 | -2.088 | 2.04913E-08 |
| ENSG00000148344 | **PTGES** | 1.02236983345893 | 1.97699538131429 | 0 | 66.5268764934559 | 52.1443410981576 | 37.5079827666481 | 0.999788404924405 | 52.0597334527538 | -5.684 | 2.21668E-08 |
| ENSG00000145911 | **N4BP3** | 393.612385881687 | 344.985694039343 | 337.730084040241 | 546.269985854715 | 602.119561359857 | 588.976702362771 | 358.776054653757 | 579.122083192448 | -0.691 | 2.35586E-08 |
| ENSG00000115590 | **IL1R2** | 0 | 1.97699538131429 | 3.10794555865252 | 43.1019199816756 | 38.3703642043046 | 50.6864631981731 | 1.69498031332227 | 44.0529157947178 | -4.702 | 2.38857E-08 |
| ENSG00000142623 | **PADI1** | 4.08947933383571 | 7.90798152525714 | 2.07196370576835 | 66.5268764934559 | 83.6277168555357 | 23.3157730711596 | 4.68980818828707 | 57.8234554733837 | -3.616 | 2.86043E-08 |
| ENSG00000084628 | **NKAIN1** | 13.2908078349661 | 17.7929584318286 | 18.6476733519151 | 75.896859098168 | 78.7084393934454 | 65.892402157625 | 16.5771465395699 | 73.4992335497461 | -2.149 | 3.37350E-08 |
| ENSG00000115290 | **GRB14** | 148.243625851545 | 159.1481281958 | 147.109423109553 | 289.532462485604 | 305.979058142019 | 268.638254950317 | 151.500392385632 | 288.049925192647 | -0.927 | 3.40970E-08 |
| ENSG00000187595 | **ZNF385C** | 47.0290123391107 | 30.6434284103714 | 26.9355281749885 | 108.69179821466 | 111.175670643242 | 117.592594619762 | 34.8693229748236 | 112.486687825888 | -1.690 | 3.45662E-08 |
| ENSG00000134058 | **CDK7** | 94.0580246782214 | 88.9647921591429 | 111.886040111491 | 361.681328541887 | 263.673271968042 | 164.224140762081 | 98.302952316285 | 263.192913757337 | -1.423 | 3.78290E-08 |
| ENSG00000179314 | **WSCD1** | 0 | 0.988497690657143 | 0 | 65.5898782329847 | 65.9183179920105 | 57.7825680459173 | 0.329499230219048 | 63.0969214236375 | -7.439 | 3.79554E-08 |
| ENSG00000171766 | **GATM** | 891.506494776185 | 1049.78454747789 | 663.028385845872 | 1467.33927589791 | 1485.62179355128 | 1339.13635769573 | 868.106476033314 | 1430.69914238164 | -0.720 | 4.13857E-08 |
| ENSG00000101460 | **MAP1LC3A** | 360.896551211002 | 336.089214823429 | 347.053920716198 | 588.43490757592 | 552.926786738954 | 521.056841677219 | 348.013228916876 | 554.139511997364 | -0.672 | 4.33998E-08 |
| ENSG00000069812 | **HES2** | 104.281723012811 | 94.8957783030857 | 84.9505119365023 | 202.391624261781 | 201.690375945704 | 207.81449911251 | 94.7093377507996 | 203.965499773332 | -1.106 | 4.53153E-08 |
| ENSG00000188153 | **COL4A5** | 0 | 0 | 0 | 27.1729495536651 | 42.3057861739769 | 41.5628998225019 | 0 | 37.0138785167146 | -7.629 | 4.75884E-08 |
| ENSG00000115318 | **LOXL3** | 141.087037017332 | 110.7117413536 | 147.109423109553 | 263.29651119241 | 253.834717043861 | 280.803006117879 | 132.969400493495 | 265.97807811805 | -1.001 | 5.07448E-08 |
| ENSG00000166741 | **NNMT** | 1.02236983345893 | 0 | 1.03598185288417 | 70.2748695353407 | 44.273497158813 | 70.9610484774423 | 0.686117228781034 | 61.8364717238654 | -6.513 | 5.08341E-08 |
| ENSG00000111863 | **ADTRP** | 0 | 0 | 0 | 59.0308904096862 | 30.4995202649601 | 24.3295023351231 | 0 | 37.9533043365898 | -7.669 | 5.63836E-08 |
| ENSG00000137462 | **TLR2** | 64.4092995079125 | 35.5859168636572 | 83.9145300836181 | 190.210646875656 | 150.529890339964 | 185.512455305314 | 61.3032488183959 | 175.417664173644 | -1.520 | 5.80307E-08 |
| ENSG00000153234 | **NR4A2** | 219.80951419367 | 241.193436520343 | 222.736098370097 | 390.728274616494 | 362.058821209849 | 446.040876143923 | 227.91301636137 | 399.609323990089 | -0.809 | 6.24592E-08 |
| ENSG00000197183 | **NOL4L** | 126.773859348907 | 113.677234425571 | 102.562203435533 | 273.603492057593 | 211.528930869884 | 234.17145997556 | 114.337765736671 | 239.767960967679 | -1.069 | 6.24919E-08 |
| ENSG00000204128 | **C2orf72** | 11.2460681680482 | 13.8389676692 | 22.7916007634518 | 71.2118677958119 | 80.6761503782815 | 66.9061314215885 | 15.9588788669 | 72.9313831985606 | -2.195 | 6.37627E-08 |
| ENSG00000225683 | **PACRG-AS3** | 0 | 0 | 0 | 35.605933897906 | 35.4187977270504 | 34.4667949747577 | 0 | 35.1638421999047 | -7.557 | 6.41759E-08 |
| ENSG00000163739 | **CXCL1** | 64.4092995079125 | 34.597419173 | 105.670148994186 | 428.208205035343 | 370.913520641611 | 514.974466093439 | 68.2256225583661 | 438.032063923464 | -2.684 | 6.89274E-08 |
| ENSG00000253293 | **HOXA10** | 491.759889893744 | 564.432181365229 | 479.659597885373 | 703.685693613878 | 841.196446017447 | 864.711062160833 | 511.950556381449 | 803.19773393072 | -0.649 | 7.46695E-08 |
| ENSG00000154914 | **USP43** | 122.684380015071 | 157.171132814486 | 142.965495698016 | 265.170507713352 | 266.624838445296 | 286.88538170166 | 140.940336175858 | 272.893575953436 | -0.952 | 8.32092E-08 |
| ENSG00000165895 | **ARHGAP42** | 295.46488186963 | 304.4572887224 | 263.13939063258 | 443.200177202882 | 505.701723102886 | 477.466483326791 | 287.68718707487 | 475.45612787752 | -0.724 | 8.63867E-08 |
| ENSG00000184557 | **SOCS3** | 233.100322028636 | 218.457989635229 | 268.319299897001 | 371.98830940707 | 424.041717232187 | 525.111758733073 | 239.959203853622 | 440.38059512411 | -0.876 | 9.24397E-08 |
| ENSG00000166126 | **AMN** | 265.816156699321 | 197.699538131429 | 268.319299897001 | 427.271206774872 | 399.445329921735 | 494.699880814169 | 243.944998242584 | 440.472139170259 | -0.853 | 1.03324E-07 |
| ENSG00000241635 | **UGT1A1** | 0 | 0 | 0 | 40.290925200262 | 35.4187977270504 | 25.3432315990865 | 0 | 33.6843181754663 | -7.496 | 1.07585E-07 |
| ENSG00000228707 | **AL691426.1** | 0 | 0 | 0 | 36.5429321583772 | 34.4349422346324 | 27.3706901270135 | 0 | 32.7828548400077 | -7.456 | 1.26808E-07 |
| ENSG00000249631 | **AC005699.1** | 1.02236983345893 | 0.988497690657143 | 5.17990926442087 | 41.2279234607332 | 33.4510867422143 | 45.6178168783558 | 2.39692559617898 | 40.0989423604344 | -4.077 | 1.33660E-07 |
| ENSG00000275410 | **HNF1B** | 22.4921363360964 | 9.88497690657143 | 14.5037459403784 | 93.699826047121 | 71.8214509465189 | 56.7688387819539 | 15.6269530610154 | 74.0967052585312 | -2.250 | 1.34603E-07 |
| ENSG00000156395 | **SORCS3** | 0 | 0 | 0 | 37.4799304188484 | 35.4187977270504 | 24.3295023351231 | 0 | 32.4094101603406 | -7.440 | 1.50422E-07 |
| ENSG00000239713 | **APOBEC3G** | 74.6329978425018 | 62.2753545114 | 79.7706026720814 | 158.352706019634 | 150.529890339964 | 223.020438071962 | 72.2263183419944 | 177.301011477187 | -1.296 | 1.61602E-07 |
| ENSG00000134245 | **WNT2B** | 67.4764090082893 | 60.2983591300857 | 56.9790019086296 | 139.61274081021 | 155.449167802055 | 149.018201802629 | 61.5845900156682 | 148.026703471631 | -1.265 | 1.73544E-07 |
| ENSG00000187372 | **PCDHB13** | 100.192243678975 | 140.366672073314 | 84.9505119365023 | 255.80052510864 | 260.721705490788 | 205.787040584583 | 108.503142562931 | 240.769757061337 | -1.149 | 1.88283E-07 |
| ENSG00000237330 | **RNF223** | 1.02236983345893 | 0.988497690657143 | 3.10794555865252 | 41.2279234607332 | 36.4026532194685 | 36.4942535026846 | 1.70627102758953 | 38.0416100609621 | -4.489 | 1.93426E-07 |
| ENSG00000145439 | **CBR4** | 442.686137887716 | 498.2028360912 | 421.644614123859 | 740.228625772256 | 703.456677078918 | 644.731811880762 | 454.177862700925 | 696.139038243978 | -0.616 | 2.06331E-07 |
| ENSG00000145819 | **ARHGAP26** | 381.34394788018 | 447.789453867686 | 411.284795595017 | 589.371905836391 | 674.924867798794 | 654.869104520396 | 413.472732447628 | 639.721959385194 | -0.629 | 2.36775E-07 |
| ENSG00000145934 | **TENM2** | 0 | 0 | 0 | 20.6139617303666 | 27.5479537877059 | 46.6315461423192 | 0 | 31.5978205534639 | -7.400 | 2.52916E-07 |
| ENSG00000113657 | **DPYSL3** | 72.5882581755839 | 50.4133822235143 | 84.9505119365023 | 155.541711238221 | 175.126277650416 | 170.306516345862 | 69.3173841118668 | 166.991501744833 | -1.270 | 2.83511E-07 |
| ENSG00000143036 | **SLC44A3** | 71.565888342125 | 101.815262137686 | 85.9864937893865 | 200.517627740839 | 202.674231438122 | 170.306516345862 | 86.4558814230657 | 191.166125174941 | -1.144 | 2.83855E-07 |
| ENSG00000205693 | **MANSC4** | 5.11184916729464 | 8.89647921591429 | 5.17990926442087 | 34.6689356374348 | 58.047474052666 | 50.6864631981731 | 6.3960792158766 | 47.8009576294246 | -2.896 | 3.15810E-07 |
| ENSG00000205089 | **CCNI2** | 125.751489515448 | 167.056109721057 | 139.857550139364 | 269.855499015708 | 277.447248861895 | 268.638254950317 | 144.221716458623 | 271.980334275974 | -0.914 | 3.74548E-07 |
| ENSG00000219891 | **ZSCAN12P1** | 224.921363360964 | 255.032404189543 | 204.088425018182 | 371.051311146599 | 401.413040906571 | 388.258308098006 | 228.014064189563 | 386.907553383725 | -0.762 | 4.00740E-07 |
| ENSG00000163686 | **ABHD6** | 235.145061695553 | 229.331464232457 | 227.916007634518 | 364.492323323301 | 424.041717232187 | 370.011181346664 | 230.79751118751 | 386.18174063405 | -0.743 | 4.69093E-07 |
| ENSG00000261780 | **LINC02582** | 765.755005260737 | 685.0288996254 | 1053.59354438321 | 1741.87976621598 | 1799.47169563264 | 2081.18617891699 | 834.792483089781 | 1874.17921358854 | -1.167 | 4.89467E-07 |
| ENSG00000077274 | **CAPN6** | 0 | 0 | 0 | 29.9839443350787 | 25.5802428028697 | 26.35696086305 | 0 | 27.3070493336662 | -7.192 | 6.14015E-07 |
| ENSG00000135114 | **OASL** | 37.8276838379803 | 47.4478891515429 | 33.1514192922936 | 133.990751247383 | 97.4016937493886 | 106.441572716163 | 39.4756640939389 | 112.611339237645 | -1.512 | 6.15822E-07 |
| ENSG00000232324 | **AC008440.3** | 1.02236983345893 | 0 | 4.1439274115367 | 34.6689356374348 | 42.3057861739769 | 31.4256071828673 | 1.72209908166521 | 36.133442998093 | -4.412 | 6.49964E-07 |
| ENSG00000155816 | **FMN2** | 0 | 0 | 0 | 26.2359512931939 | 37.3865087118865 | 19.2608560153058 | 0 | 27.6277720067954 | -7.209 | 6.93665E-07 |
| ENSG00000167680 | **SEMA6B** | 782.11292259608 | 803.648622504257 | 540.782527205539 | 1204.0427647055 | 1077.32176419778 | 1085.70404170487 | 708.848024101959 | 1122.35619020272 | -0.663 | 7.67549E-07 |
| ENSG00000283480 | **AL512380.2** | 34.7605743376036 | 31.6319261010286 | 18.6476733519151 | 113.376789517016 | 91.4985607948802 | 78.0571533251866 | 28.3467245968491 | 94.3108345456944 | -1.734 | 7.93924E-07 |
| ENSG00000085741 | **WNT11** | 42.939533005275 | 48.4363868422 | 43.5112378211353 | 120.872775600786 | 108.224104165987 | 119.620053147688 | 44.9623858895368 | 116.238977638154 | -1.370 | 8.05263E-07 |
| ENSG00000129474 | **AJUBA** | 406.903193716653 | 380.571610903 | 380.205340008492 | 639.969811901836 | 538.168954352683 | 604.182641322223 | 389.226714876049 | 594.107135858914 | -0.610 | 9.11555E-07 |
| novel.129 | **-** | 9.20132850113035 | 2.96549307197143 | 1.03598185288417 | 37.4799304188484 | 39.3542196967227 | 47.6452754062827 | 4.40093447532865 | 41.4931418406179 | -3.237 | 1.13015E-06 |
| ENSG00000027075 | **PRKCH** | 264.793786865862 | 240.204938829686 | 265.211354338349 | 438.515185900526 | 405.348462876244 | 396.368142209714 | 256.736693344632 | 413.410596995494 | -0.688 | 1.13737E-06 |
| ENSG00000263786 | **AC022211.1** | 131.885708516202 | 135.424183620029 | 116.029967523028 | 226.753579034033 | 264.65712746046 | 232.144001447633 | 127.779953219753 | 241.184902647375 | -0.916 | 1.20868E-06 |
| ENSG00000118526 | **TCF21** | 4.08947933383571 | 0.988497690657143 | 2.07196370576835 | 42.1649217212044 | 23.6125318180336 | 40.5491705585385 | 2.38331357675373 | 35.4422080325922 | -3.901 | 1.27858E-06 |
| ENSG00000119514 | **GALNT12** | 198.339747691032 | 223.400478088514 | 177.152896843194 | 416.027227649217 | 326.640023482798 | 323.379635204344 | 199.631040874247 | 355.34896211212 | -0.832 | 1.32985E-06 |
| ENSG00000130707 | **ASS1** | 113.483051513941 | 98.8497690657143 | 85.9864937893865 | 216.446598168849 | 182.99712158976 | 206.800769848546 | 99.4397714563473 | 202.081496535719 | -1.023 | 1.33894E-06 |
| ENSG00000254714 | **AP006587.1** | 17.3802871688018 | 19.7699538131429 | 13.4677640874943 | 63.7158817120423 | 76.7407284086092 | 58.7962973098808 | 16.8726683564796 | 66.4176358101774 | -1.975 | 1.40340E-06 |
| ENSG00000142273 | **CBLC** | 130.863338682743 | 112.688736734914 | 141.929513845132 | 258.611519890054 | 243.996162119681 | 224.034167335925 | 128.493863087596 | 242.213949781887 | -0.916 | 1.45875E-06 |
| ENSG00000125848 | **FLRT3** | 0 | 1.97699538131429 | 3.10794555865252 | 28.1099478141363 | 27.5479537877059 | 48.6590046702462 | 1.69498031332227 | 34.7723020906961 | -4.359 | 1.48126E-06 |
| ENSG00000103811 | **CTSH** | 338.404414874905 | 376.617620140372 | 305.614646600831 | 543.458991073302 | 501.766301133214 | 539.303968428562 | 340.212227205369 | 528.176420211693 | -0.634 | 1.61332E-06 |
| ENSG00000105088 | **OLFM2** | 23.5145061695554 | 27.6779353384 | 19.6836552047993 | 98.384817349477 | 62.9667515147563 | 82.1120703810404 | 23.6253655709182 | 81.1545464150912 | -1.779 | 1.71348E-06 |
| ENSG00000013588 | **GPRC5A** | 2861.61316385154 | 2454.43976590169 | 3785.47769043877 | 5891.84506184297 | 6737.44241207892 | 7609.05185530974 | 3033.843540064 | 6746.11310974388 | -1.153 | 1.81659E-06 |
| ENSG00000272398 | **CD24** | 3.06710950037678 | 3.95399076262857 | 8.2878548230734 | 45.9129147630893 | 33.4510867422143 | 41.5628998225019 | 5.10298502869292 | 40.3089671092685 | -2.987 | 1.92355E-06 |
| ENSG00000133985 | **TTC9** | 219.80951419367 | 216.480994253914 | 198.908515753761 | 373.862305928013 | 353.204121778086 | 327.434552260198 | 211.733008067115 | 351.500326655432 | -0.732 | 1.96048E-06 |
| ENSG00000074370 | **ATP2A3** | 3.06710950037678 | 5.93098614394286 | 3.10794555865252 | 40.290925200262 | 34.4349422346324 | 35.4805242387212 | 4.03534706765739 | 36.7354638912052 | -3.181 | 2.04933E-06 |
| ENSG00000125850 | **OVOL2** | 39.8724235048982 | 36.5744145543143 | 36.2593648509461 | 112.439791256545 | 89.5308498100441 | 102.38665566031 | 37.5687343033862 | 101.4524322423 | -1.434 | 2.52361E-06 |
| ENSG00000153976 | **HS3ST3A1** | 0 | 0 | 2.07196370576835 | 36.5429321583772 | 42.3057861739769 | 40.5491705585385 | 0.69065456858945 | 39.7992962969642 | -5.875 | 2.56851E-06 |
| ENSG00000069535 | **MAOB** | 0 | 0 | 0 | 24.3619547722515 | 19.6771098483613 | 25.3432315990865 | 0 | 23.1274320732331 | -6.952 | 2.57371E-06 |
| ENSG00000163235 | **TGFA** | 29.6487251703089 | 31.6319261010286 | 31.0794555865252 | 90.8888312657073 | 82.6438613631176 | 91.2356337567116 | 30.7867022859542 | 88.2561087951788 | -1.519 | 3.19764E-06 |
| ENSG00000154451 | **GBP5** | 1.02236983345893 | 2.96549307197143 | 0 | 37.4799304188484 | 25.5802428028697 | 33.4530657107942 | 1.32928763514345 | 32.1710796441708 | -4.576 | 3.25807E-06 |
| ENSG00000142765 | **SYTL1** | 284.218813701582 | 292.595316434514 | 285.930991396032 | 414.153231128275 | 448.638104542639 | 479.493941854717 | 287.581707177376 | 447.428425841877 | -0.637 | 3.40030E-06 |
| ENSG00000262714 | **AC007342.5** | 25.5592458364732 | 17.7929584318286 | 17.611691499031 | 64.6528799725135 | 79.6922948858634 | 67.9198606855519 | 20.3212985891109 | 70.7550118479763 | -1.800 | 3.77370E-06 |
| ENSG00000197872 | **FAM49A** | 1.02236983345893 | 0 | 0 | 45.9129147630893 | 41.3219306815588 | 22.3020438071962 | 0.340789944486309 | 36.5122964172814 | -6.651 | 3.82108E-06 |
| ENSG00000078401 | **EDN1** | 0 | 1.97699538131429 | 1.03598185288417 | 25.2989530327227 | 22.6286763256155 | 57.7825680459173 | 1.00432574473282 | 35.2367324680852 | -5.117 | 3.96881E-06 |
| ENSG00000171368 | **TPPP** | 88.9461755109268 | 103.792257519 | 92.2023849066915 | 177.092671229059 | 172.174711173162 | 236.198918503487 | 94.9802726455394 | 195.155433635236 | -1.037 | 4.14271E-06 |
| ENSG00000174950 | **CD164L2** | 46.0066425056518 | 53.3788752954857 | 50.7631107913245 | 128.368761684556 | 102.320971211479 | 133.812262843177 | 50.049542864154 | 121.500665246404 | -1.279 | 4.32795E-06 |
| ENSG00000105997 | **HOXA3** | 88.9461755109268 | 71.1718337273143 | 40.4032922624828 | 158.352706019634 | 164.303867233817 | 161.18295297019 | 66.8404338335746 | 161.279842074547 | -1.270 | 4.38947E-06 |
| ENSG00000272841 | **AL139393.2** | 124.729119681989 | 99.8382667563715 | 149.181386815321 | 256.737523369111 | 229.23832973341 | 227.075355127815 | 124.582924417894 | 237.683736076779 | -0.934 | 4.91748E-06 |
| ENSG00000228594 | **FNDC10** | 61.3421900075357 | 58.3213637487714 | 31.0794555865252 | 132.116754726441 | 126.917358521931 | 121.647511675615 | 50.2476697809441 | 126.893874974662 | -1.335 | 7.08698E-06 |
| ENSG00000261804 | **AC007342.4** | 15.3355475018839 | 14.8274653598571 | 23.827582616336 | 71.2118677958119 | 62.9667515147563 | 59.8100265738442 | 17.996865159359 | 64.6628819614708 | -1.848 | 7.68284E-06 |
| ENSG00000186340 | **THBS2** | 13.2908078349661 | 11.8619722878857 | 17.611691499031 | 57.1568938887438 | 66.9021734844286 | 47.6452754062827 | 14.2548238739609 | 57.234780926485 | -2.008 | 8.05297E-06 |
| ENSG00000146955 | **RAB19** | 10.2236983345893 | 16.8044607411714 | 7.25187297018922 | 63.7158817120423 | 47.2250636360672 | 47.6452754062827 | 11.42667734865 | 52.8620735847974 | -2.206 | 8.16188E-06 |
| ENSG00000127084 | **FGD3** | 100.192243678975 | 90.9417875404572 | 75.6266752605447 | 199.580629480368 | 179.061699620088 | 160.169223706227 | 88.9202354933256 | 179.603850935561 | -1.015 | 8.42061E-06 |
| ENSG00000229891 | **LINC01315** | 213.675295192916 | 178.918082008943 | 154.361296079742 | 332.634382467279 | 299.092069695092 | 308.173696244892 | 182.3182244272 | 313.300049469088 | -0.781 | 8.57455E-06 |
| ENSG00000183196 | **CHST6** | 47.0290123391107 | 58.3213637487714 | 34.1874011451778 | 116.18778429843 | 115.111092612914 | 114.551406827871 | 46.51259241102 | 115.283427913072 | -1.307 | 9.07723E-06 |
| ENSG00000150782 | **IL18** | 2.04473966691786 | 2.96549307197143 | 2.07196370576835 | 29.9839443350787 | 31.4833757573781 | 27.3706901270135 | 2.36073214821921 | 29.6126700731568 | -3.645 | 9.20118E-06 |
| ENSG00000011465 | **DCN** | 5.11184916729464 | 2.96549307197143 | 6.21589111730505 | 49.6609078049741 | 37.3865087118865 | 24.3295023351231 | 4.76441111885704 | 37.1256396173279 | -2.969 | 9.42918E-06 |
| ENSG00000184669 | **OR7E14P** | 1.02236983345893 | 0 | 1.03598185288417 | 47.7869112840317 | 26.5640982952878 | 29.3981486549404 | 0.686117228781034 | 34.5830527447533 | -5.675 | 9.90879E-06 |
| ENSG00000100979 | **PLTP** | 528.565203898266 | 505.1223199258 | 411.284795595017 | 812.377491828539 | 652.296191473179 | 711.63794330235 | 481.657439806361 | 725.437208868022 | -0.591 | 1.09073E-05 |
| ENSG00000198944 | **SOWAHA** | 47.0290123391107 | 56.3443683674572 | 39.3673104095986 | 108.69179821466 | 121.014225567422 | 112.523948299944 | 47.5802303720555 | 114.076657360676 | -1.260 | 1.13404E-05 |
| ENSG00000197461 | **PDGFA** | 74.6329978425018 | 84.0223037058572 | 75.6266752605447 | 145.234730373038 | 158.400734279309 | 173.347704137752 | 78.0939922696346 | 158.994389596699 | -1.024 | 1.18096E-05 |
| ENSG00000126561 | **STAT5A** | 2.04473966691786 | 7.90798152525714 | 3.10794555865252 | 42.1649217212044 | 30.4995202649601 | 33.4530657107942 | 4.35355558360917 | 35.3725025656529 | -3.014 | 1.19613E-05 |
| ENSG00000160588 | **MPZL3** | 158.467324186134 | 162.113621267771 | 181.29682425473 | 259.548518150525 | 308.930624619273 | 283.844193909769 | 167.292589902879 | 284.107778893189 | -0.764 | 1.26100E-05 |
| ENSG00000262434 | **AC087392.4** | 7.1565888342125 | 2.96549307197143 | 4.1439274115367 | 32.7949391164923 | 30.4995202649601 | 42.5766290864654 | 4.75533643924021 | 35.2903628226393 | -2.894 | 1.33922E-05 |
| ENSG00000135046 | **ANXA1** | 1710.42473137679 | 2072.87965730803 | 2528.83170289027 | 3925.0857131139 | 3547.78290565955 | 4017.4090730872 | 2104.04536385836 | 3830.09256395355 | -0.864 | 1.36937E-05 |
| ENSG00000149177 | **PTPRJ** | 318.979388039186 | 357.836164017886 | 401.96095891906 | 503.16806587304 | 569.652330110061 | 549.441261068196 | 359.592170325377 | 540.753885683766 | -0.588 | 1.55724E-05 |
| ENSG00000198626 | **RYR2** | 0 | 0.988497690657143 | 0 | 30.9209425955499 | 28.5318092801239 | 27.3706901270135 | 0.329499230219048 | 28.9411473342291 | -6.314 | 1.63353E-05 |
| ENSG00000158220 | **ESYT3** | 64.4092995079125 | 74.1373267992857 | 92.2023849066915 | 147.10872689398 | 172.174711173162 | 158.1417651783 | 76.9163370712966 | 159.141734415147 | -1.049 | 1.63695E-05 |
| ENSG00000139329 | **LUM** | 2.04473966691786 | 0 | 0 | 49.6609078049741 | 22.6286763256155 | 26.35696086305 | 0.681579888972619 | 32.8821816645466 | -5.603 | 1.87100E-05 |
| ENSG00000064787 | **BCAS1** | 1.02236983345893 | 5.93098614394286 | 4.1439274115367 | 40.290925200262 | 32.4672312497962 | 25.3432315990865 | 3.69909446297949 | 32.7004626830483 | -3.140 | 1.97695E-05 |
| ENSG00000006555 | **TTC22** | 276.039855033911 | 266.894376477429 | 227.916007634518 | 381.358292011782 | 406.332318368662 | 409.546622641239 | 256.950079715286 | 399.079077673894 | -0.634 | 1.98242E-05 |
| ENSG00000215386 | **MIR99AHG** | 0 | 0.988497690657143 | 0 | 41.2279234607332 | 24.5963873104517 | 19.2608560153058 | 0.329499230219048 | 28.3617222621636 | -6.287 | 2.38835E-05 |
| ENSG00000165509 | **MAGEC3** | 0 | 0 | 0 | 15.9289704280106 | 15.7416878786891 | 20.2745852792692 | 0 | 17.315081195323 | -6.534 | 2.60641E-05 |
| ENSG00000260896 | **LINC02170** | 14.313177668425 | 7.90798152525714 | 17.611691499031 | 52.4719025863877 | 59.031329545084 | 47.6452754062827 | 13.277616897571 | 53.0495025125848 | -2.003 | 2.67669E-05 |
| ENSG00000120327 | **PCDHB14** | 99.169873845516 | 105.769252900314 | 84.9505119365023 | 168.659686884818 | 190.867965529105 | 187.53991383324 | 96.6298795607776 | 182.355855415721 | -0.915 | 2.95484E-05 |
| ENSG00000170323 | **FABP4** | 12.2684380015071 | 12.8504699785429 | 8.2878548230734 | 51.5349043259165 | 59.031329545084 | 35.4805242387212 | 11.1355876010411 | 48.6822527032406 | -2.127 | 3.00018E-05 |
| ENSG00000128283 | **CDC42EP1** | 425.305850718914 | 271.836864930714 | 378.133376302724 | 633.410824078538 | 538.168954352683 | 551.468719596123 | 358.425363984117 | 574.349499342448 | -0.681 | 3.01789E-05 |
| ENSG00000265800 | **AC022211.3** | 71.565888342125 | 86.9877967778286 | 77.6986389663131 | 161.163700801048 | 182.99712158976 | 133.812262843177 | 78.7507746954222 | 159.324361744662 | -1.017 | 3.41355E-05 |
| ENSG00000053918 | **KCNQ1** | 11.2460681680482 | 11.8619722878857 | 3.10794555865252 | 51.5349043259165 | 40.3380751891408 | 40.5491705585385 | 8.73866200486215 | 44.1407166911986 | -2.332 | 3.62676E-05 |
| ENSG00000138131 | **LOXL4** | 17.3802871688018 | 17.7929584318286 | 10.3598185288417 | 57.1568938887438 | 40.3380751891408 | 79.07088258915 | 15.1776880431574 | 58.8552838890115 | -1.952 | 3.72340E-05 |
| ENSG00000184454 | **NCMAP** | 7.1565888342125 | 5.93098614394286 | 5.17990926442087 | 31.8579408560211 | 34.4349422346324 | 40.5491705585385 | 6.08916141419208 | 35.614017883064 | -2.547 | 3.78170E-05 |
| ENSG00000272189 | **AL024508.2** | 20.4473966691786 | 7.90798152525714 | 10.3598185288417 | 75.896859098168 | 36.4026532194685 | 55.7551095179904 | 12.9050655744258 | 56.0182072785423 | -2.121 | 4.20860E-05 |
| ENSG00000251348 | **HSPD1P11** | 22.4921363360964 | 31.6319261010286 | 35.2233829980619 | 81.5188486609952 | 86.5792833327899 | 75.0159655332962 | 29.782481811729 | 81.0380325090271 | -1.444 | 4.27694E-05 |
| ENSG00000106541 | **AGR2** | 0 | 0 | 0 | 12.1809773861257 | 18.6932543559433 | 17.2333974873789 | 0 | 16.035876409816 | -6.423 | 4.83759E-05 |
| ENSG00000114646 | **CSPG5** | 191.18315885682 | 194.734045059457 | 177.152896843194 | 301.71343987173 | 292.205081248166 | 301.077591397148 | 187.69003358649 | 298.332037505681 | -0.668 | 4.88432E-05 |
| ENSG00000269416 | **LINC01224** | 123.70674984853 | 152.2286443612 | 68.3748022903555 | 232.37556859686 | 228.254474240992 | 241.267564823304 | 114.770065500029 | 233.965869220385 | -1.026 | 4.92909E-05 |
| ENSG00000085117 | **CD82** | 96.1027643451392 | 84.0223037058572 | 100.490239729765 | 188.336650354713 | 159.384589771727 | 178.416350457569 | 93.5384359269204 | 175.379196861336 | -0.908 | 4.95506E-05 |
| ENSG00000249988 | **AC092546.1** | 1.02236983345893 | 0 | 3.10794555865252 | 31.8579408560211 | 35.4187977270504 | 13.178480431525 | 1.37677179737048 | 26.8184063381989 | -4.304 | 5.08969E-05 |
| ENSG00000272341 | **AL137003.2** | 30.6710950037678 | 51.4018799141714 | 39.3673104095986 | 88.0778364842937 | 95.4339827645525 | 120.633782411652 | 40.4800951091793 | 101.381867220166 | -1.322 | 5.33743E-05 |
| ENSG00000177096 | **FAM109B** | 12.2684380015071 | 15.8159630505143 | 15.5397277932626 | 42.1649217212044 | 52.1443410981576 | 66.9061314215885 | 14.5413762817613 | 53.7384647469835 | -1.884 | 5.45064E-05 |
| ENSG00000090530 | **P3H2** | 2.04473966691786 | 1.97699538131429 | 0 | 27.1729495536651 | 29.515664772542 | 18.2471267513423 | 1.34057834941071 | 24.9785803591831 | -4.210 | 5.69885E-05 |
| ENSG00000237438 | **CECR7** | 0 | 0 | 0 | 17.802966948953 | 15.7416878786891 | 13.178480431525 | 0 | 15.5743784197224 | -6.383 | 5.75164E-05 |
| ENSG00000074527 | **NTN4** | 46.0066425056518 | 36.5744145543143 | 53.8710563499771 | 117.124782558901 | 100.353260226643 | 99.3454678684193 | 45.4840378033144 | 105.607836884654 | -1.218 | 5.99435E-05 |
| ENSG00000185567 | **AHNAK2** | 98.1475040120571 | 163.102118958429 | 171.972987578773 | 594.056897138747 | 368.945809656775 | 347.709137539467 | 144.407536849753 | 436.903948111663 | -1.597 | 6.07610E-05 |
| novel.89 | **-** | 1.02236983345893 | 9.88497690657143 | 4.1439274115367 | 25.2989530327227 | 46.2412081436492 | 35.4805242387212 | 5.01709138385569 | 35.673561805031 | -2.819 | 6.93083E-05 |
| ENSG00000163362 | **INAVA** | 206.518706358703 | 183.860570462229 | 152.289332373974 | 327.949391164923 | 266.624838445296 | 306.146237716965 | 180.889536398302 | 300.240155775728 | -0.731 | 7.39916E-05 |
| ENSG00000261949 | **GFY** | 1.02236983345893 | 0.988497690657143 | 1.03598185288417 | 29.0469460746075 | 19.6771098483613 | 23.3157730711596 | 1.01561645900008 | 24.0132763313762 | -4.565 | 7.59884E-05 |
| ENSG00000256008 | **AC125616.1** | 51.1184916729464 | 52.3903776048286 | 54.9070382028612 | 130.242758205498 | 142.65904640062 | 87.1807167008577 | 52.8053024935454 | 120.027507102325 | -1.186 | 7.67066E-05 |
| ENSG00000177181 | **RIMKLA** | 149.265995685004 | 166.0676120304 | 122.245858640333 | 214.572601647907 | 279.414959846731 | 270.665713478244 | 145.859822118579 | 254.884424990961 | -0.803 | 7.90302E-05 |
| ENSG00000125872 | **LRRN4** | 4.08947933383571 | 3.95399076262857 | 0 | 28.1099478141363 | 25.5802428028697 | 27.3706901270135 | 2.68115669882143 | 27.0202935813398 | -3.324 | 7.95587E-05 |
| ENSG00000181652 | **ATG9B** | 255.592458364732 | 260.963390333486 | 219.628152811445 | 386.98028157461 | 388.622919505137 | 348.722866803431 | 245.394667169888 | 374.775355961059 | -0.611 | 7.98302E-05 |
| ENSG00000235280 | **MCF2L-AS1** | 21.4697665026375 | 17.7929584318286 | 11.3958003817259 | 54.3458991073302 | 53.1281965905756 | 62.8512143657346 | 16.8861751053973 | 56.7751033545468 | -1.748 | 8.57339E-05 |
| ENSG00000157765 | **SLC34A2** | 0 | 0.988497690657143 | 0 | 15.9289704280106 | 23.6125318180336 | 28.3844193909769 | 0.329499230219048 | 22.641973879007 | -5.958 | 9.93224E-05 |
| ENSG00000136378 | **ADAMTS7** | 10.2236983345893 | 5.93098614394286 | 10.3598185288417 | 38.4169286793196 | 44.273497158813 | 36.4942535026846 | 8.83816766912463 | 39.7282264469391 | -2.172 | 1.06628E-04 |
| ENSG00000213030 | **CGB8** | 0 | 0 | 0 | 16.8659686884818 | 11.8062659090168 | 14.1922096954885 | 0 | 14.2881480976623 | -6.258 | 1.09519E-04 |
| ENSG00000149599 | **DUSP15** | 15.3355475018839 | 5.93098614394286 | 10.3598185288417 | 45.9129147630893 | 42.3057861739769 | 43.5903583504289 | 10.5421173915562 | 43.9363530958317 | -2.063 | 1.16198E-04 |
| ENSG00000100949 | **RABGGTA** | 173.802871688018 | 169.033105102371 | 163.6851327557 | 239.87155468063 | 288.269659278494 | 291.954028021477 | 168.840369848696 | 273.3650806602 | -0.694 | 1.21275E-04 |
| ENSG00000131620 | **ANO1** | 174.825241521477 | 190.780054296829 | 177.152896843194 | 266.107505973824 | 284.334237308821 | 310.201154772819 | 180.919397553833 | 286.880966018488 | -0.664 | 1.23179E-04 |
| ENSG00000153982 | **GDPD1** | 214.697665026375 | 272.825362621371 | 233.095916898939 | 360.744330281416 | 397.477618936899 | 348.722866803431 | 240.206314848895 | 368.981605340582 | -0.619 | 1.29481E-04 |
| ENSG00000170421 | **KRT8** | 14809.0270376526 | 12585.5525974467 | 18006.4005849798 | 24098.658261059 | 23976.5583502283 | 27880.5959467871 | 15133.6600733597 | 25318.6041860248 | -0.742 | 1.34995E-04 |
| ENSG00000158560 | **DYNC1I1** | 161.534433686511 | 169.033105102371 | 129.497731610522 | 293.280455527489 | 271.544115907387 | 218.965521016108 | 153.355090133135 | 261.263364150328 | -0.769 | 1.35063E-04 |
| ENSG00000179178 | **TMEM125** | 1.02236983345893 | 0 | 8.2878548230734 | 70.2748695353407 | 76.7407284086092 | 99.3454678684193 | 3.10340821884411 | 82.1203552707897 | -4.735 | 1.41006E-04 |
| ENSG00000112902 | **SEMA5A** | 0 | 0 | 0 | 10.3069808651833 | 9.83855492418067 | 22.3020438071962 | 0 | 14.1491931988534 | -6.241 | 1.48469E-04 |
| ENSG00000108821 | **COL1A1** | 25.5592458364732 | 39.5399076262857 | 27.9715100278727 | 87.1408382238225 | 80.6761503782815 | 70.9610484774423 | 31.0235544968772 | 79.5926790265154 | -1.358 | 1.61493E-04 |
| ENSG00000169495 | **HTRA4** | 30.6710950037678 | 16.8044607411714 | 23.827582616336 | 53.408900846859 | 95.4339827645525 | 64.8786728936616 | 23.7677127870918 | 71.2405188350243 | -1.585 | 1.66577E-04 |
| ENSG00000002745 | **WNT16** | 59.2974503406178 | 75.1258244899429 | 74.5906934076606 | 109.628796475132 | 152.4976013248 | 165.237870026044 | 69.6713227460738 | 142.454755941992 | -1.030 | 1.68817E-04 |
| ENSG00000196565 | **HBG2** | 3.06710950037678 | 0.988497690657143 | 3.10794555865252 | 28.1099478141363 | 20.6609653407794 | 23.3157730711596 | 2.38785091656215 | 24.0288954086918 | -3.340 | 1.71084E-04 |
| ENSG00000174567 | **GOLT1A** | 0 | 0 | 0 | 14.9919721675394 | 10.8224104165987 | 14.1922096954885 | 0 | 13.3355307598755 | -6.158 | 1.76184E-04 |
| ENSG00000249697 | **AC034245.1** | 0 | 0 | 0 | 14.0549739070681 | 9.83855492418067 | 16.2196682234154 | 0 | 13.3710656848881 | -6.161 | 1.80248E-04 |
| ENSG00000168032 | **ENTPD3** | 7.1565888342125 | 3.95399076262857 | 2.07196370576835 | 29.0469460746075 | 43.289641666395 | 19.2608560153058 | 4.39418110086981 | 30.5324812521027 | -2.796 | 1.92659E-04 |
| ENSG00000183960 | **KCNH8** | 172.780501854559 | 175.952588936971 | 210.304316135487 | 318.579408560211 | 263.673271968042 | 306.146237716965 | 186.345802309006 | 296.132972748406 | -0.669 | 1.93526E-04 |
| ENSG00000261553 | **AL137782.1** | 130.863338682743 | 149.263151289229 | 161.613169049931 | 262.359512931939 | 234.1576071955 | 229.102813655742 | 147.246553007301 | 241.87331126106 | -0.717 | 1.96649E-04 |
| ENSG00000130720 | **FIBCD1** | 50.0961218394875 | 25.7009399570857 | 30.0434737336411 | 91.8258295261786 | 85.5954278403718 | 87.1807167008577 | 35.2801785100714 | 88.2006580224694 | -1.323 | 1.96700E-04 |
| ENSG00000224081 | **SLC44A3-AS1** | 28.62635533685 | 38.5514099356286 | 22.7916007634518 | 74.9598608376968 | 83.6277168555357 | 72.9885070053693 | 29.9897886786435 | 77.1920282328672 | -1.361 | 2.07160E-04 |
| ENSG00000237289 | **CKMT1B** | 139.042297350414 | 110.7117413536 | 104.634167141302 | 217.383596429321 | 205.625797915376 | 190.581101625131 | 118.129401948439 | 204.530165323276 | -0.793 | 2.07570E-04 |
| ENSG00000236345 | **AL354719.2** | 40.8947933383571 | 36.5744145543143 | 31.0794555865252 | 95.5738225680634 | 78.7084393934454 | 82.1120703810404 | 36.1828878263989 | 85.4647774475164 | -1.240 | 2.29907E-04 |
| ENSG00000237517 | **DGCR5** | 0 | 0.988497690657143 | 0 | 21.5509599908378 | 18.6932543559433 | 18.2471267513423 | 0.329499230219048 | 19.4971136993745 | -5.745 | 2.32671E-04 |
| ENSG00000175591 | **P2RY2** | 28.62635533685 | 26.6894376477429 | 20.7196370576835 | 87.1408382238225 | 63.9506070071744 | 57.7825680459173 | 25.3451433474254 | 69.6246710923047 | -1.459 | 2.34386E-04 |
| ENSG00000282221 | **AC119427.1** | 0 | 0 | 0 | 19.6769634698954 | 3.93542196967227 | 18.2471267513423 | 0 | 13.9531707303033 | -6.224 | 2.38814E-04 |
| ENSG00000176532 | **PRR15** | 30.6710950037678 | 27.6779353384 | 17.611691499031 | 66.5268764934559 | 73.789161931355 | 65.892402157625 | 25.3202406137329 | 68.736146860812 | -1.439 | 2.44289E-04 |
| ENSG00000119915 | **ELOVL3** | 117.572530847777 | 79.0798152525714 | 68.3748022903555 | 171.470681666231 | 169.223144695908 | 165.237870026044 | 88.3423827969012 | 168.643898796061 | -0.933 | 2.65763E-04 |
| ENSG00000103528 | **SYT17** | 19.4250268357196 | 31.6319261010286 | 9.32383667595757 | 68.4008730143983 | 59.031329545084 | 64.8786728936616 | 20.1269298709019 | 64.103625151048 | -1.666 | 2.67149E-04 |
| ENSG00000107731 | **UNC5B** | 55.2079710067821 | 69.194838346 | 69.4107841432397 | 136.801746028797 | 133.804346968857 | 112.523948299944 | 64.6045311653406 | 127.710013765866 | -0.984 | 2.76842E-04 |
| ENSG00000225655 | **BX255923.1** | 122.684380015071 | 124.5507090228 | 154.361296079742 | 230.501572075918 | 216.448208331975 | 218.965521016108 | 133.865461705871 | 221.971767141333 | -0.731 | 2.78400E-04 |
| ENSG00000106327 | **TFR2** | 196.295008024114 | 171.998598174343 | 137.785586433595 | 311.083422476442 | 253.834717043861 | 265.597067158427 | 168.693064210684 | 276.838402226243 | -0.715 | 2.84470E-04 |
| ENSG00000168071 | **CCDC88B** | 57.2527106737 | 107.746248281629 | 66.3028385845872 | 172.407679926703 | 151.513745832382 | 144.963284746775 | 77.1005991799719 | 156.294903501953 | -1.018 | 2.86983E-04 |
| ENSG00000137843 | **PAK6** | 143.13177668425 | 160.136625886457 | 154.361296079742 | 250.178535545813 | 229.23832973341 | 251.404857462939 | 152.543232883483 | 243.607240914054 | -0.675 | 2.87712E-04 |
| ENSG00000122863 | **CHST3** | 0 | 0 | 1.03598185288417 | 14.0549739070681 | 19.6771098483613 | 23.3157730711596 | 0.345327284294725 | 19.0159522755297 | -5.706 | 2.90446E-04 |
| ENSG00000152689 | **RASGRP3** | 30.6710950037678 | 10.8734745972286 | 32.1154374394094 | 92.7628277866498 | 59.031329545084 | 69.9473192134789 | 24.5533356801353 | 73.9138255150709 | -1.595 | 2.92836E-04 |
| ENSG00000198203 | **SULT1C2** | 0 | 0 | 0 | 15.9289704280106 | 14.757832386271 | 7.09610484774423 | 0 | 12.5943025540086 | -6.077 | 3.04801E-04 |
| ENSG00000205293 | **LINC01602** | 1.02236983345893 | 0 | 2.07196370576835 | 21.5509599908378 | 19.6771098483613 | 20.2745852792692 | 1.03144451307576 | 20.5008850394895 | -4.332 | 3.09662E-04 |
| ENSG00000225489 | **AL354707.1** | 26.5816156699321 | 37.5629122449714 | 20.7196370576835 | 74.9598608376968 | 71.8214509465189 | 72.9885070053693 | 28.2880549908624 | 73.256606263195 | -1.370 | 3.16517E-04 |
| ENSG00000114654 | **EFCC1** | 57.2527106737 | 42.5054006982572 | 48.6911470855562 | 108.69179821466 | 97.4016937493886 | 106.441572716163 | 49.4830861525044 | 104.178354893404 | -1.075 | 3.30978E-04 |
| ENSG00000138771 | **SHROOM3** | 35.7829441710625 | 20.7584515038 | 33.1514192922936 | 76.8338573586392 | 74.7730174237731 | 74.0022362693327 | 29.897604989052 | 75.2030370172483 | -1.333 | 3.41123E-04 |
| ENSG00000104490 | **NCALD** | 0 | 0 | 0 | 12.1809773861257 | 12.7901214014349 | 11.1510219035981 | 0 | 12.0407068970529 | -6.011 | 3.47090E-04 |
| ENSG00000119125 | **GDA** | 0 | 0 | 0 | 17.802966948953 | 7.87084393934454 | 11.1510219035981 | 0 | 12.2749442639652 | -6.040 | 3.61157E-04 |
| ENSG00000005001 | **PRSS22** | 314.88990870535 | 245.147427282971 | 211.340297988372 | 434.767192858641 | 359.107254732594 | 404.477976321421 | 257.125877992231 | 399.450807970886 | -0.636 | 3.66495E-04 |
| ENSG00000124126 | **PREX1** | 10.2236983345893 | 4.94248845328572 | 4.1439274115367 | 44.9759165026181 | 27.5479537877059 | 27.3706901270135 | 6.43670473313723 | 33.2981868057791 | -2.374 | 3.70602E-04 |
| ENSG00000039139 | **DNAH5** | 80.7672168432553 | 68.2063406553429 | 106.70613084707 | 146.171728633509 | 172.174711173162 | 160.169223706227 | 85.2265627818894 | 159.505221170966 | -0.905 | 3.70602E-04 |
| ENSG00000279012 | **OR51B2** | 4.08947933383571 | 5.93098614394286 | 2.07196370576835 | 29.0469460746075 | 24.5963873104517 | 25.3432315990865 | 4.03080972784897 | 26.3288549947152 | -2.701 | 4.10756E-04 |
| ENSG00000260577 | **AC126773.2** | 3.06710950037678 | 6.9194838346 | 7.25187297018922 | 29.9839443350787 | 25.5802428028697 | 35.4805242387212 | 5.74615543505534 | 30.3482371255565 | -2.399 | 4.39420E-04 |
| ENSG00000095596 | **CYP26A1** | 14.313177668425 | 15.8159630505143 | 9.32383667595757 | 42.1649217212044 | 50.1766301133214 | 42.5766290864654 | 13.1509924649656 | 44.9727269736638 | -1.771 | 4.57869E-04 |
| ENSG00000154556 | **SORBS2** | 0 | 0.988497690657143 | 0 | 19.6769634698954 | 18.6932543559433 | 14.1922096954885 | 0.329499230219048 | 17.5208091737757 | -5.591 | 4.60758E-04 |
| ENSG00000103089 | **FA2H** | 100.192243678975 | 73.1488291086286 | 58.0149837615138 | 166.785690363875 | 160.368445264145 | 125.702428731469 | 77.1186855163724 | 150.95218811983 | -0.970 | 4.89581E-04 |
| ENSG00000158055 | **GRHL3** | 0 | 1.97699538131429 | 5.17990926442087 | 26.2359512931939 | 12.7901214014349 | 33.4530657107942 | 2.38563488191172 | 24.1597128018077 | -3.346 | 4.91587E-04 |
| ENSG00000176884 | **GRIN1** | 185.048939856066 | 205.607519656686 | 200.98047945953 | 298.902445090316 | 277.447248861895 | 316.2835303566 | 197.212312990761 | 297.544408102937 | -0.593 | 5.28462E-04 |
| ENSG00000157388 | **CACNA1D** | 34.7605743376036 | 34.597419173 | 37.2953467038303 | 76.8338573586392 | 75.7568729161912 | 90.2219044927481 | 35.5511134048113 | 80.9375449225262 | -1.187 | 5.37499E-04 |
| ENSG00000111845 | **PAK1IP1** | 136.997557683496 | 143.332165145286 | 228.951989487403 | 373.862305928013 | 244.980017612099 | 283.844193909769 | 169.760570772062 | 300.895505816627 | -0.827 | 5.37499E-04 |
| ENSG00000257671 | **KRT7-AS** | 0 | 0 | 0 | 11.2439791256545 | 7.87084393934454 | 15.2059389594519 | 0 | 11.4402540081503 | -5.936 | 5.44740E-04 |
| ENSG00000116661 | **FBXO2** | 77.7001073428786 | 89.9532898498 | 68.3748022903555 | 173.344678187174 | 114.127237120496 | 165.237870026044 | 78.6760664943447 | 150.903261777905 | -0.939 | 5.45893E-04 |
| ENSG00000197587 | **DMBX1** | 73.6106280090428 | 64.2523498927143 | 59.0509656143979 | 141.486737331153 | 121.99808105984 | 114.551406827871 | 65.6379811720517 | 126.012075072955 | -0.942 | 5.48472E-04 |
| ENSG00000236883 | **AP001615.1** | 4.08947933383571 | 11.8619722878857 | 13.4677640874943 | 42.1649217212044 | 39.3542196967227 | 36.4942535026846 | 9.80640523640523 | 39.3377983068706 | -2.004 | 5.51810E-04 |
| novel.608 | **-** | 3.06710950037678 | 6.9194838346 | 3.10794555865252 | 62.7788834515711 | 23.6125318180336 | 11.1510219035981 | 4.36484629787644 | 32.5141457244009 | -2.895 | 5.53728E-04 |
| ENSG00000280027 | **AC007342.9** | 9.20132850113035 | 5.93098614394286 | 7.25187297018922 | 25.2989530327227 | 38.3703642043046 | 36.4942535026846 | 7.46139587175414 | 33.3878569132373 | -2.162 | 5.68941E-04 |
| ENSG00000164362 | **TERT** | 90.9909151778446 | 98.8497690657143 | 92.2023849066915 | 145.234730373038 | 173.15856666558 | 174.361433401715 | 94.0143563834168 | 164.251576813444 | -0.804 | 5.70828E-04 |
| ENSG00000114656 | **KIAA1257** | 168.691022520723 | 159.1481281958 | 150.217368668205 | 238.934556420158 | 245.963873104517 | 256.473503782756 | 159.352173128243 | 247.123977769144 | -0.633 | 6.34981E-04 |
| ENSG00000137868 | **STRA6** | 0 | 1.97699538131429 | 3.10794555865252 | 17.802966948953 | 19.6771098483613 | 22.3020438071962 | 1.69498031332227 | 19.9273735348368 | -3.557 | 6.84740E-04 |
| ENSG00000196872 | **KIAA1211L** | 17.3802871688018 | 19.7699538131429 | 21.7556189105677 | 59.0308904096862 | 53.1281965905756 | 51.7001924621366 | 19.6352866308374 | 54.6197598207995 | -1.477 | 6.88664E-04 |
| ENSG00000264301 | **LINC01444** | 0 | 0 | 0 | 5.62198956282726 | 13.7739768938529 | 14.1922096954885 | 0 | 11.1960587173896 | -5.903 | 7.11826E-04 |
| ENSG00000234949 | **AC104667.2** | 17.3802871688018 | 18.7814561224857 | 12.4317822346101 | 45.9129147630893 | 57.0636185602479 | 44.6040876143923 | 16.1978418419659 | 49.1935403125765 | -1.601 | 7.67605E-04 |
| ENSG00000181045 | **SLC26A11** | 153.355475018839 | 145.3091605266 | 156.43325978551 | 237.060559899216 | 207.593508900212 | 284.857923173733 | 151.69929844365 | 243.170663991054 | -0.680 | 8.14348E-04 |
| ENSG00000058085 | **LAMC2** | 3023.14759753805 | 2389.19891831832 | 3432.20787860527 | 5692.2644323626 | 4738.24805148541 | 4384.37906664197 | 2948.18479815388 | 4938.29718349666 | -0.744 | 8.85945E-04 |
| ENSG00000136883 | **KIF12** | 64.4092995079125 | 81.0568106338857 | 59.0509656143979 | 121.809773861257 | 125.933503029513 | 135.839721371104 | 68.1723585853987 | 127.860999420625 | -0.905 | 8.87667E-04 |
| ENSG00000136828 | **RALGPS1** | 155.400214685757 | 227.354468851143 | 149.181386815321 | 276.414486839007 | 305.979058142019 | 273.706901270135 | 177.31202345074 | 285.366815417053 | -0.685 | 8.99271E-04 |
| ENSG00000108370 | **RGS9** | 51.1184916729464 | 56.3443683674572 | 39.3673104095986 | 113.376789517016 | 92.4824162872983 | 95.2905508125654 | 48.9433901500007 | 100.383252205627 | -1.036 | 1.01437E-03 |
| ENSG00000250240 | **AC008840.1** | 17.3802871688018 | 15.8159630505143 | 7.25187297018922 | 35.605933897906 | 52.1443410981576 | 47.6452754062827 | 13.4827077298351 | 45.1318501341154 | -1.739 | 1.05176E-03 |
| ENSG00000010319 | **SEMA3G** | 140.064667183873 | 167.056109721057 | 105.670148994186 | 255.80052510864 | 222.351341286483 | 208.828228376473 | 137.596975299705 | 228.993364923866 | -0.734 | 1.10014E-03 |
| ENSG00000074211 | **PPP2R2C** | 4.08947933383571 | 1.97699538131429 | 3.10794555865252 | 28.1099478141363 | 16.7255433711071 | 21.2883145432327 | 3.05814009126751 | 22.0412685761587 | -2.854 | 1.10524E-03 |
| ENSG00000266094 | **RASSF5** | 46.0066425056518 | 60.2983591300857 | 31.0794555865252 | 118.998779079844 | 103.304826703897 | 80.0846118531135 | 45.7948190740876 | 100.796072545618 | -1.137 | 1.10533E-03 |
| ENSG00000279692 | **AC110285.7** | 198.339747691032 | 172.987095865 | 154.361296079742 | 312.957418997384 | 246.947728596935 | 258.500962310683 | 175.229379878591 | 272.802036635001 | -0.639 | 1.11027E-03 |
| ENSG00000101665 | **SMAD7** | 10.2236983345893 | 4.94248845328572 | 9.32383667595757 | 46.8499130235605 | 25.5802428028697 | 30.4118779189039 | 8.16334115461086 | 34.2806779151114 | -2.076 | 1.13096E-03 |
| ENSG00000204054 | **LINC00963** | 107.348832513187 | 77.1028198712572 | 77.6986389663131 | 215.509599908378 | 139.707479923366 | 137.867179899031 | 87.3834304502526 | 164.361419910258 | -0.913 | 1.13609E-03 |
| ENSG00000197565 | **COL4A6** | 0 | 0 | 0 | 11.2439791256545 | 7.87084393934454 | 11.1510219035981 | 0 | 10.0886149895324 | -5.755 | 1.14123E-03 |
| ENSG00000189196 | **LINC00994** | 0 | 0.988497690657143 | 0 | 9.3699826047121 | 18.6932543559433 | 17.2333974873789 | 0.329499230219048 | 15.0988781493447 | -5.373 | 1.22527E-03 |
| ENSG00000016402 | **IL20RA** | 22.4921363360964 | 27.6779353384 | 32.1154374394094 | 65.5898782329847 | 60.9990405299202 | 70.9610484774423 | 27.4285030379686 | 65.8499890801157 | -1.264 | 1.31320E-03 |
| ENSG00000226919 | **AL365184.1** | 0 | 1.97699538131429 | 0 | 13.1179756465969 | 21.6448208331975 | 18.2471267513423 | 0.658998460438095 | 17.6699744103789 | -4.711 | 1.32528E-03 |
| ENSG00000081041 | **CXCL2** | 43.9619028387339 | 33.6089214823429 | 53.8710563499771 | 84.3298434424089 | 88.546994317626 | 104.414114188237 | 43.8139602236846 | 92.4303173160905 | -1.078 | 1.35024E-03 |
| ENSG00000159618 | **ADGRG5** | 174.825241521477 | 177.929584318286 | 164.721114608584 | 283.910472922777 | 266.624838445296 | 233.157730711596 | 172.491980149449 | 261.231014026556 | -0.599 | 1.38186E-03 |
| ENSG00000158125 | **XDH** | 0 | 0 | 0 | 13.1179756465969 | 4.91927746209034 | 12.1647511675615 | 0 | 10.0673347587496 | -5.753 | 1.38396E-03 |
| ENSG00000099617 | **EFNA2** | 16.3579173353429 | 23.7239445757714 | 12.4317822346101 | 48.7239095445029 | 57.0636185602479 | 45.6178168783558 | 17.5045480485748 | 50.4684483277022 | -1.524 | 1.42715E-03 |
| ENSG00000268869 | **ESPNP** | 5.11184916729464 | 9.88497690657143 | 4.1439274115367 | 35.605933897906 | 28.5318092801239 | 24.3295023351231 | 6.38025116180092 | 29.4890818377177 | -2.204 | 1.42908E-03 |
| ENSG00000261437 | **AC108860.2** | 46.0066425056518 | 49.4248845328572 | 35.2233829980619 | 105.880803433247 | 87.563138825208 | 80.0846118531135 | 43.551636678857 | 91.1761847038561 | -1.066 | 1.48200E-03 |
| novel.272 | **-** | 55.2079710067821 | 44.4823960795714 | 70.4467659961239 | 102.132810391362 | 230.222185225828 | 317.297259620564 | 56.7123776941591 | 216.550751745918 | -1.933 | 1.48408E-03 |
| ENSG00000106991 | **ENG** | 18.4026570022607 | 18.7814561224857 | 7.25187297018922 | 46.8499130235605 | 37.3865087118865 | 57.7825680459173 | 14.8119953649785 | 47.3396632604548 | -1.672 | 1.53012E-03 |
| ENSG00000128965 | **CHAC1** | 83.8343263436321 | 58.3213637487714 | 79.7706026720814 | 124.620768642671 | 126.917358521931 | 154.086848122446 | 73.975430921495 | 135.208325095683 | -0.871 | 1.60416E-03 |
| ENSG00000163995 | **ABLIM2** | 113.483051513941 | 108.734745972286 | 93.2383667595757 | 163.974695582462 | 187.916399051851 | 169.292787081898 | 105.152054748601 | 173.72796057207 | -0.724 | 1.62191E-03 |
| ENSG00000108375 | **RNF43** | 0 | 0 | 0 | 8.43298434424089 | 9.83855492418067 | 10.1372926396346 | 0 | 9.46961063601873 | -5.663 | 1.65468E-03 |
| ENSG00000227053 | **AC105446.1** | 2.04473966691786 | 10.8734745972286 | 6.21589111730505 | 36.5429321583772 | 27.5479537877059 | 26.35696086305 | 6.37803512715049 | 30.149282269711 | -2.236 | 1.66206E-03 |
| novel.880 | **-** | 630.802187244159 | 568.386172127857 | 1214.17073158025 | 1862.75254181676 | 2074.9512335097 | 2798.90649780312 | 804.453030317423 | 2245.53675770986 | -1.481 | 1.69417E-03 |
| ENSG00000137440 | **FGFBP1** | 31.6934648372268 | 54.3673729861429 | 12.4317822346101 | 89.9518330052361 | 105.272537688733 | 68.9335899495154 | 32.8308733526599 | 88.0526535478282 | -1.420 | 1.81977E-03 |
| ENSG00000189398 | **OR7E12P** | 0 | 0 | 0 | 13.1179756465969 | 6.88698844692647 | 8.10983411170769 | 0 | 9.3715994017437 | -5.651 | 1.90403E-03 |
| ENSG00000107014 | **RLN2** | 54.1856011733232 | 42.5054006982572 | 31.0794555865252 | 94.6368243075922 | 73.789161931355 | 104.414114188237 | 42.5901524860352 | 90.9467001423946 | -1.094 | 2.01158E-03 |
| ENSG00000092330 | **TINF2** | 24.5368760030143 | 20.7584515038 | 24.8635644692202 | 70.2748695353407 | 24.5963873104517 | 129.757345787323 | 23.3862973253448 | 74.8762008777052 | -1.678 | 2.21774E-03 |
| ENSG00000074771 | **NOX3** | 3.06710950037678 | 3.95399076262857 | 4.1439274115367 | 19.6769634698954 | 18.6932543559433 | 27.3706901270135 | 3.72167589151402 | 21.913635984284 | -2.556 | 2.28258E-03 |
| ENSG00000027869 | **SH2D2A** | 118.594900681236 | 87.9762944684857 | 71.482747849008 | 172.407679926703 | 152.4976013248 | 159.155494442264 | 92.6846476662432 | 161.353591897922 | -0.800 | 2.28758E-03 |
| ENSG00000203386 | **LINC01317** | 0 | 0 | 0 | 14.0549739070681 | 3.93542196967227 | 10.1372926396346 | 0 | 9.37589617212501 | -5.651 | 2.28982E-03 |
| ENSG00000001617 | **SEMA3F** | 160.512063853052 | 154.205639742514 | 142.965495698016 | 236.123561638745 | 247.931584089353 | 210.8556869044 | 152.561066431194 | 231.636944210833 | -0.603 | 2.37962E-03 |
| ENSG00000177989 | **ODF3B** | 64.4092995079125 | 54.3673729861429 | 46.6191833797878 | 98.384817349477 | 100.353260226643 | 116.578865355798 | 55.1319519579477 | 105.105647643973 | -0.930 | 2.39406E-03 |
| ENSG00000188818 | **ZDHHC11** | 29.6487251703089 | 64.2523498927143 | 45.5832015269037 | 89.0148347447649 | 98.3855492418067 | 104.414114188237 | 46.494758863309 | 97.2714993916027 | -1.062 | 2.73879E-03 |
| ENSG00000259953 | **AL138756.1** | 62.3645598409946 | 55.3558706768 | 61.1229293201663 | 104.943805172775 | 115.111092612914 | 106.441572716163 | 59.6144532793203 | 108.832156833951 | -0.869 | 2.77321E-03 |
| ENSG00000278771 | **RN7SL3** | 151.310735351921 | 101.815262137686 | 91.1664030538073 | 465.688135454191 | 575.555463064569 | 192.608560153058 | 114.764133514471 | 411.284052890606 | -1.842 | 2.77658E-03 |
| ENSG00000168772 | **CXXC4** | 68.4987788417482 | 111.700239044257 | 81.8425663778498 | 142.423735591624 | 136.755913446111 | 188.553643097204 | 87.3471947546184 | 155.911097378313 | -0.834 | 3.03617E-03 |
| ENSG00000178038 | **ALS2CL** | 36.8053140045214 | 40.5284053169429 | 39.3673104095986 | 73.0858643167544 | 77.7245839010273 | 88.1944459648212 | 38.9003432436876 | 79.6682980608676 | -1.033 | 3.07051E-03 |
| ENSG00000142677 | **IL22RA1** | 0 | 2.96549307197143 | 0 | 22.487958251309 | 16.7255433711071 | 9.12356337567116 | 0.988497690657143 | 16.1123549993624 | -4.000 | 3.15141E-03 |
| ENSG00000132677 | **RHBG** | 0 | 0 | 0 | 8.43298434424089 | 7.87084393934454 | 9.12356337567116 | 0 | 8.47579721975219 | -5.504 | 3.15210E-03 |
| ENSG00000196337 | **CGB7** | 3.06710950037678 | 4.94248845328572 | 0 | 19.6769634698954 | 20.6609653407794 | 18.2471267513423 | 2.66986598455417 | 19.5283518540057 | -2.859 | 3.31147E-03 |
| ENSG00000179071 | **CCDC89** | 33.7382045041446 | 33.6089214823429 | 47.655165232672 | 97.4478190890058 | 71.8214509465189 | 72.9885070053693 | 38.3340970730532 | 80.7525923469647 | -1.078 | 3.36824E-03 |
| ENSG00000186977 | **KRTAP19-5** | 1.02236983345893 | 0 | 0 | 8.43298434424089 | 11.8062659090168 | 17.2333974873789 | 0.340789944486309 | 12.4908825802122 | -5.099 | 3.38989E-03 |
| ENSG00000101335 | **MYL9** | 38.8500536714393 | 15.8159630505143 | 17.611691499031 | 73.0858643167544 | 60.9990405299202 | 52.7139217261 | 24.0925694069948 | 62.2662755242582 | -1.372 | 3.40587E-03 |
| ENSG00000283486 | **FAM95C** | 43.9619028387339 | 59.3098614394286 | 71.482747849008 | 96.5108208285346 | 121.014225567422 | 112.523948299944 | 58.2515040423902 | 110.0163315653 | -0.917 | 3.44350E-03 |
| ENSG00000104814 | **MAP4K1** | 6.13421900075357 | 5.93098614394286 | 0 | 20.6139617303666 | 29.515664772542 | 20.2745852792692 | 4.02173504823214 | 23.4680705940593 | -2.537 | 3.52058E-03 |
| ENSG00000183117 | **CSMD1** | 1.02236983345893 | 0.988497690657143 | 4.1439274115367 | 18.7399652094242 | 14.757832386271 | 18.2471267513423 | 2.05159831188426 | 17.2483081156792 | -3.082 | 3.66907E-03 |
| ENSG00000175746 | **C15orf54** | 0 | 0 | 0 | 3.74799304188484 | 11.8062659090168 | 10.1372926396346 | 0 | 8.56385053017875 | -5.517 | 3.68232E-03 |
| ENSG00000111981 | **ULBP1** | 50.0961218394875 | 87.9762944684857 | 50.7631107913245 | 108.69179821466 | 142.65904640062 | 111.510219035981 | 62.9451756997659 | 120.953687883754 | -0.940 | 3.79669E-03 |
| ENSG00000166704 | **ZNF606** | 5.11184916729464 | 4.94248845328572 | 11.3958003817259 | 27.1729495536651 | 36.4026532194685 | 23.3157730711596 | 7.15004600076876 | 28.9637919480977 | -2.023 | 3.79936E-03 |
| ENSG00000210144 | **MT-TY** | 77.7001073428786 | 76.1143221806 | 84.9505119365023 | 127.431763424085 | 123.965792044676 | 155.10057738641 | 79.5883138199936 | 135.49937761839 | -0.767 | 3.91537E-03 |
| ENSG00000233067 | **PTCHD1-AS** | 0 | 0 | 0 | 6.55898782329847 | 10.8224104165987 | 7.09610484774423 | 0 | 8.15916769588048 | -5.449 | 4.08147E-03 |
| ENSG00000284648 | **AC097493.4** | 21.4697665026375 | 25.7009399570857 | 30.0434737336411 | 63.7158817120423 | 62.9667515147563 | 51.7001924621366 | 25.7380600644548 | 59.4609418963117 | -1.209 | 4.09168E-03 |
| ENSG00000229209 | **AC073987.1** | 0 | 0 | 0 | 7.49598608376968 | 10.8224104165987 | 6.08237558378077 | 0 | 8.1335906947164 | -5.445 | 4.16275E-03 |
| ENSG00000210195 | **MT-TT** | 164.601543186887 | 156.182635123829 | 204.088425018182 | 246.430542503928 | 233.173751703082 | 323.379635204344 | 174.957534442966 | 267.661309803785 | -0.613 | 4.31357E-03 |
| ENSG00000025423 | **HSD17B6** | 34.7605743376036 | 35.5859168636572 | 41.439274115367 | 72.1488660562832 | 93.4662717797164 | 65.892402157625 | 37.2619217722092 | 77.1691799978748 | -1.051 | 4.37507E-03 |
| ENSG00000143624 | **INTS3** | 409.97030321703 | 386.502597046943 | 340.838029598893 | 1086.9179821466 | 510.621000564977 | 1001.5645127959 | 379.103643287622 | 866.367831835827 | -1.192 | 4.42300E-03 |
| ENSG00000227619 | **AL391056.1** | 1.02236983345893 | 2.96549307197143 | 5.17990926442087 | 16.8659686884818 | 19.6771098483613 | 21.2883145432327 | 3.05592405661708 | 19.2771310266919 | -2.659 | 4.47279E-03 |
| ENSG00000151651 | **ADAM8** | 82.8119565101732 | 82.0453083245429 | 84.9505119365023 | 132.116754726441 | 139.707479923366 | 140.908367690921 | 83.2692589237395 | 137.577534113576 | -0.724 | 4.49235E-03 |
| ENSG00000127561 | **SYNGR3** | 131.885708516202 | 166.0676120304 | 78.7346208191972 | 238.934556420158 | 204.641942422958 | 199.704665000802 | 125.562647121933 | 214.427054614639 | -0.771 | 4.49353E-03 |
| ENSG00000115266 | **APC2** | 215.720034859834 | 180.895077390257 | 139.857550139364 | 292.343457267017 | 245.963873104517 | 275.734359798062 | 178.824220796485 | 271.347230056532 | -0.601 | 4.52045E-03 |
| ENSG00000138449 | **SLC40A1** | 71.565888342125 | 94.8957783030857 | 73.5547115547764 | 146.171728633509 | 153.481456817218 | 114.551406827871 | 80.0054593999957 | 138.068197426199 | -0.787 | 4.56807E-03 |
| novel.678 | **-** | 2.04473966691786 | 5.93098614394286 | 5.17990926442087 | 52.4719025863877 | 8.8546994317626 | 160.169223706227 | 4.38521169176053 | 73.8319419081258 | -4.072 | 4.65528E-03 |
| ENSG00000144843 | **ADPRH** | 107.348832513187 | 95.8842759937429 | 67.3388204374713 | 165.848692103404 | 152.4976013248 | 139.894638426958 | 90.1906429814672 | 152.746977285054 | -0.760 | 4.66076E-03 |
| ENSG00000169894 | **MUC3A** | 20.4473966691786 | 24.7124422664286 | 12.4317822346101 | 49.6609078049741 | 52.1443410981576 | 46.6315461423192 | 19.1972070567391 | 49.478931681817 | -1.363 | 4.69601E-03 |
| ENSG00000180263 | **FGD6** | 123.70674984853 | 160.136625886457 | 160.577187197047 | 220.194591210734 | 228.254474240992 | 220.992979544035 | 148.140187644012 | 223.14734833192 | -0.591 | 4.80539E-03 |
| ENSG00000267868 | **AL356740.1** | 3.06710950037678 | 5.93098614394286 | 6.21589111730505 | 20.6139617303666 | 29.515664772542 | 20.2745852792692 | 5.07132892054156 | 23.4680705940593 | -2.209 | 4.90843E-03 |
| ENSG00000177628 | **GBA** | 11.2460681680482 | 6.9194838346 | 5.17990926442087 | 65.5898782329847 | 23.6125318180336 | 14.1922096954885 | 7.78182042235636 | 34.4648732488356 | -2.151 | 4.96002E-03 |
| ENSG00000156966 | **B3GNT7** | 1.02236983345893 | 2.96549307197143 | 0 | 15.9289704280106 | 15.7416878786891 | 13.178480431525 | 1.32928763514345 | 14.9497129127415 | -3.473 | 5.07611E-03 |
| ENSG00000132932 | **ATP8A2** | 11.2460681680482 | 20.7584515038 | 11.3958003817259 | 47.7869112840317 | 41.3219306815588 | 35.4805242387212 | 14.4667733511914 | 41.5297887347706 | -1.519 | 5.18402E-03 |
| ENSG00000250634 | **LINC01182** | 0 | 0 | 0 | 8.43298434424089 | 9.83855492418067 | 5.06864631981731 | 0 | 7.78006186274629 | -5.381 | 5.35690E-03 |
| ENSG00000101276 | **SLC52A3** | 113.483051513941 | 131.4701928574 | 122.245858640333 | 200.517627740839 | 177.093988635252 | 185.512455305314 | 122.399701003891 | 187.708023893802 | -0.617 | 5.35766E-03 |
| ENSG00000278709 | **NKILA** | 0 | 2.96549307197143 | 2.07196370576835 | 20.6139617303666 | 15.7416878786891 | 11.1510219035981 | 1.67915225924659 | 15.8355571708846 | -3.232 | 5.42590E-03 |
| ENSG00000254526 | **AC090791.1** | 0 | 0 | 1.03598185288417 | 9.3699826047121 | 11.8062659090168 | 12.1647511675615 | 0.345327284294725 | 11.1136665604301 | -4.932 | 5.60377E-03 |
| ENSG00000153563 | **CD8A** | 27.6039855033911 | 20.7584515038 | 19.6836552047993 | 60.9048869306286 | 55.0959075754118 | 45.6178168783558 | 22.6820307373301 | 53.8728704614654 | -1.249 | 5.60377E-03 |
| ENSG00000154930 | **ACSS1** | 56.2303408402411 | 56.3443683674572 | 62.1589111730505 | 94.6368243075922 | 103.304826703897 | 114.551406827871 | 58.2445401269162 | 104.16435261312 | -0.838 | 5.65676E-03 |
| ENSG00000154529 | **CNTNAP3B** | 36.8053140045214 | 43.4938983889143 | 34.1874011451778 | 83.3928451819377 | 60.0151850375021 | 91.2356337567116 | 38.1622045128712 | 78.2145546587171 | -1.034 | 5.75175E-03 |
| ENSG00000205744 | **DENND1C** | 12.2684380015071 | 10.8734745972286 | 4.1439274115367 | 28.1099478141363 | 32.4672312497962 | 34.4667949747577 | 9.09528000342414 | 31.6813246795634 | -1.797 | 5.75969E-03 |
| ENSG00000272674 | **PCDHB16** | 109.393572180105 | 139.378174382657 | 102.562203435533 | 189.273648615184 | 182.99712158976 | 177.402621193606 | 117.111316666099 | 183.224463799517 | -0.645 | 5.77844E-03 |
| ENSG00000136275 | **C7orf69** | 2.04473966691786 | 1.97699538131429 | 3.10794555865252 | 14.9919721675394 | 18.6932543559433 | 16.2196682234154 | 2.37656020229489 | 16.6349649156327 | -2.810 | 5.88493E-03 |
| ENSG00000171951 | **SCG2** | 48.0513821725696 | 81.0568106338857 | 120.173894934564 | 146.171728633509 | 507.669434087723 | 822.134433074368 | 83.0940292470065 | 491.9918652652 | -2.566 | 6.00827E-03 |
| ENSG00000182580 | **EPHB3** | 44.9842726721928 | 45.4708937702286 | 33.1514192922936 | 90.8888312657073 | 72.805306438937 | 80.0846118531135 | 41.202195244905 | 81.2595831859193 | -0.979 | 6.04035E-03 |
| ENSG00000243276 | **AC068633.1** | 0 | 0 | 0 | 7.49598608376968 | 6.88698844692647 | 8.10983411170769 | 0 | 7.49760288080128 | -5.327 | 6.14571E-03 |
| ENSG00000108387 | **SEPT4** | 8.17895866767143 | 4.94248845328572 | 7.25187297018922 | 28.1099478141363 | 26.5640982952878 | 23.3157730711596 | 6.79110669704879 | 25.9966063935279 | -1.940 | 6.29825E-03 |
| ENSG00000238042 | **LINC02257** | 0 | 0 | 0 | 4.68499130235605 | 8.8546994317626 | 9.12356337567116 | 0 | 7.5544180365966 | -5.336 | 6.36188E-03 |
| ENSG00000184371 | **CSF1** | 39.8724235048982 | 46.4593914608857 | 36.2593648509461 | 92.7628277866498 | 71.8214509465189 | 76.0296947972596 | 40.8637266055767 | 80.2046578434761 | -0.973 | 6.63621E-03 |
| ENSG00000101333 | **PLCB4** | 1.02236983345893 | 0 | 0 | 8.43298434424089 | 13.7739768938529 | 10.1372926396346 | 0.340789944486309 | 10.7814179592428 | -4.889 | 6.68023E-03 |
| ENSG00000007516 | **BAIAP3** | 116.550161014318 | 132.458690548057 | 120.173894934564 | 194.895638178012 | 201.690375945704 | 167.265328553971 | 123.06091549898 | 187.950447559229 | -0.611 | 6.77968E-03 |
| ENSG00000136010 | **ALDH1L2** | 65.4316693413714 | 72.1603314179714 | 59.0509656143979 | 129.305759945027 | 123.965792044676 | 92.249363020675 | 65.5476554579136 | 115.173638336793 | -0.814 | 7.17962E-03 |
| ENSG00000278540 | **ACACA** | 346.583373542577 | 487.329361493972 | 275.57117286719 | 627.788834515711 | 910.066330486712 | 673.116231271739 | 369.827969301246 | 736.99046542472 | -0.994 | 7.22942E-03 |
| ENSG00000196517 | **SLC6A9** | 56.2303408402411 | 73.1488291086286 | 78.7346208191972 | 120.872775600786 | 113.143381628078 | 122.661240939579 | 69.3712635893556 | 118.892466056148 | -0.777 | 7.29337E-03 |
| ENSG00000169242 | **EFNA1** | 55.2079710067821 | 43.4938983889143 | 29.0074918807569 | 88.0778364842937 | 86.5792833327899 | 78.0571533251866 | 42.5697870921511 | 84.2380910474234 | -0.984 | 7.50537E-03 |
| ENSG00000168875 | **SOX14** | 0 | 0 | 0 | 6.55898782329847 | 6.88698844692647 | 8.10983411170769 | 0 | 7.18527012731088 | -5.265 | 7.70584E-03 |
| ENSG00000185669 | **SNAI3** | 92.0132850113035 | 105.769252900314 | 54.9070382028612 | 138.675742549739 | 131.836635984021 | 172.333974873789 | 84.2298587048264 | 147.61545113585 | -0.807 | 7.81559E-03 |
| ENSG00000196503 | **ARL9** | 59.2974503406178 | 70.1833360366572 | 29.0074918807569 | 109.628796475132 | 117.07880359775 | 87.1807167008577 | 52.8294260860106 | 104.62943892458 | -0.984 | 7.99904E-03 |
| ENSG00000173406 | **DAB1** | 0 | 0 | 0 | 7.49598608376968 | 4.91927746209034 | 9.12356337567116 | 0 | 7.17960897384372 | -5.264 | 8.10384E-03 |
| ENSG00000247134 | **AC090204.1** | 32.7158346706857 | 19.7699538131429 | 31.0794555865252 | 55.2828973678014 | 63.9506070071744 | 62.8512143657346 | 27.8550813567846 | 60.6949062469035 | -1.125 | 8.39019E-03 |
| ENSG00000224568 | **LINC01886** | 0 | 0 | 0 | 5.62198956282726 | 10.8224104165987 | 5.06864631981731 | 0 | 7.1710154330811 | -5.263 | 8.54529E-03 |
| ENSG00000186891 | **TNFRSF18** | 10.2236983345893 | 7.90798152525714 | 8.2878548230734 | 42.1649217212044 | 20.6609653407794 | 27.3706901270135 | 8.80651156097327 | 30.0655257296658 | -1.774 | 8.55955E-03 |
| ENSG00000106852 | **LHX6** | 71.565888342125 | 89.9532898498 | 84.9505119365023 | 140.549739070681 | 126.917358521931 | 133.812262843177 | 82.1565633761424 | 133.75978681193 | -0.703 | 8.81950E-03 |
| ENSG00000183682 | **BMP8A** | 36.8053140045214 | 28.6664330290571 | 30.0434737336411 | 81.5188486609952 | 60.9990405299202 | 56.7688387819539 | 31.8384069224065 | 66.4289093242898 | -1.063 | 9.05807E-03 |
| ENSG00000077782 | **FGFR1** | 1.02236983345893 | 2.96549307197143 | 1.03598185288417 | 15.9289704280106 | 6.88698844692647 | 22.3020438071962 | 1.67461491943818 | 15.0393342273777 | -3.156 | 9.16664E-03 |
| ENSG00000182752 | **PAPPA** | 0 | 0 | 1.03598185288417 | 5.62198956282726 | 12.7901214014349 | 12.1647511675615 | 0.345327284294725 | 10.1922873772746 | -4.806 | 9.40280E-03 |
| ENSG00000225526 | **MKRN2OS** | 19.4250268357196 | 16.8044607411714 | 10.3598185288417 | 40.290925200262 | 49.1927746209034 | 34.4667949747577 | 15.5297687019109 | 41.316831598641 | -1.411 | 9.44254E-03 |
| ENSG00000119922 | **IFIT2** | 109.393572180105 | 155.194137433171 | 110.850058258607 | 234.249565117802 | 198.73880946845 | 161.18295297019 | 125.145922623961 | 198.057109185481 | -0.662 | 9.75639E-03 |
| ENSG00000036672 | **USP2** | 42.939533005275 | 53.3788752954857 | 34.1874011451778 | 73.0858643167544 | 78.7084393934454 | 100.359197132383 | 43.5019364819795 | 84.0511669475275 | -0.947 | 9.77056E-03 |
| ENSG00000175600 | **SUGCT** | 84.856696177091 | 66.2293452740286 | 73.5547115547764 | 136.801746028797 | 121.014225567422 | 114.551406827871 | 74.8802510019653 | 124.122459474697 | -0.731 | 9.99434E-03 |
| ENSG00000095932 | **SMIM24** | 2.04473966691786 | 1.97699538131429 | 1.03598185288417 | 11.2439791256545 | 17.7093988635252 | 13.178480431525 | 1.68590563370544 | 14.0439528069016 | -3.054 | 1.00686E-02 |
| ENSG00000102003 | **SYP** | 36.8053140045214 | 53.3788752954857 | 36.2593648509461 | 77.7708556191104 | 74.7730174237731 | 90.2219044927481 | 42.1478513836511 | 80.9219258452105 | -0.939 | 1.03639E-02 |
| ENSG00000128422 | **KRT17** | 40.8947933383571 | 6.9194838346 | 27.9715100278727 | 78.7078538795816 | 46.2412081436492 | 76.0296947972596 | 25.2619290669433 | 66.9929189401635 | -1.411 | 1.04588E-02 |
| ENSG00000116819 | **TFAP2E** | 109.393572180105 | 106.757750590971 | 108.778094552838 | 170.53368340576 | 147.57832386271 | 179.430079721533 | 108.309805774638 | 165.847362330001 | -0.615 | 1.05129E-02 |
| ENSG00000236359 | **OR51B8P** | 0 | 0.988497690657143 | 0 | 14.0549739070681 | 2.9515664772542 | 14.1922096954885 | 0.329499230219048 | 10.3995833599369 | -4.838 | 1.05470E-02 |
| ENSG00000198774 | **RASSF9** | 0 | 0 | 0 | 7.49598608376968 | 1.96771098483613 | 12.1647511675615 | 0 | 7.20948274538912 | -5.269 | 1.06474E-02 |
| ENSG00000223813 | **AC007255.1** | 3.06710950037678 | 2.96549307197143 | 5.17990926442087 | 25.2989530327227 | 22.6286763256155 | 10.1372926396346 | 3.7375039455897 | 19.3549739993243 | -2.378 | 1.06717E-02 |
| ENSG00000135116 | **HRK** | 20.4473966691786 | 19.7699538131429 | 18.6476733519151 | 45.9129147630893 | 51.1604856057395 | 41.5628998225019 | 19.6216746114122 | 46.2121000637769 | -1.236 | 1.07388E-02 |
| ENSG00000231056 | **LINC02522** | 5.11184916729464 | 7.90798152525714 | 3.10794555865252 | 34.6689356374348 | 15.7416878786891 | 19.2608560153058 | 5.3759254170681 | 23.2238265104765 | -2.108 | 1.07512E-02 |
| ENSG00000146666 | **LINC00525** | 0 | 0.988497690657143 | 0 | 6.55898782329847 | 13.7739768938529 | 9.12356337567116 | 0.329499230219048 | 9.81884269760752 | -4.753 | 1.07832E-02 |
| ENSG00000254285 | **KRT8P3** | 83.8343263436321 | 55.3558706768 | 87.0224756422706 | 114.313787777488 | 126.917358521931 | 141.922096954885 | 75.4042242209009 | 127.717747751434 | -0.761 | 1.09121E-02 |
| ENSG00000019186 | **CYP24A1** | 0 | 0 | 0 | 6.55898782329847 | 9.83855492418067 | 4.05491705585385 | 0 | 6.817486601111 | -5.191 | 1.09193E-02 |
| ENSG00000136274 | **NACAD** | 104.281723012811 | 95.8842759937429 | 105.670148994186 | 167.722688624347 | 174.142422157998 | 132.798533579214 | 101.945382666913 | 158.221214787186 | -0.635 | 1.25790E-02 |
| ENSG00000203697 | **CAPN8** | 1.02236983345893 | 3.95399076262857 | 4.1439274115367 | 15.9289704280106 | 13.7739768938529 | 21.2883145432327 | 3.0400960025414 | 16.9970872883654 | -2.481 | 1.30566E-02 |
| novel.49 | **-** | 21.4697665026375 | 3.95399076262857 | 15.5397277932626 | 35.605933897906 | 41.3219306815588 | 42.5766290864654 | 13.6544950195096 | 39.8348312219767 | -1.549 | 1.33077E-02 |
| ENSG00000139445 | **FOXN4** | 12.2684380015071 | 14.8274653598571 | 9.32383667595757 | 34.6689356374348 | 23.6125318180336 | 46.6315461423192 | 12.139913345774 | 34.9710045325959 | -1.523 | 1.36942E-02 |
| ENSG00000092929 | **UNC13D** | 32.7158346706857 | 50.4133822235143 | 50.7631107913245 | 101.195812130891 | 80.6761503782815 | 70.9610484774423 | 44.6307758951748 | 84.2776703288715 | -0.918 | 1.38911E-02 |
| ENSG00000123201 | **GUCY1B2** | 25.5592458364732 | 19.7699538131429 | 21.7556189105677 | 50.5979060654453 | 55.0959075754118 | 43.5903583504289 | 22.3616061867279 | 49.761390663762 | -1.155 | 1.38960E-02 |
| ENSG00000107968 | **MAP3K8** | 138.019927516955 | 99.8382667563715 | 94.2743486124599 | 175.218674708116 | 173.15856666558 | 163.210411498117 | 110.710847628596 | 170.529217623938 | -0.624 | 1.45726E-02 |
| ENSG00000104643 | **MTMR9** | 40.8947933383571 | 35.5859168636572 | 41.439274115367 | 53.408900846859 | 87.563138825208 | 89.2081752287846 | 39.3066614391271 | 76.7267383002839 | -0.964 | 1.47981E-02 |
| ENSG00000104818 | **CGB2** | 6.13421900075357 | 2.96549307197143 | 5.17990926442087 | 24.3619547722515 | 16.7255433711071 | 19.2608560153058 | 4.75987377904862 | 20.1161180528881 | -2.084 | 1.49663E-02 |
| ENSG00000125968 | **ID1** | 200.38448735795 | 192.757049678143 | 381.241321861376 | 521.908031082464 | 476.186058330345 | 668.047584951921 | 258.12761963249 | 555.380558121577 | -1.106 | 1.53660E-02 |
| ENSG00000262580 | **AC087741.1** | 32.7158346706857 | 34.597419173 | 20.7196370576835 | 54.3458991073302 | 63.9506070071744 | 63.8649436296981 | 29.3442969671231 | 60.7204832480675 | -1.047 | 1.56056E-02 |
| ENSG00000184845 | **DRD1** | 17.3802871688018 | 21.7469491944571 | 21.7556189105677 | 48.7239095445029 | 43.289641666395 | 45.6178168783558 | 20.2942850912755 | 45.8771226964179 | -1.177 | 1.62662E-02 |
| ENSG00000188385 | **JAKMIP3** | 0 | 0 | 3.10794555865252 | 11.2439791256545 | 9.83855492418067 | 15.2059389594519 | 1.03598185288417 | 12.0961576697624 | -3.565 | 1.63545E-02 |
| ENSG00000146072 | **TNFRSF21** | 1168.56871964355 | 1029.02609597409 | 1702.1181842887 | 2018.29425305499 | 2102.49918729741 | 2404.56581412133 | 1299.90433330211 | 2175.11975149124 | -0.743 | 1.65536E-02 |
| ENSG00000272703 | **AP005137.2** | 0 | 0 | 0 | 5.62198956282726 | 3.93542196967227 | 9.12356337567116 | 0 | 6.2269916360569 | -5.058 | 1.68738E-02 |
| ENSG00000233006 | **MIR3936HG** | 62.3645598409946 | 90.9417875404572 | 72.5187297018922 | 122.746772121728 | 121.014225567422 | 123.674970203542 | 75.275025694448 | 122.478655964231 | -0.701 | 1.68738E-02 |
| ENSG00000044459 | **CNTLN** | 0 | 0 | 0 | 6.55898782329847 | 7.87084393934454 | 4.05491705585385 | 0 | 6.16158293949895 | -5.045 | 1.71236E-02 |
| ENSG00000227745 | **AC098826.2** | 1.02236983345893 | 0 | 1.03598185288417 | 8.43298434424089 | 12.7901214014349 | 11.1510219035981 | 0.686117228781034 | 10.7913758830913 | -3.990 | 1.73119E-02 |
| ENSG00000278920 | **AC005005.4** | 0 | 0 | 0 | 2.81099478141363 | 8.8546994317626 | 7.09610484774423 | 0 | 6.25393302030682 | -5.063 | 1.75671E-02 |
| ENSG00000235576 | **LINC01871** | 0 | 0.988497690657143 | 1.03598185288417 | 12.1809773861257 | 7.87084393934454 | 12.1647511675615 | 0.674826514513773 | 10.7388574976773 | -3.989 | 1.77748E-02 |
| ENSG00000128165 | **ADM2** | 23.5145061695554 | 43.4938983889143 | 21.7556189105677 | 68.4008730143983 | 57.0636185602479 | 60.8237558378077 | 29.5880078230124 | 62.096082470818 | -1.067 | 1.82425E-02 |
| ENSG00000274173 | **AL035661.1** | 0 | 0 | 0 | 9.3699826047121 | 3.93542196967227 | 5.06864631981731 | 0 | 6.12468363140056 | -5.038 | 1.83071E-02 |
| ENSG00000257084 | **MIR200CHG** | 42.939533005275 | 18.7814561224857 | 46.6191833797878 | 86.2038399633513 | 64.9344624995924 | 69.9473192134789 | 36.1133908358495 | 73.6952072254742 | -1.033 | 1.84316E-02 |
| ENSG00000215039 | **CD27-AS1** | 73.6106280090428 | 98.8497690657143 | 88.0584574951548 | 153.667714717278 | 113.143381628078 | 149.018201802629 | 86.8396181899707 | 138.609766049328 | -0.674 | 1.85524E-02 |
| ENSG00000106714 | **CNTNAP3** | 17.3802871688018 | 14.8274653598571 | 7.25187297018922 | 40.290925200262 | 26.5640982952878 | 40.5491705585385 | 13.153208499616 | 35.8013980180294 | -1.443 | 1.90683E-02 |
| ENSG00000169432 | **SCN9A** | 52.1408615064053 | 89.9532898498 | 59.0509656143979 | 129.305759945027 | 130.852780491603 | 90.2219044927481 | 67.0483723235344 | 116.793481643126 | -0.800 | 1.91051E-02 |
| ENSG00000174527 | **MYO1H** | 21.4697665026375 | 27.6779353384 | 33.1514192922936 | 71.2118677958119 | 53.1281965905756 | 47.6452754062827 | 27.433040377777 | 57.3284465975568 | -1.066 | 1.94219E-02 |
| ENSG00000213420 | **GPC2** | 107.348832513187 | 101.815262137686 | 97.3822941711124 | 126.494765163613 | 173.15856666558 | 171.320245609825 | 102.182129607329 | 156.991192479673 | -0.618 | 1.96038E-02 |
| ENSG00000236204 | **LINC01376** | 19.4250268357196 | 26.6894376477429 | 15.5397277932626 | 49.6609078049741 | 42.3057861739769 | 46.6315461423192 | 20.551397425575 | 46.1994133737567 | -1.166 | 2.06286E-02 |
| ENSG00000151715 | **TMEM45B** | 0 | 0 | 0 | 5.62198956282726 | 6.88698844692647 | 5.06864631981731 | 0 | 5.85920810985701 | -4.971 | 2.07409E-02 |
| ENSG00000124466 | **LYPD3** | 62.3645598409946 | 51.4018799141714 | 63.1948930259346 | 133.053752986912 | 100.353260226643 | 76.0296947972596 | 58.9871109270336 | 103.145569336938 | -0.809 | 2.08820E-02 |
| ENSG00000111319 | **SCNN1A** | 53.1632313398643 | 46.4593914608857 | 40.4032922624828 | 92.7628277866498 | 81.6600058706996 | 74.0022362693327 | 46.6753050210776 | 82.8083566422274 | -0.828 | 2.09383E-02 |
| ENSG00000166206 | **GABRB3** | 0 | 0 | 0 | 3.74799304188484 | 7.87084393934454 | 6.08237558378077 | 0 | 5.90040418833672 | -4.980 | 2.10767E-02 |
| ENSG00000224294 | **PINCR** | 0 | 0 | 0 | 11.2439791256545 | 2.9515664772542 | 4.05491705585385 | 0 | 6.08348755292086 | -5.029 | 2.12872E-02 |
| ENSG00000248698 | **LINC01085** | 0 | 0 | 0 | 3.74799304188484 | 4.91927746209034 | 9.12356337567116 | 0 | 5.93027795988211 | -4.986 | 2.13423E-02 |
| ENSG00000180818 | **HOXC10** | 103.259353179352 | 141.355169763971 | 91.1664030538073 | 167.722688624347 | 176.110133142834 | 167.265328553971 | 111.926975332377 | 170.366050107051 | -0.604 | 2.14296E-02 |
| ENSG00000272795 | **AC126283.1** | 0 | 0 | 0 | 7.49598608376968 | 4.91927746209034 | 5.06864631981731 | 0 | 5.82796995522577 | -4.965 | 2.14797E-02 |
| ENSG00000111344 | **RASAL1** | 121.662010181612 | 129.493197476086 | 93.2383667595757 | 194.895638178012 | 189.884110036687 | 140.908367690921 | 114.797858139091 | 175.22937196854 | -0.610 | 2.18364E-02 |
| ENSG00000210140 | **MT-TC** | 50.0961218394875 | 49.4248845328572 | 46.6191833797878 | 73.0858643167544 | 87.563138825208 | 94.276821548602 | 48.7133965840442 | 84.9752748968548 | -0.801 | 2.22269E-02 |
| ENSG00000162654 | **GBP4** | 0 | 2.96549307197143 | 2.07196370576835 | 7.49598608376968 | 19.6771098483613 | 12.1647511675615 | 1.67915225924659 | 13.1126156998975 | -2.957 | 2.24961E-02 |
| ENSG00000114812 | **VIPR1** | 25.5592458364732 | 25.7009399570857 | 17.611691499031 | 46.8499130235605 | 55.0959075754118 | 45.6178168783558 | 22.9572924308633 | 49.1878791591093 | -1.098 | 2.26994E-02 |
| ENSG00000153093 | **ACOXL** | 0 | 0.988497690657143 | 2.07196370576835 | 14.0549739070681 | 6.88698844692647 | 12.1647511675615 | 1.0201537988085 | 11.0355711738521 | -3.441 | 2.28646E-02 |
| ENSG00000215915 | **ATAD3C** | 6.13421900075357 | 6.9194838346 | 13.4677640874943 | 29.0469460746075 | 23.6125318180336 | 28.3844193909769 | 8.84048897428261 | 27.0146324278727 | -1.615 | 2.29844E-02 |
| ENSG00000271138 | **IGLVIVOR22-1** | 0 | 0.988497690657143 | 0 | 17.802966948953 | 6.88698844692647 | 2.02745852792692 | 0.329499230219048 | 8.90580464126879 | -4.618 | 2.33020E-02 |
| ENSG00000109321 | **AREG** | 401.791344549359 | 214.5039988726 | 551.142345734381 | 926.691279606026 | 770.358850563347 | 880.930730384248 | 389.145896385447 | 859.326953517874 | -1.143 | 2.45080E-02 |
| ENSG00000196408 | **NOXO1** | 22.4921363360964 | 35.5859168636572 | 14.5037459403784 | 57.1568938887438 | 44.273497158813 | 57.7825680459173 | 24.1939330467107 | 53.0709863644914 | -1.130 | 2.51246E-02 |
| ENSG00000163219 | **ARHGAP25** | 11.2460681680482 | 22.7354468851143 | 10.3598185288417 | 34.6689356374348 | 37.3865087118865 | 39.535441294575 | 14.7804445273347 | 37.1969618812988 | -1.327 | 2.61036E-02 |
| ENSG00000172350 | **ABCG4** | 44.9842726721928 | 68.2063406553429 | 39.3673104095986 | 93.699826047121 | 83.6277168555357 | 91.2356337567116 | 50.8526412457114 | 89.5210588864561 | -0.814 | 2.62566E-02 |
| ENSG00000138400 | **MDH1B** | 27.6039855033911 | 37.5629122449714 | 44.5472196740195 | 76.8338573586392 | 66.9021734844286 | 61.8374851017712 | 36.5713724741273 | 68.5245053149463 | -0.907 | 2.68430E-02 |
| ENSG00000124507 | **PACSIN1** | 0 | 0 | 0 | 6.55898782329847 | 3.93542196967227 | 6.08237558378077 | 0 | 5.52559512558384 | -4.887 | 2.73347E-02 |
| ENSG00000219061 | **TRIM51FP** | 1.02236983345893 | 0 | 1.03598185288417 | 10.3069808651833 | 11.8062659090168 | 7.09610484774423 | 0.686117228781034 | 9.73645054064811 | -3.843 | 2.76732E-02 |
| ENSG00000130635 | **COL5A1** | 0 | 0 | 0 | 7.49598608376968 | 4.91927746209034 | 4.05491705585385 | 0 | 5.49006020057129 | -4.879 | 2.83044E-02 |
| ENSG00000108924 | **HLF** | 10.2236983345893 | 6.9194838346 | 18.6476733519151 | 36.5429321583772 | 28.5318092801239 | 31.4256071828673 | 11.9302851737015 | 32.1667828737895 | -1.436 | 2.86635E-02 |
| ENSG00000198846 | **TOX** | 2.04473966691786 | 0 | 2.07196370576835 | 15.9289704280106 | 9.83855492418067 | 8.10983411170769 | 1.37223445756207 | 11.292453154633 | -3.056 | 3.05112E-02 |
| ENSG00000086548 | **CEACAM6** | 0 | 0 | 0 | 7.49598608376968 | 1.96771098483613 | 7.09610484774423 | 0 | 5.51993397211668 | -4.886 | 3.12617E-02 |
| ENSG00000135373 | **EHF** | 1.02236983345893 | 0 | 4.1439274115367 | 13.1179756465969 | 8.8546994317626 | 15.2059389594519 | 1.72209908166521 | 12.3928713459372 | -2.862 | 3.13537E-02 |
| ENSG00000224166 | **PRYP1** | 7.1565888342125 | 1.97699538131429 | 4.1439274115367 | 21.5509599908378 | 11.8062659090168 | 21.2883145432327 | 4.42583720902116 | 18.2151801476958 | -2.046 | 3.18200E-02 |
| ENSG00000145103 | **ILDR1** | 10.2236983345893 | 15.8159630505143 | 8.2878548230734 | 40.290925200262 | 25.5802428028697 | 26.35696086305 | 11.4425054027257 | 30.7427096220606 | -1.424 | 3.28338E-02 |
| ENSG00000279741 | **AC007342.8** | 2.04473966691786 | 4.94248845328572 | 3.10794555865252 | 14.9919721675394 | 13.7739768938529 | 17.2333974873789 | 3.36505789295203 | 15.333115516257 | -2.183 | 3.34604E-02 |
| ENSG00000134962 | **KLB** | 0 | 0 | 0 | 4.68499130235605 | 5.9031329545084 | 5.06864631981731 | 0 | 5.21892352556059 | -4.804 | 3.37732E-02 |
| ENSG00000229931 | **AL137003.1** | 15.3355475018839 | 21.7469491944571 | 18.6476733519151 | 42.1649217212044 | 32.4672312497962 | 49.6727339342096 | 18.5767233494187 | 41.4349623017368 | -1.156 | 3.39130E-02 |
| novel.777 | **-** | 1.02236983345893 | 0.988497690657143 | 1.03598185288417 | 4.68499130235605 | 31.4833757573781 | 1.01372926396346 | 1.01561645900008 | 12.3940321078992 | -3.609 | 3.40526E-02 |
| ENSG00000210117 | **MT-TW** | 25.5592458364732 | 29.6549307197143 | 32.1154374394094 | 63.7158817120423 | 46.2412081436492 | 58.7962973098808 | 29.1098713318656 | 56.2511290551907 | -0.951 | 3.52192E-02 |
| ENSG00000200087 | **SNORA73B** | 73.6106280090428 | 71.1718337273143 | 121.209876787448 | 131.179756465969 | 149.546034847546 | 137.867179899031 | 88.6641128412685 | 139.530990404182 | -0.655 | 3.54511E-02 |
| ENSG00000275578 | **AC007742.2** | 9.20132850113035 | 11.8619722878857 | 6.21589111730505 | 36.5429321583772 | 22.6286763256155 | 20.2745852792692 | 9.09306396877371 | 26.482064587754 | -1.542 | 3.61326E-02 |
| ENSG00000233559 | **LINC00513** | 41.9171631718161 | 42.5054006982572 | 72.5187297018922 | 90.8888312657073 | 82.6438613631176 | 97.3180093404923 | 52.3137645239885 | 90.2835673231058 | -0.789 | 3.61584E-02 |
| ENSG00000068831 | **RASGRP2** | 18.4026570022607 | 19.7699538131429 | 16.5757096461468 | 53.408900846859 | 35.4187977270504 | 33.4530657107942 | 18.2494401538501 | 40.7602547615679 | -1.161 | 3.63203E-02 |
| ENSG00000272695 | **GAS6-AS2** | 1.02236983345893 | 2.96549307197143 | 4.1439274115367 | 17.802966948953 | 9.83855492418067 | 14.1922096954885 | 2.71059677232235 | 13.9445771895407 | -2.365 | 3.68379E-02 |
| ENSG00000167588 | **GPD1** | 19.4250268357196 | 25.7009399570857 | 15.5397277932626 | 48.7239095445029 | 33.4510867422143 | 49.6727339342096 | 20.221898195356 | 43.9492434069756 | -1.118 | 3.75177E-02 |
| ENSG00000115041 | **KCNIP3** | 16.3579173353429 | 17.7929584318286 | 8.2878548230734 | 31.8579408560211 | 34.4349422346324 | 36.4942535026846 | 14.1462435300816 | 34.262378864446 | -1.273 | 3.93142E-02 |
| ENSG00000267077 | **AC020663.2** | 4.08947933383571 | 2.96549307197143 | 4.1439274115367 | 19.6769634698954 | 13.7739768938529 | 13.178480431525 | 3.73296660578128 | 15.5431402650911 | -2.062 | 4.09343E-02 |
| ENSG00000080493 | **SLC4A4** | 61.3421900075357 | 84.0223037058572 | 77.6986389663131 | 98.384817349477 | 125.933503029513 | 124.688699467506 | 74.354377559902 | 116.335673282165 | -0.644 | 4.18188E-02 |
| ENSG00000118777 | **ABCG2** | 13.2908078349661 | 18.7814561224857 | 19.6836552047993 | 39.3539269397908 | 44.273497158813 | 31.4256071828673 | 17.2519730540837 | 38.351010427157 | -1.153 | 4.31296E-02 |
| ENSG00000100100 | **PIK3IP1** | 25.5592458364732 | 24.7124422664286 | 14.5037459403784 | 48.7239095445029 | 35.4187977270504 | 52.7139217261 | 21.5918113477601 | 45.6188763325511 | -1.077 | 4.36940E-02 |
| ENSG00000260401 | **AP002761.4** | 5.11184916729464 | 6.9194838346 | 2.07196370576835 | 17.802966948953 | 16.7255433711071 | 18.2471267513423 | 4.70109890255433 | 17.5918790238008 | -1.898 | 4.38821E-02 |
| ENSG00000233834 | **AC005083.1** | 12.2684380015071 | 13.8389676692 | 5.17990926442087 | 32.7949391164923 | 19.6771098483613 | 33.4530657107942 | 10.429104978376 | 28.6417048918826 | -1.454 | 4.39027E-02 |
| ENSG00000184545 | **DUSP8** | 30.6710950037678 | 28.6664330290571 | 13.4677640874943 | 88.0778364842937 | 45.2573526512311 | 31.4256071828673 | 24.2684307067731 | 54.920265439464 | -1.180 | 4.46407E-02 |
| ENSG00000164690 | **SHH** | 1.02236983345893 | 1.97699538131429 | 9.32383667595757 | 16.8659686884818 | 17.7093988635252 | 17.2333974873789 | 4.10773396357693 | 17.2695883464619 | -2.080 | 4.60946E-02 |
| ENSG00000138646 | **HERC5** | 21.4697665026375 | 23.7239445757714 | 9.32383667595757 | 60.9048869306286 | 27.5479537877059 | 39.535441294575 | 18.1725159181222 | 42.6627606709698 | -1.230 | 4.65906E-02 |
| ENSG00000184697 | **CLDN6** | 14.313177668425 | 13.8389676692 | 13.4677640874943 | 31.8579408560211 | 33.4510867422143 | 31.4256071828673 | 13.8733031417064 | 32.2448782603676 | -1.217 | 4.74733E-02 |
| ENSG00000255550 | **AP006587.5** | 1.02236983345893 | 1.97699538131429 | 1.03598185288417 | 13.1179756465969 | 6.88698844692647 | 10.1372926396346 | 1.34511568921913 | 10.0474189110527 | -2.896 | 4.78007E-02 |
| ENSG00000276386 | **CNTNAP3P2** | 36.8053140045214 | 43.4938983889143 | 45.5832015269037 | 63.7158817120423 | 87.563138825208 | 66.9061314215885 | 41.9608046401131 | 72.7283839862796 | -0.793 | 4.79175E-02 |
| ENSG00000125895 | **TMEM74B** | 62.3645598409946 | 85.0108013965143 | 78.7346208191972 | 99.3218156099482 | 124.949647537095 | 124.688699467506 | 75.3699940189021 | 116.32005420485 | -0.624 | 4.93935E-02 |

**Supplementary Fig. 1**


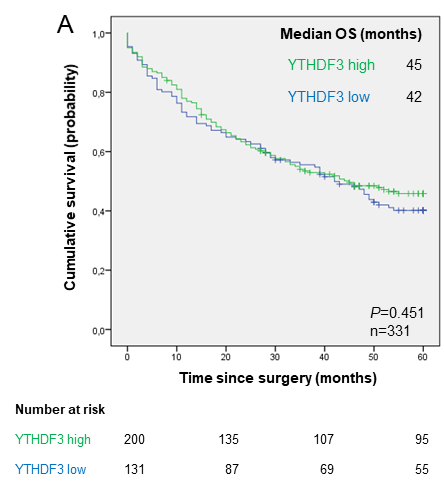


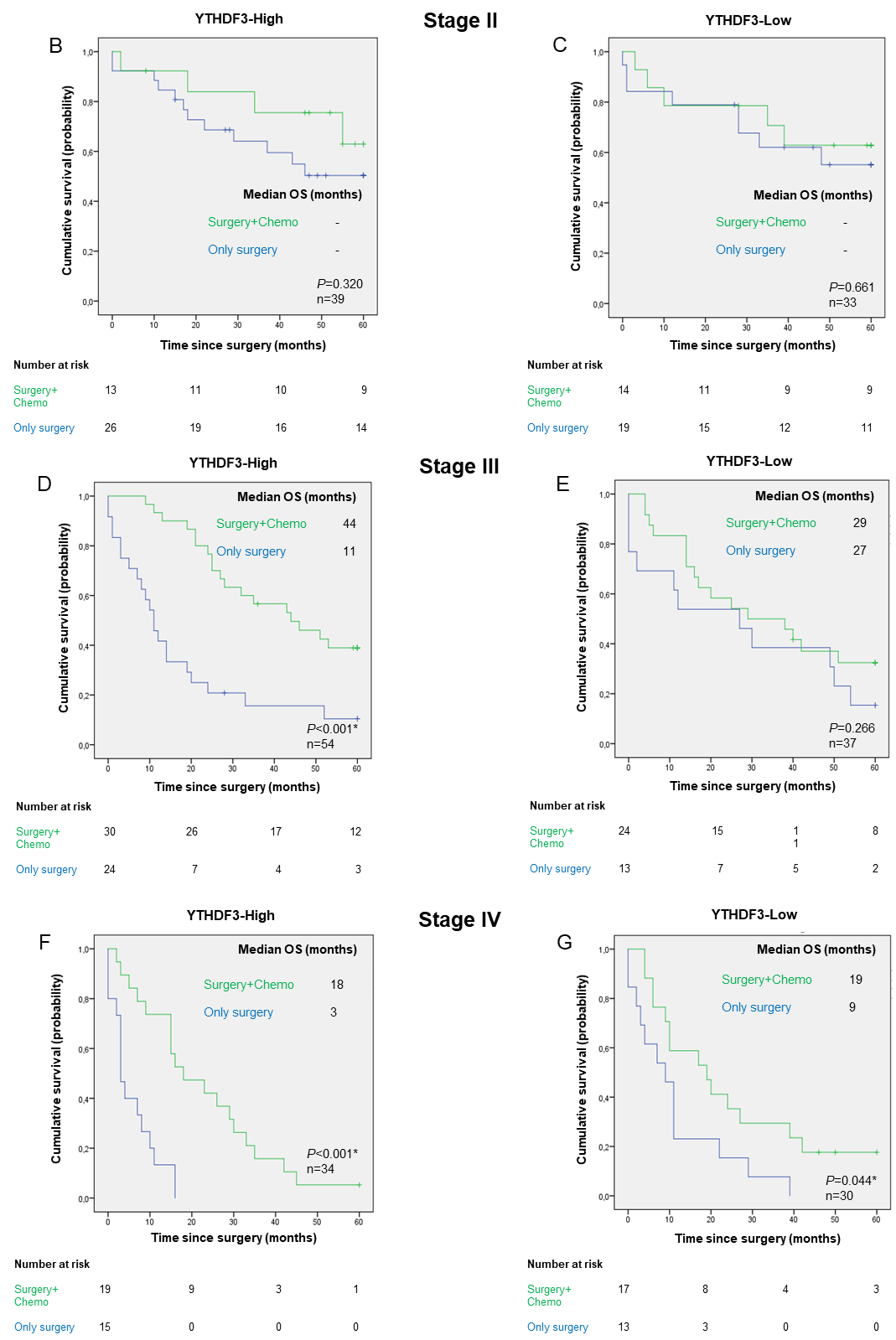


**Supplementary Fig. S1.** Kaplan-Meier curves showing the probability of OS according to **A**. YTHDF3 expression and according to **B, C, D, E, F, and G**. treatment options in patients having High and Low expression of YTHDF3, and stratified by stage. Median OS is shown for each subgroup of patients. The log-rank test was used to test for differences in OS between patients subjected only to surgery versus the ones treated with chemotherapy after surgery. * *p*<0.05. The number of patients at risk is specified for 0, 20, 40, and 60 months.

**Supplementary Fig. 2**

**A**

**
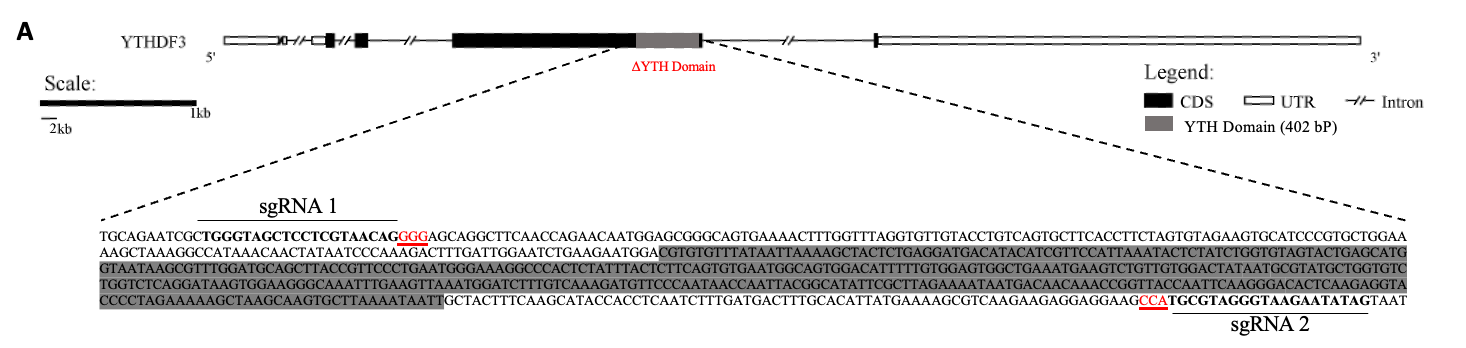
**

**C**

**
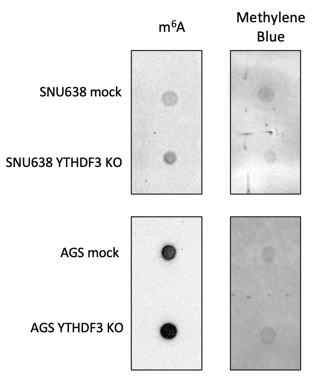
**

**B**


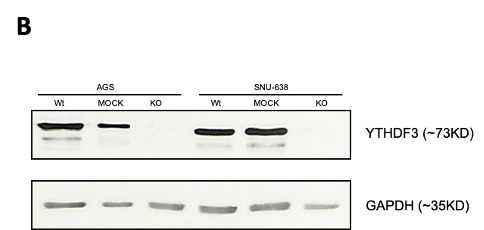


**
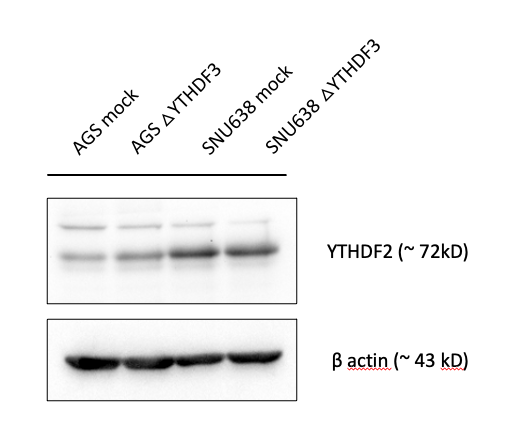

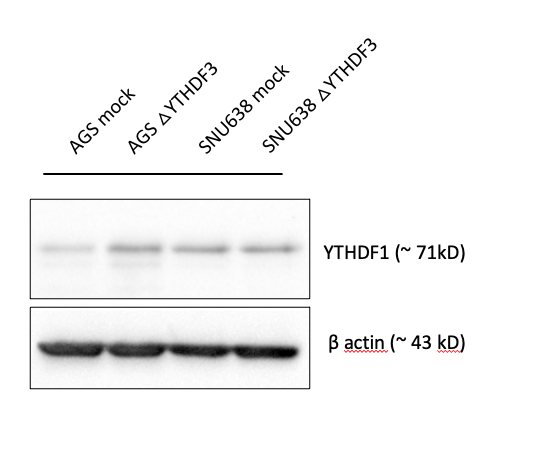
**
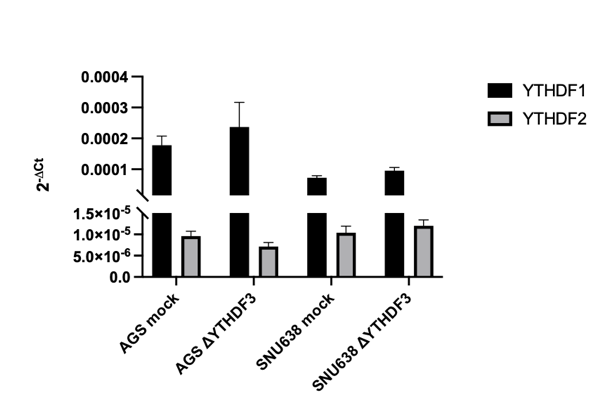


**E**

**D**

**Supplementary Fig. S2. CRISPR-Cas9 ΔYTHDF3 cell lines generation and validation. A.** Localization of the sgRNAs, flanking the genomic sequence that codifies the functional YTH domain (deletion is of 601bp). **B.** Western blot showing the efficient KO of *YTHDF3* in AGS and SNU638 cell lines. **C**. m^6^A dot blot in AGS mock, AGS *ΔYTHDF3*, SNU638 mock and SNU638 *ΔYTHDF3* cell lines. Staining with Methylene Blue was used as an RNA loading control. **D**. qRT-PCR of YTHDF1 and YTHDF2. **E.** Protein expression detection of YTHDF1 and YTHDF2 by Western Blot in generated cell lines.

**Supplementary Fig. 3**

**A**

**B**


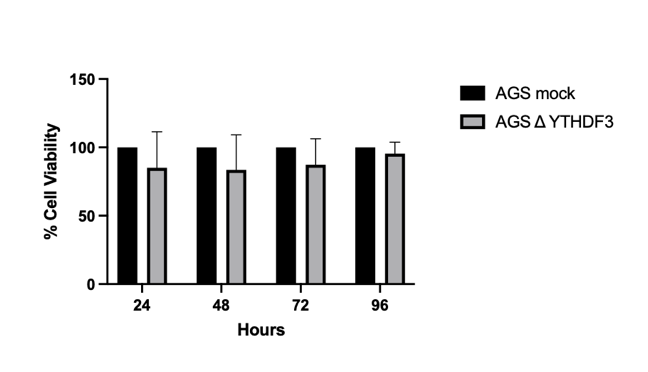
**
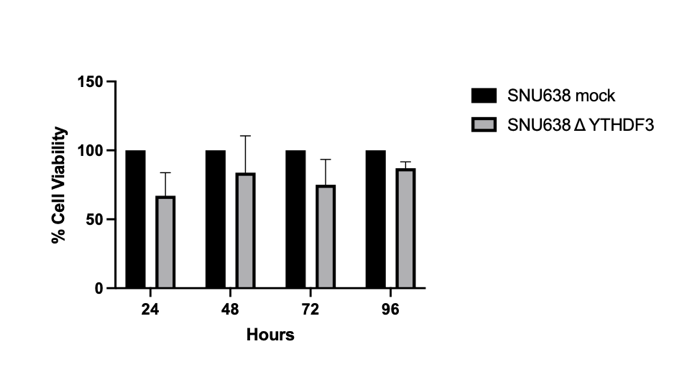
**

**
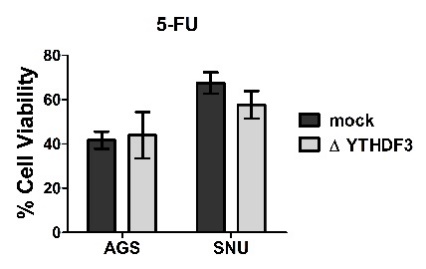

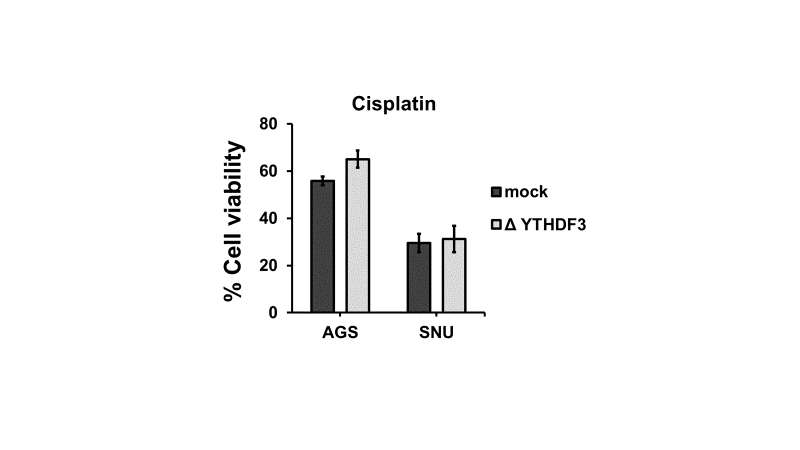

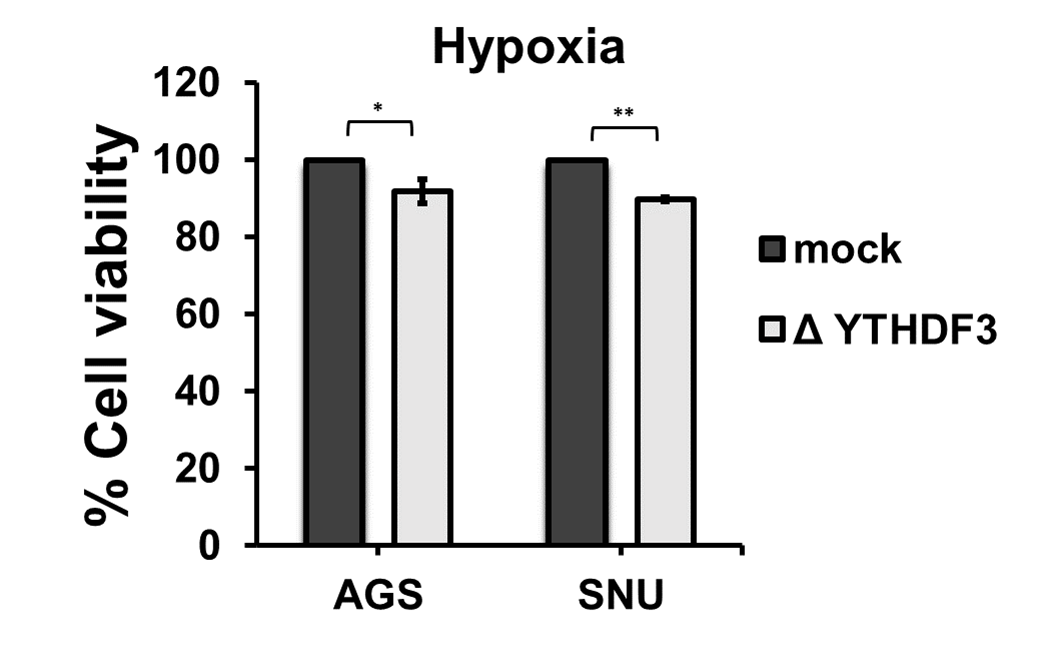
**

**E**

**D**

**C**

**
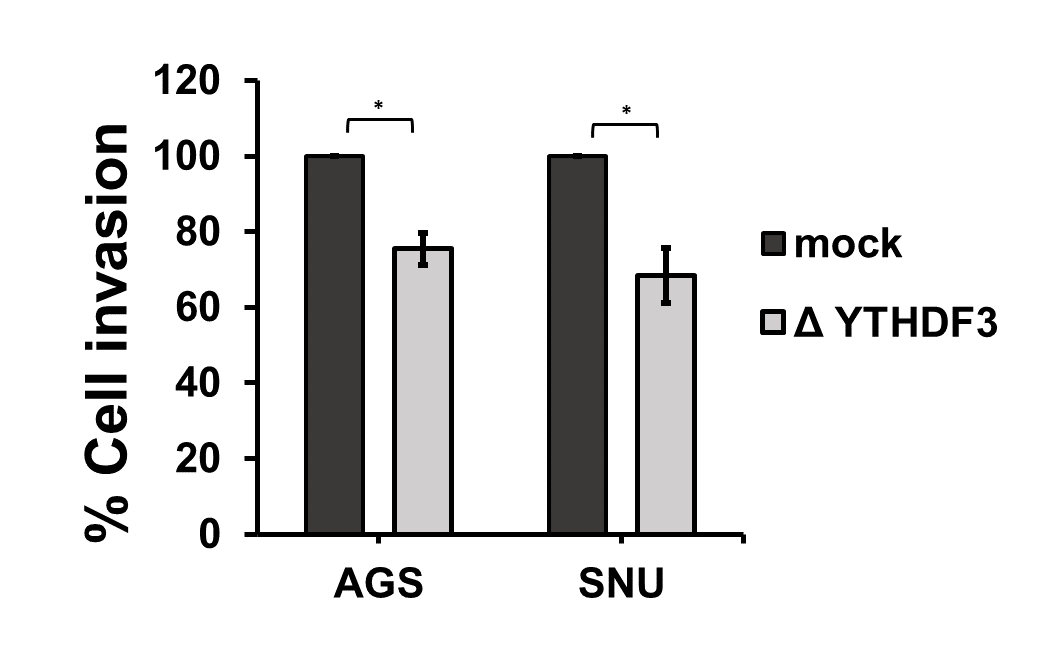
**

**
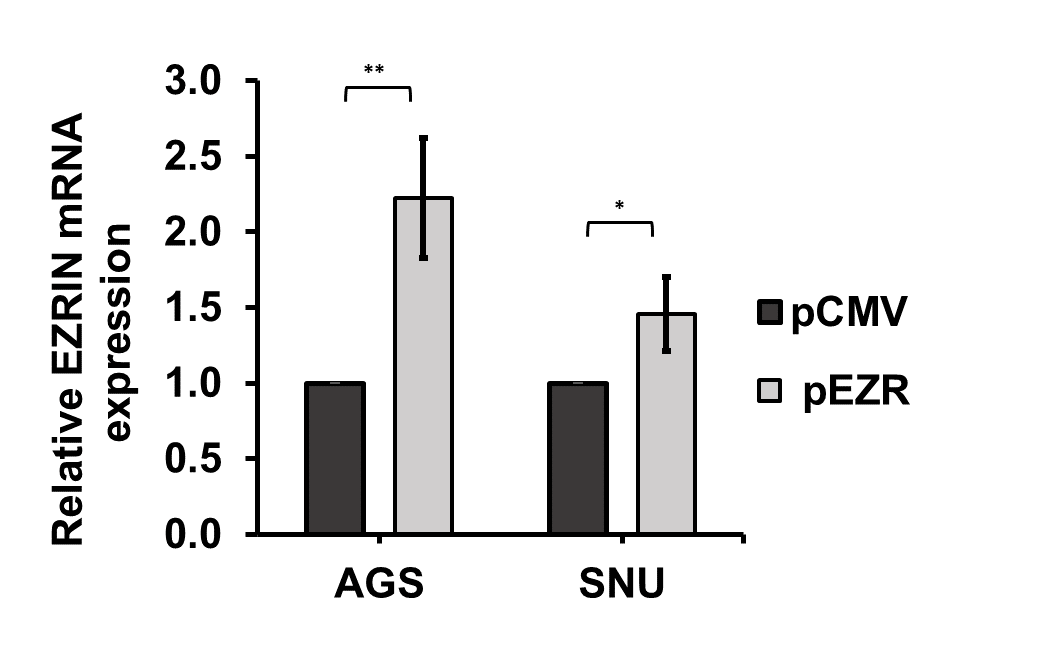

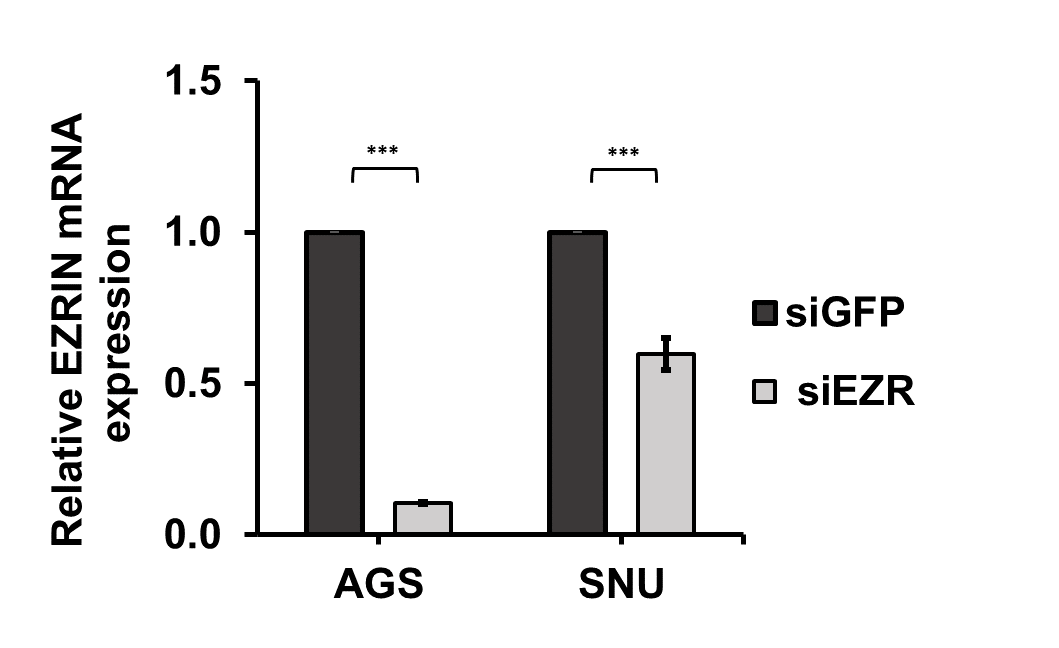
**

**H**

**G**

**F**

**Supplementary Fig. S3**. Effect of YTHDF3 KO in cell viability. **A and B.** Percentage of cell viability in AGS *ΔYTHDF3* vs AGS mock, SNU638 *ΔYTHDF3* vs SNU638 mock. Viability of mock cells was considered as 100% in each time-point. **C.** Percentage of cell viability in AGS *ΔYTHDF3* vs AGS mock, SNU638 *ΔYTHDF3* vs SNU638 mock under hypoxia (1% O_2_), in a hypoxic chamber. **D and E.** Percentage of cell viability evaluated in the same cells after treatment with 4.5 μg/mL 5-FU and 6μg/mL Cisplatin. DMSO was used as a negative control. **F.** Quantification of the transwell invasion capacity evaluated for the same cells. **G and H.** Relative levels of *EZR* assessed by real-time PCR after overexpression of EZR in AGS *ΔYTHDF3* and SNU638 *ΔYTHDF3* cells and after silencing EZR in AGS and SNU638 cells*.* **p*<0.05; ** *p*<0.01; ** *p*<0.001.

**Supplementary Fig. 4**

**A**

**
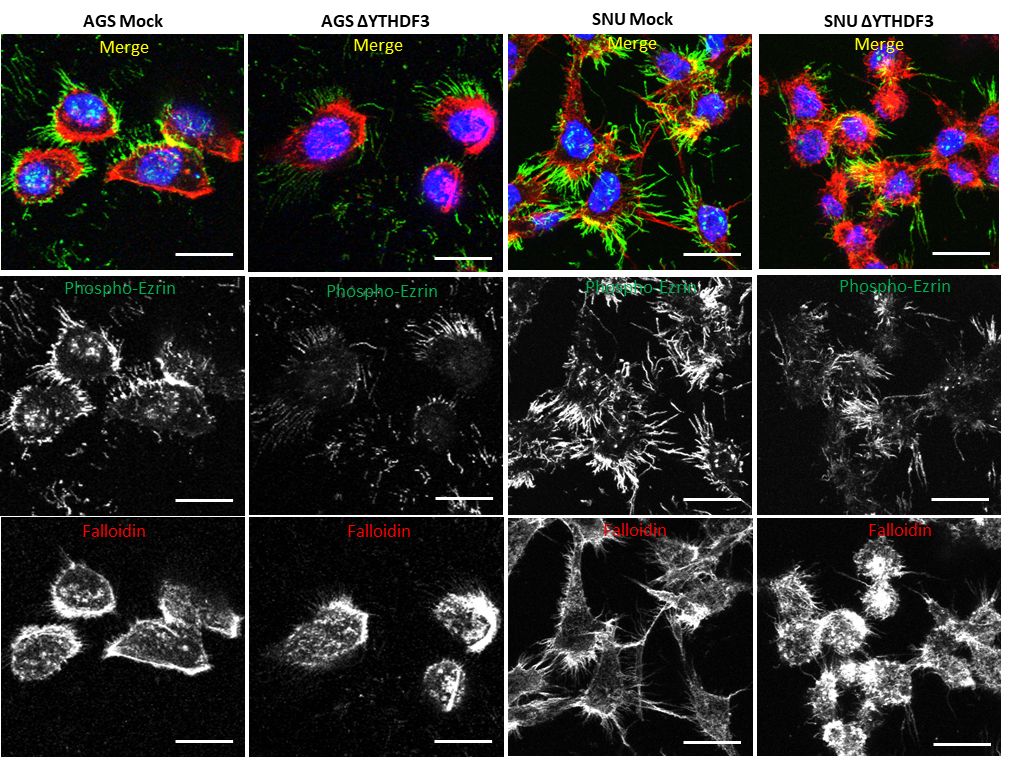
**

**B**


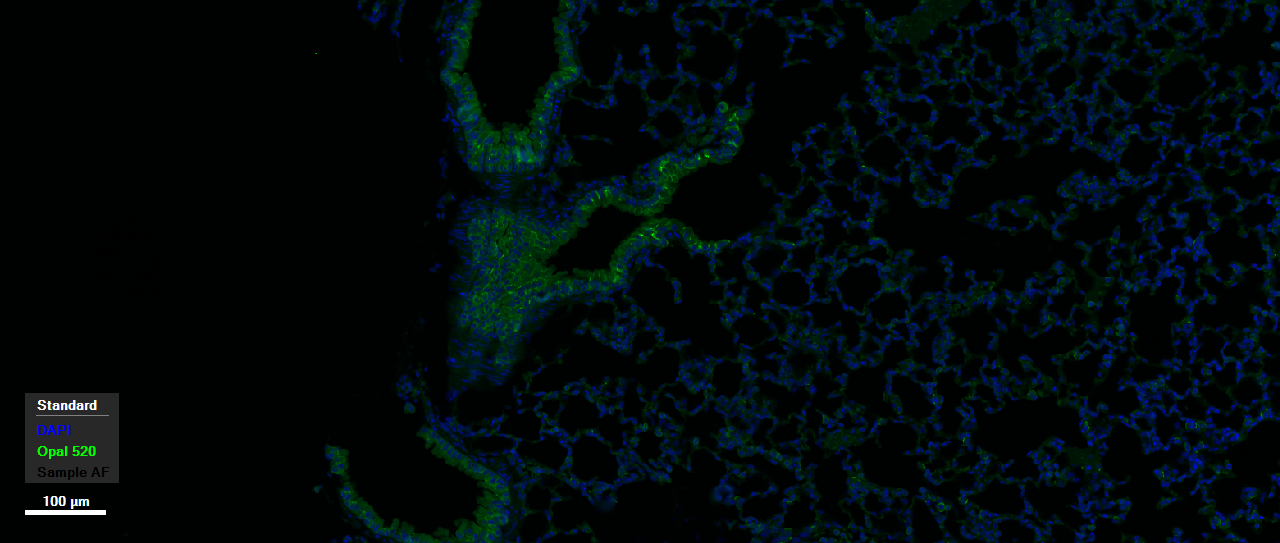

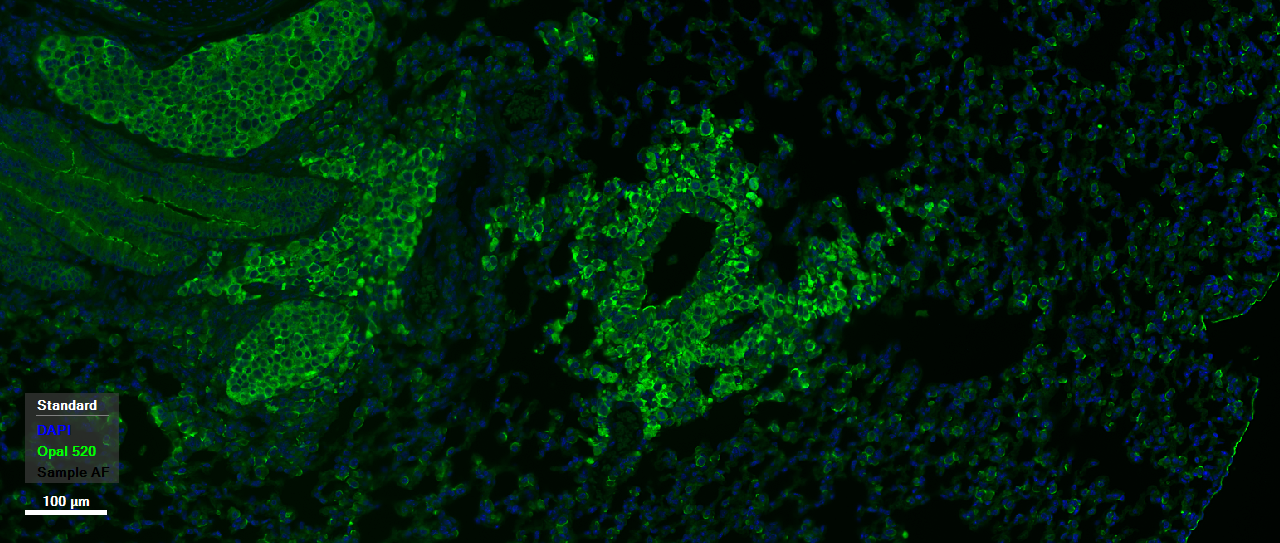

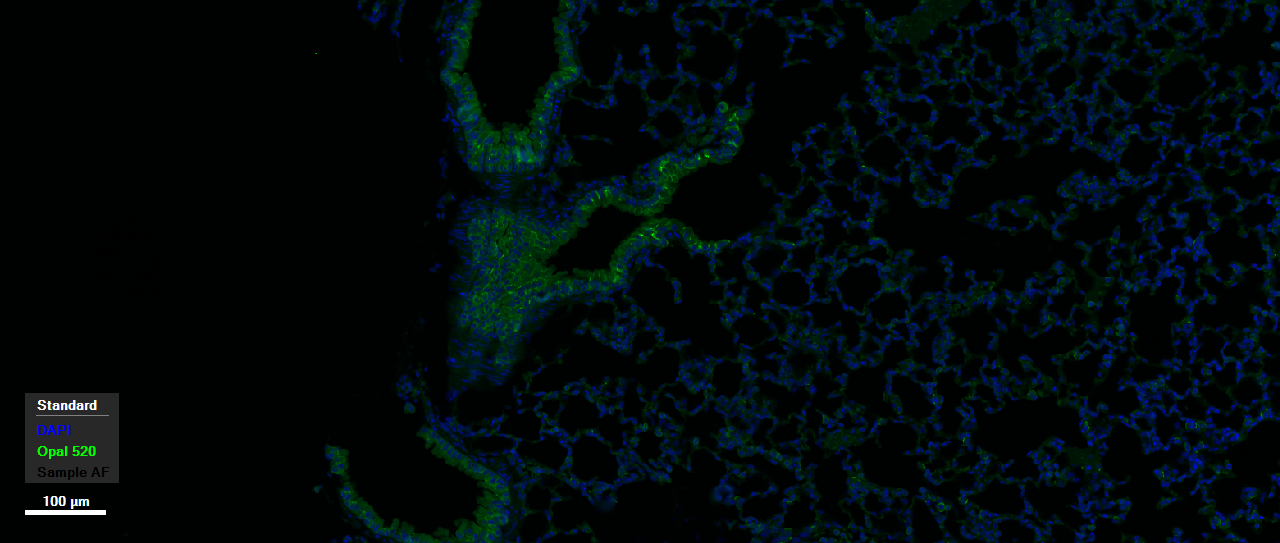

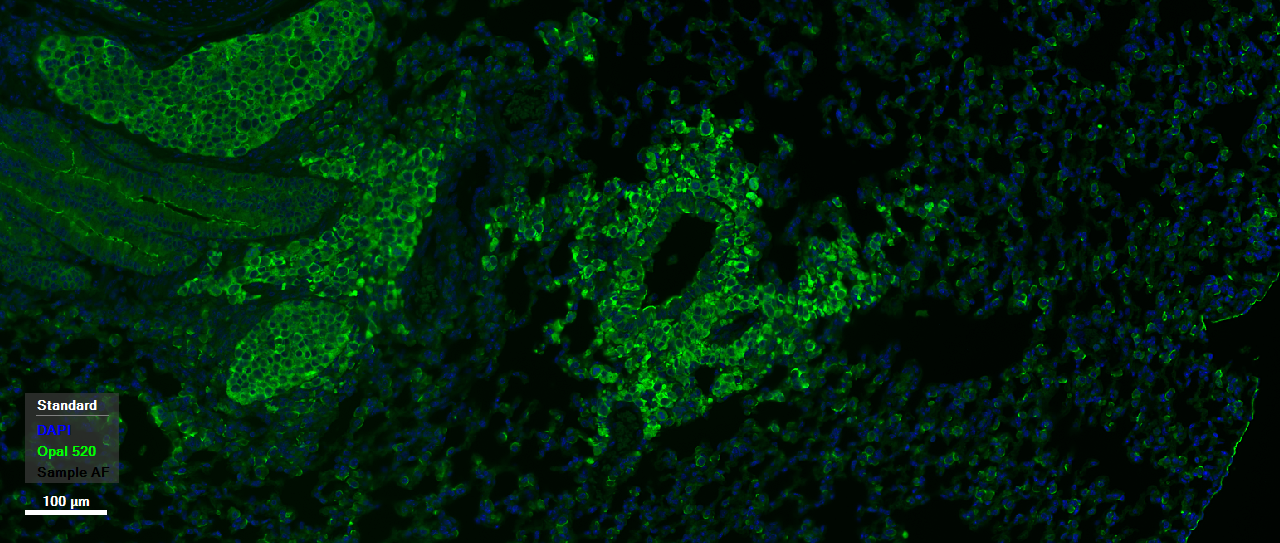


**SNU638 ΔYTHDF3**

**SNU638 mock**

**Supplementary Fig. S4**. **A.** Immunofluorescence staining for phosphorylated EZR and F-actin in AGS and SNU638 mock and KO cell lines and visualized by confocal microscopy in the same cell lines. Nuclei are stained with DAPI. The scale bar corresponds to 20 μm and images were acquired with a 40x objective. **B.** Immunofluorescence staining for EZR in the mouse metastasis obtained from SNU638 mock and SNU638 *ΔYTHDF3*. Nuclei are stained with DAPI. Scale bar corresponds to 100 μm.

**Supplementary Fig. 5**

**
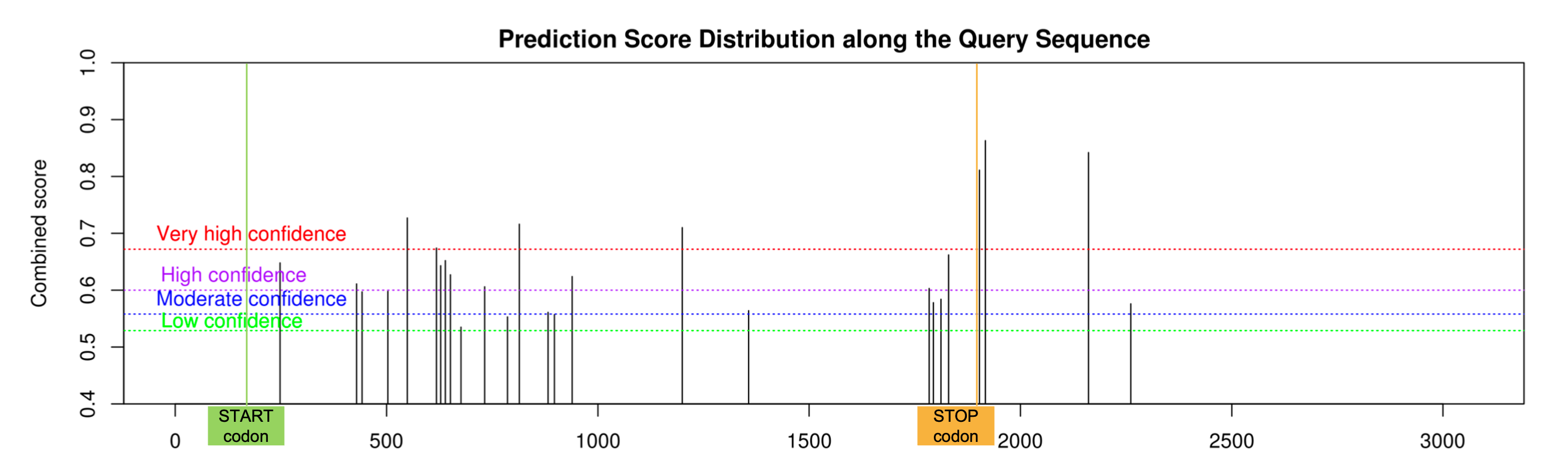
**

**
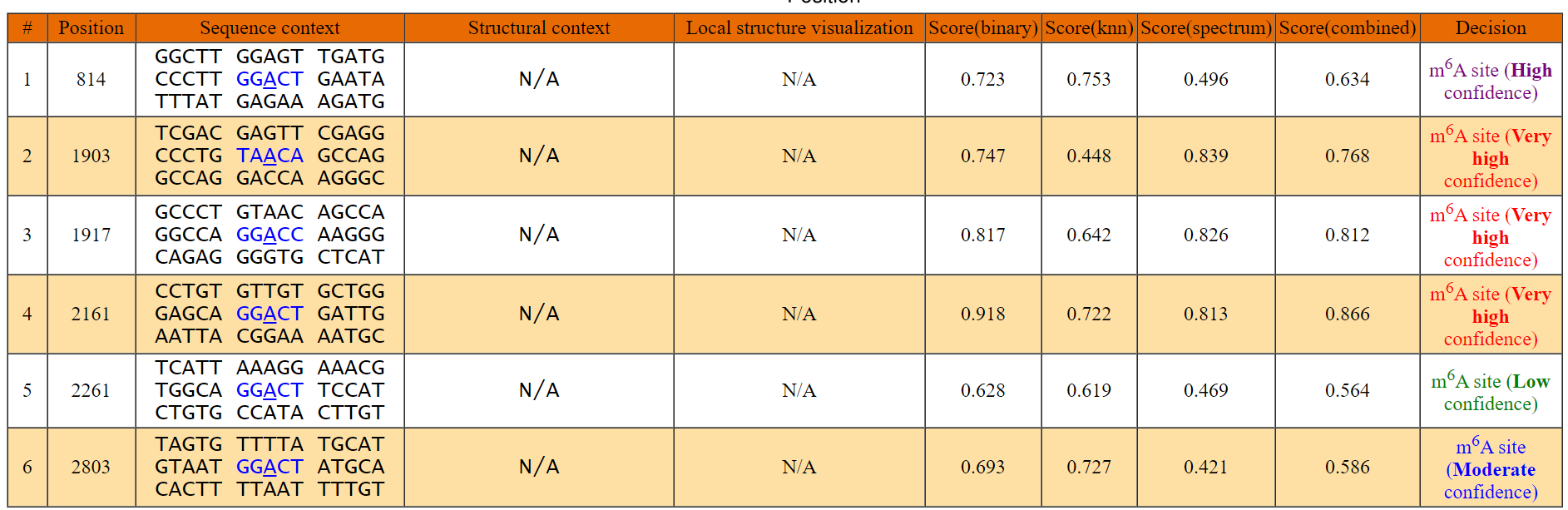
**

**Supplementary Fig. S5**. Prediction of m6A sites in Ezrin transcript with the Sequence-based RNA adenosine methylation site predictor SRAMP (http://www.cuilab.cn/sramp). Arrows indicate the two of the most probable m6A sites analysed.
